# Supplementary figures and images for: The genomic landscape of meiotic crossovers and gene conversions in Arabidopsis thaliana
Source: eLife. 2013 Dec 17;2:e01426. doi: 10.7554/eLife.01426 (PMC3865688; doi:10.7554/eLife.01426)

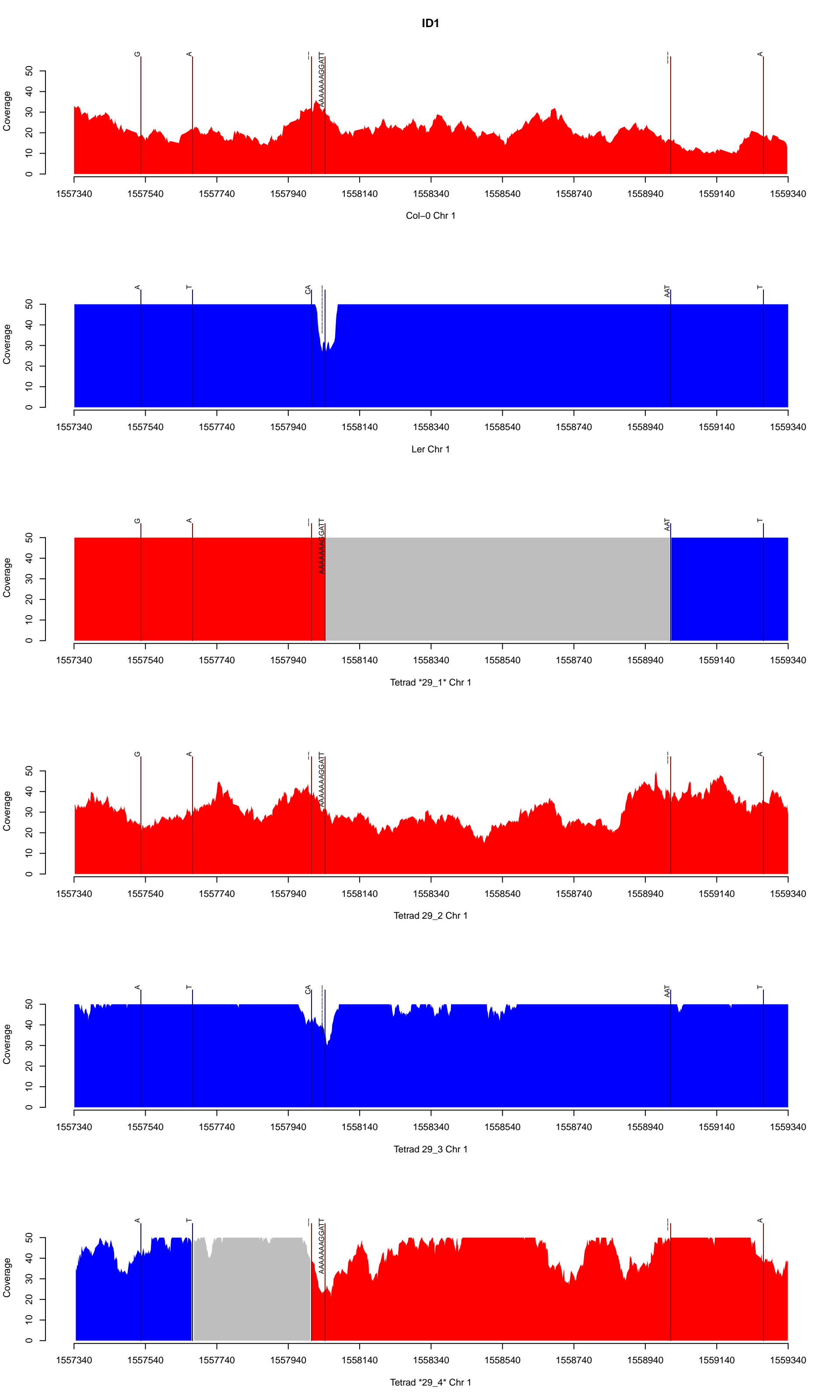

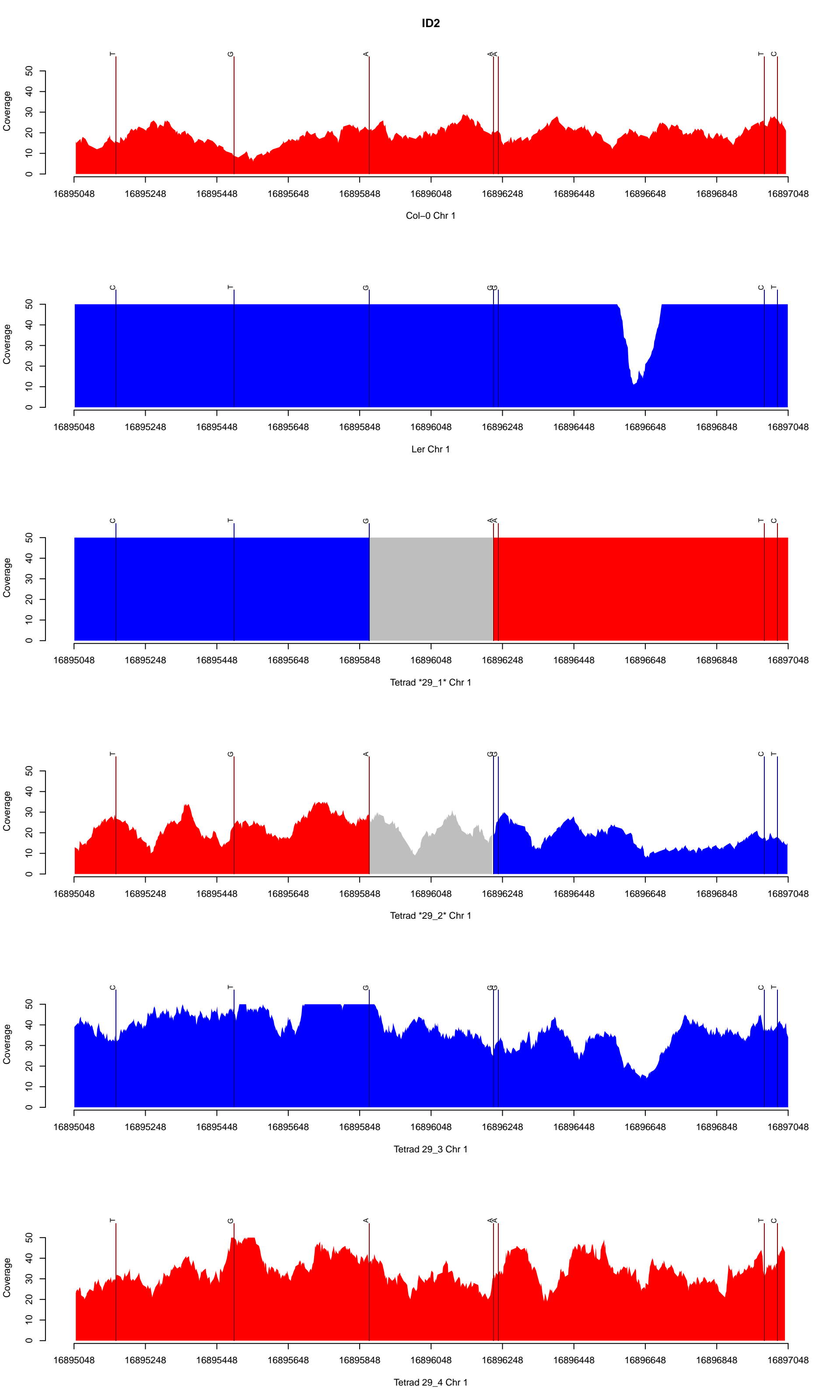

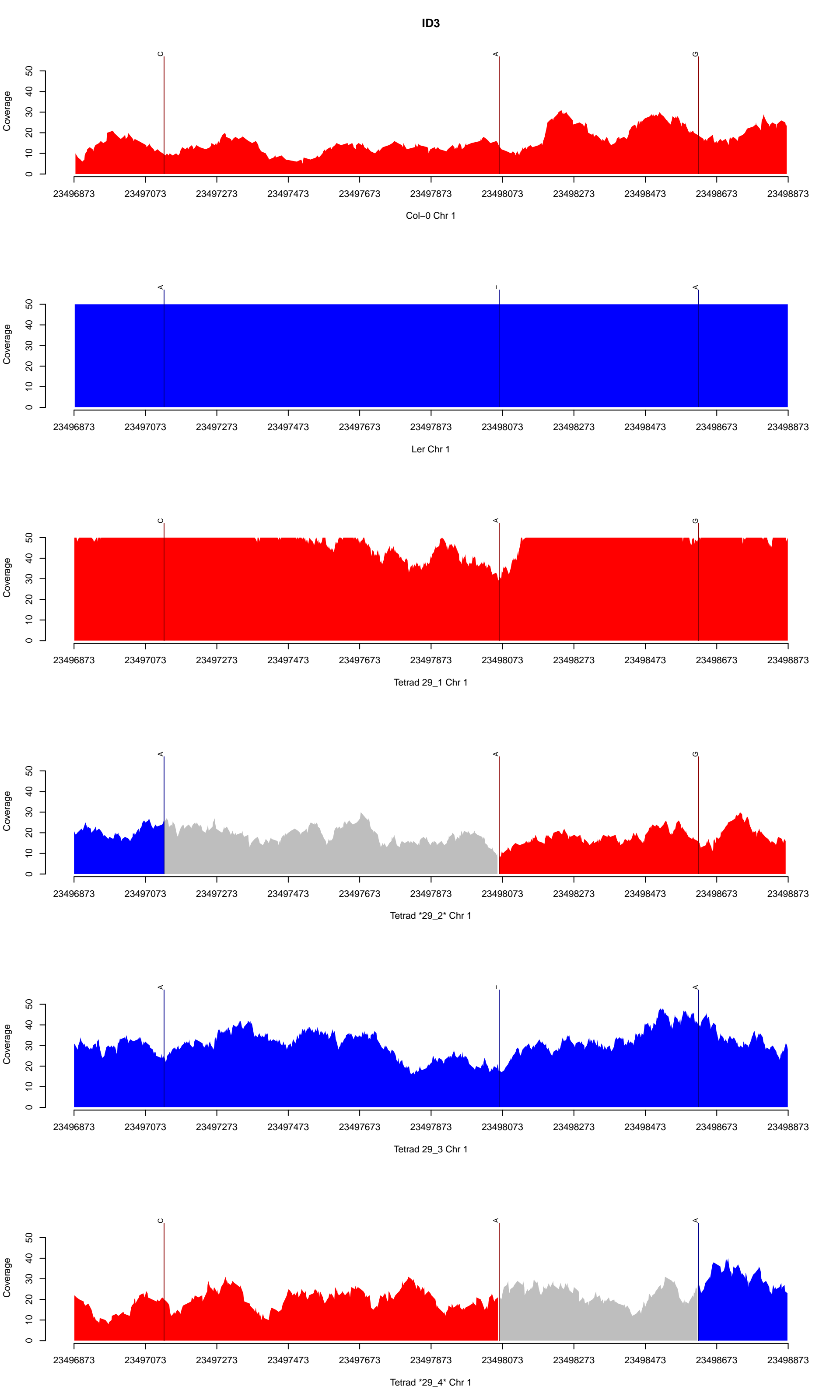

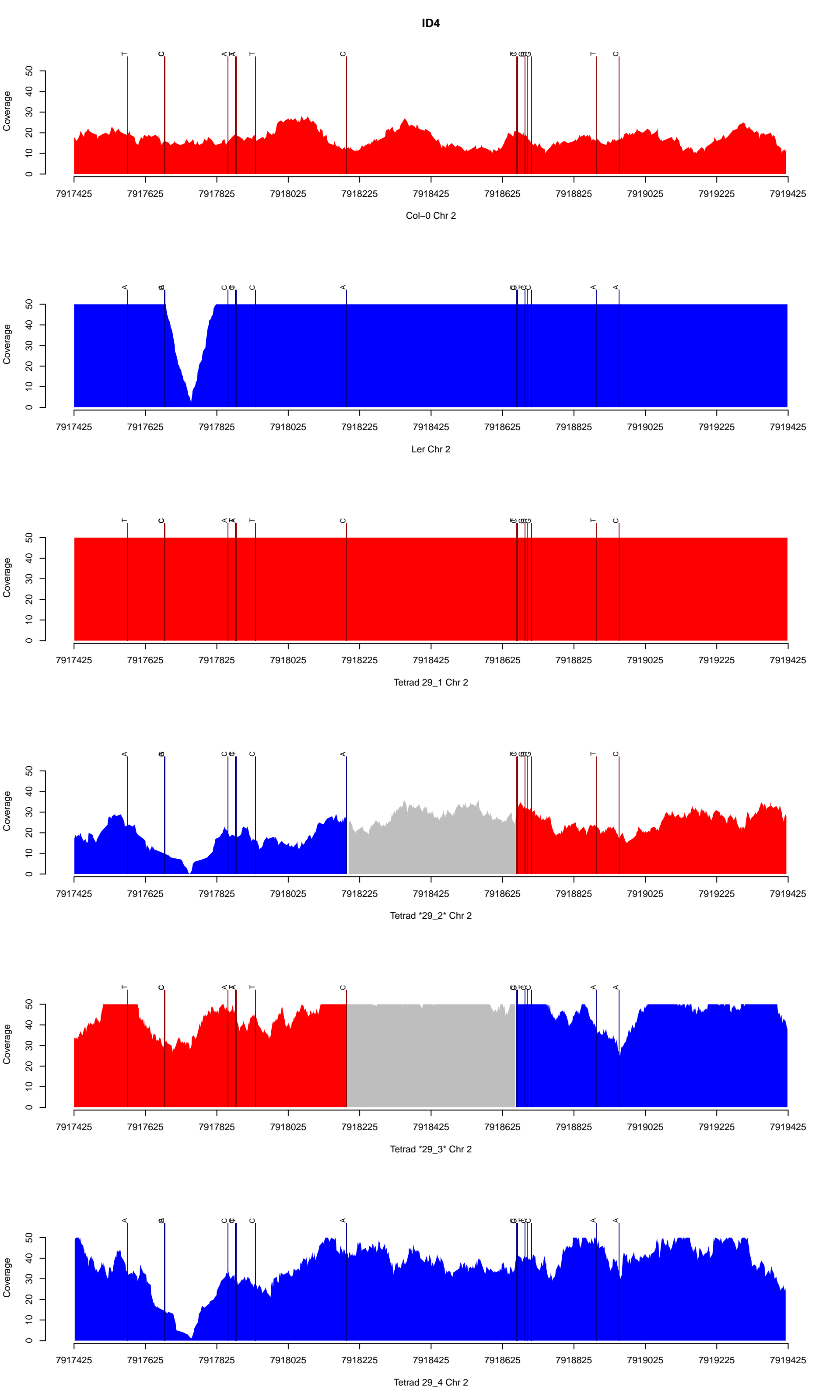

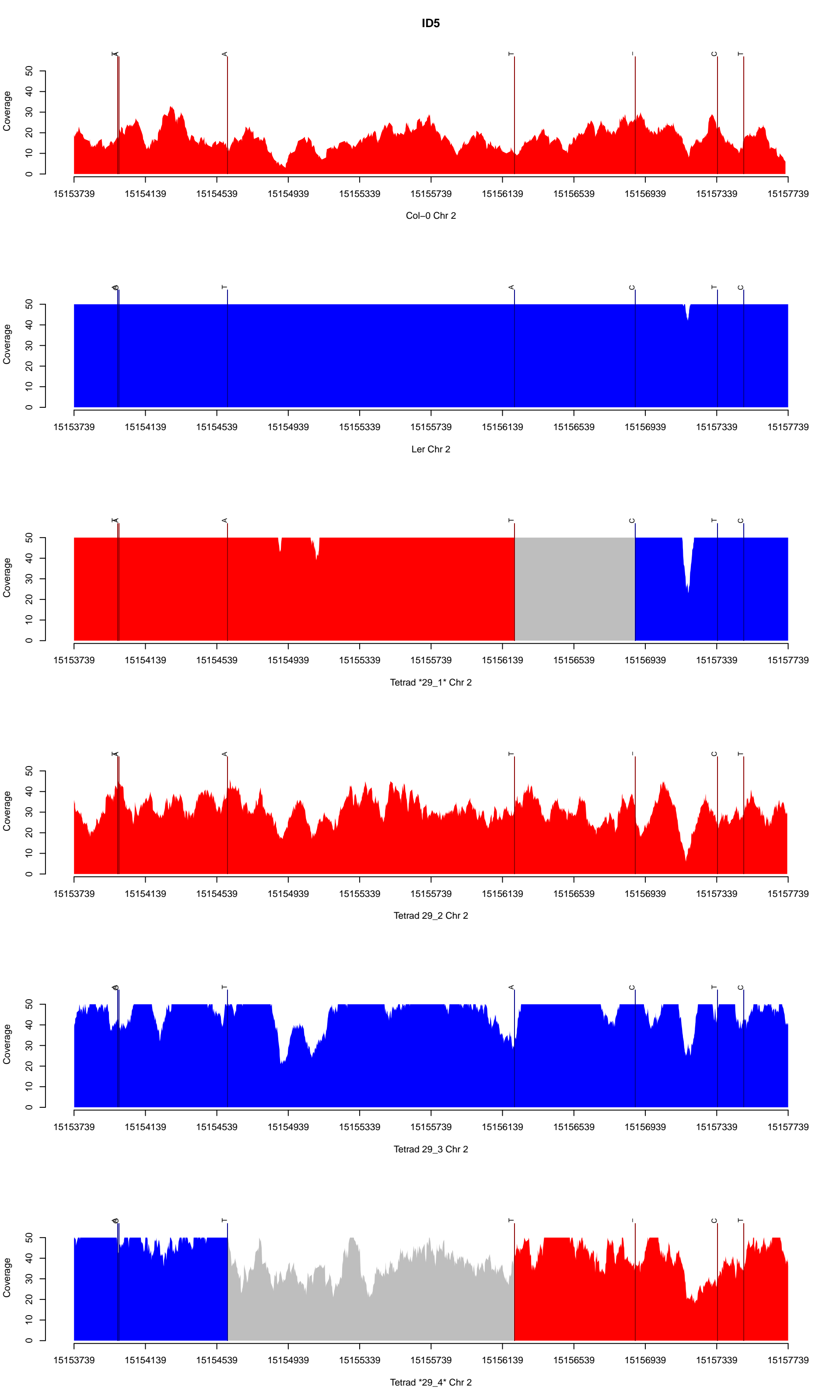

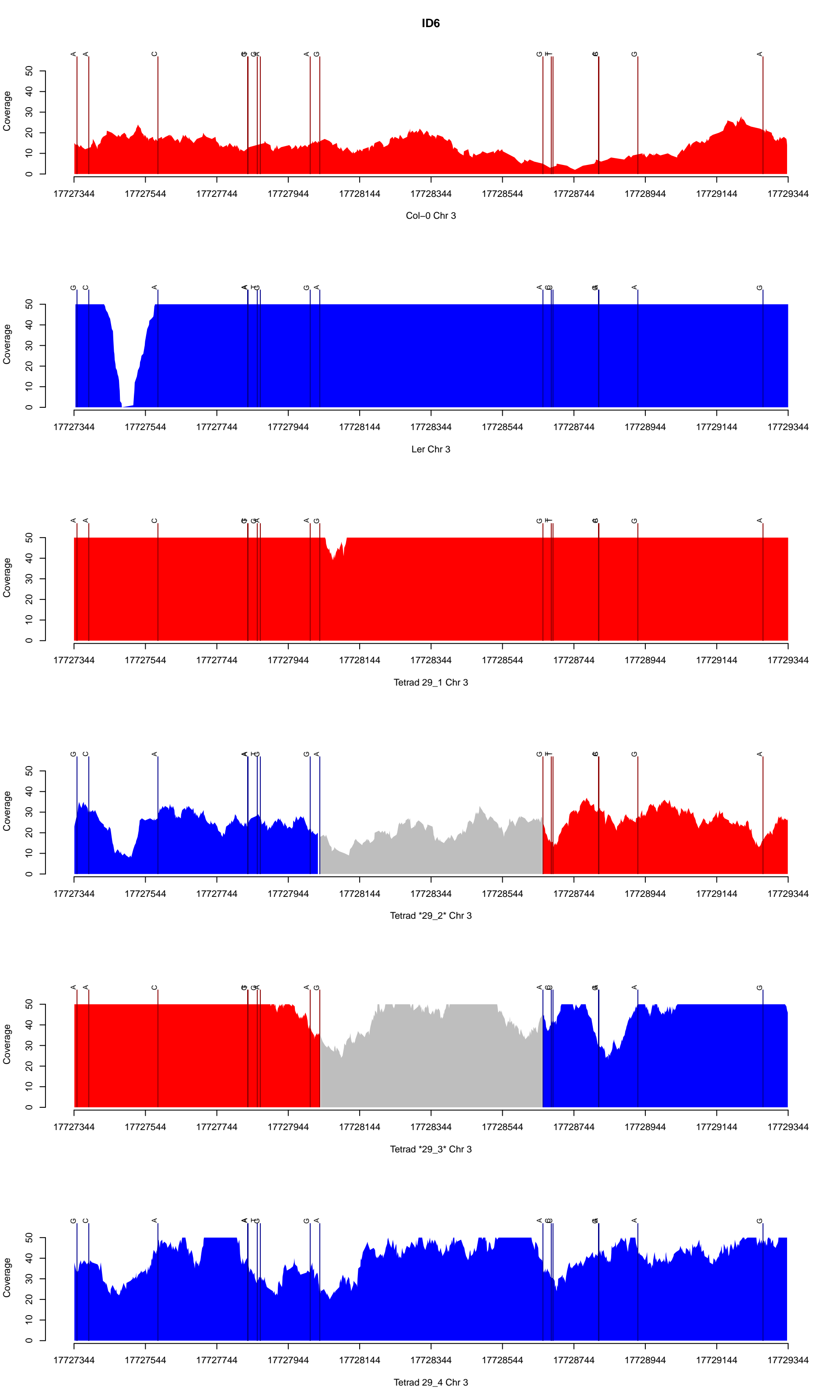

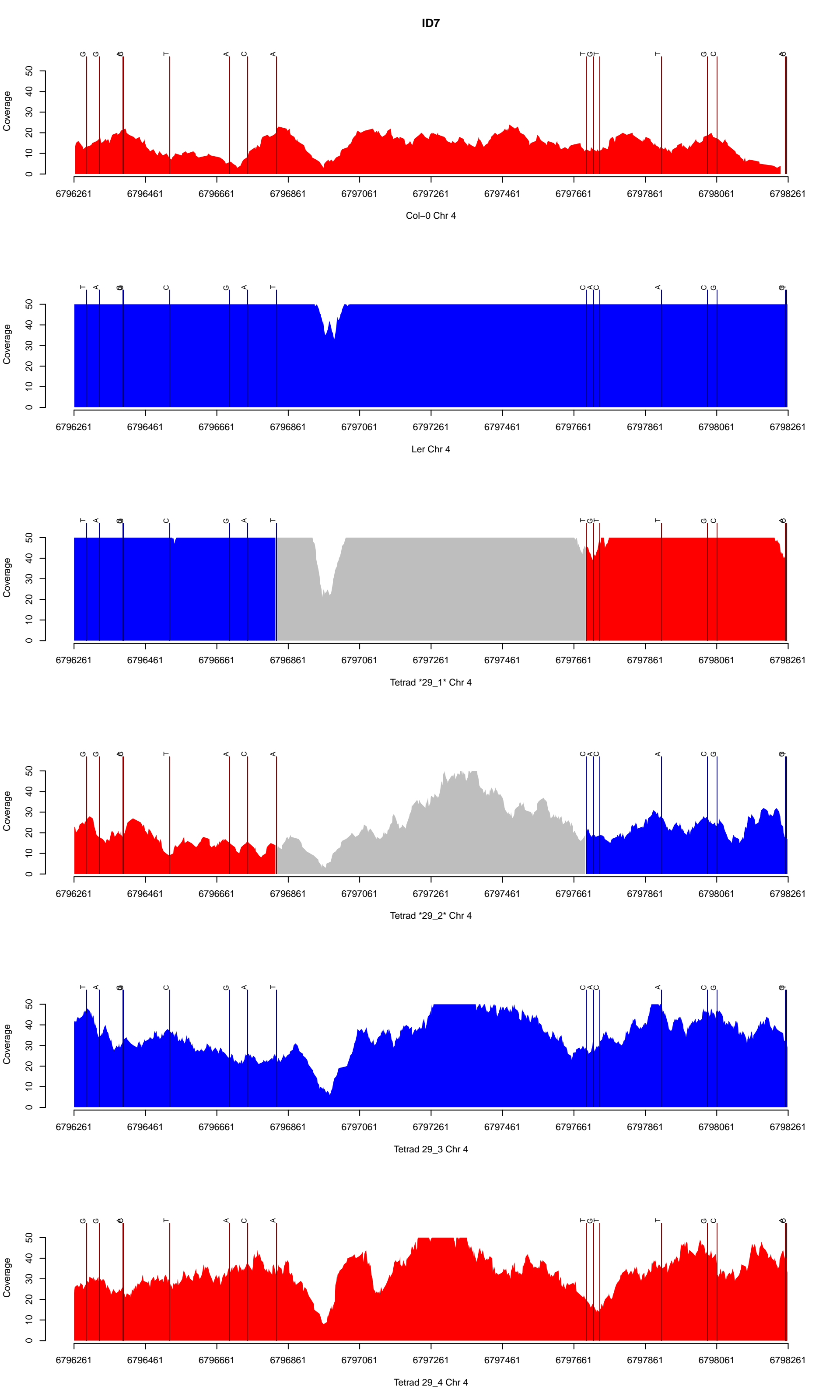

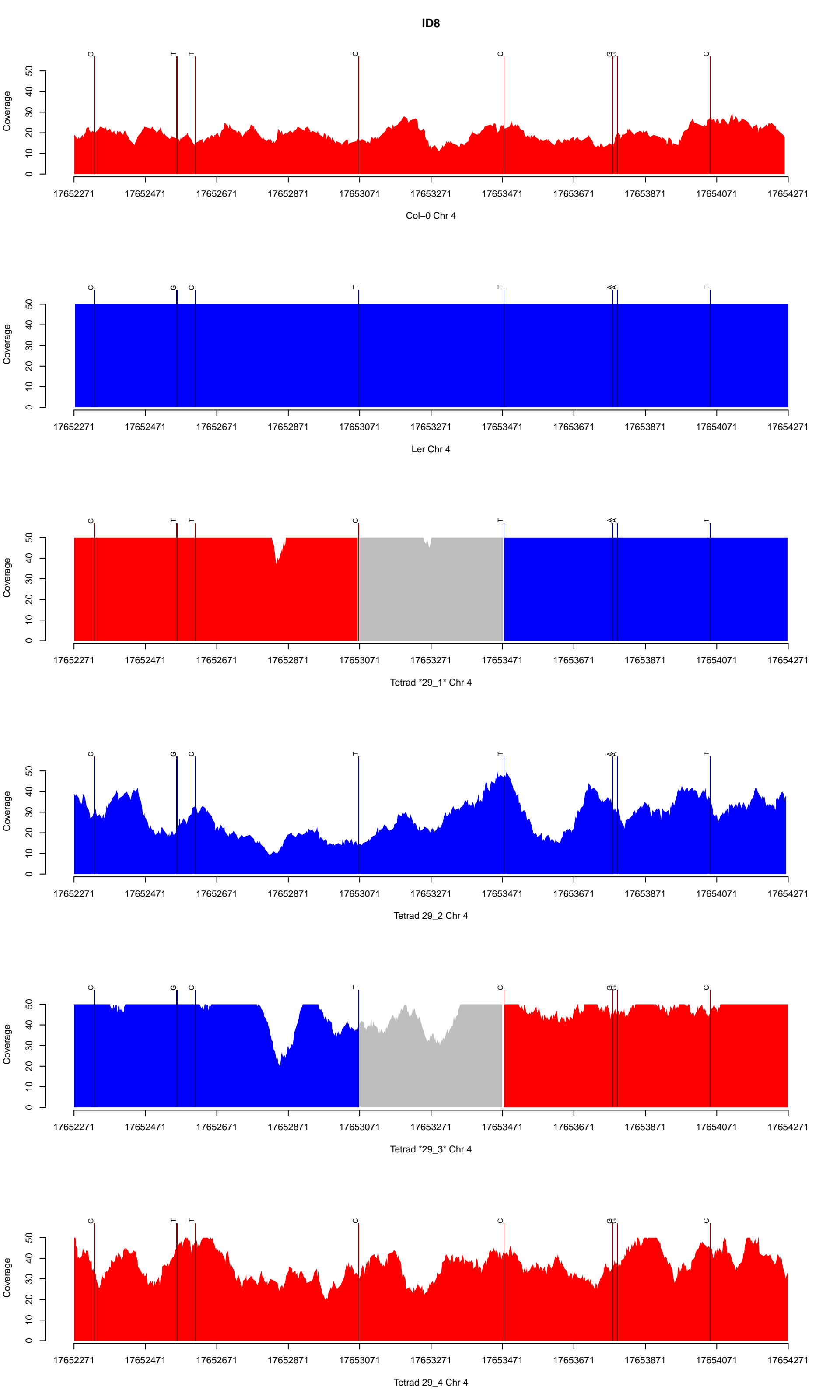

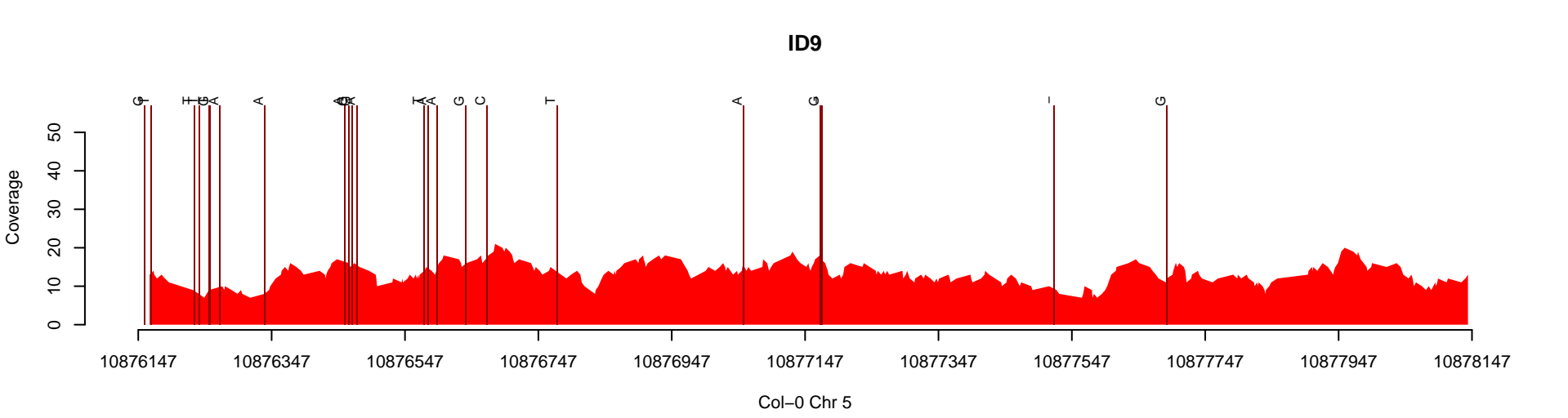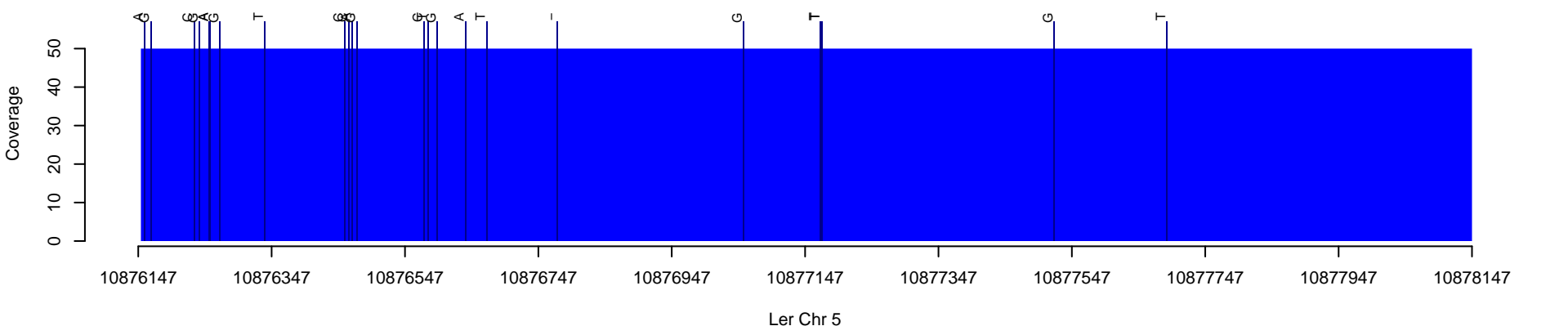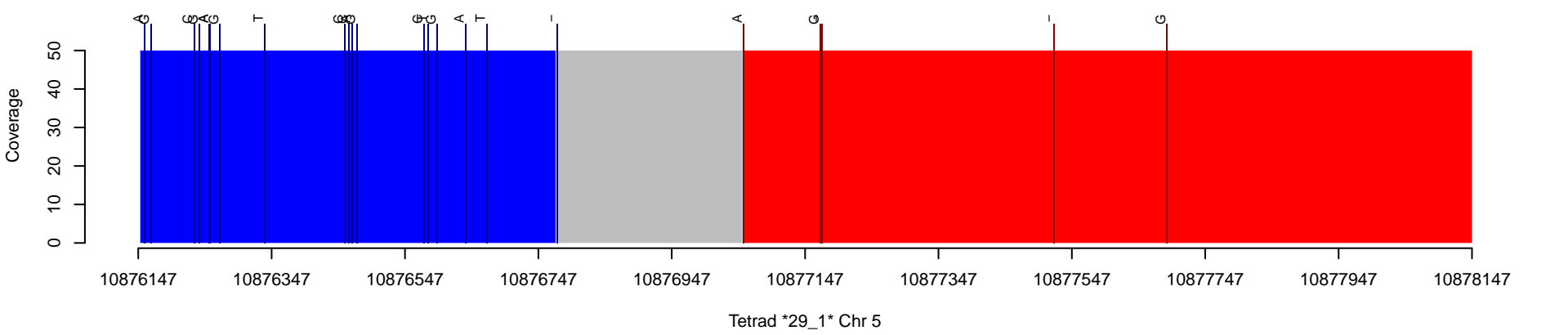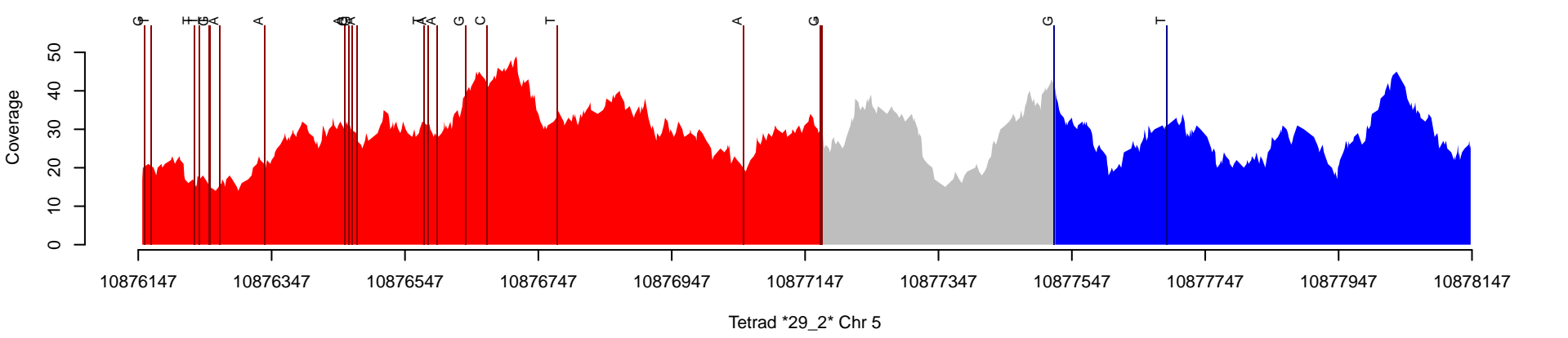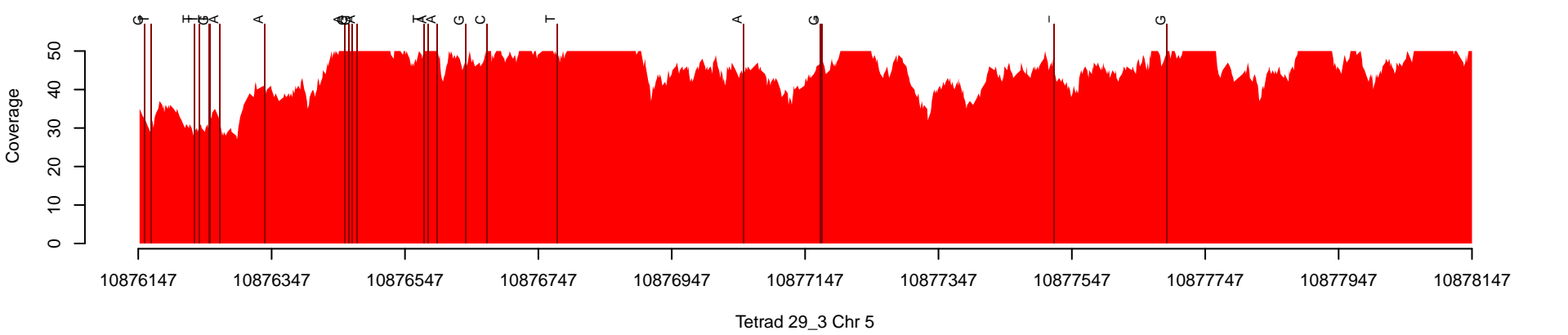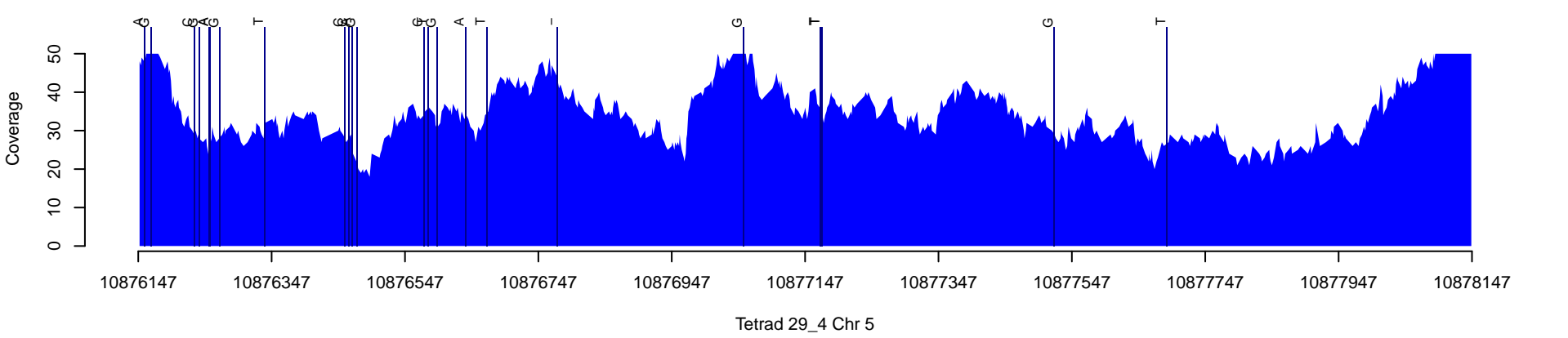

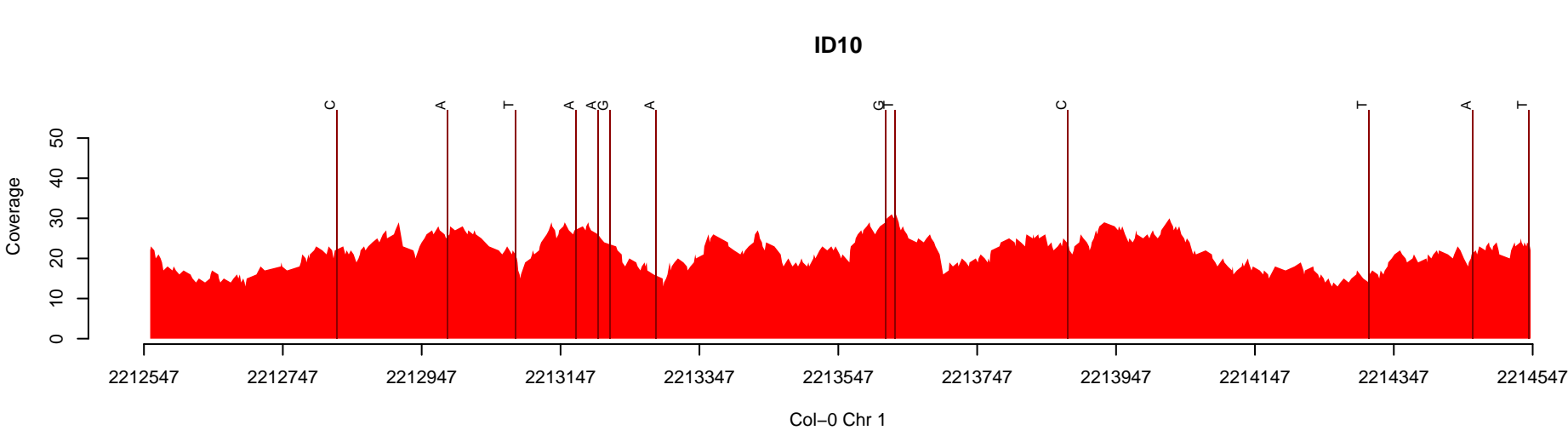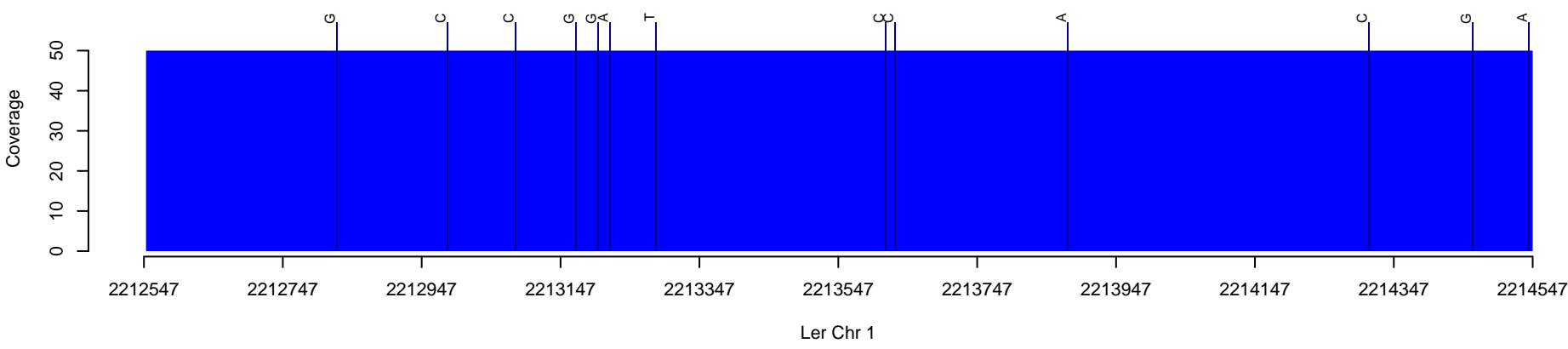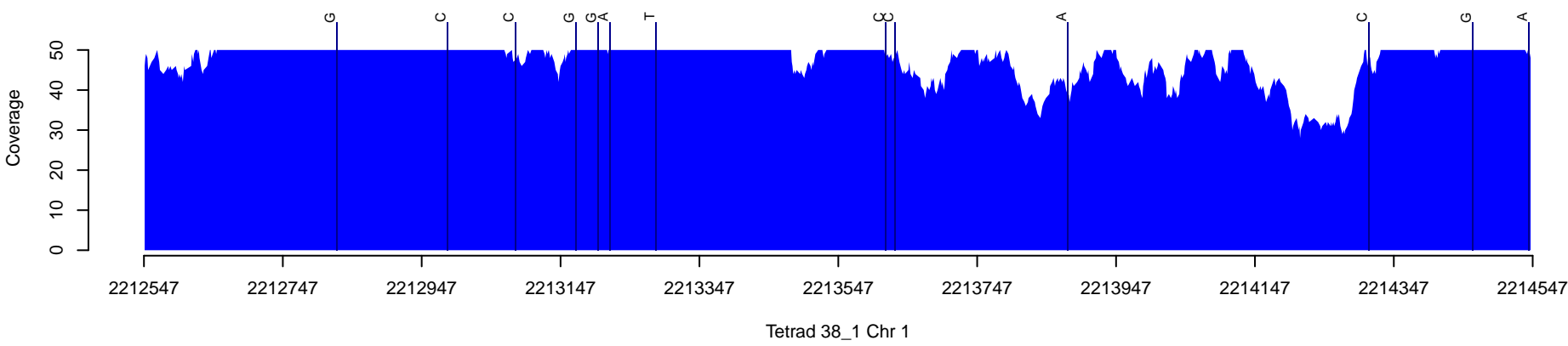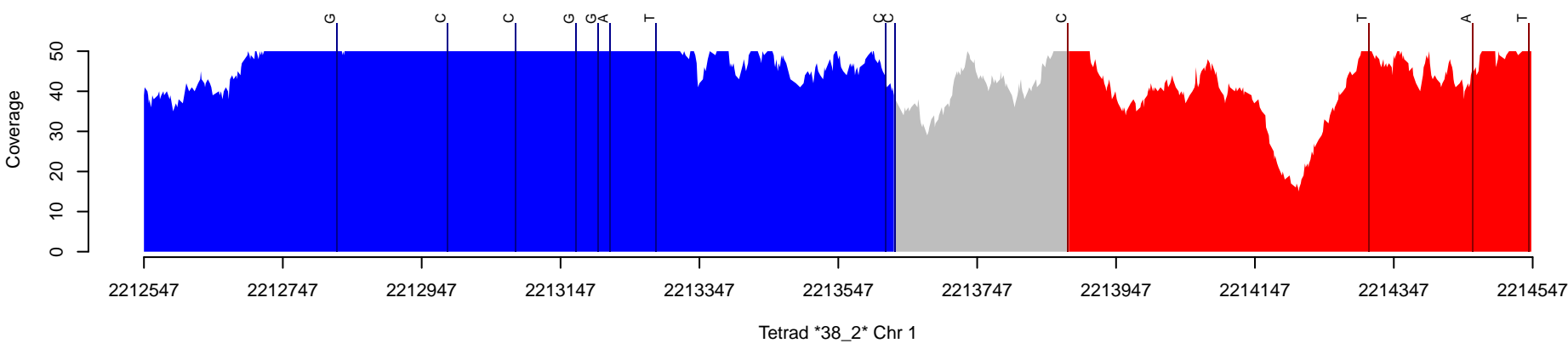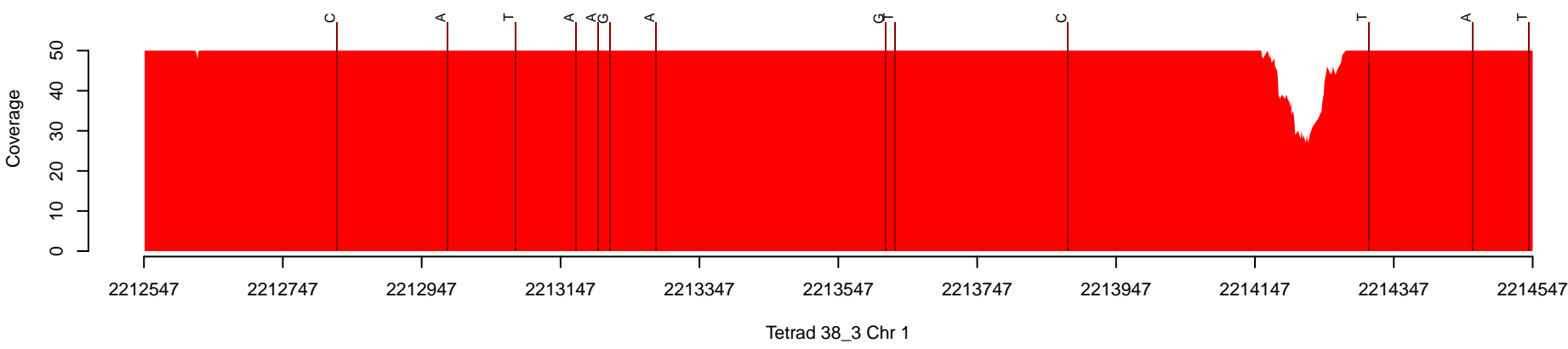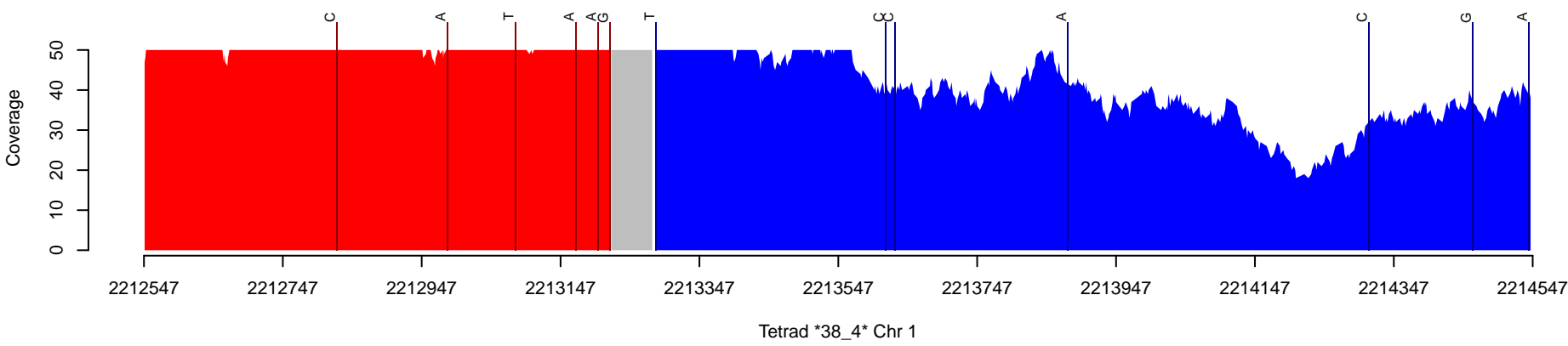

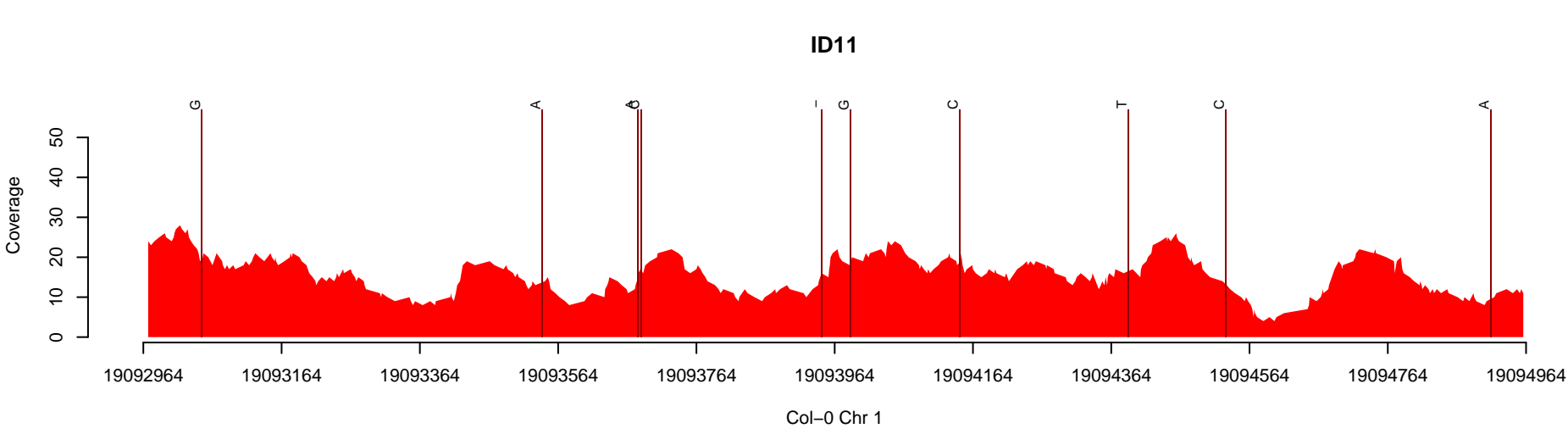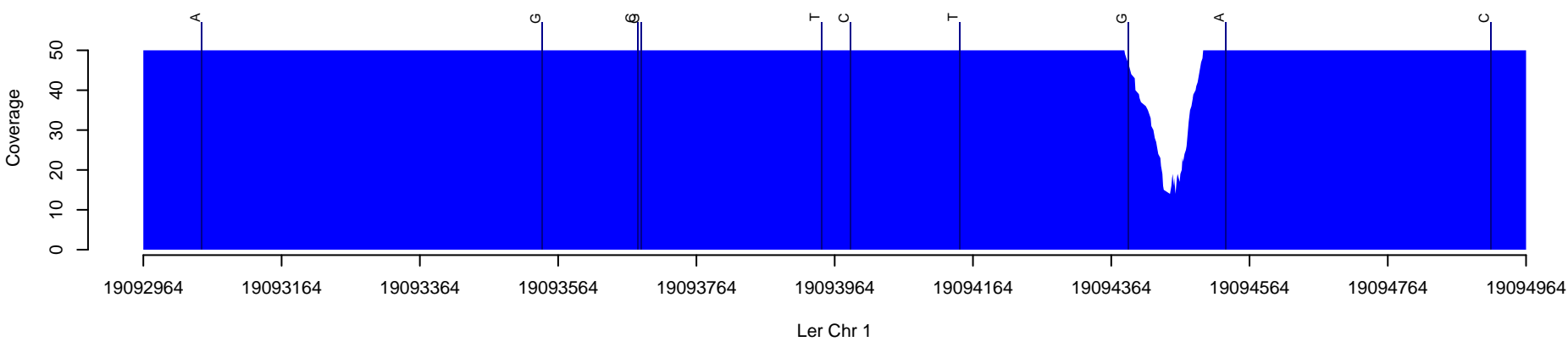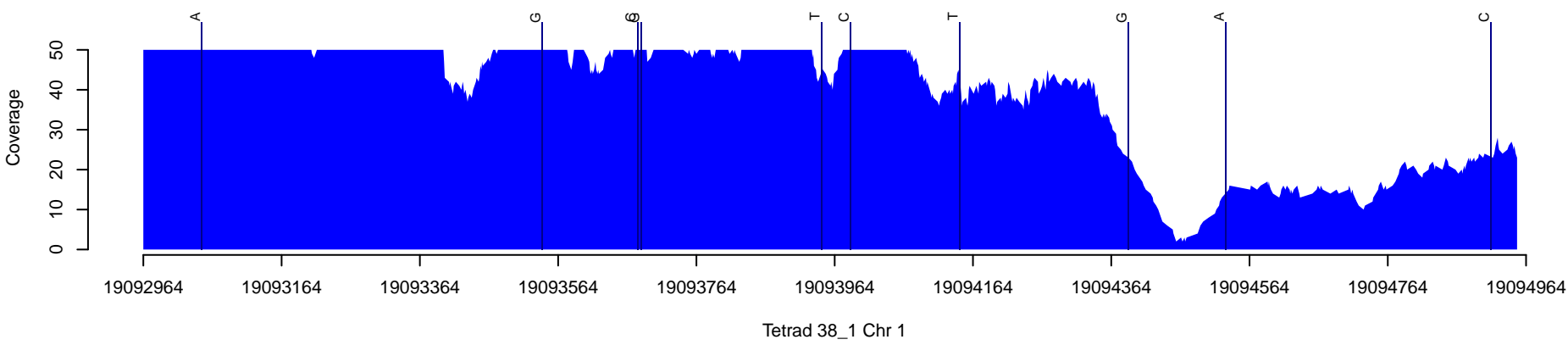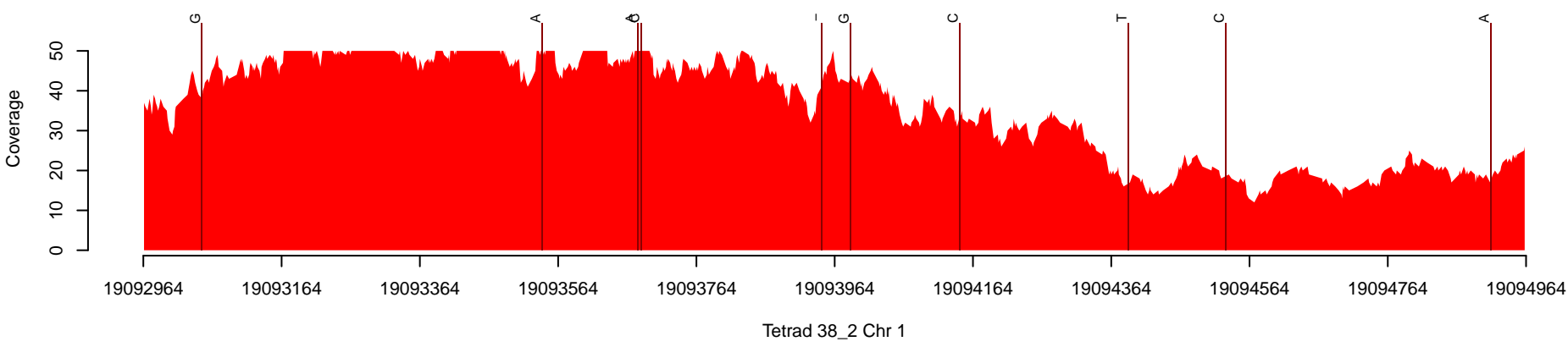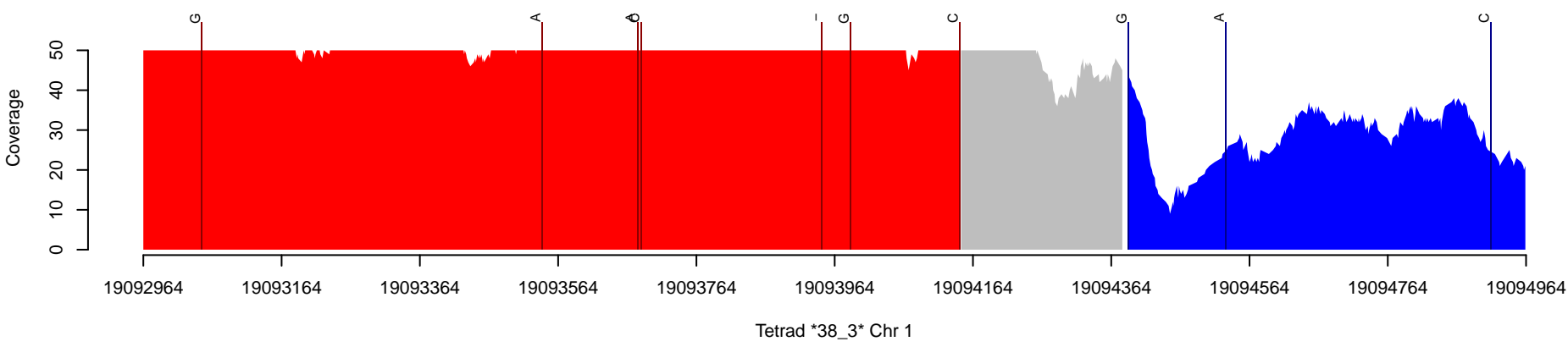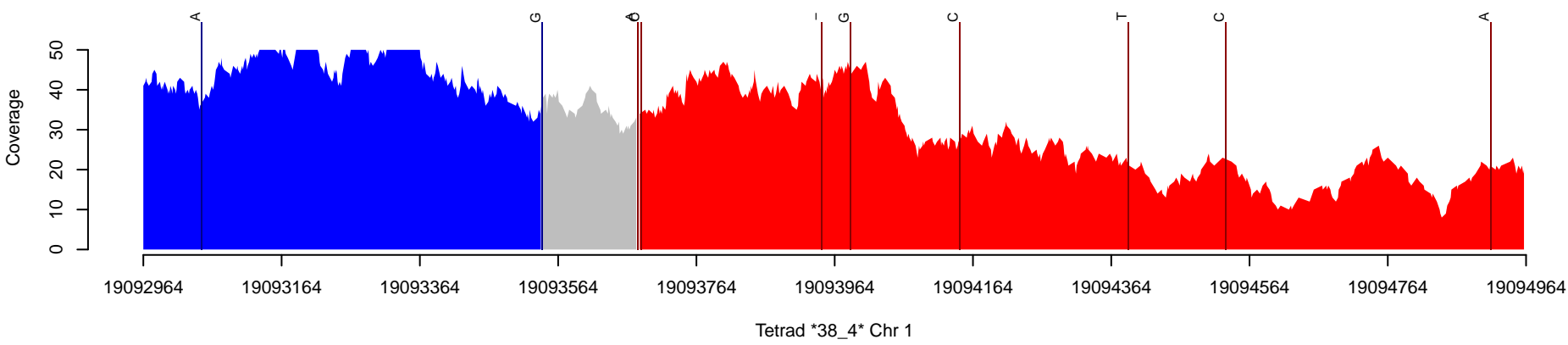

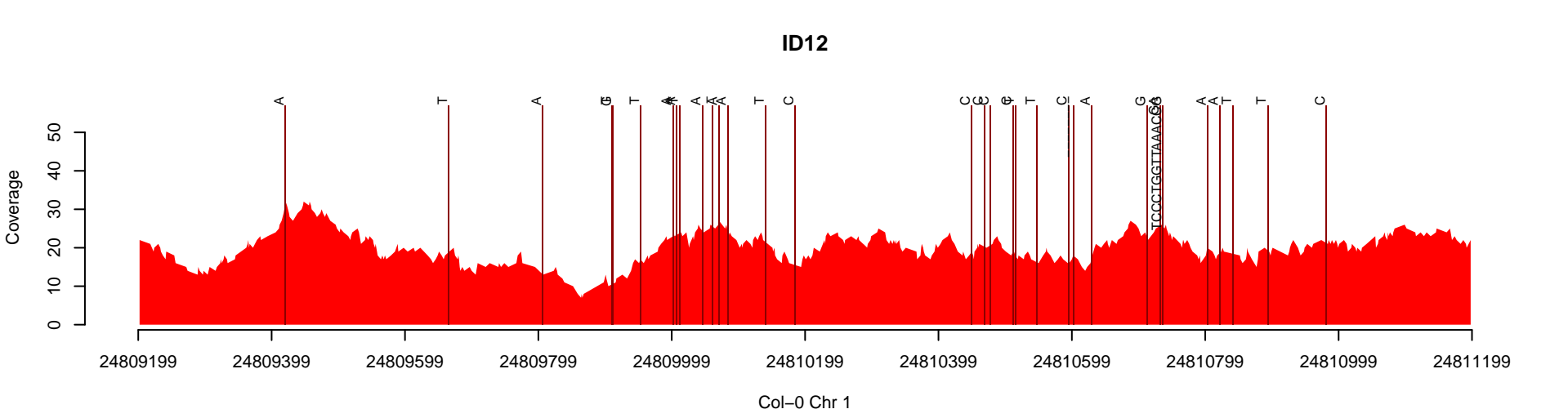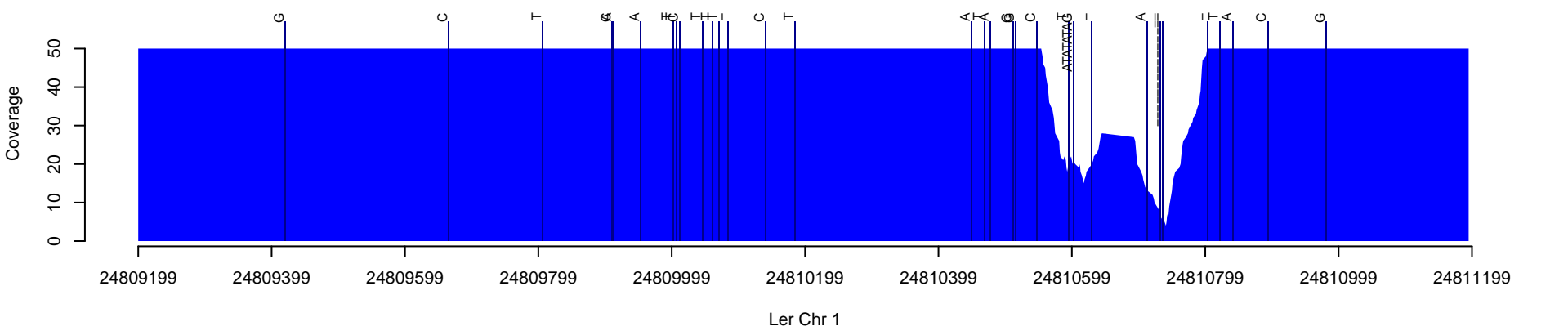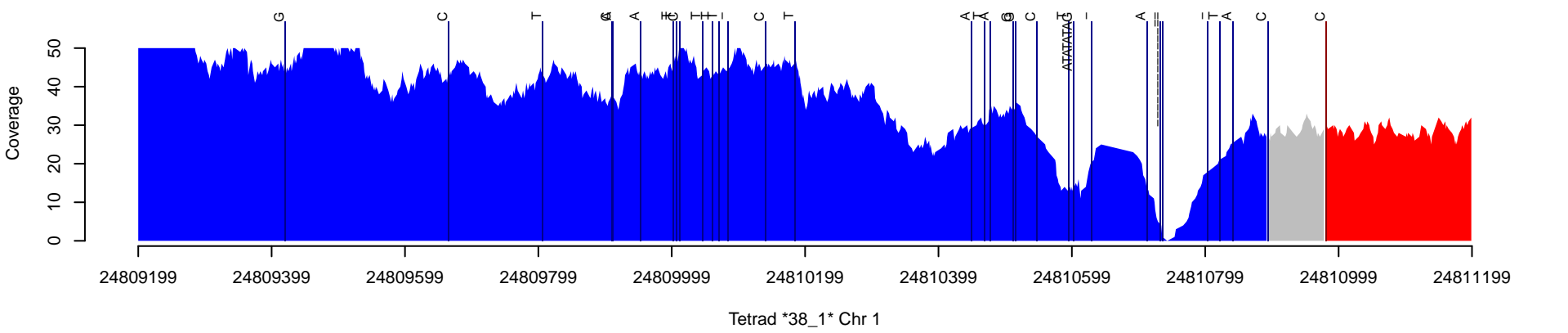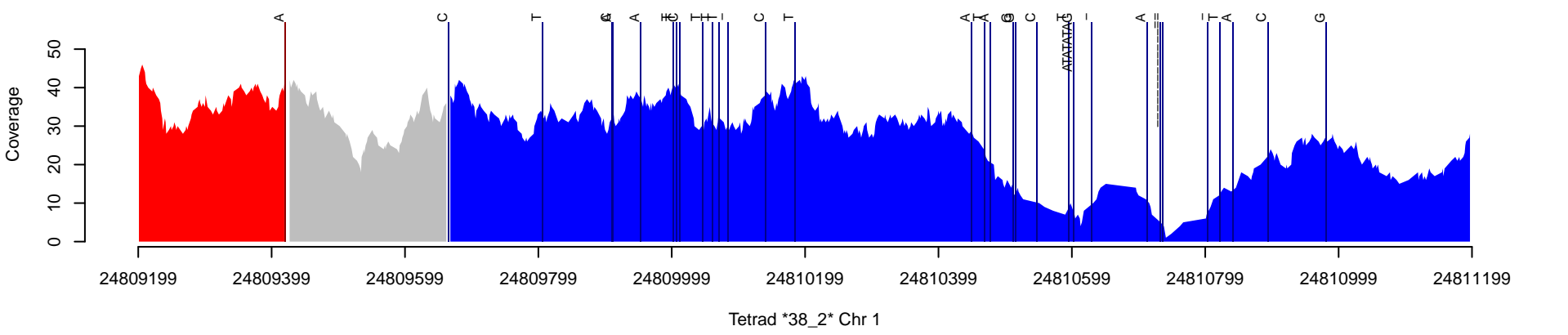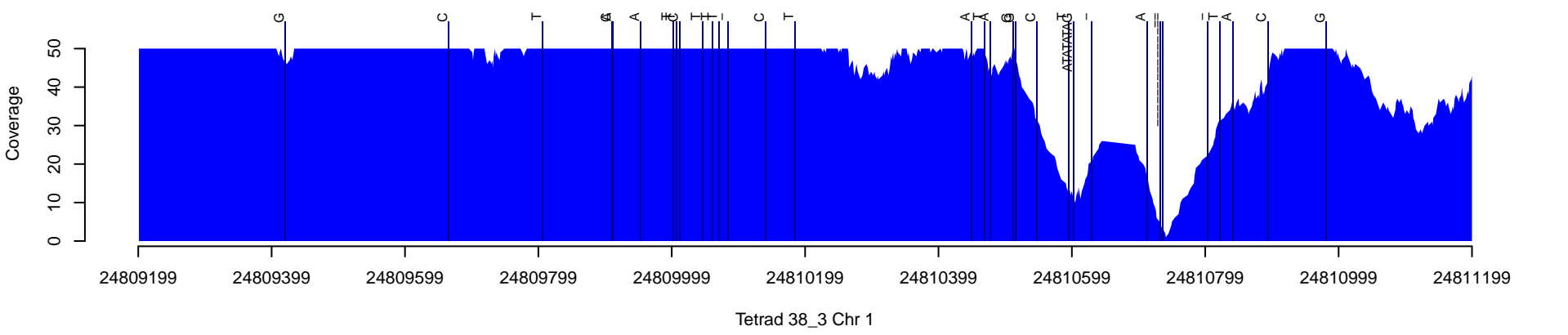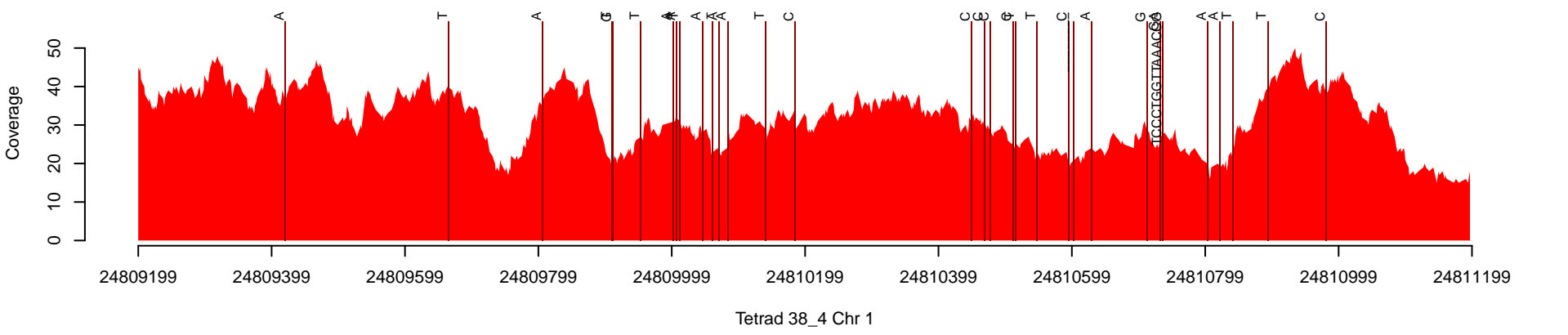

ID13

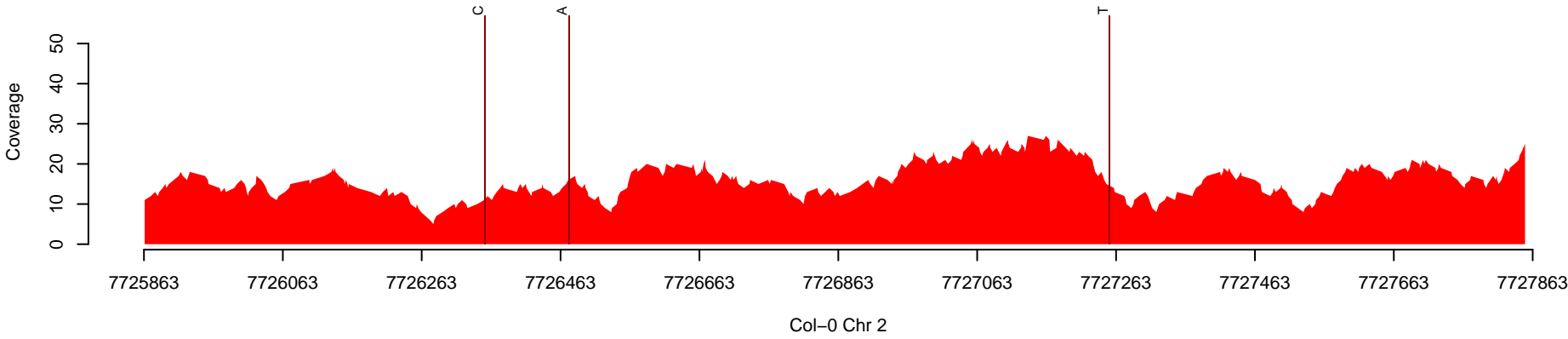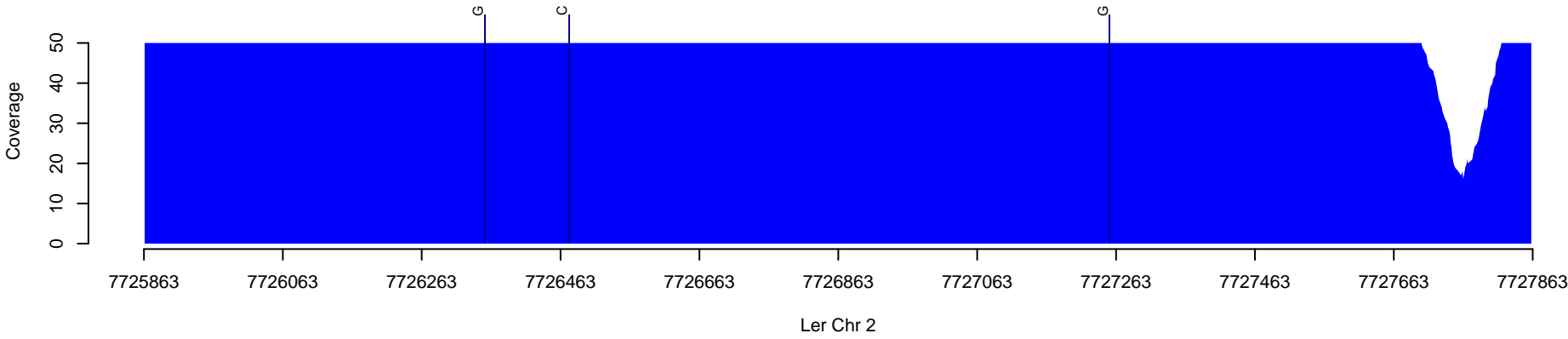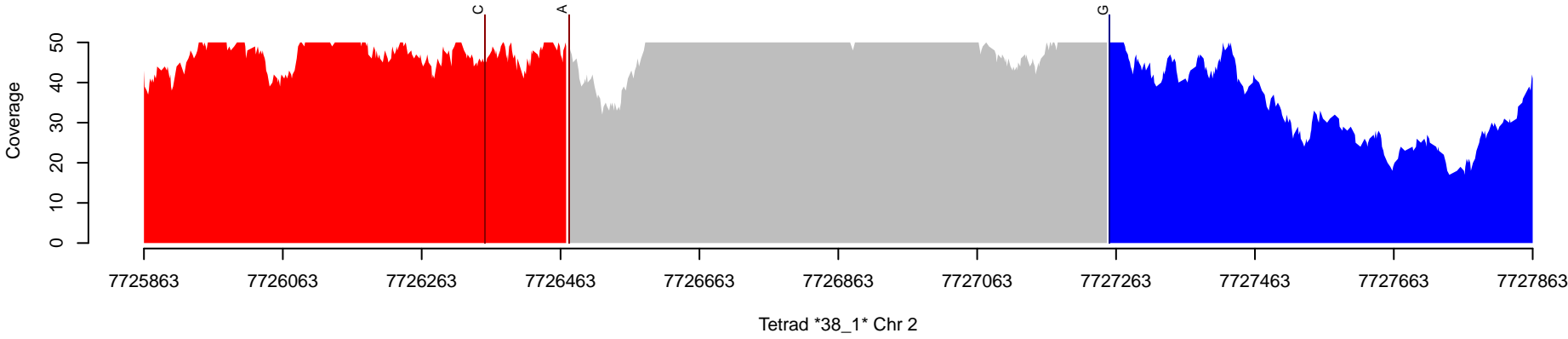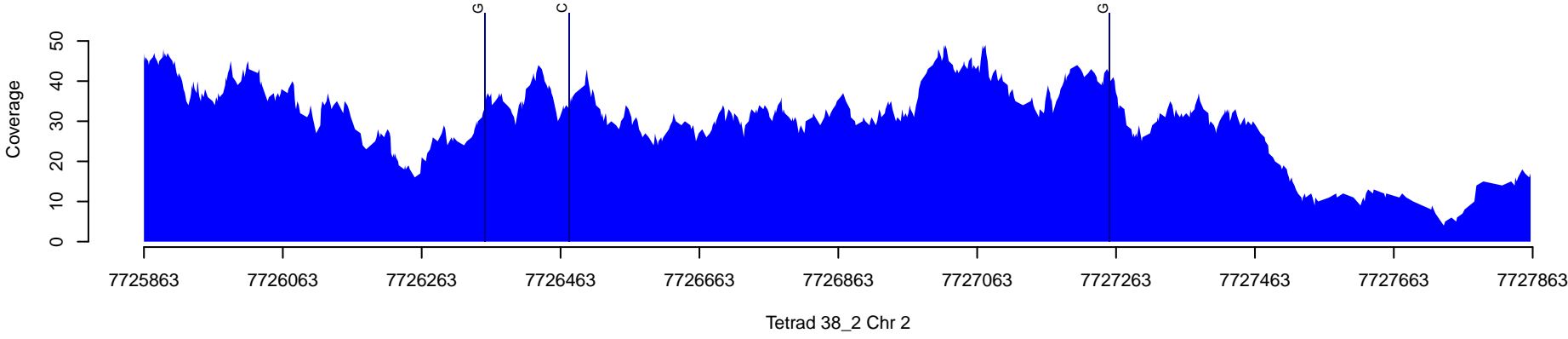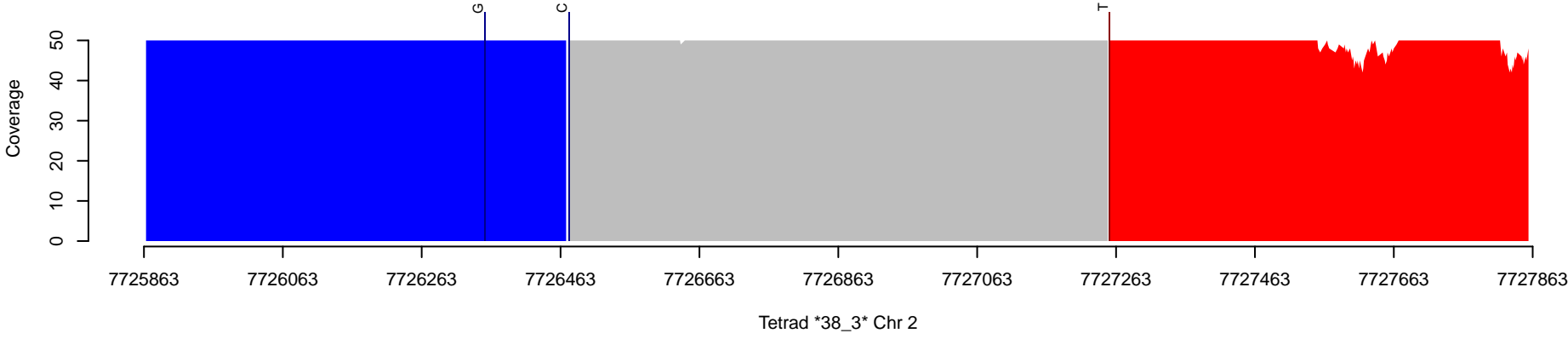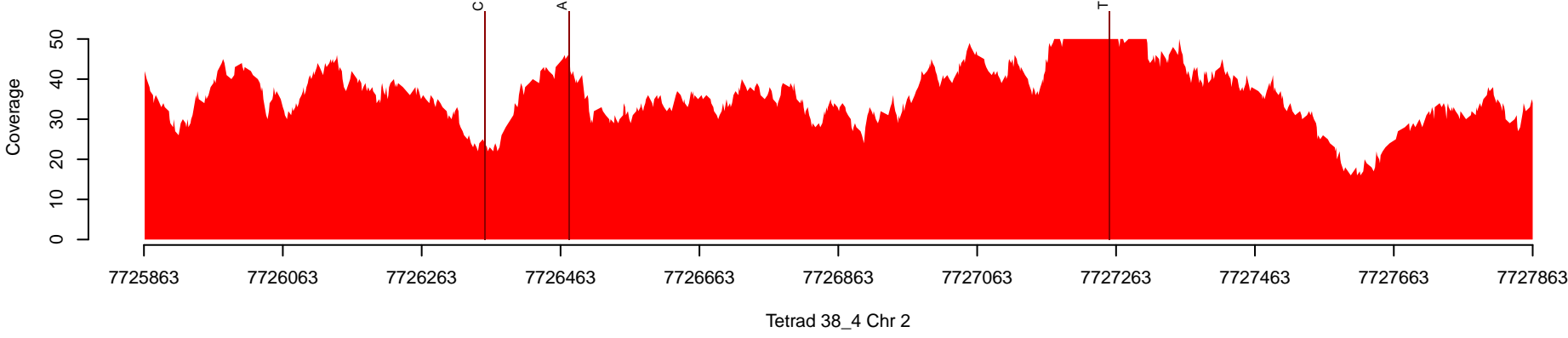

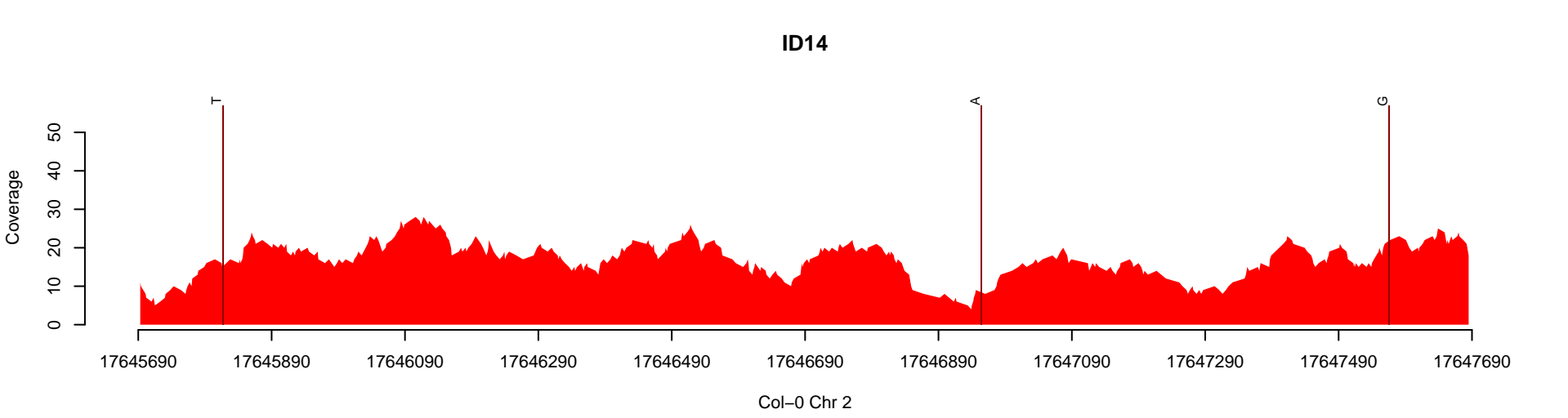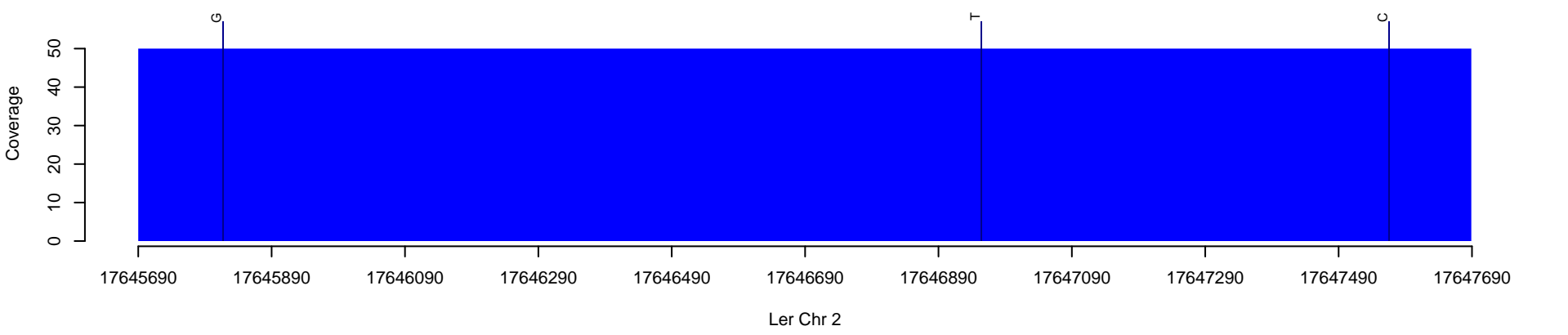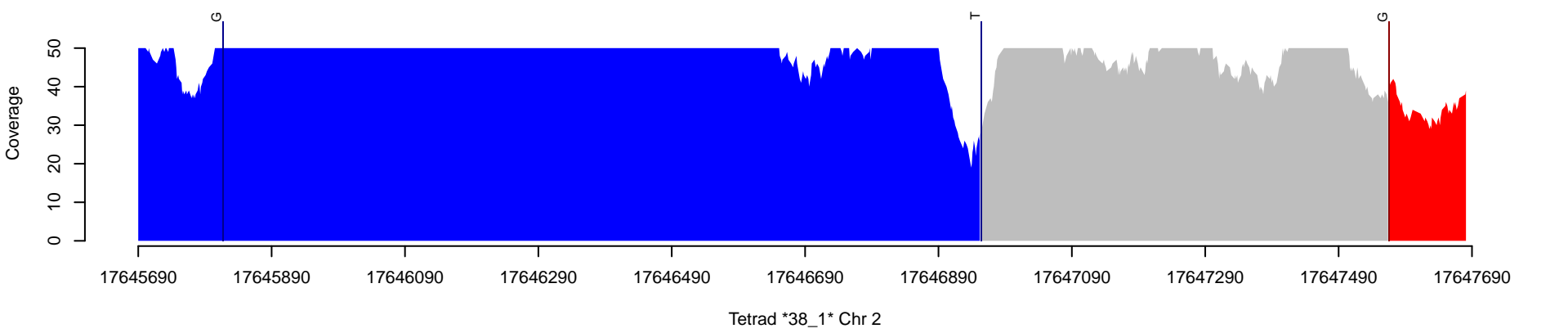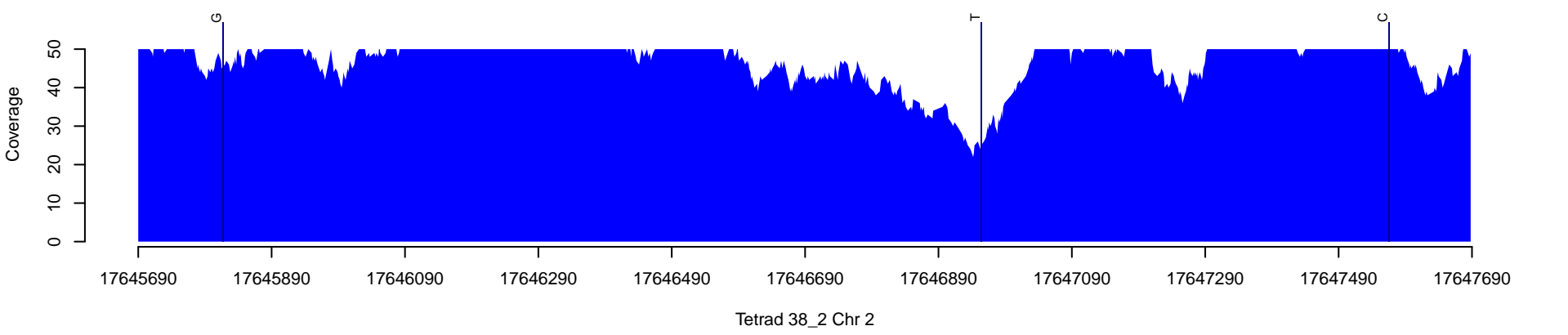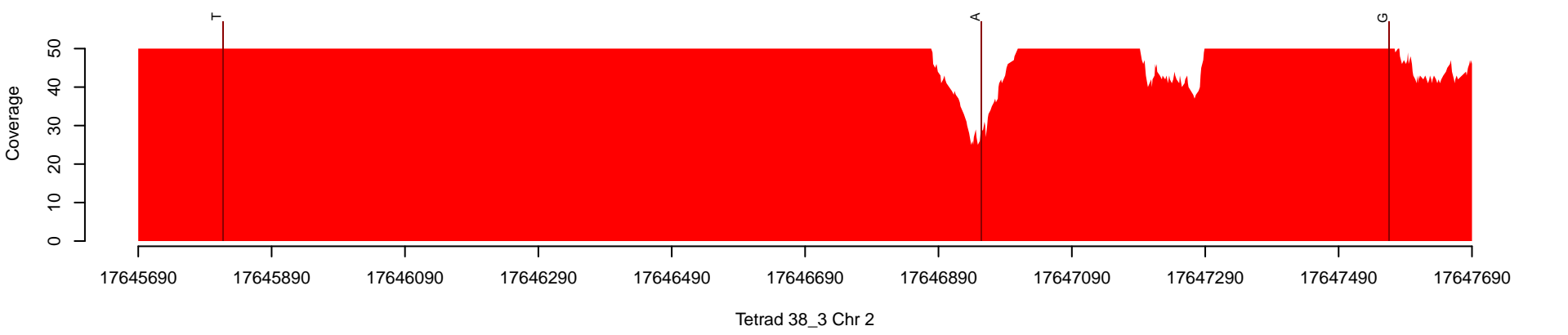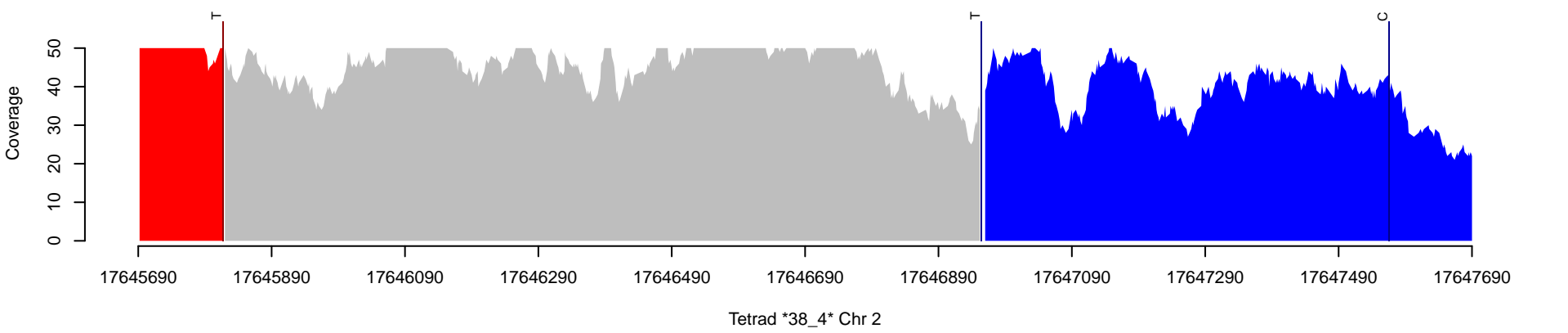

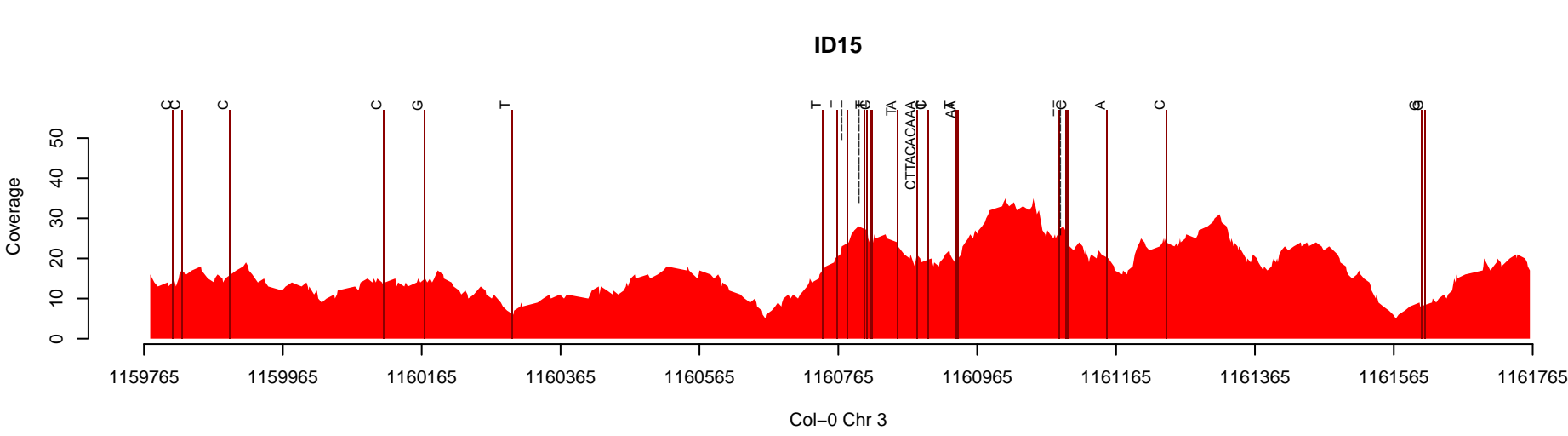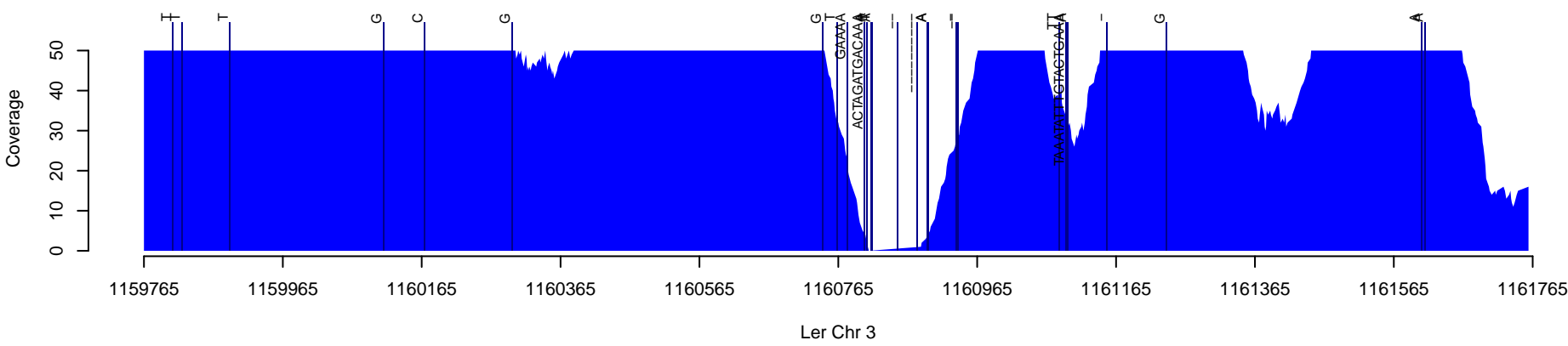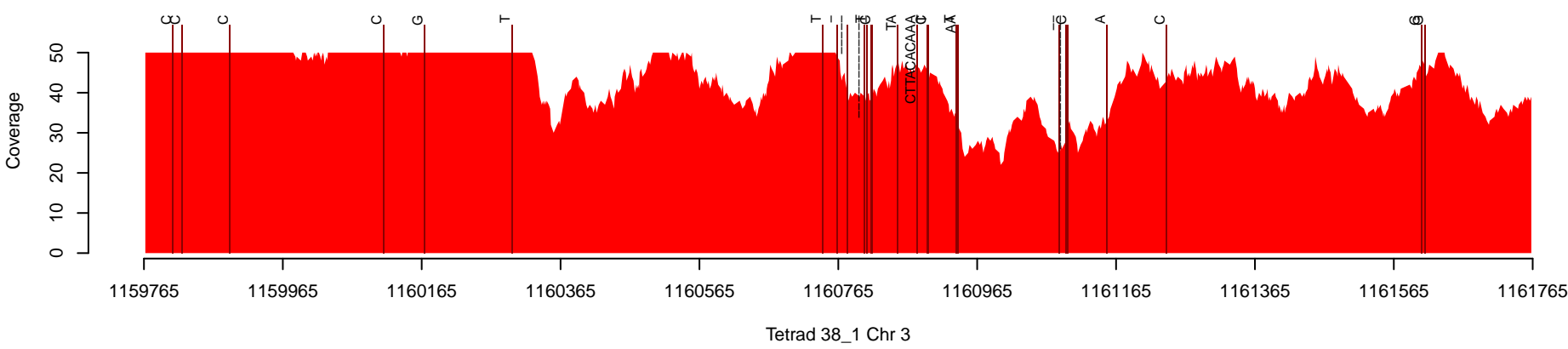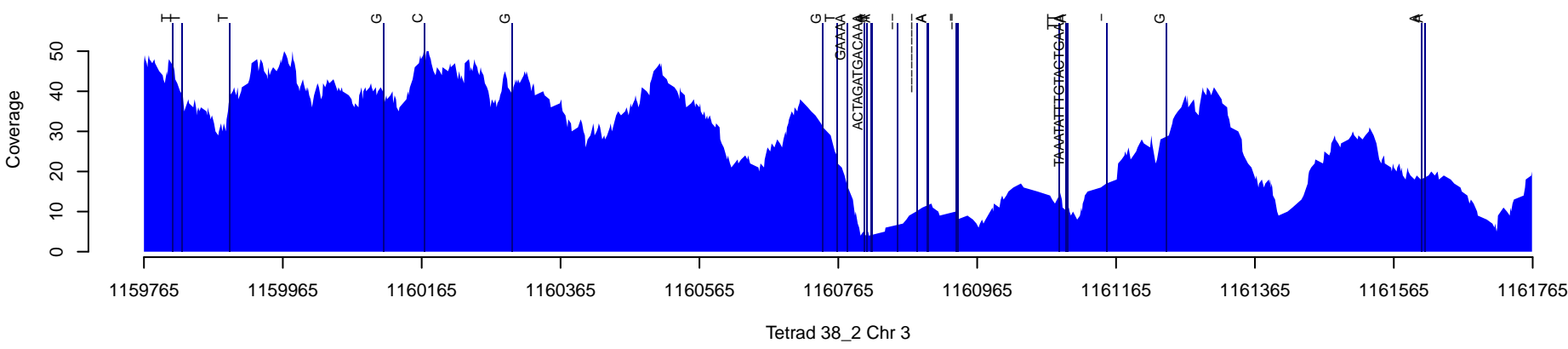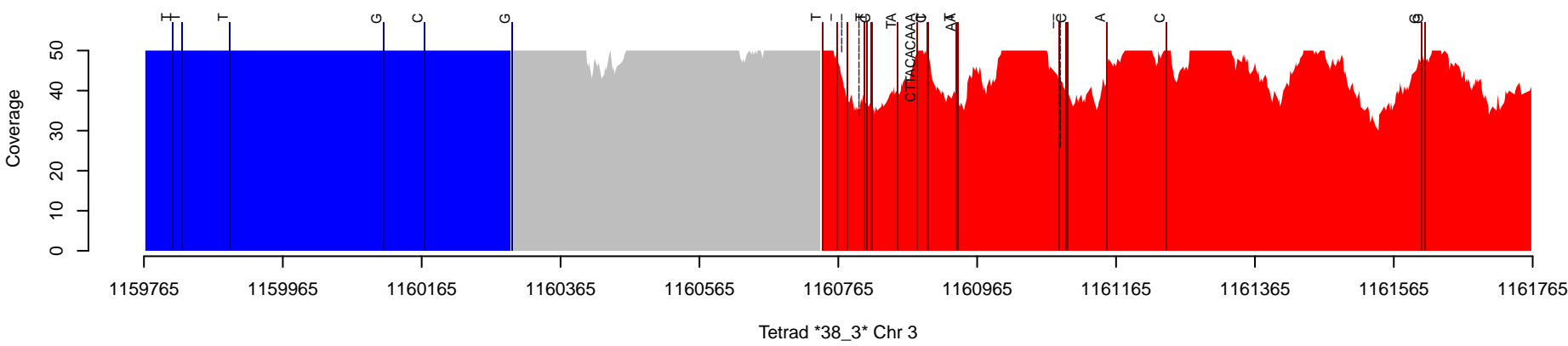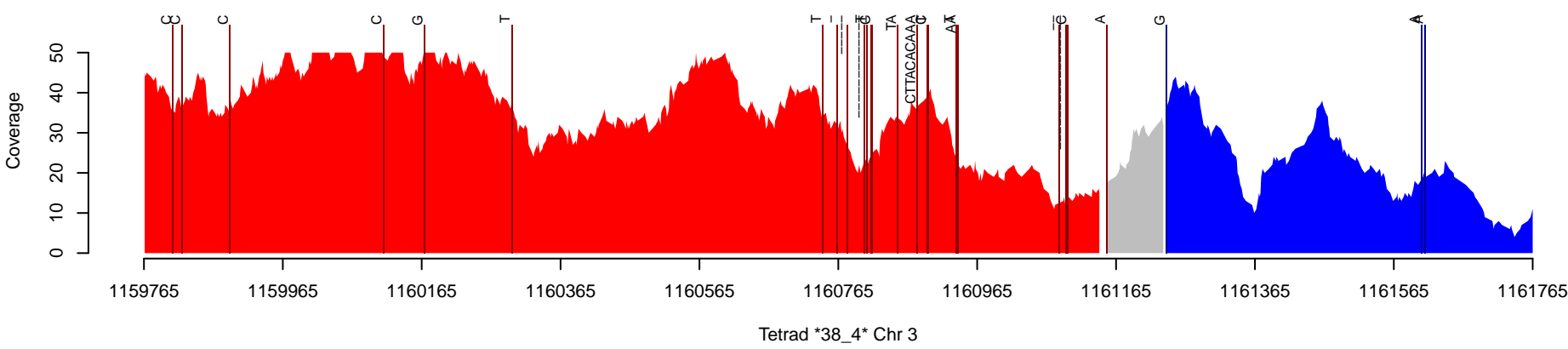

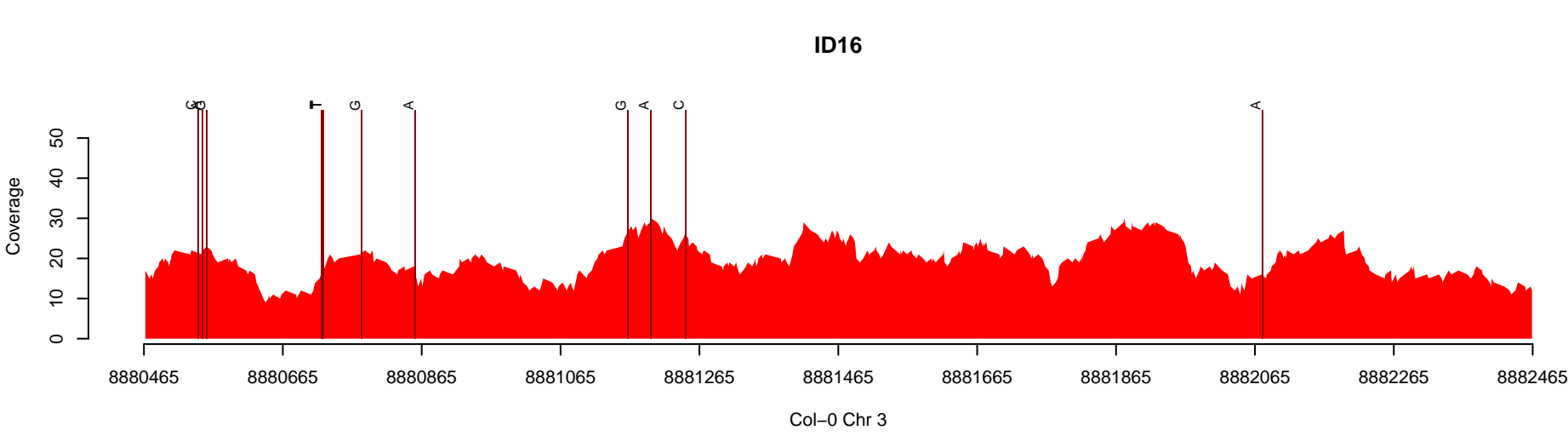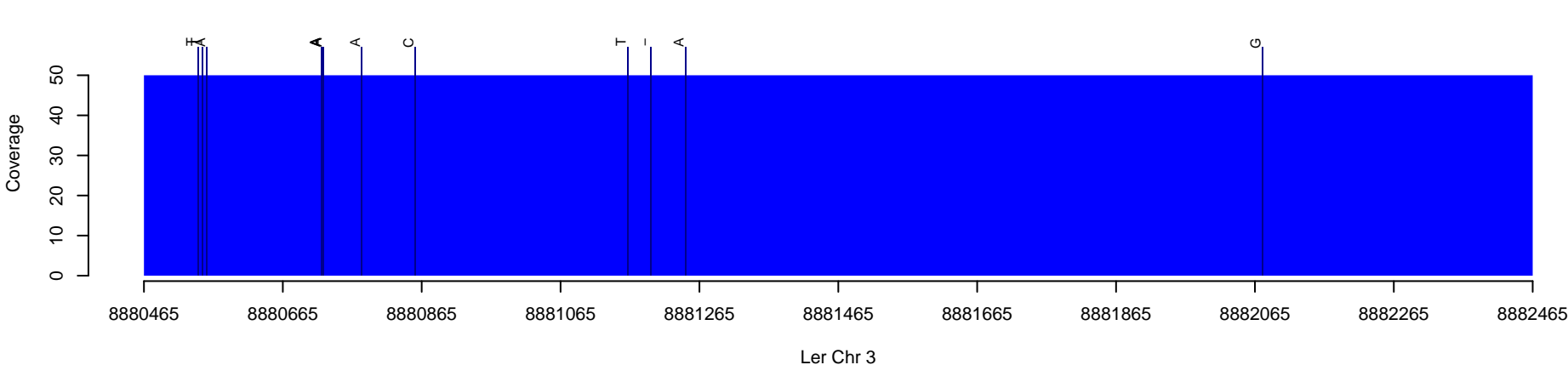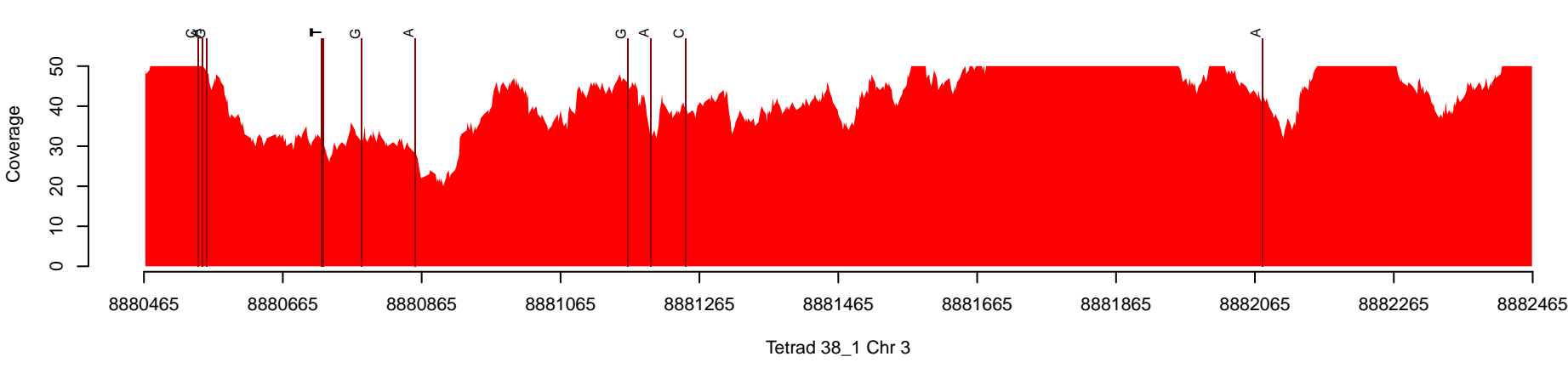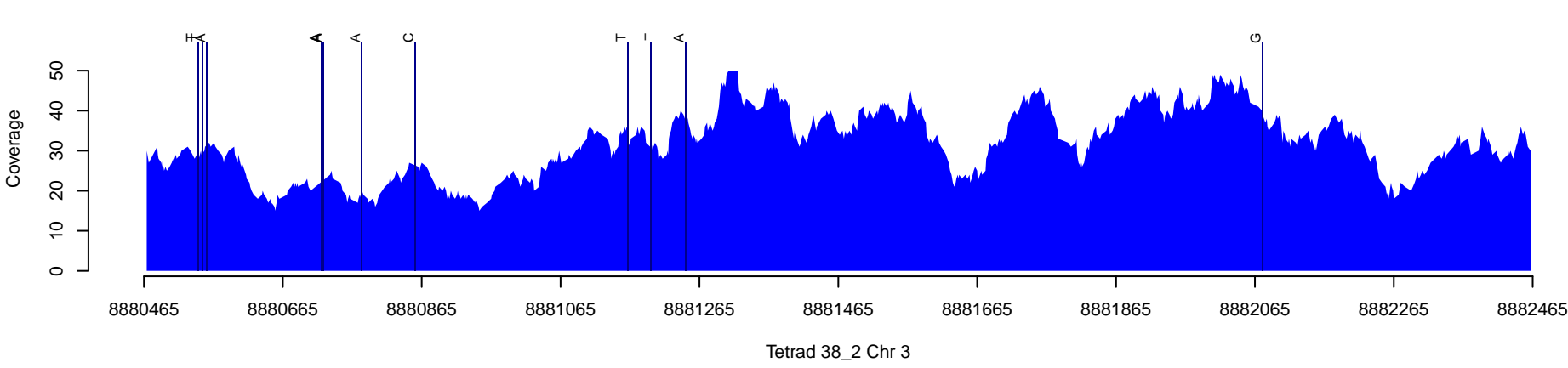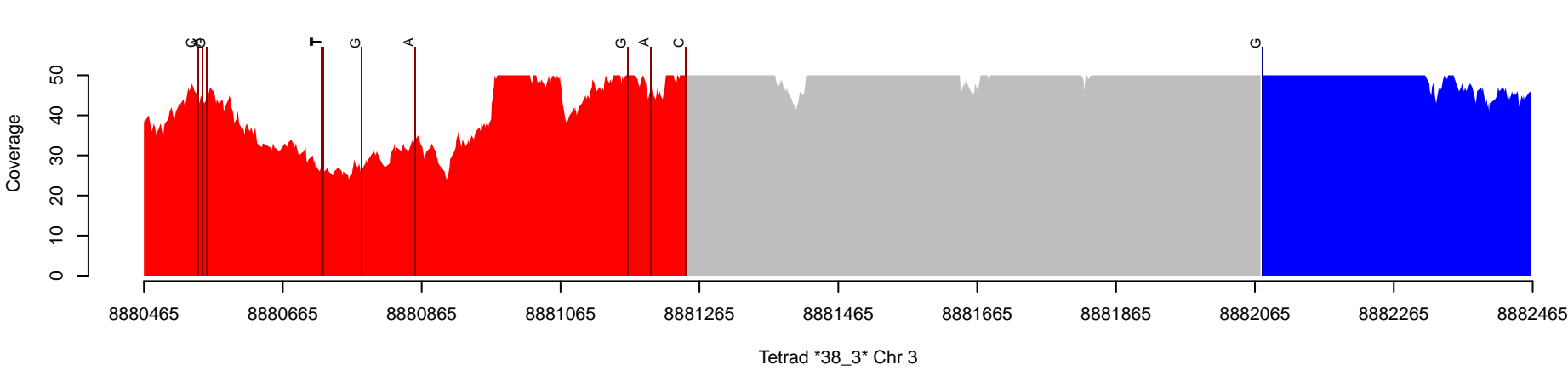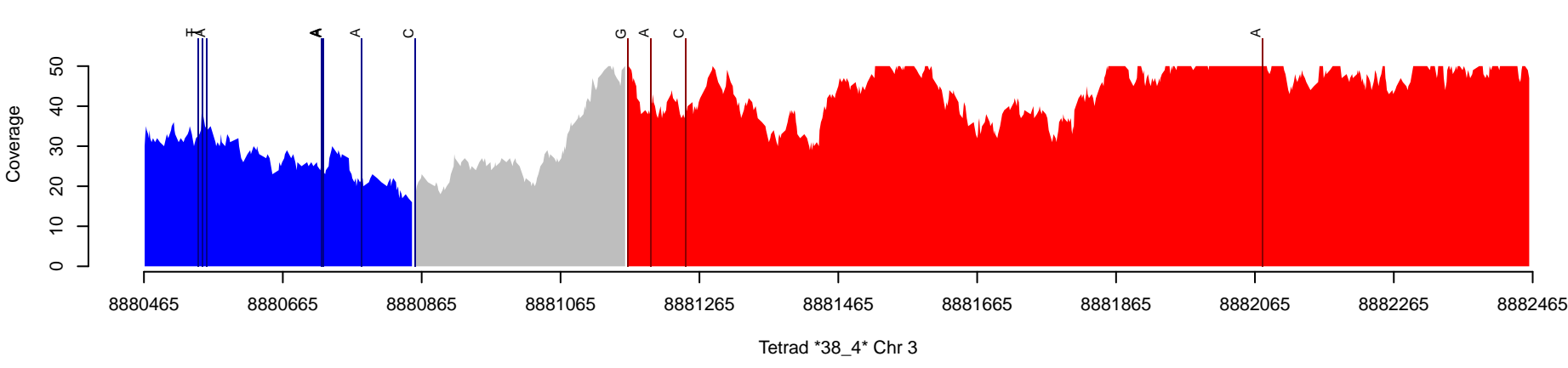

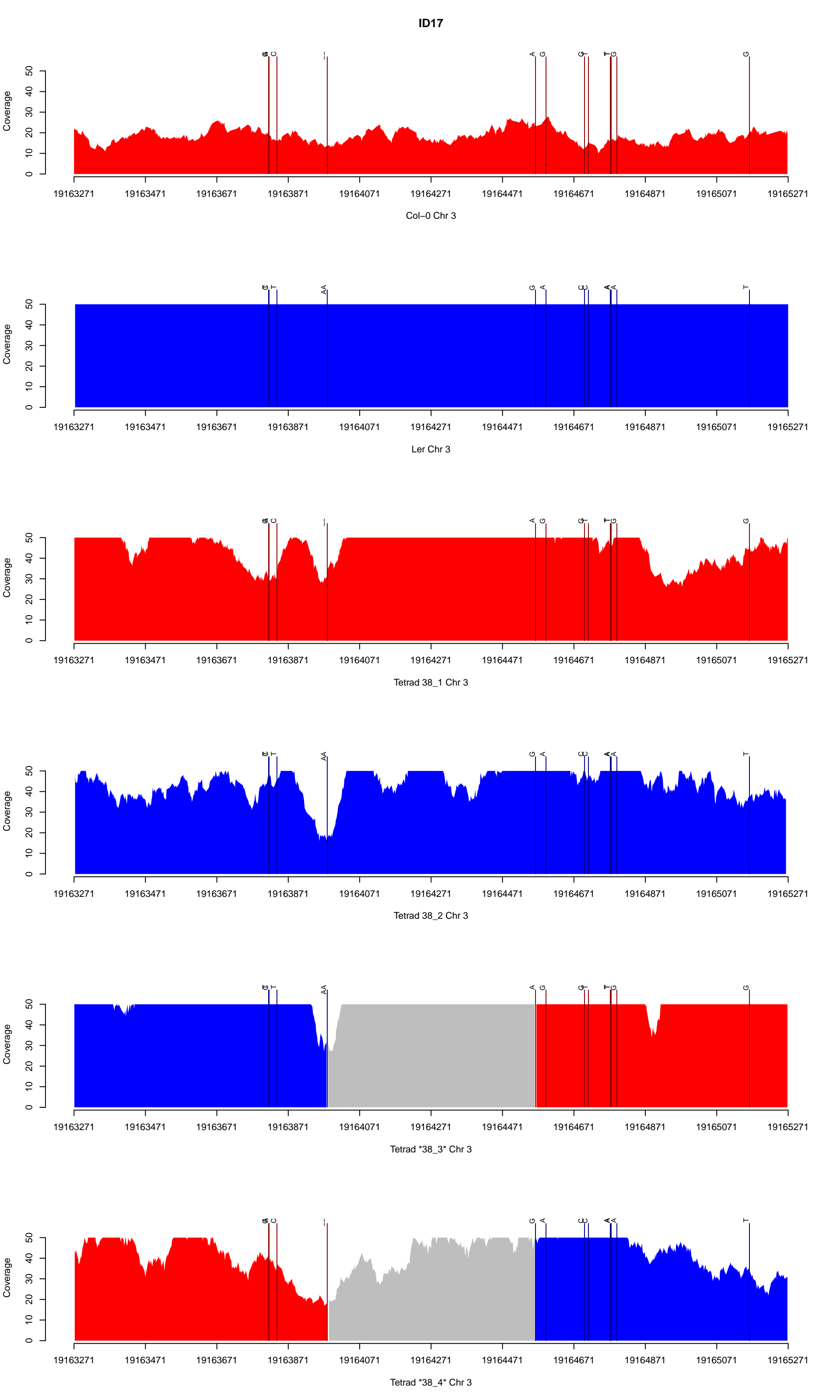

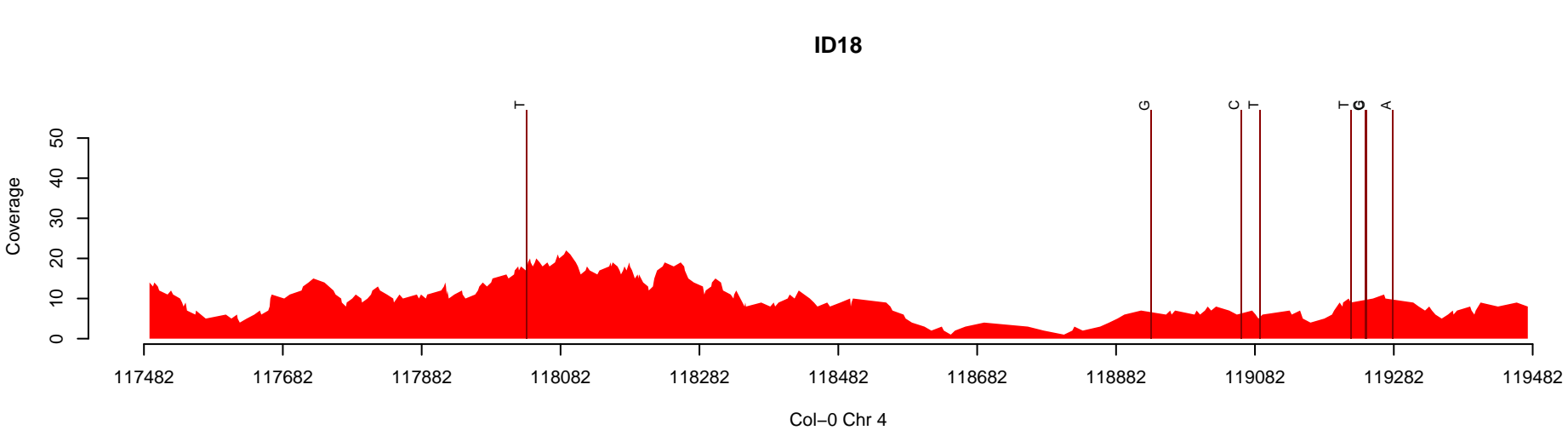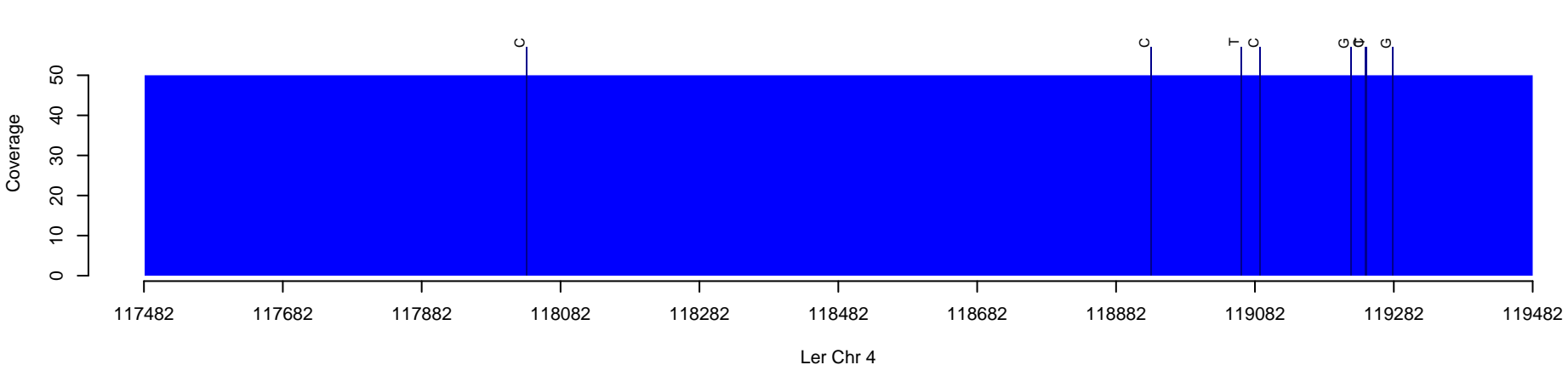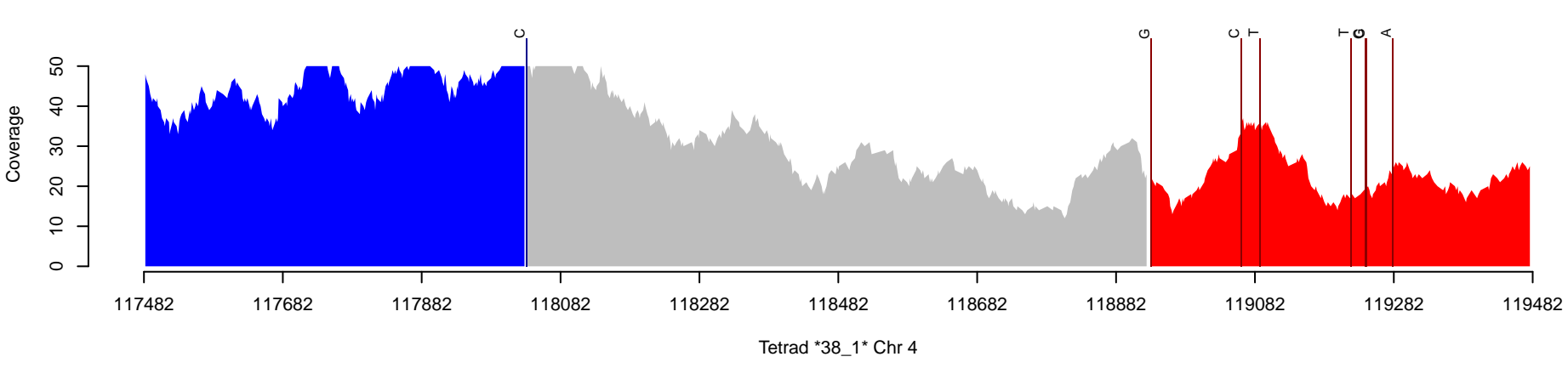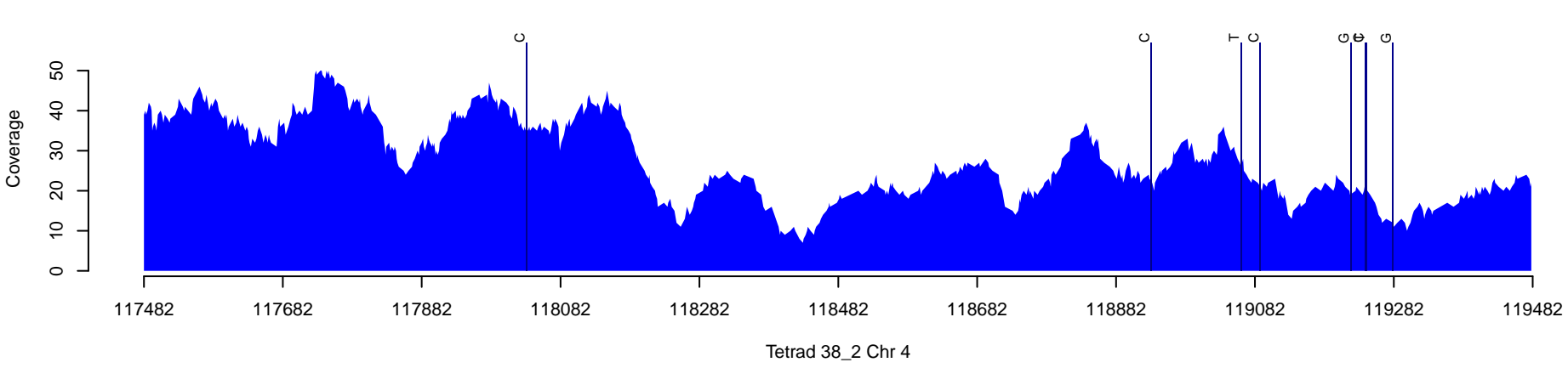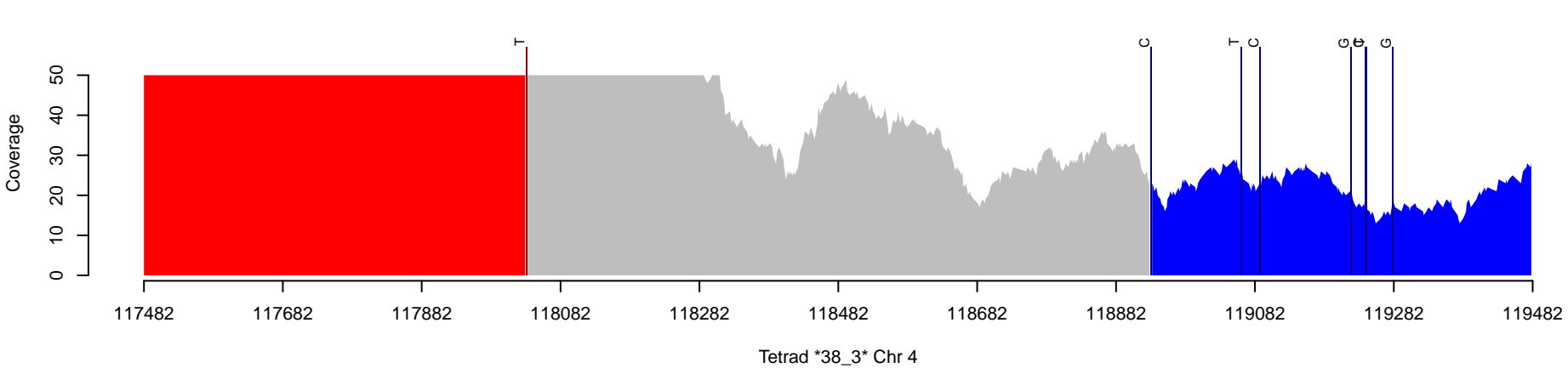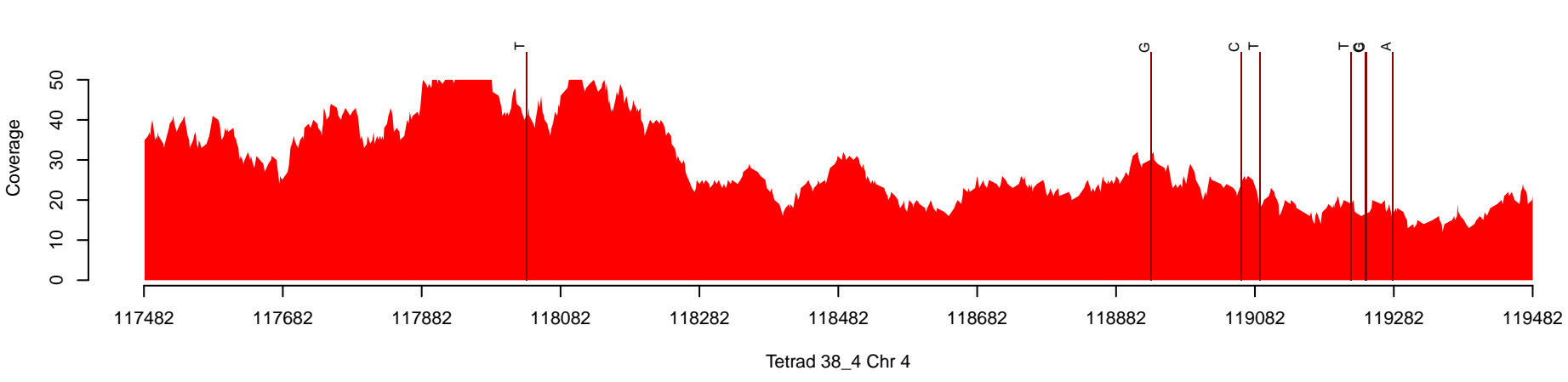

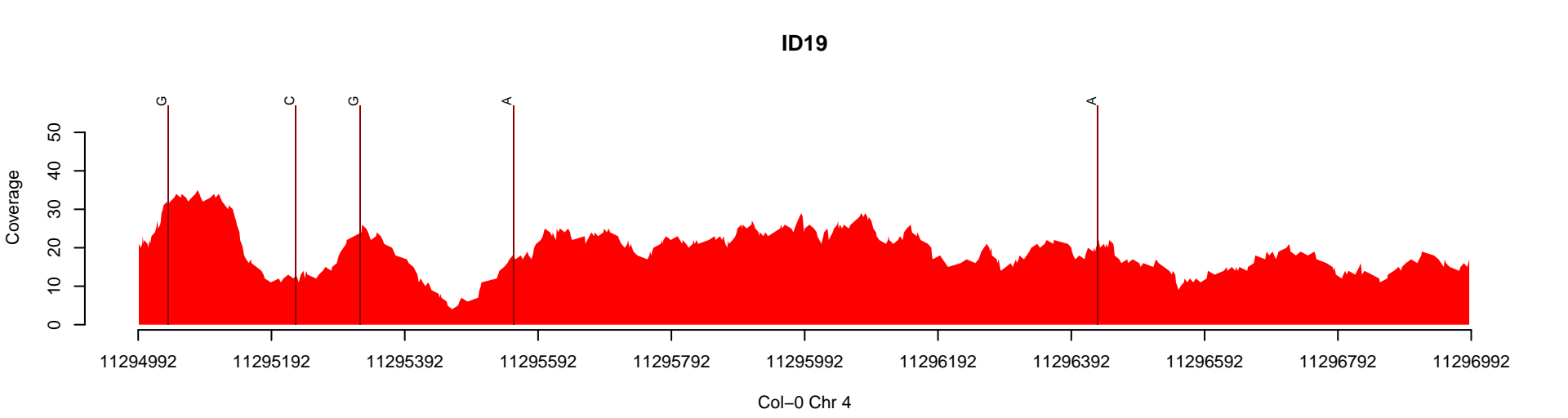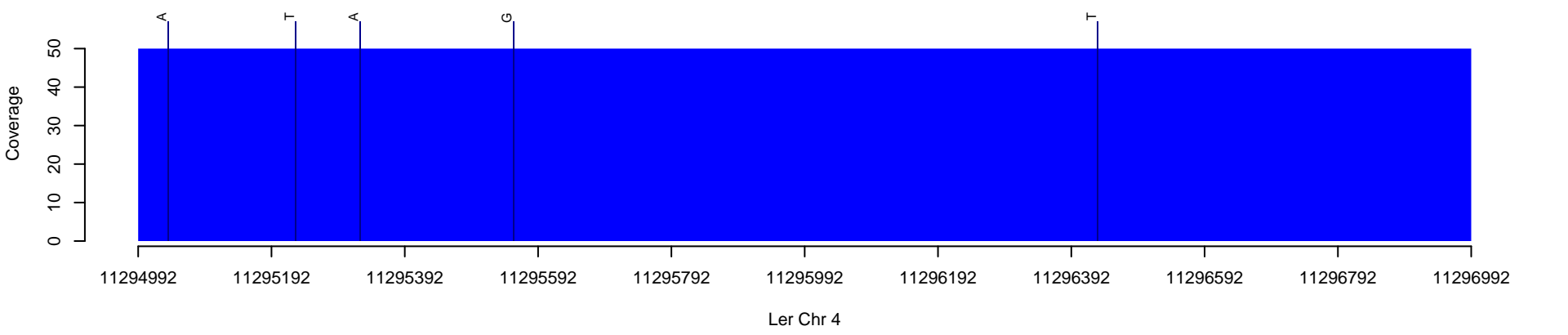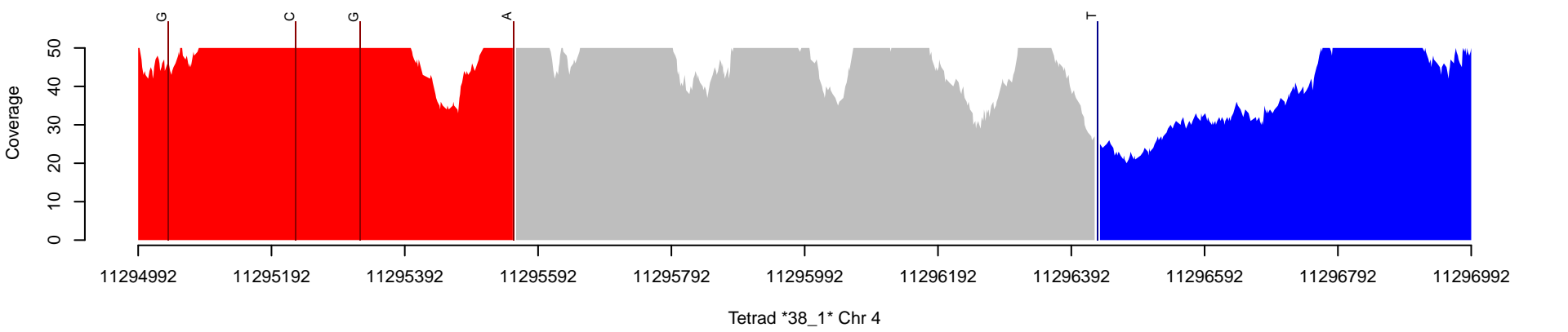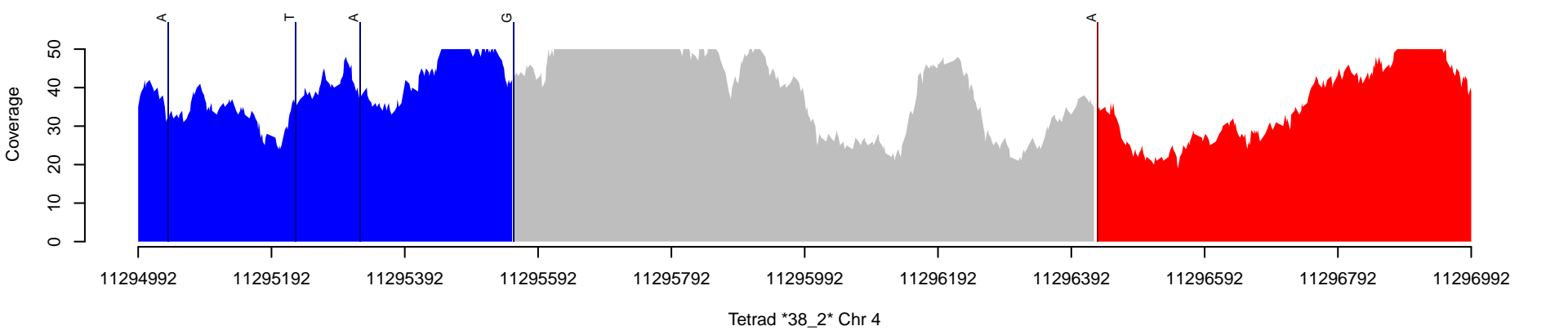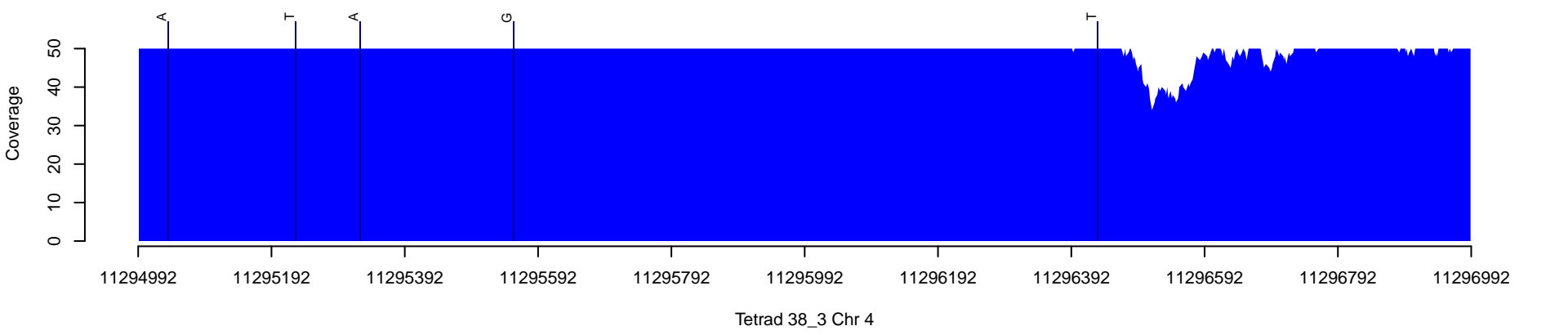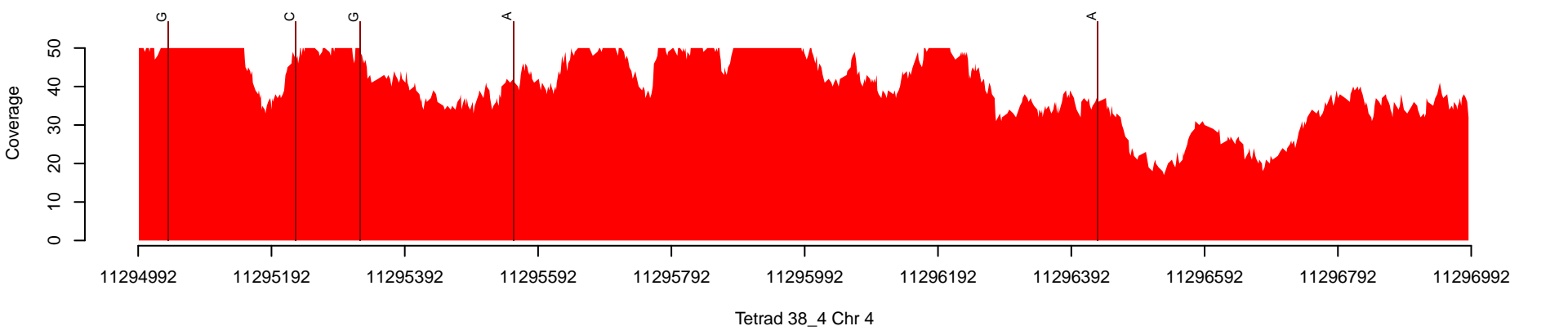

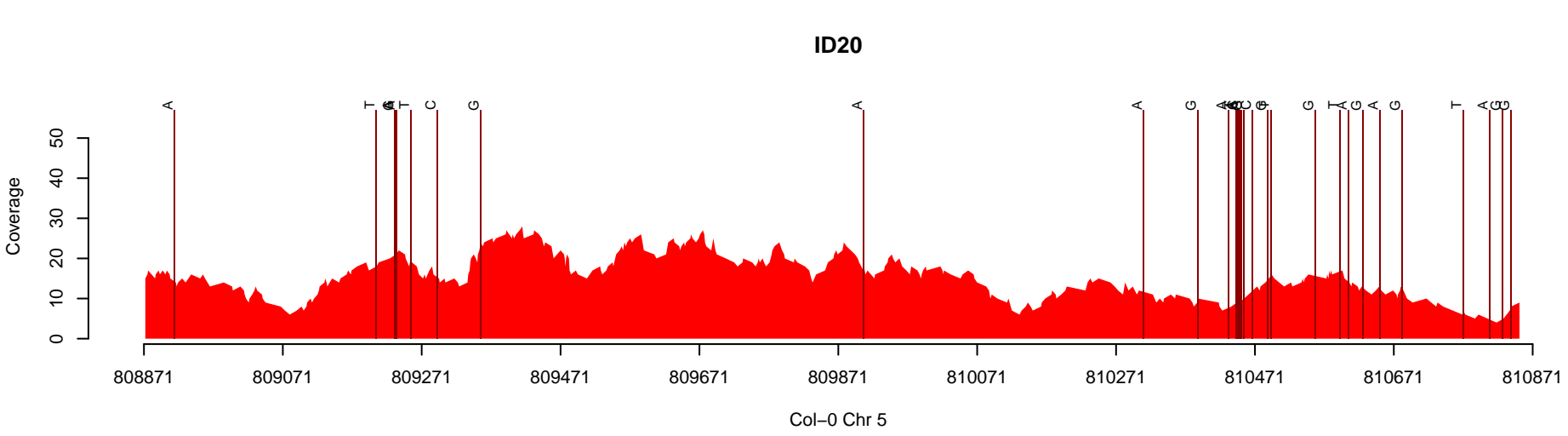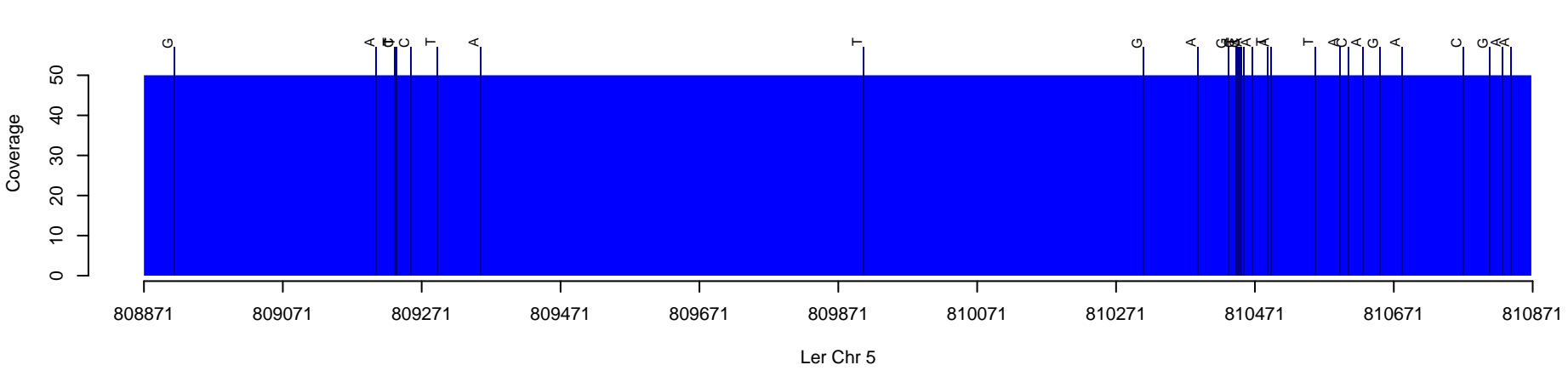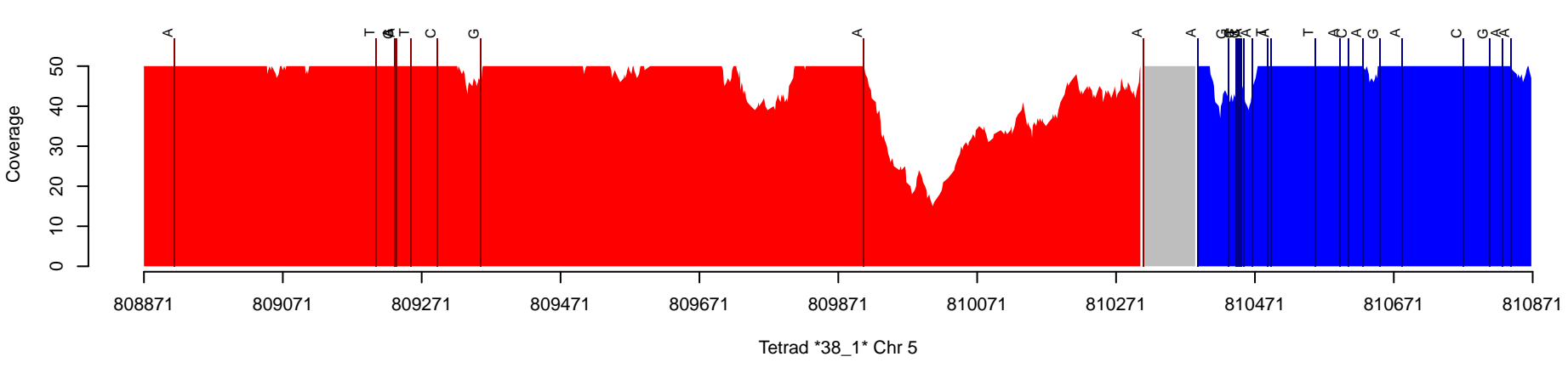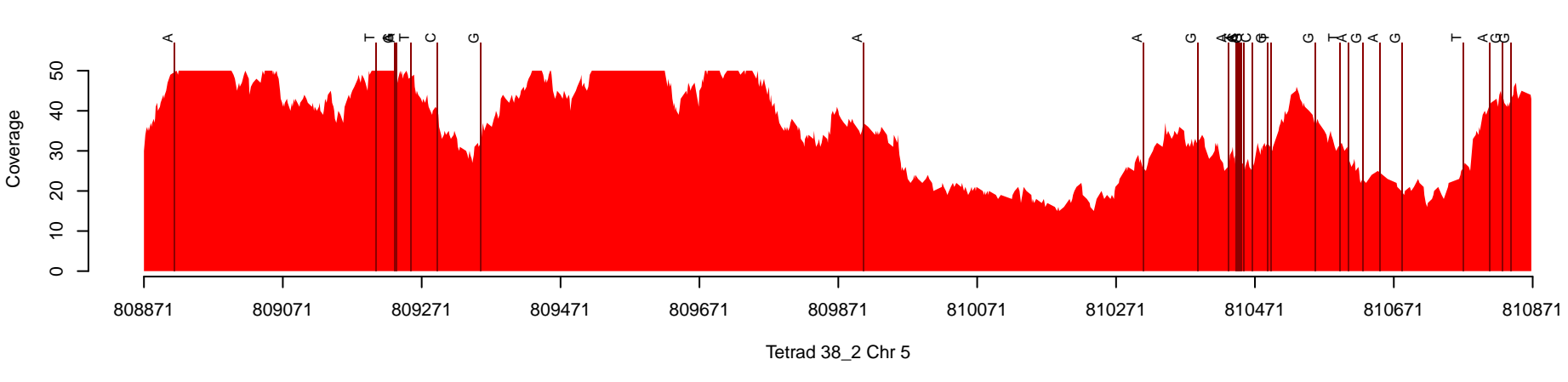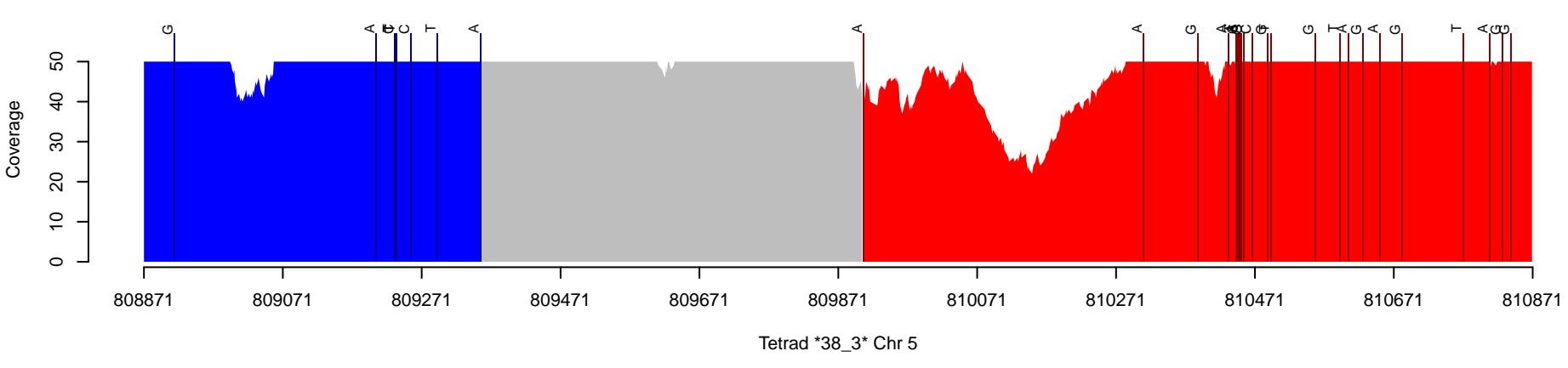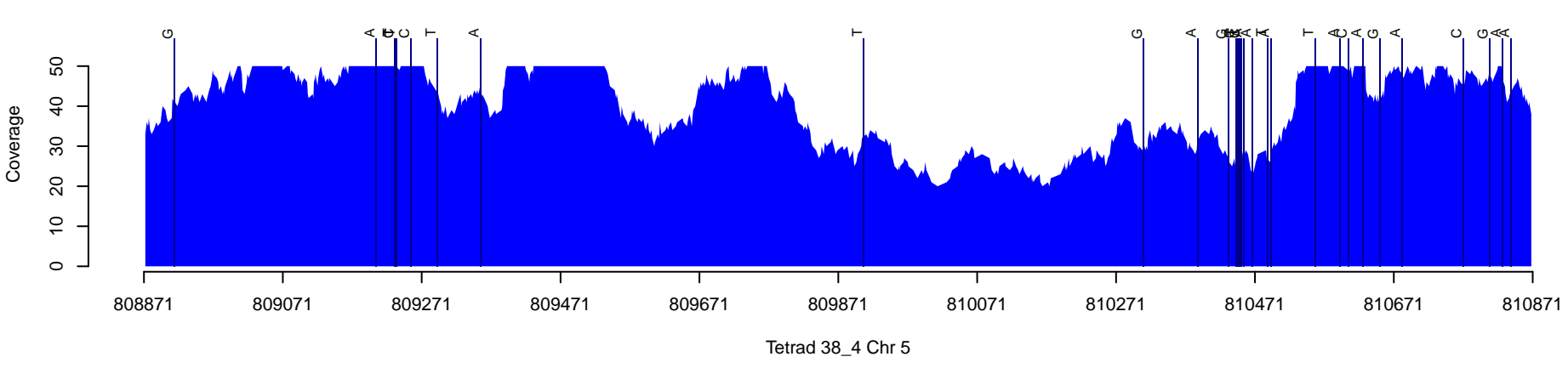

ID21

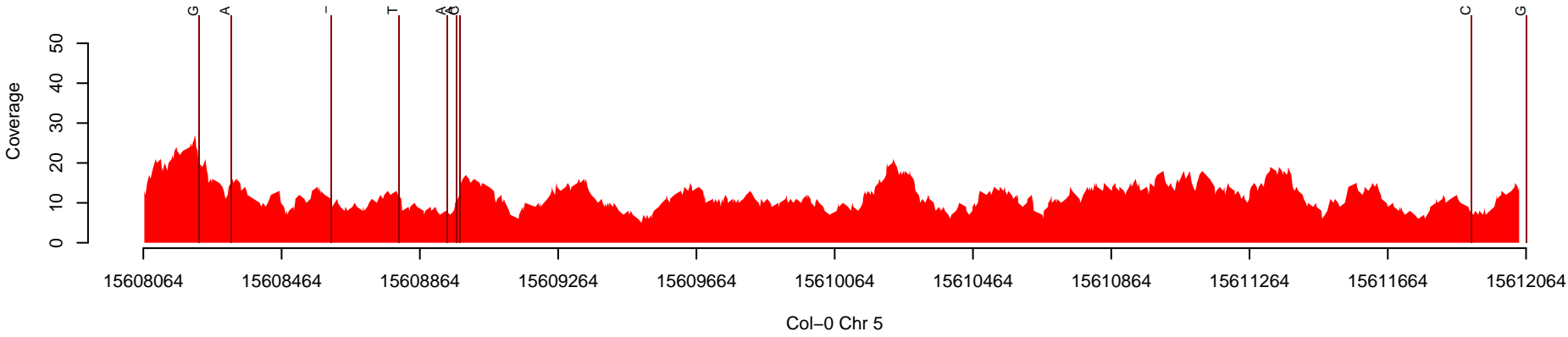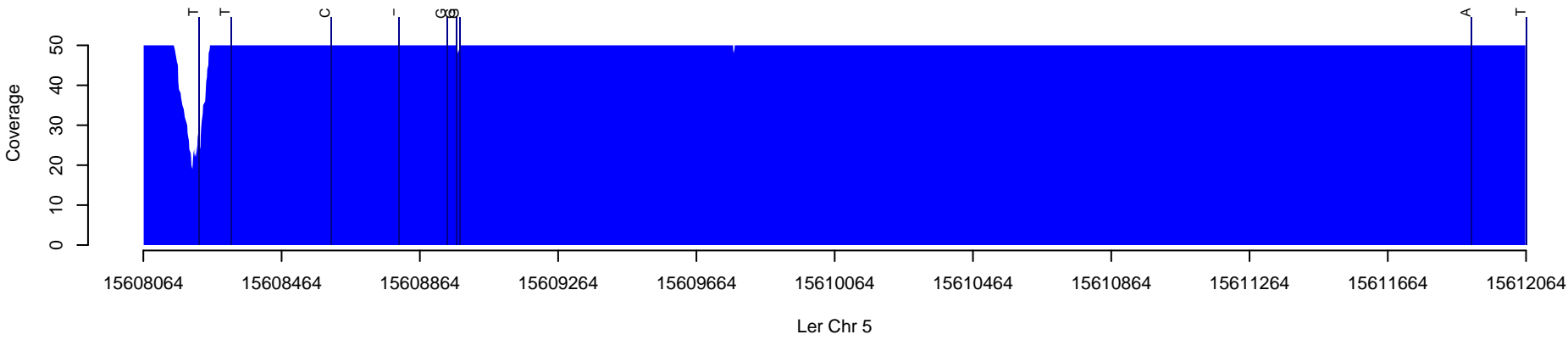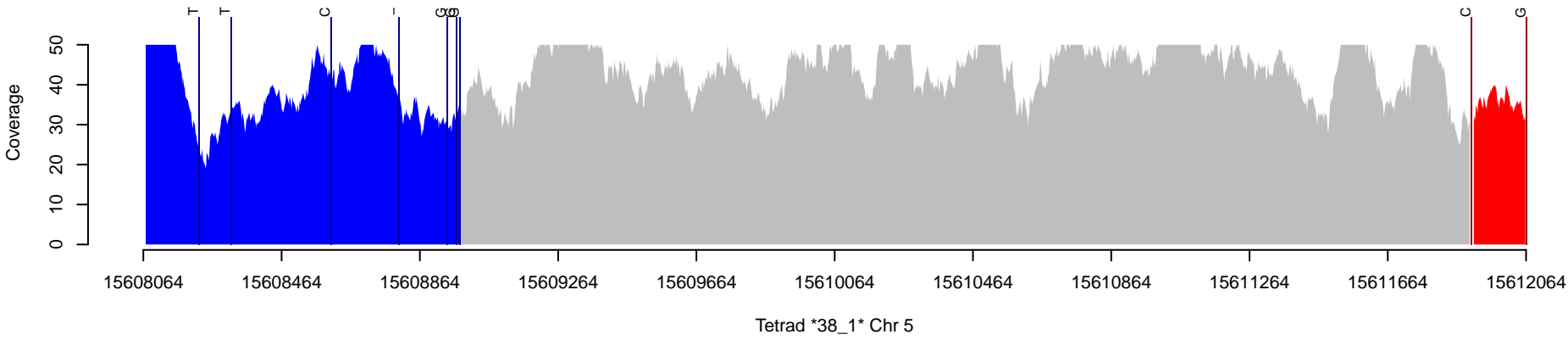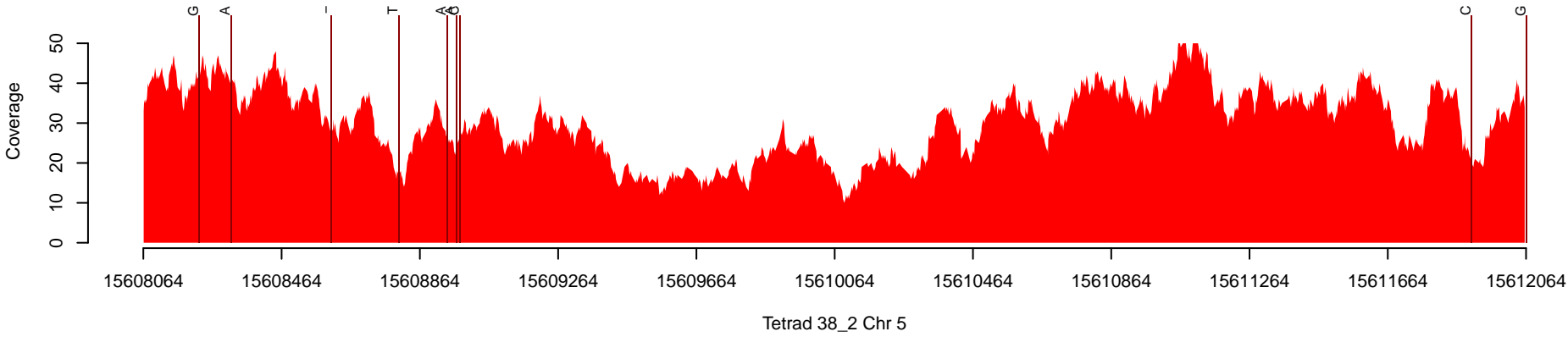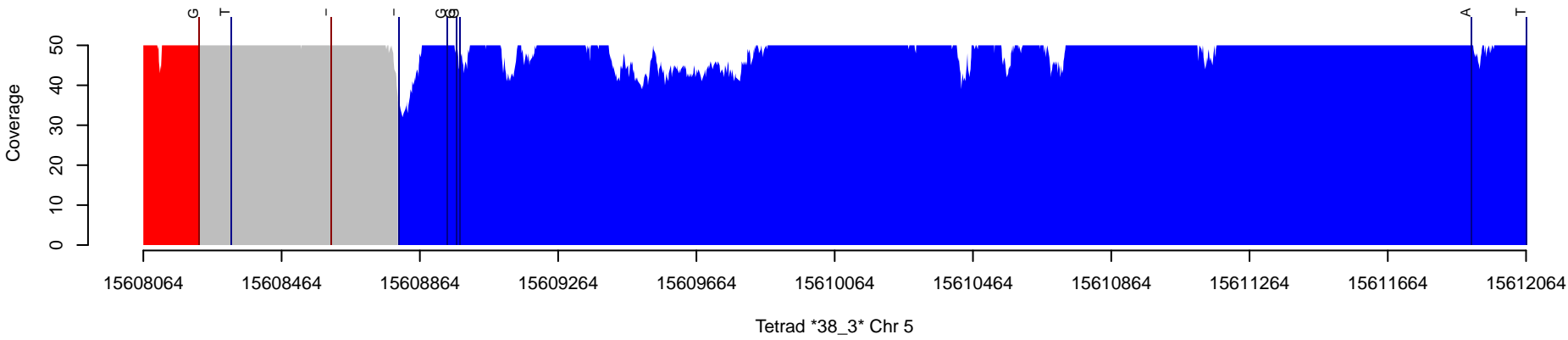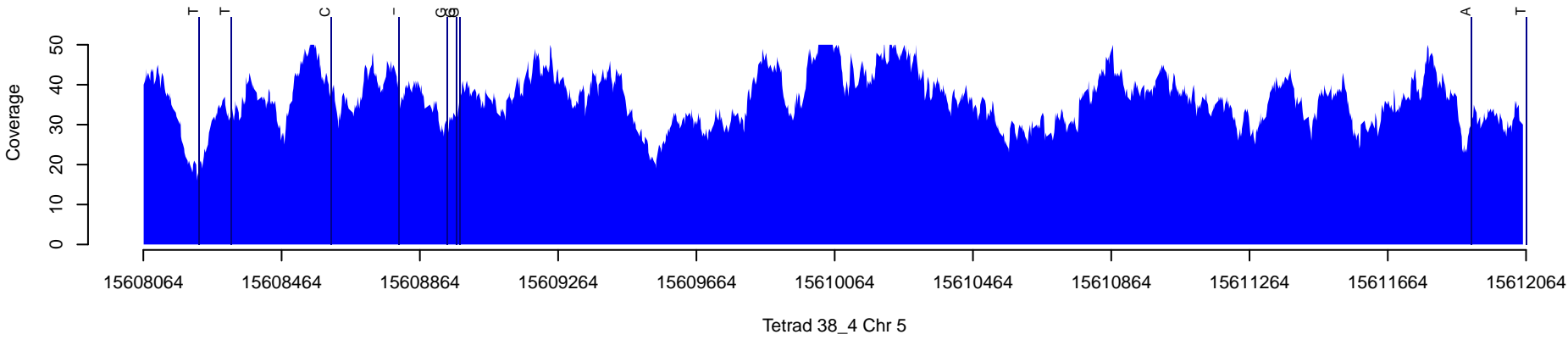

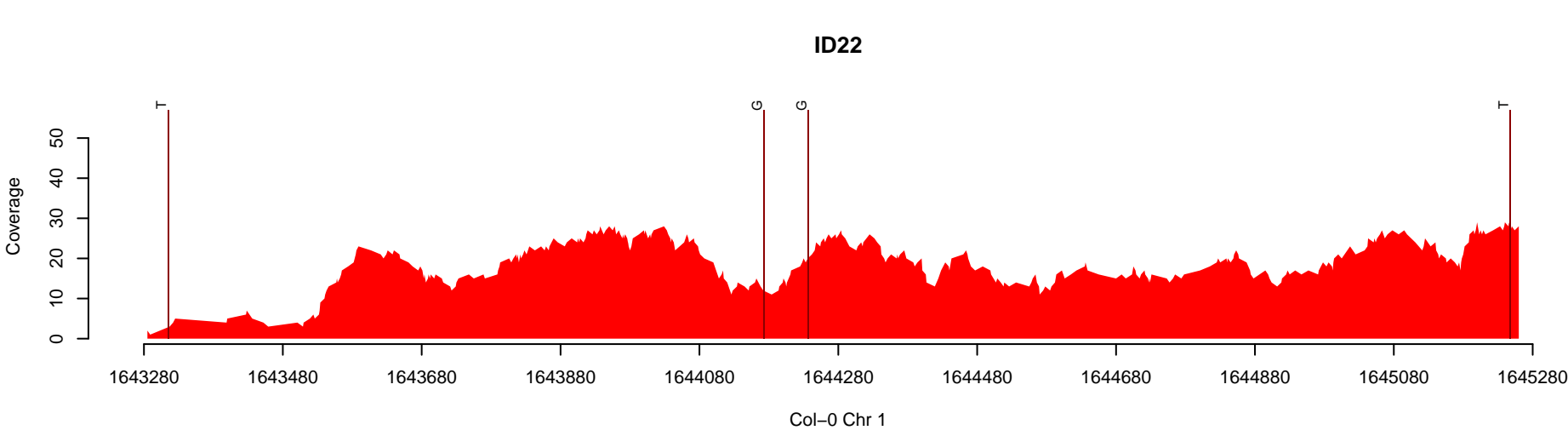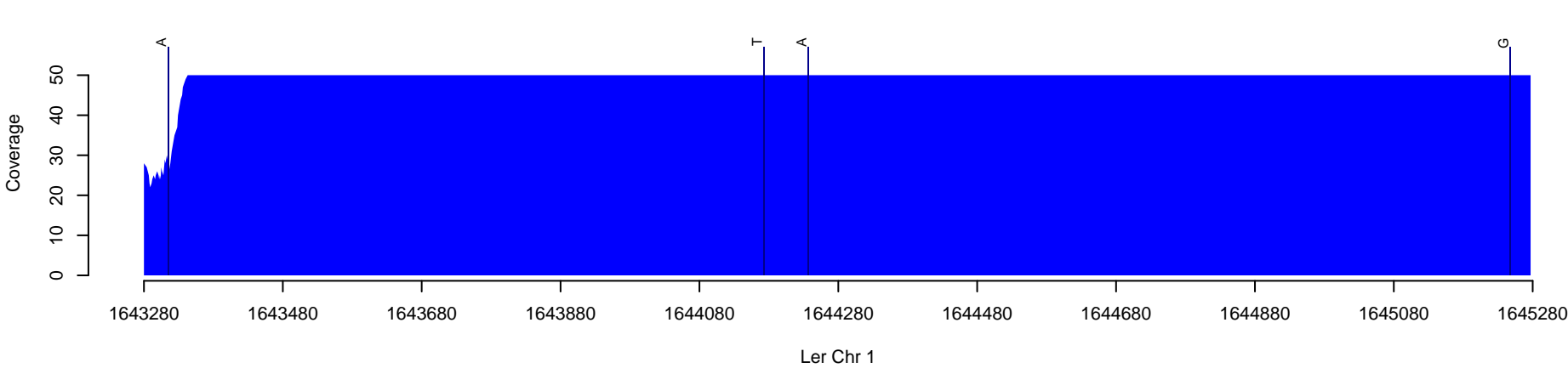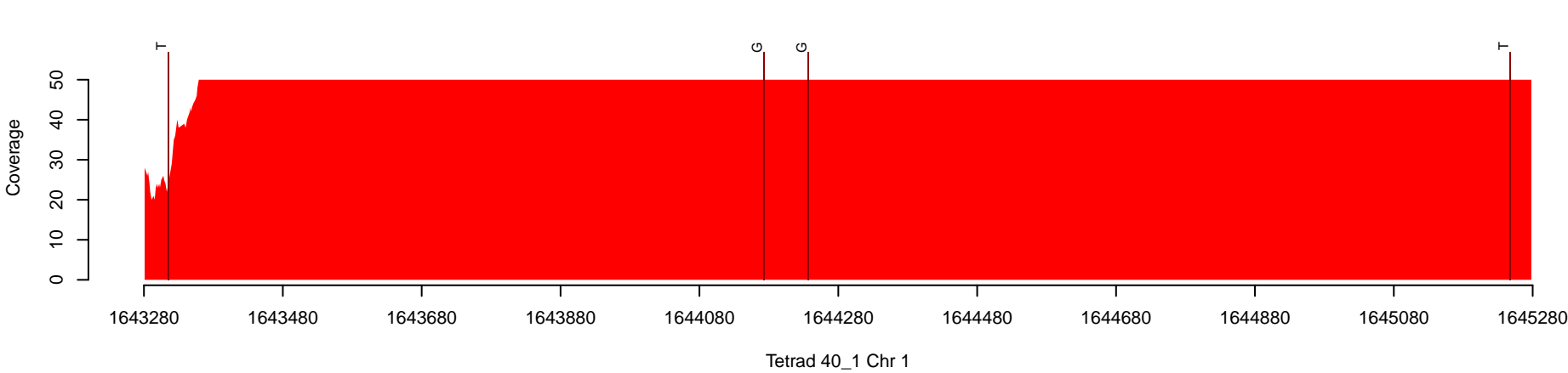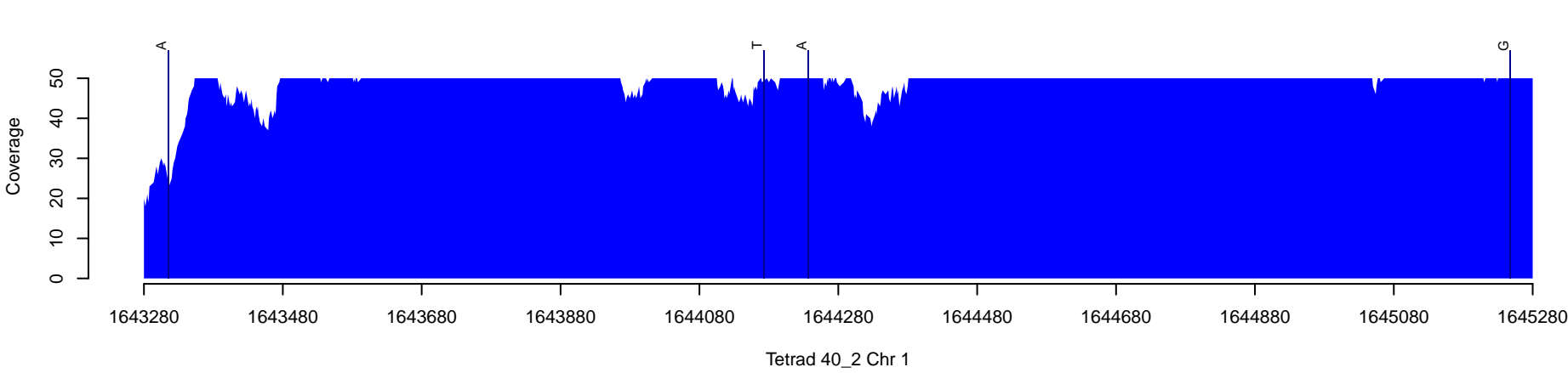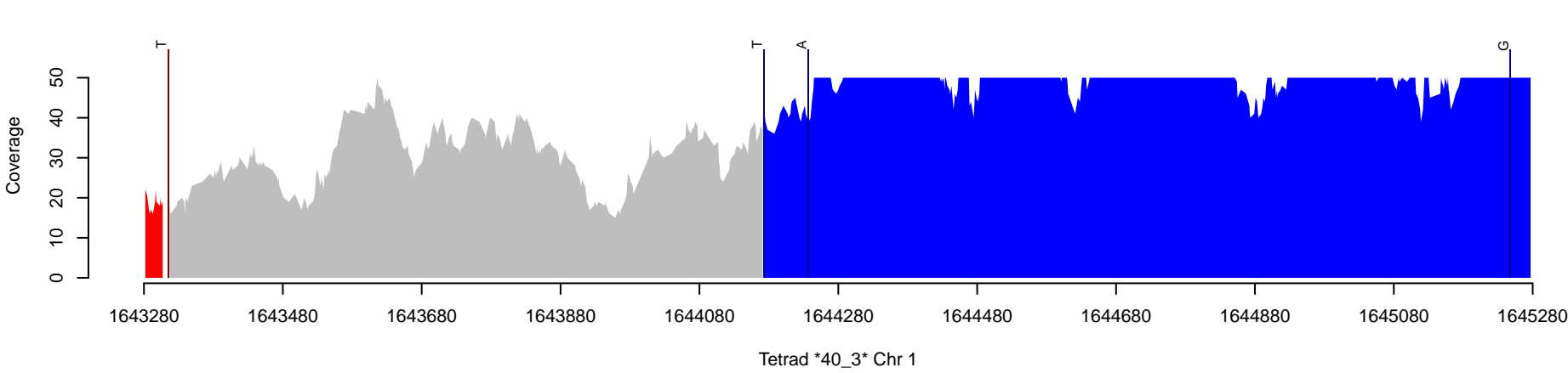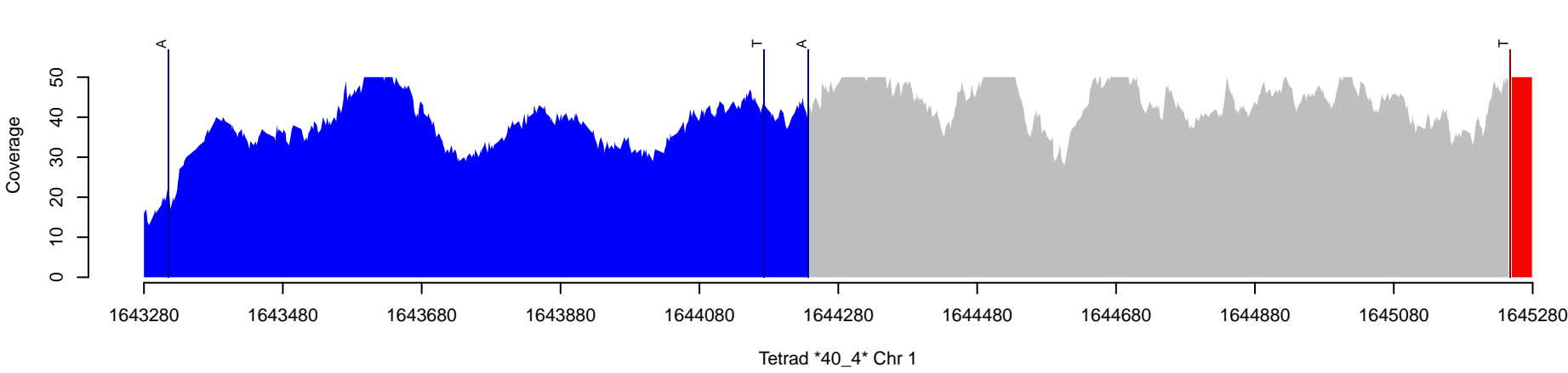

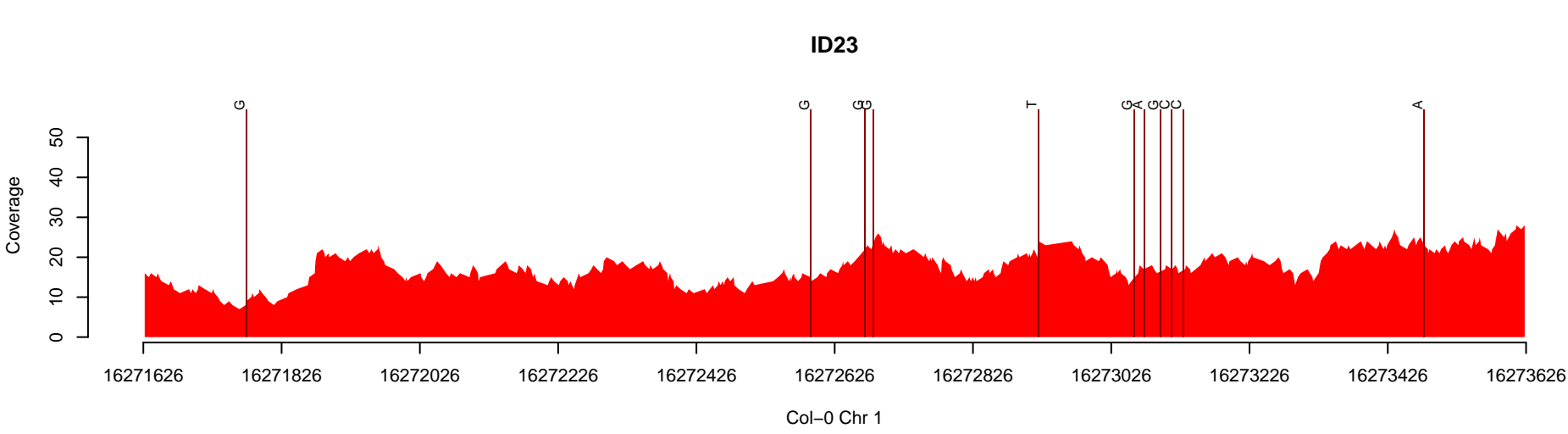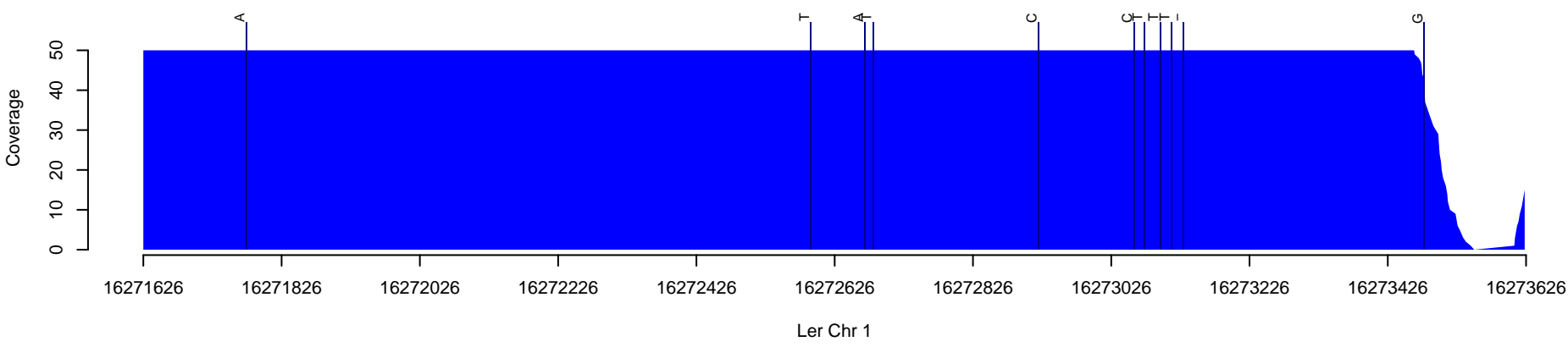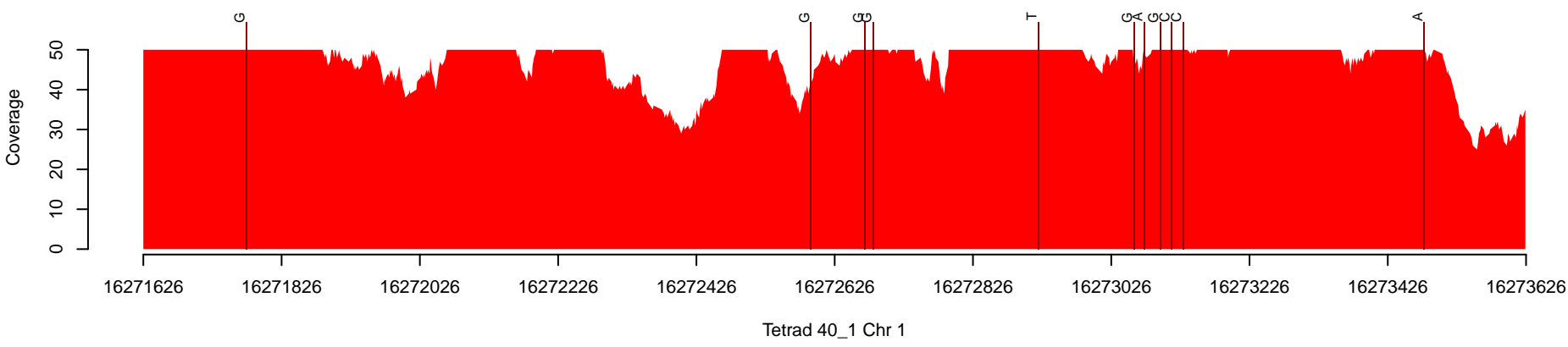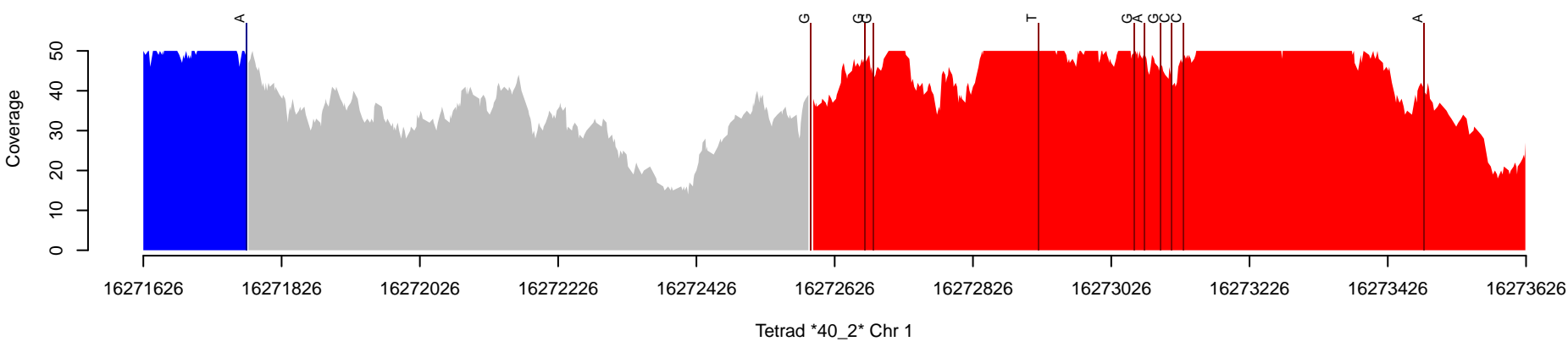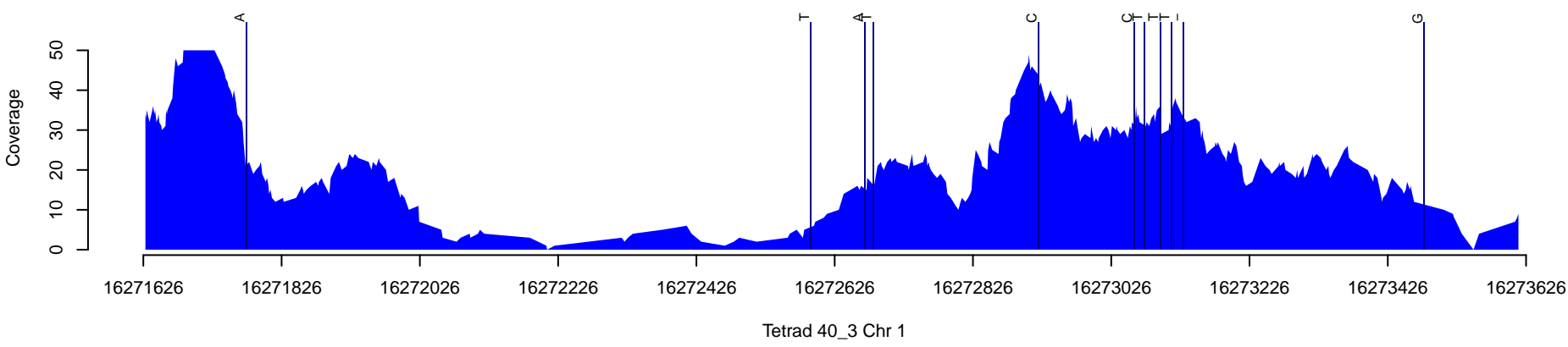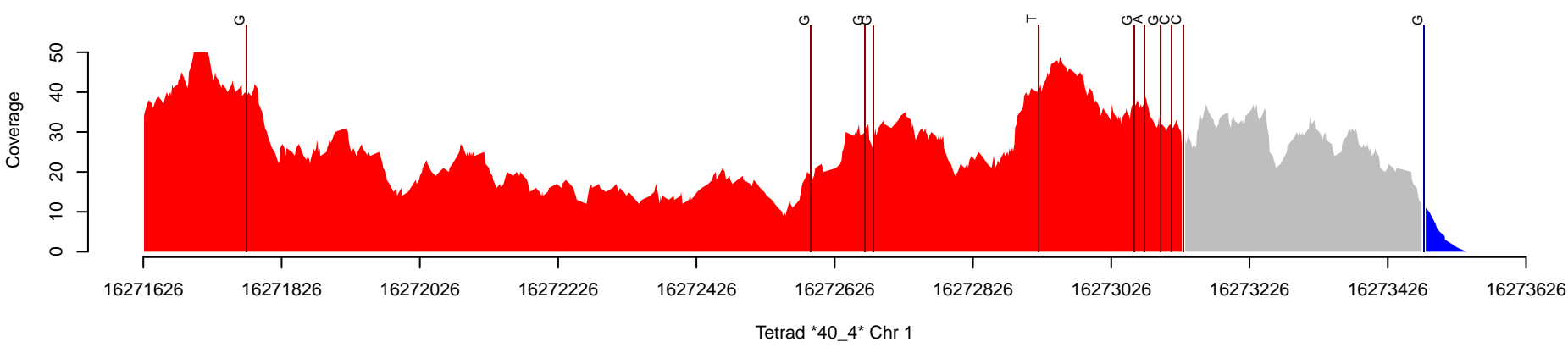

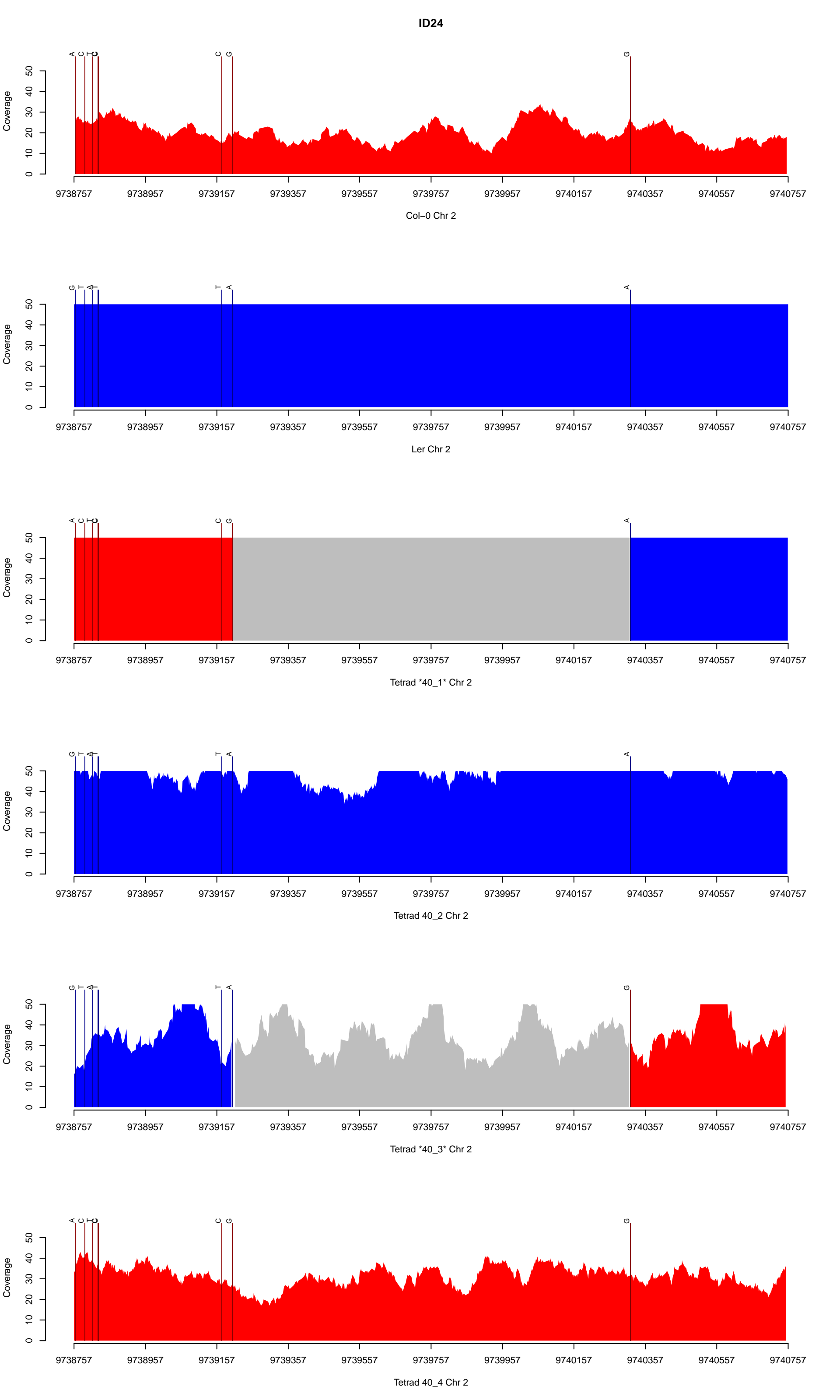

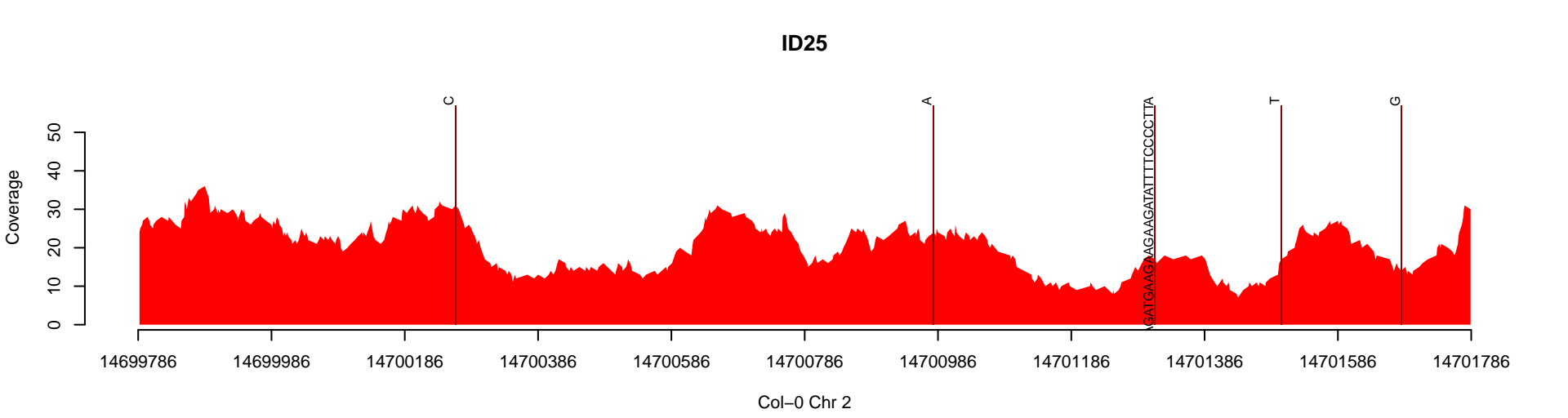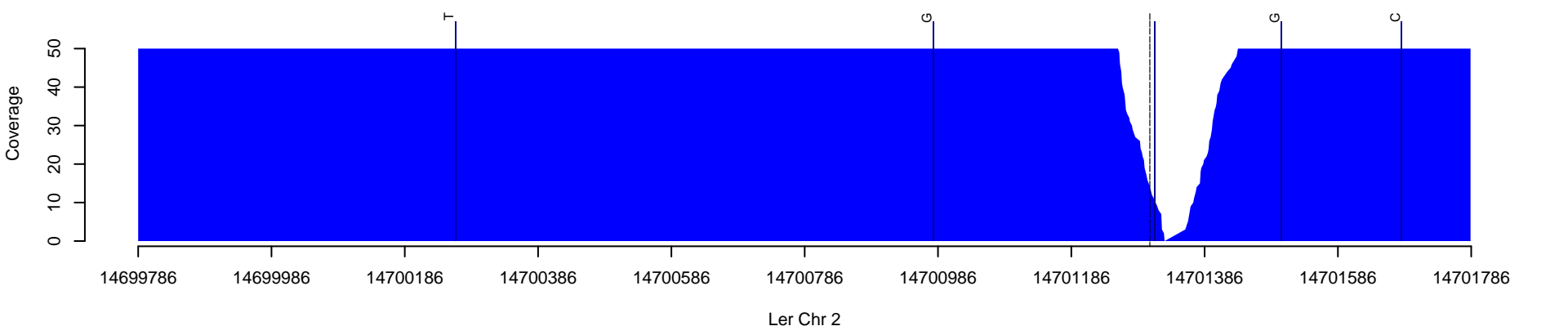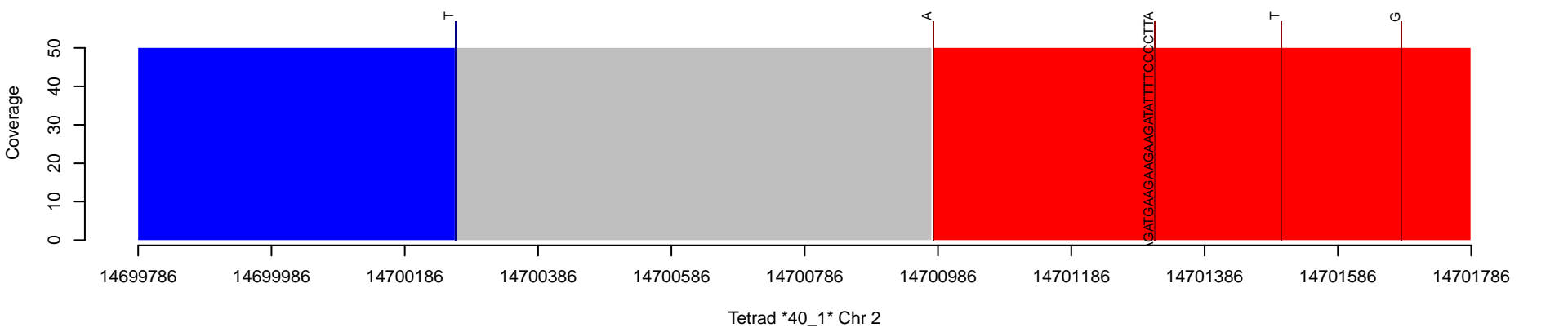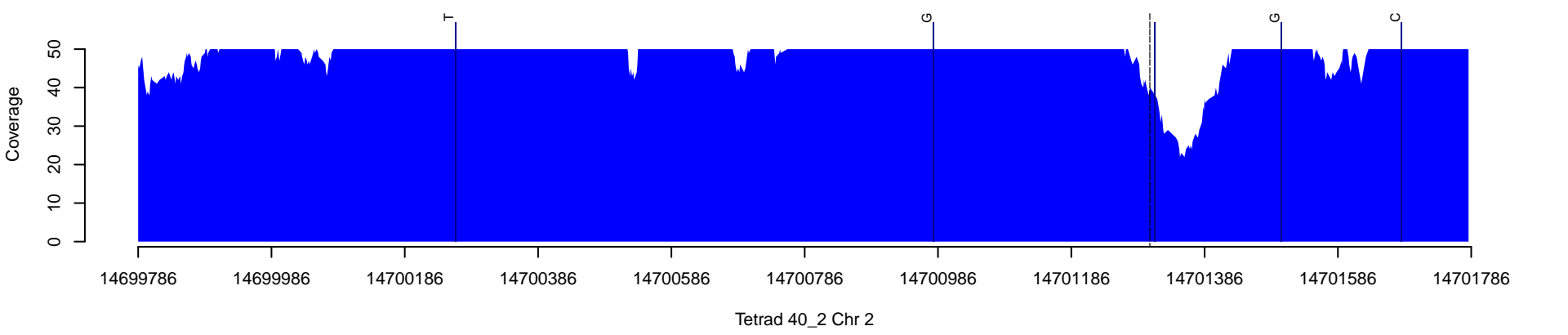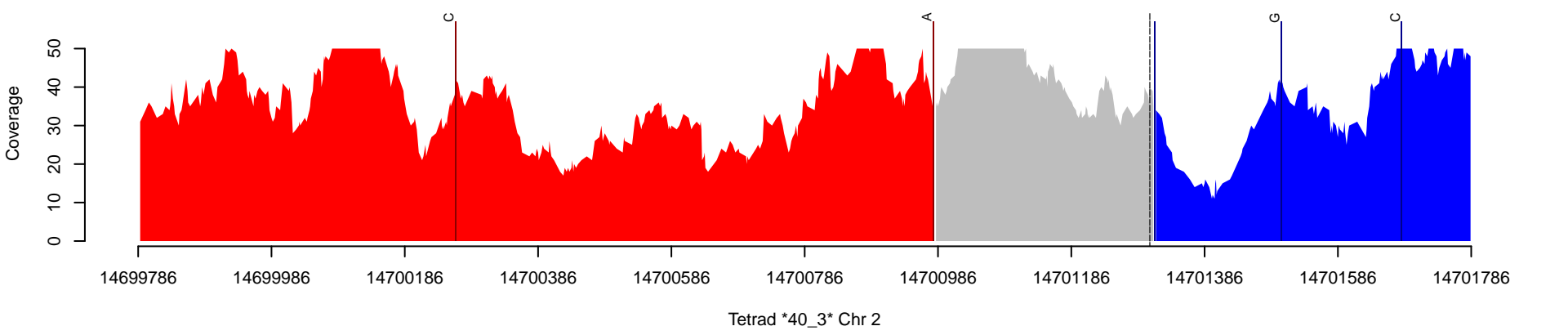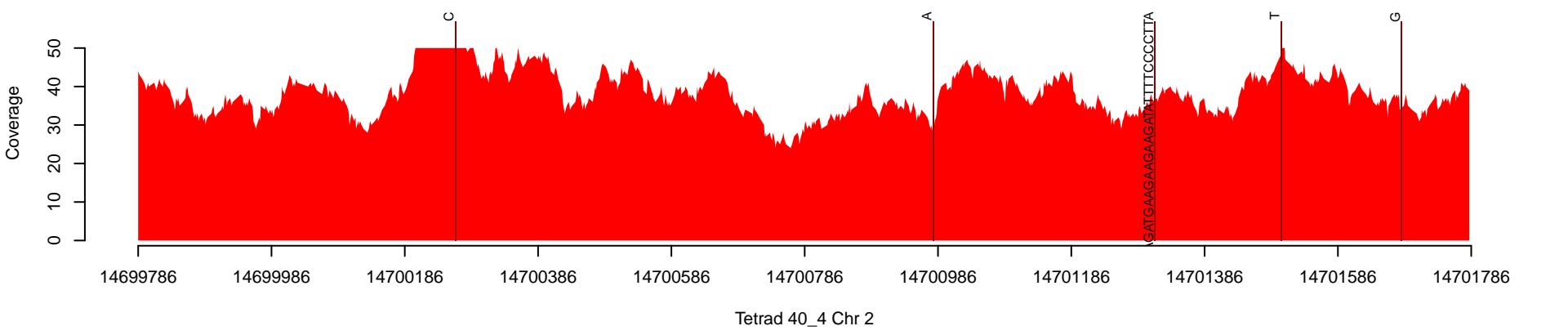

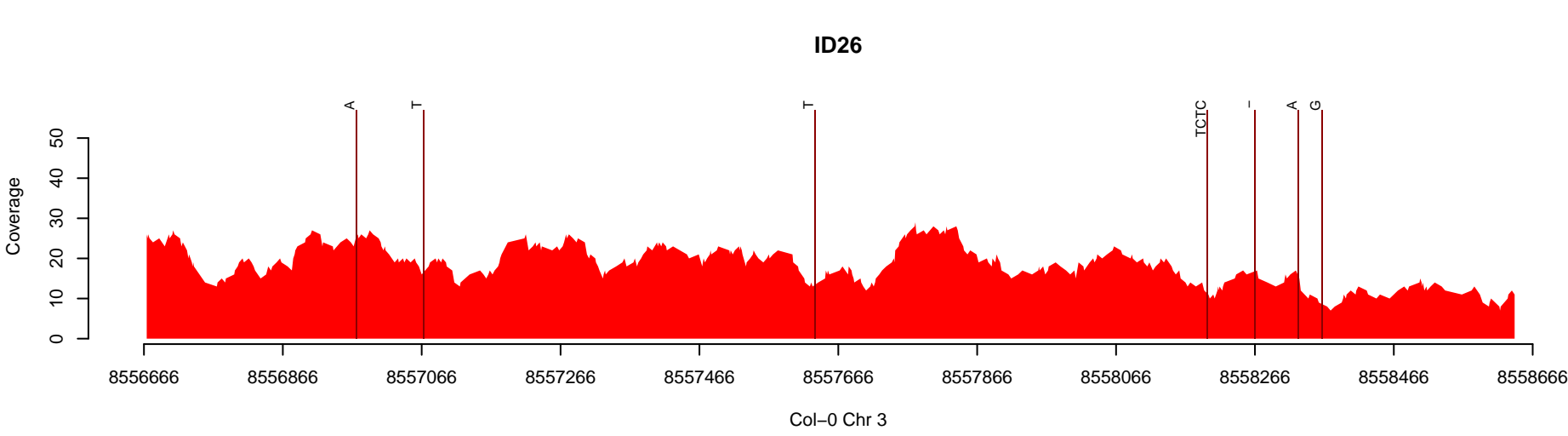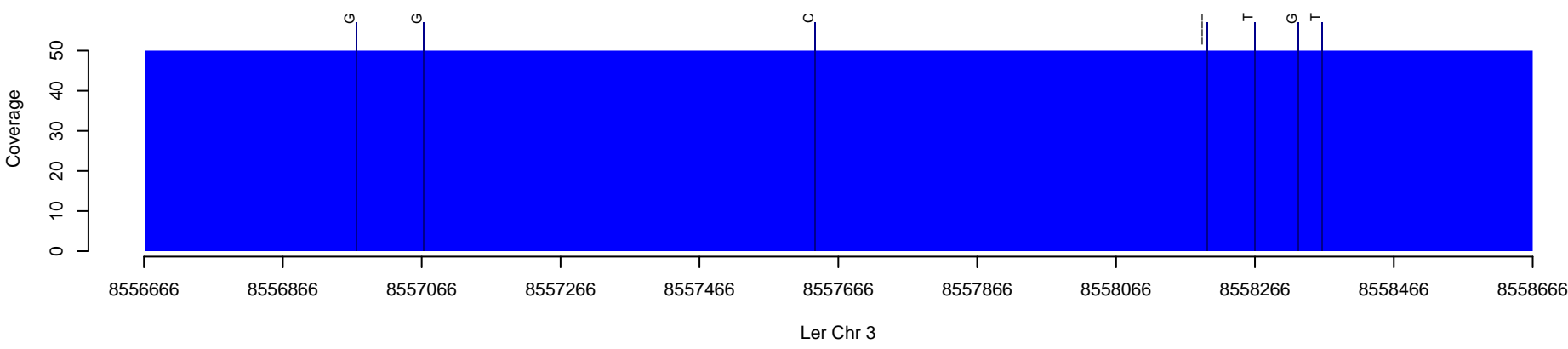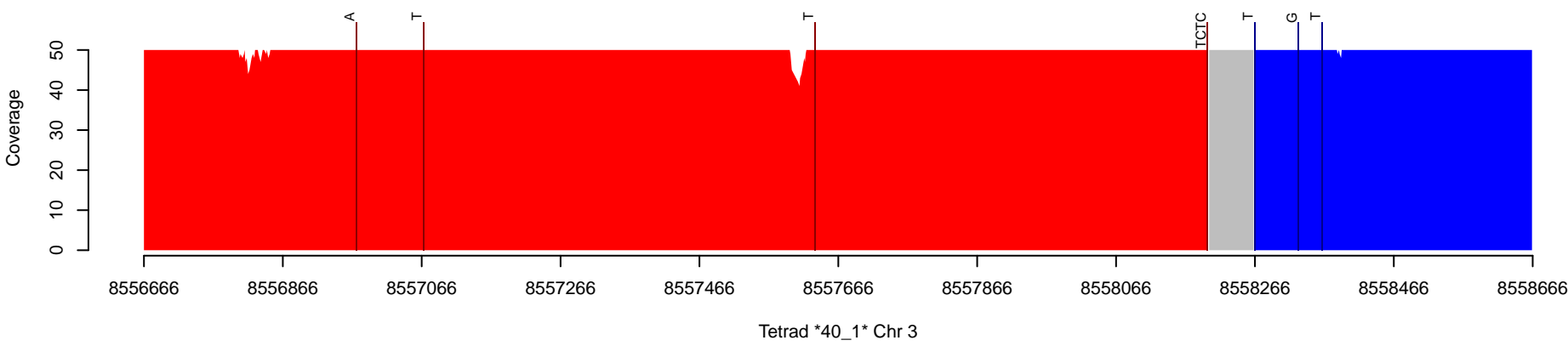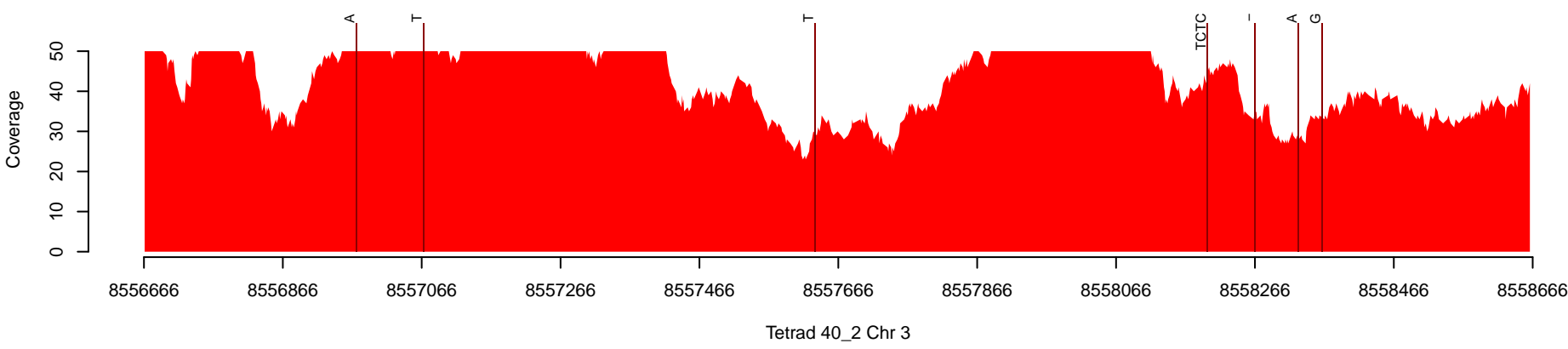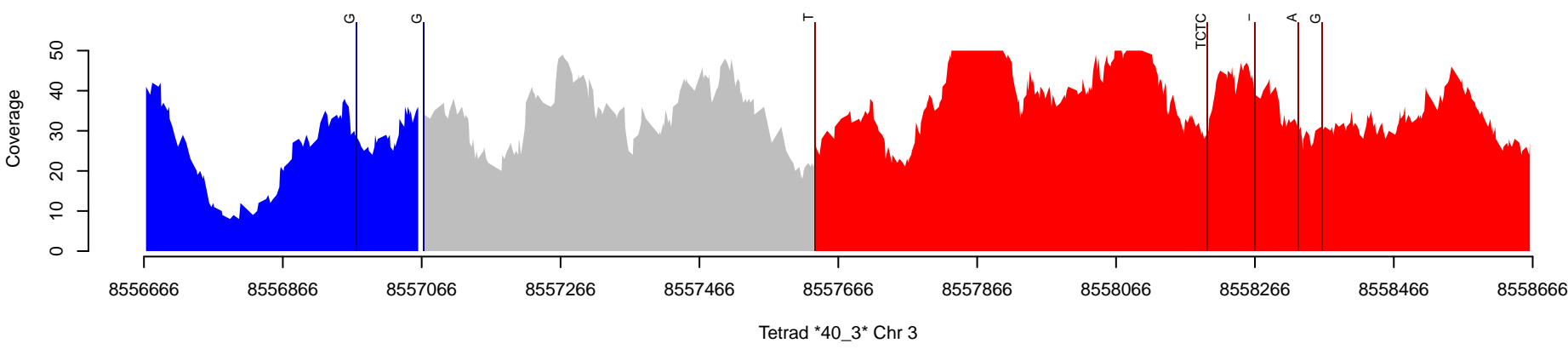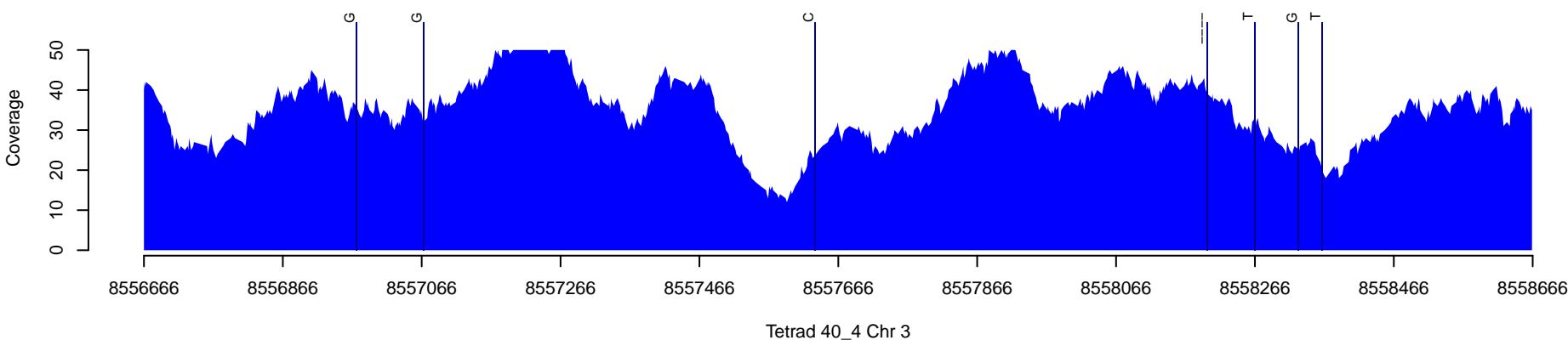

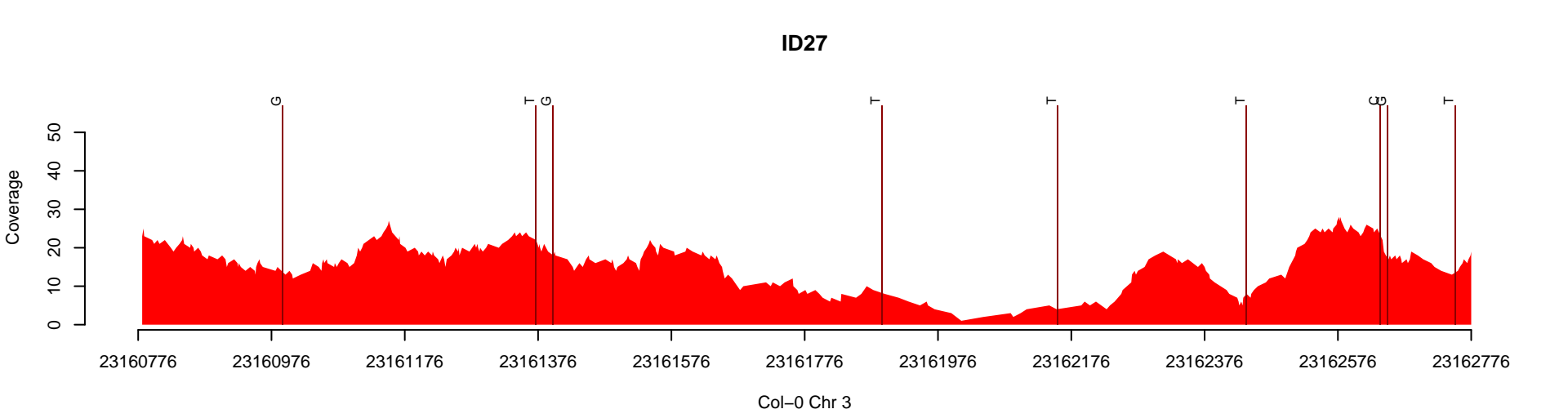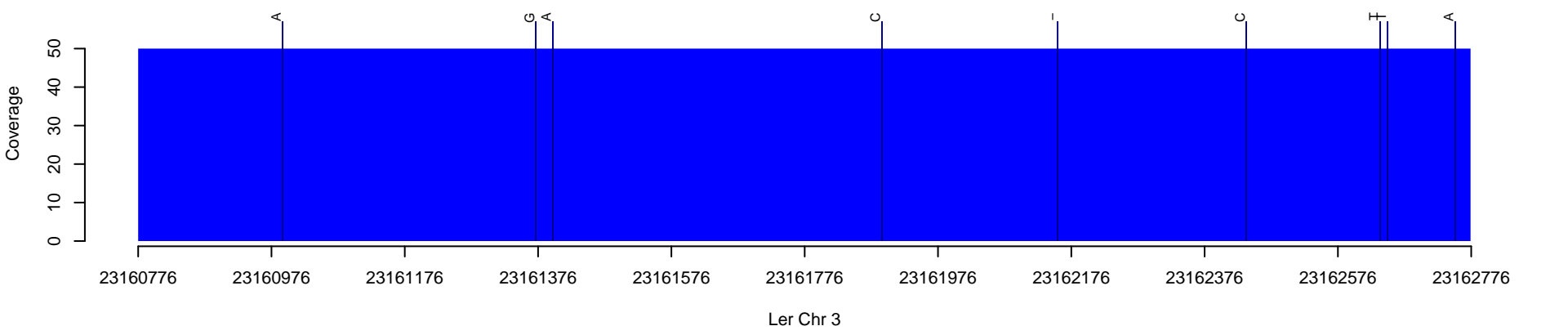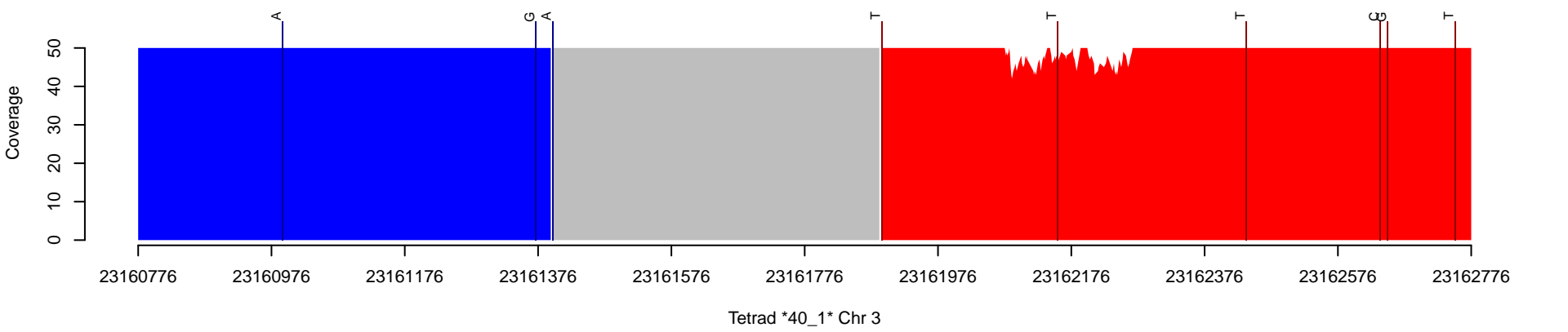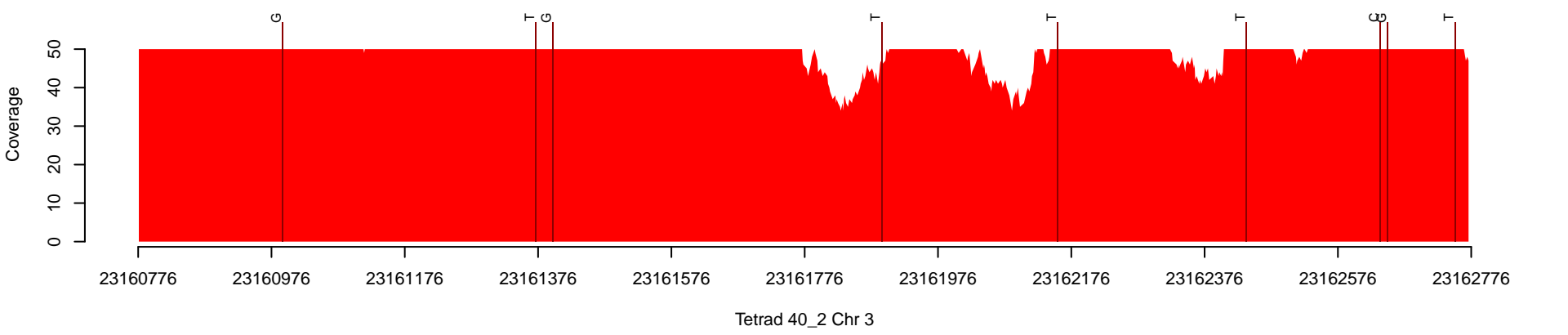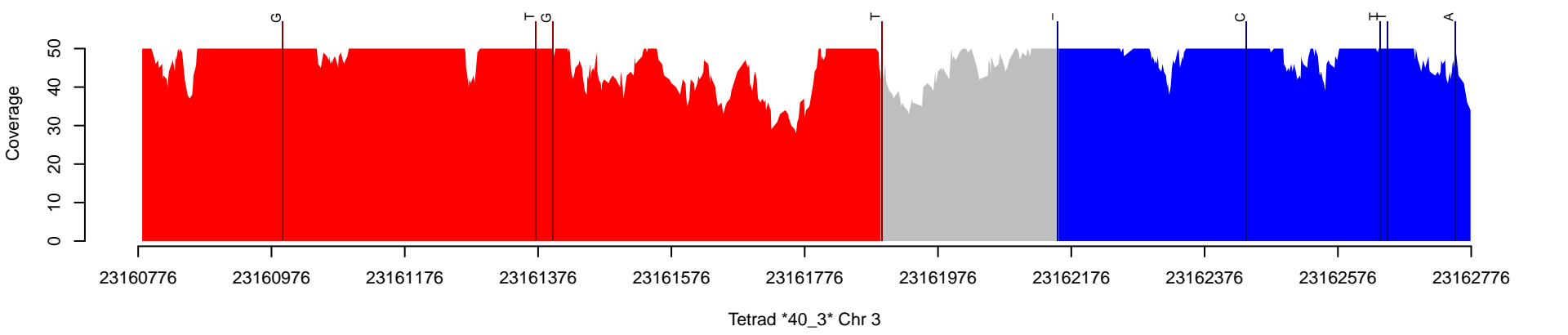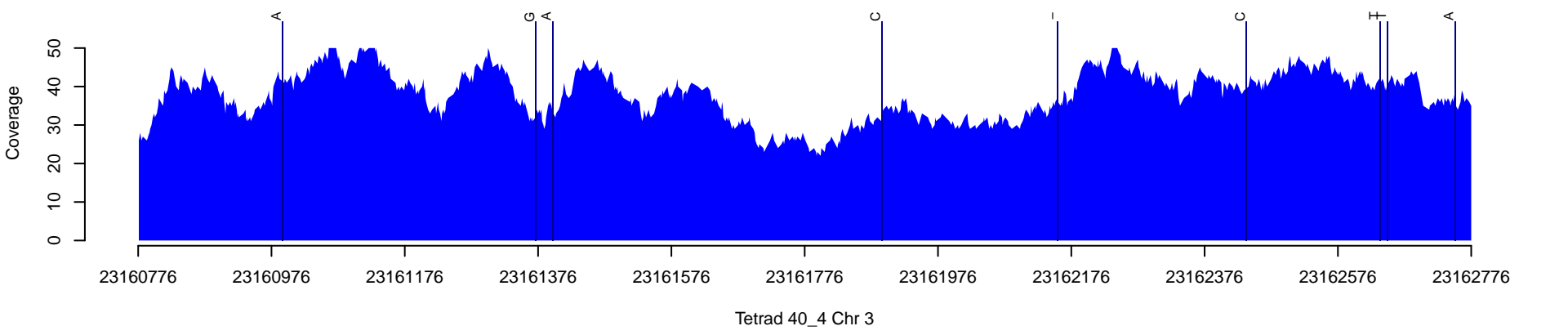

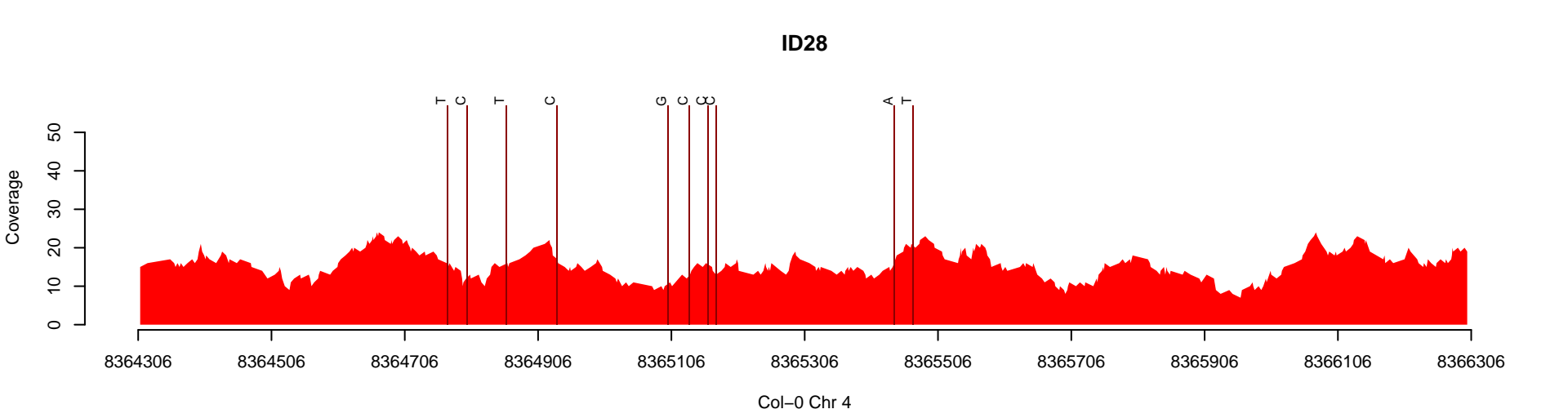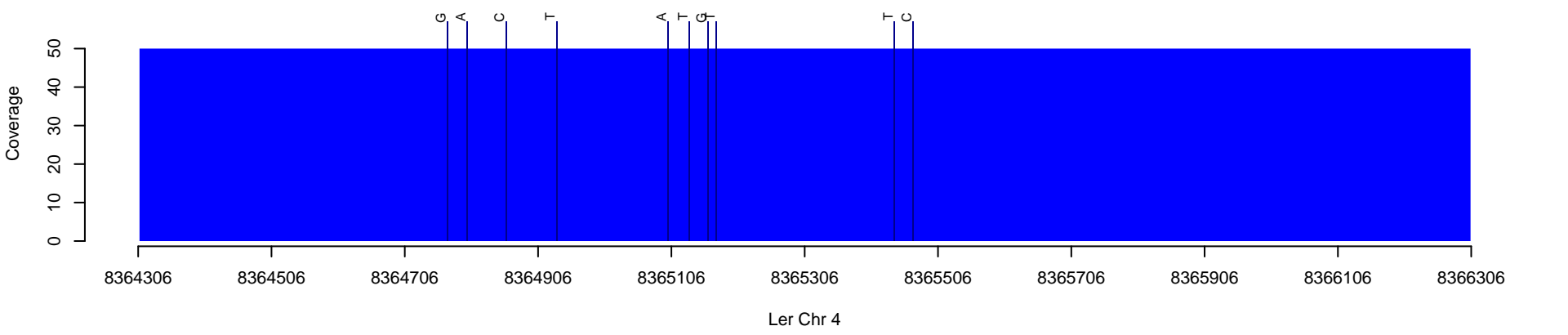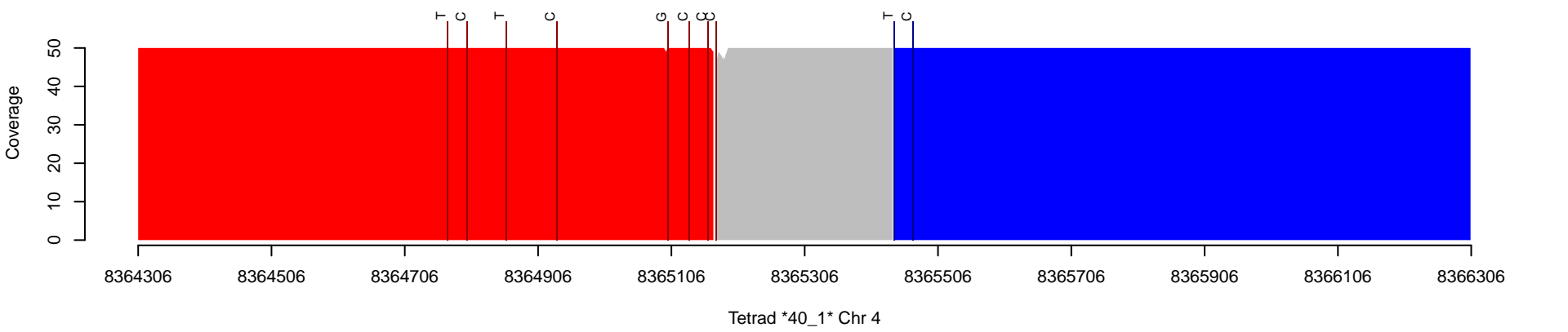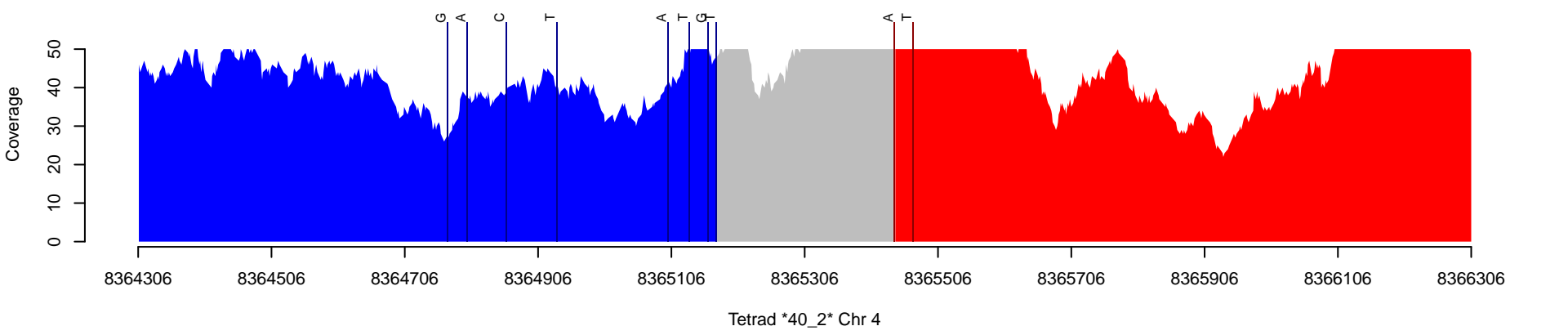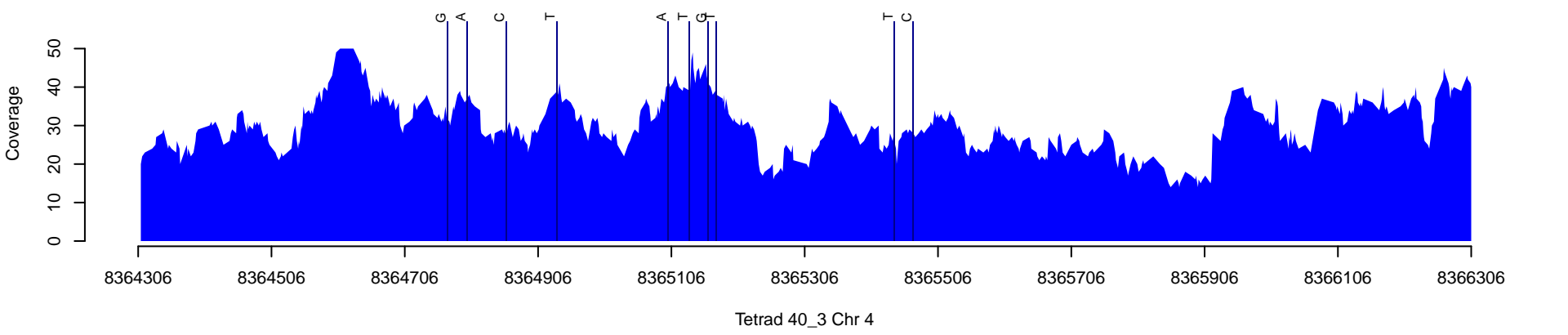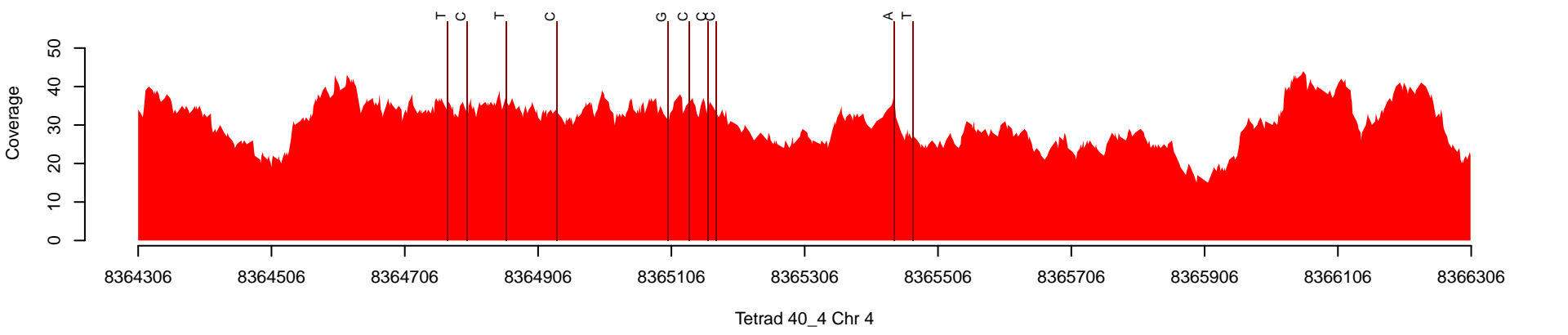

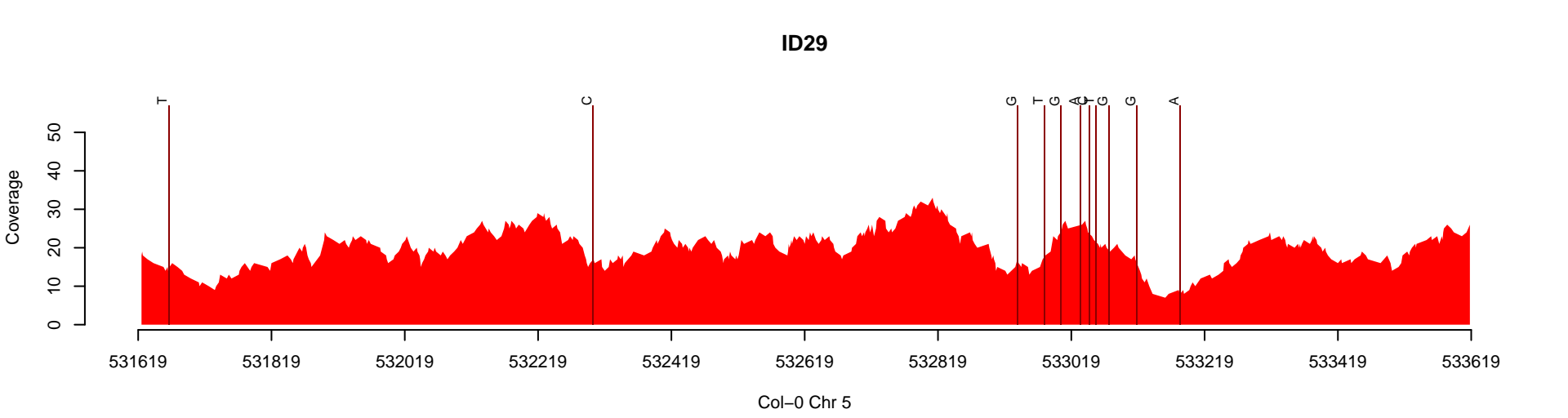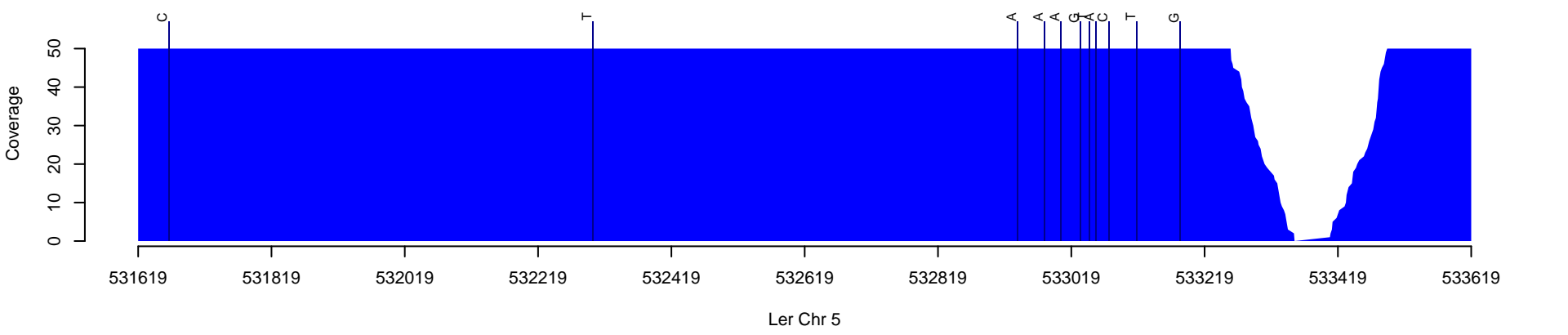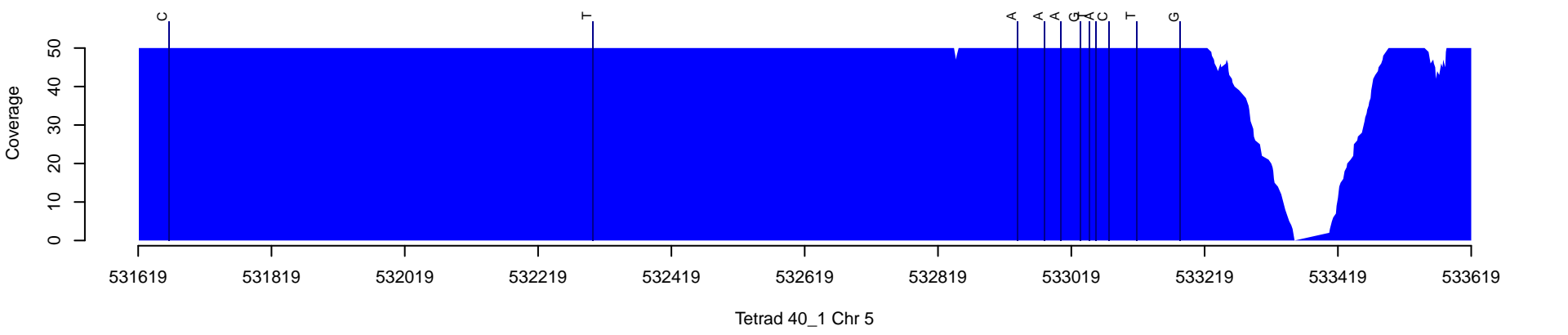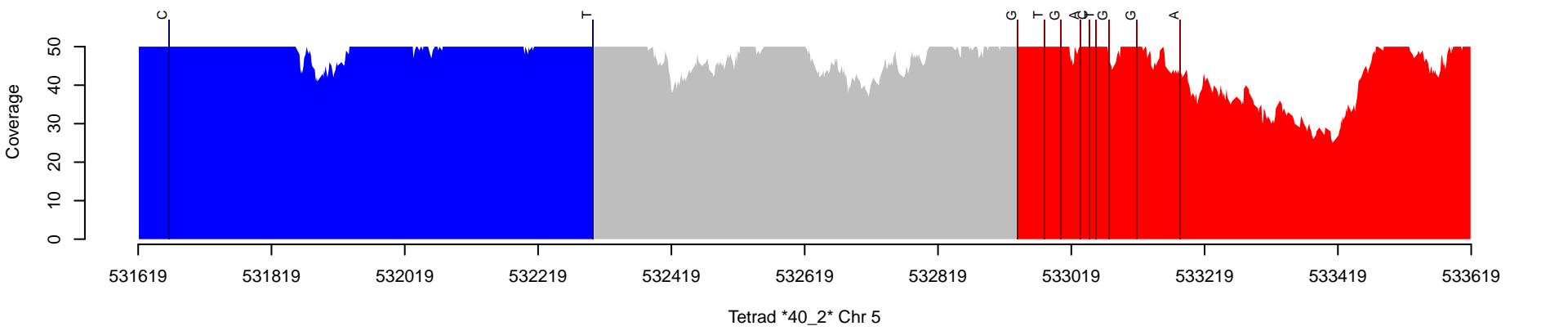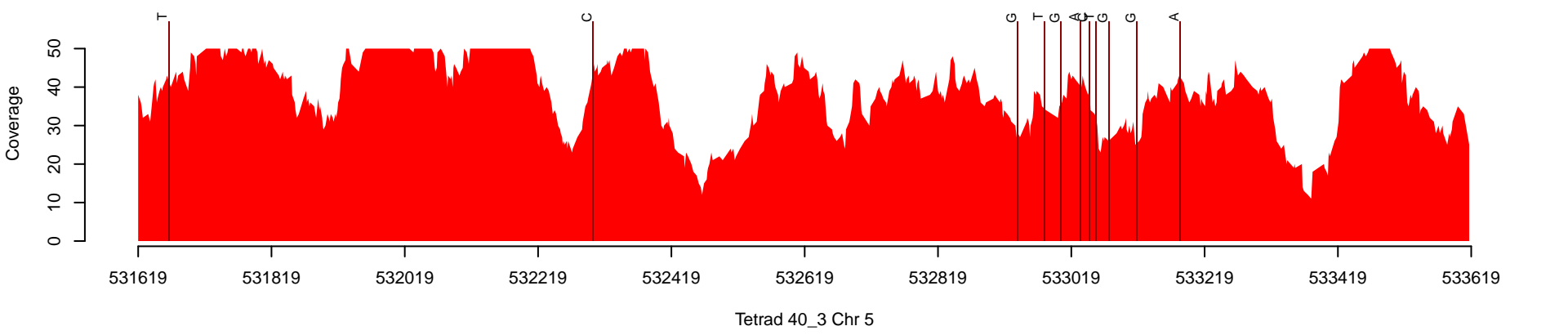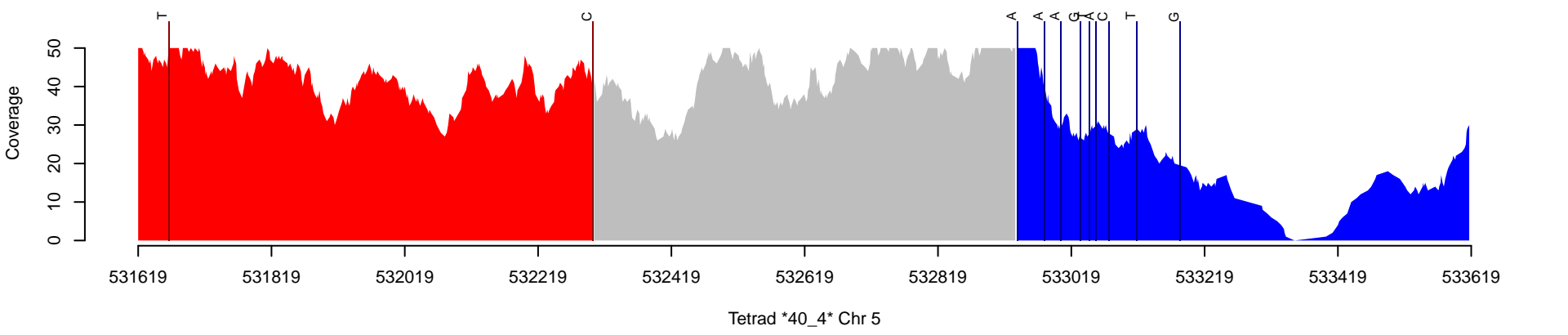

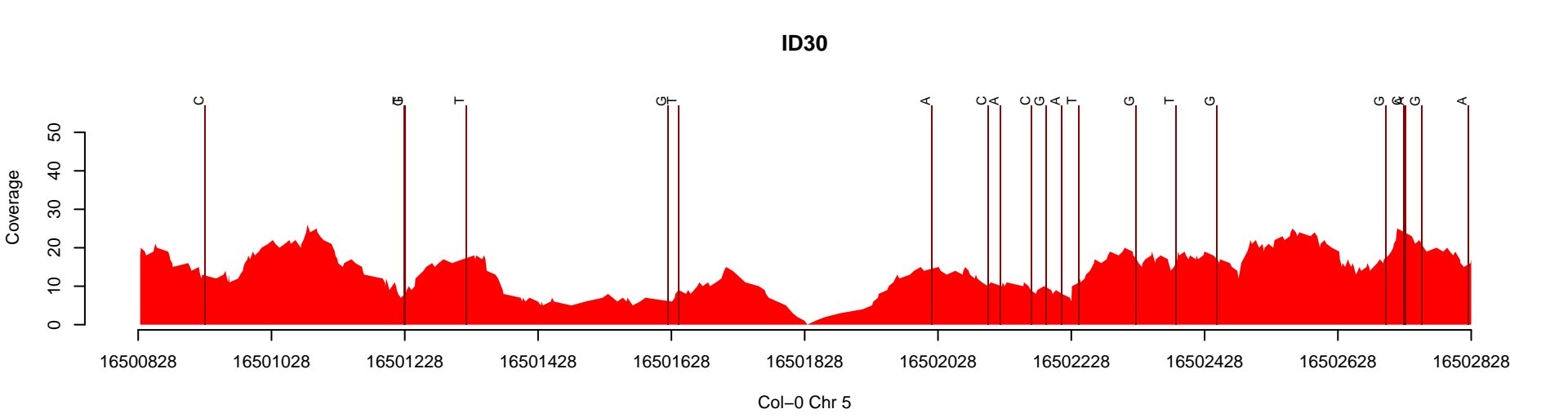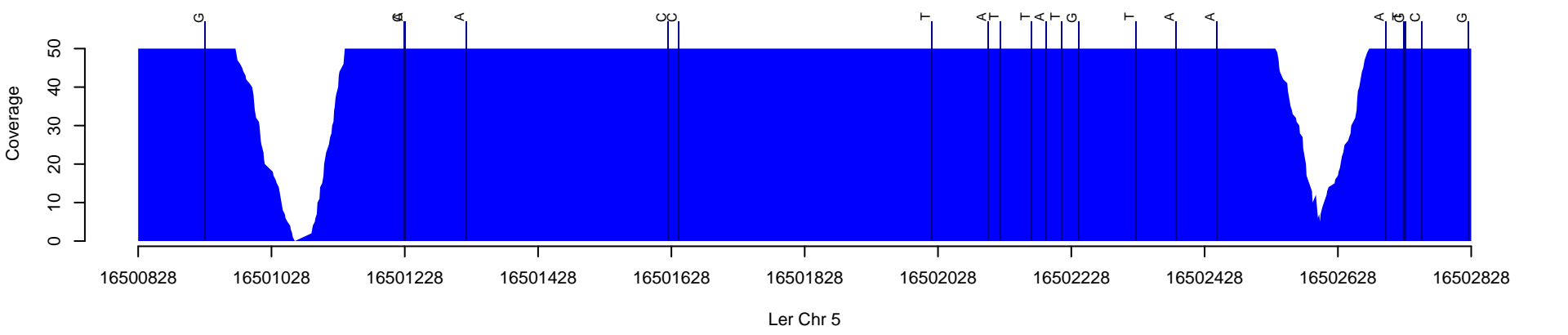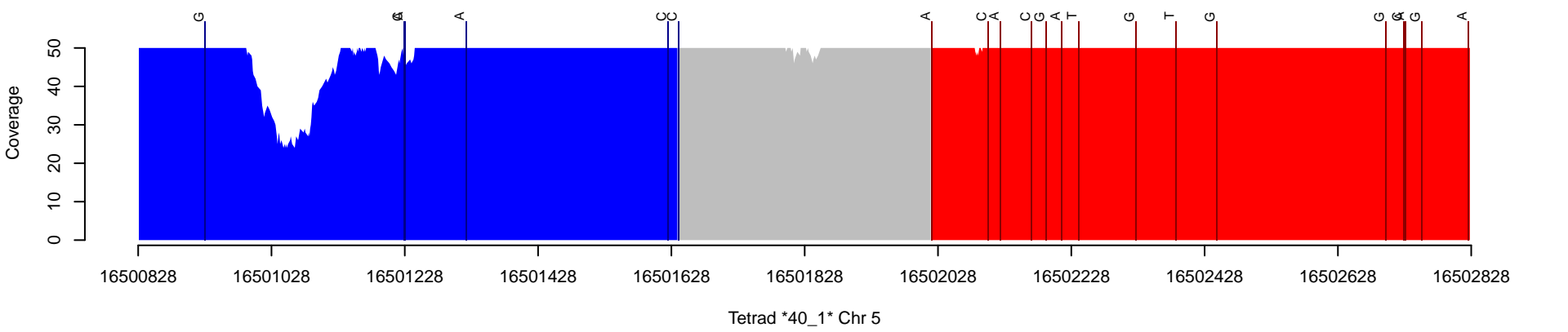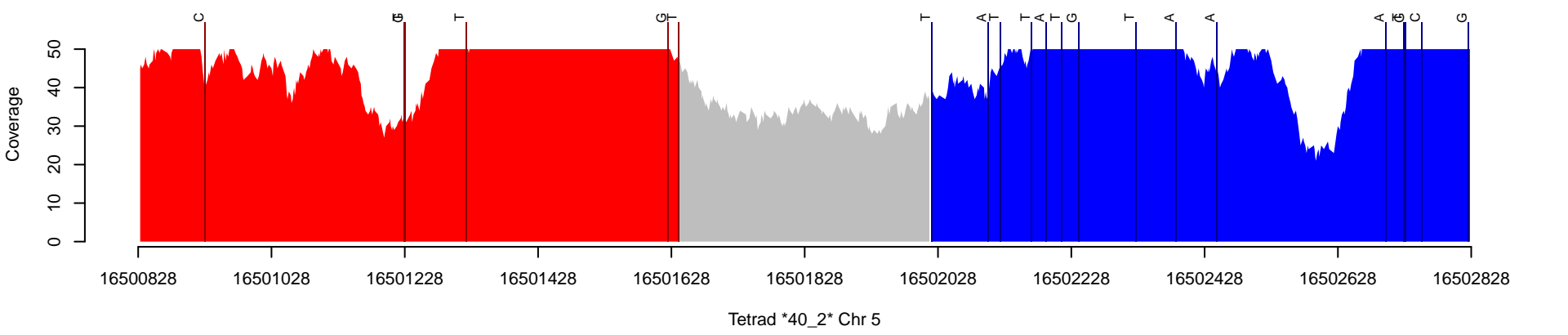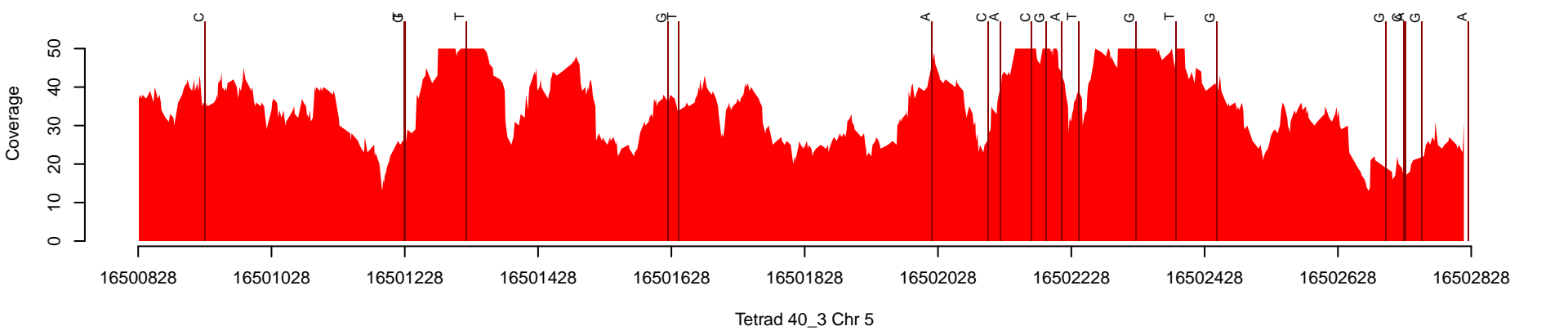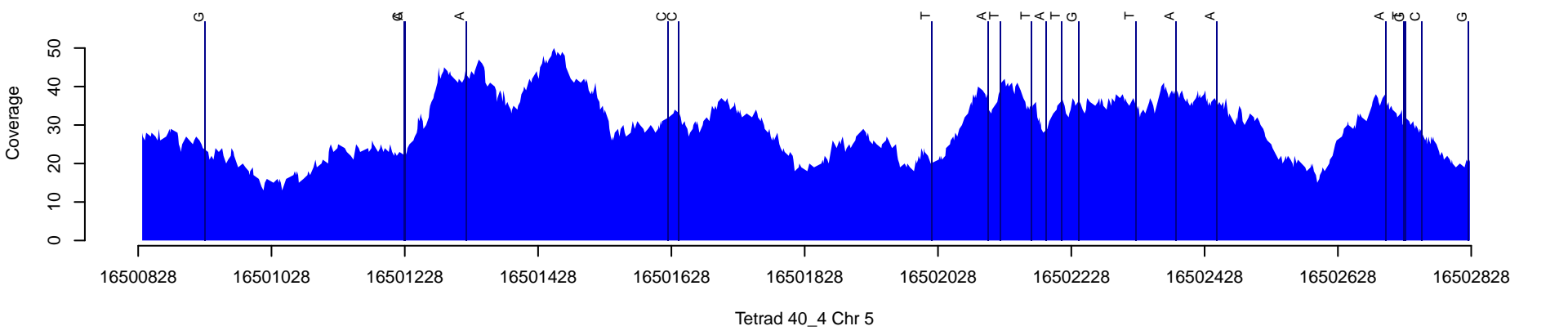

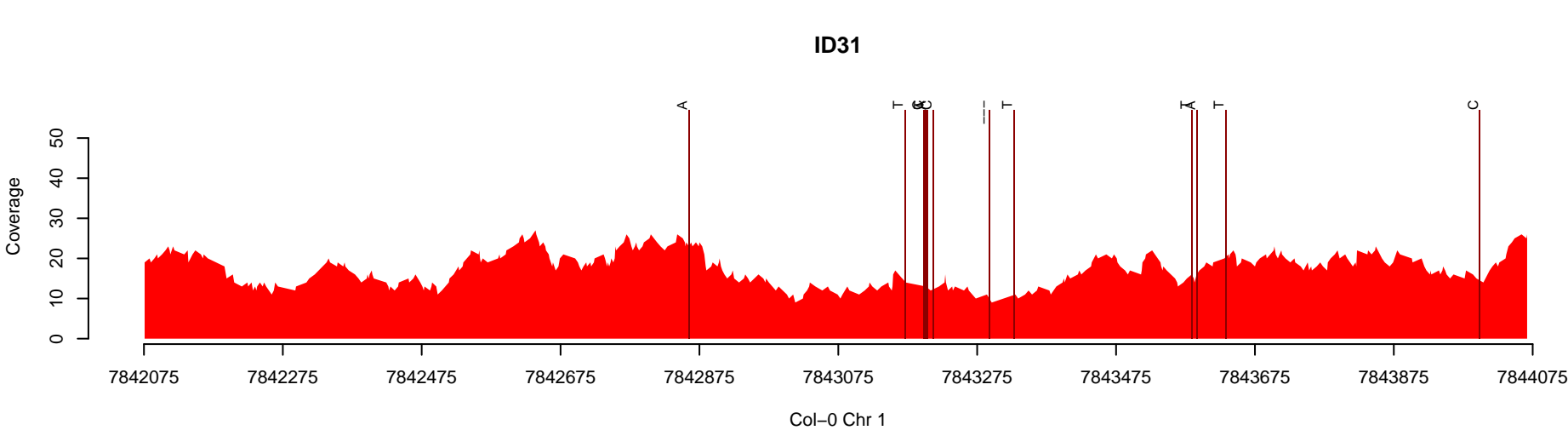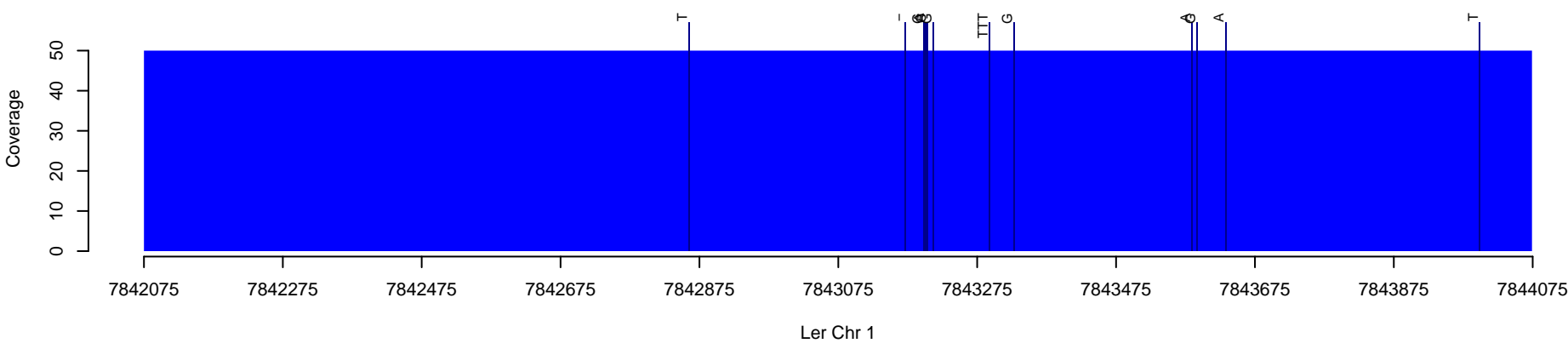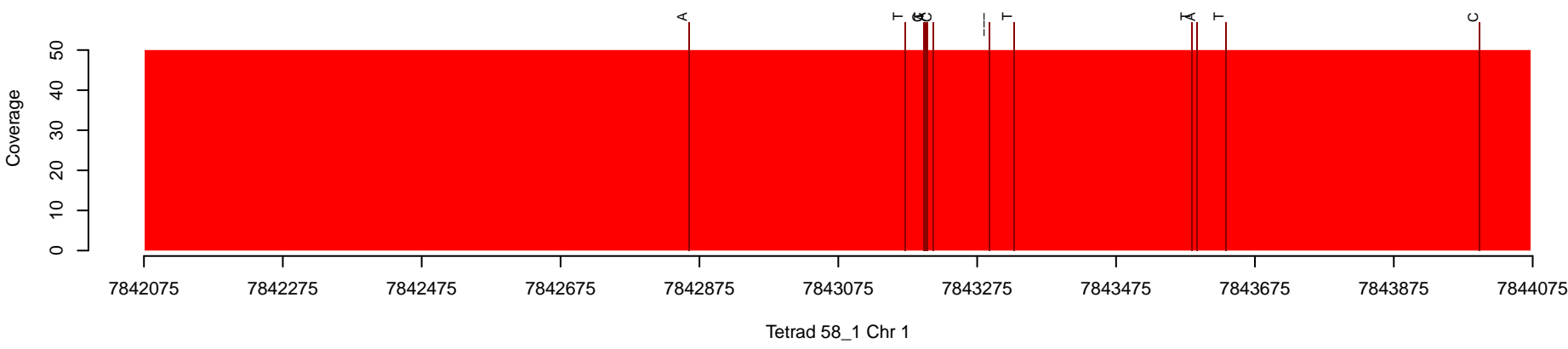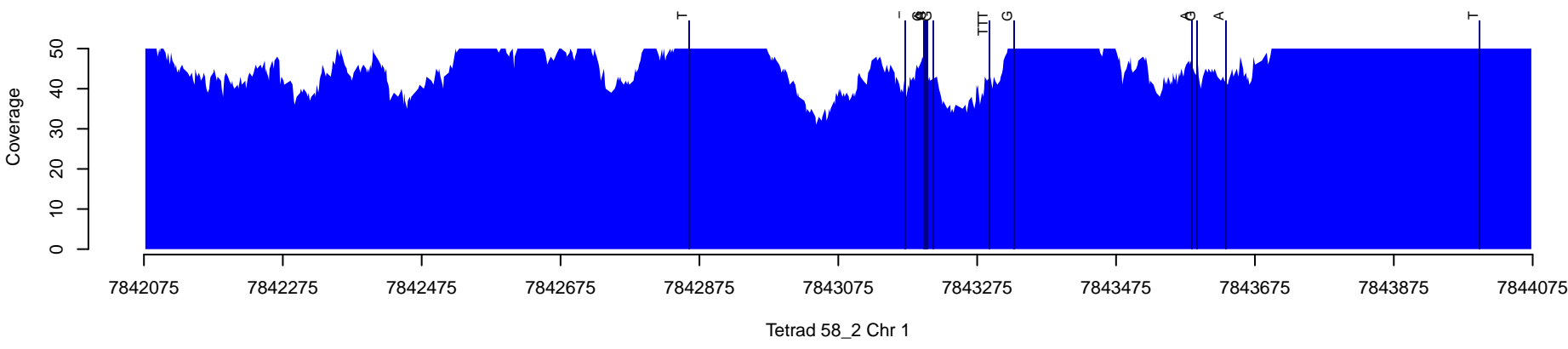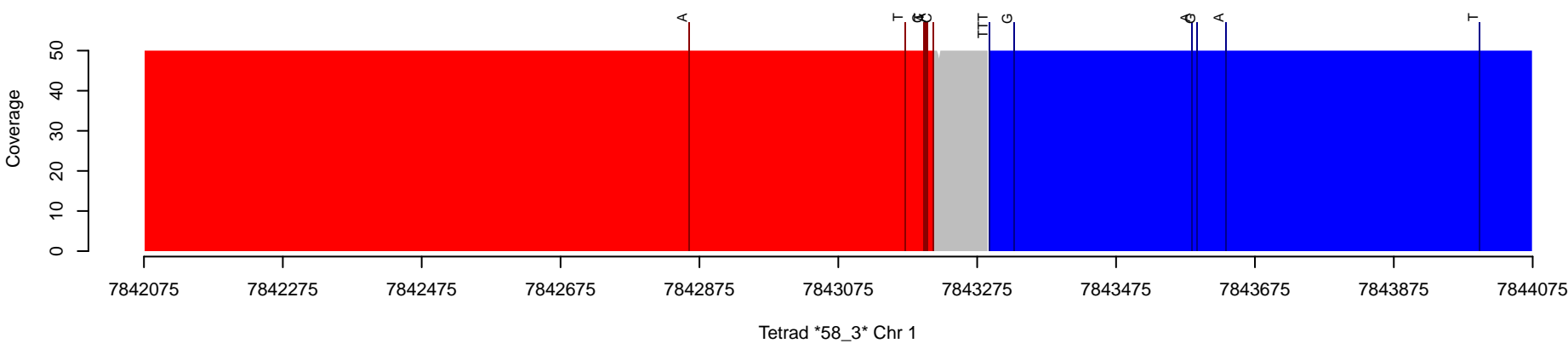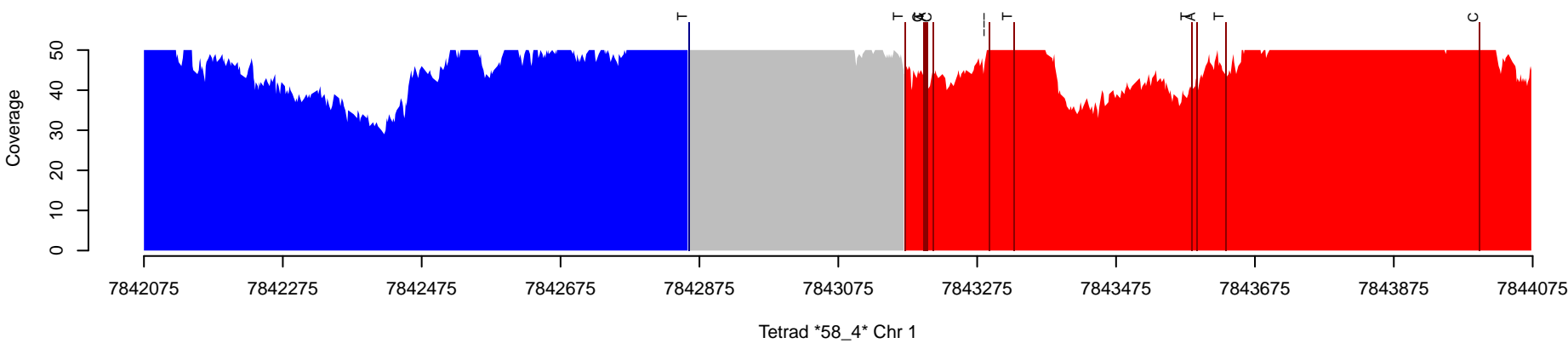

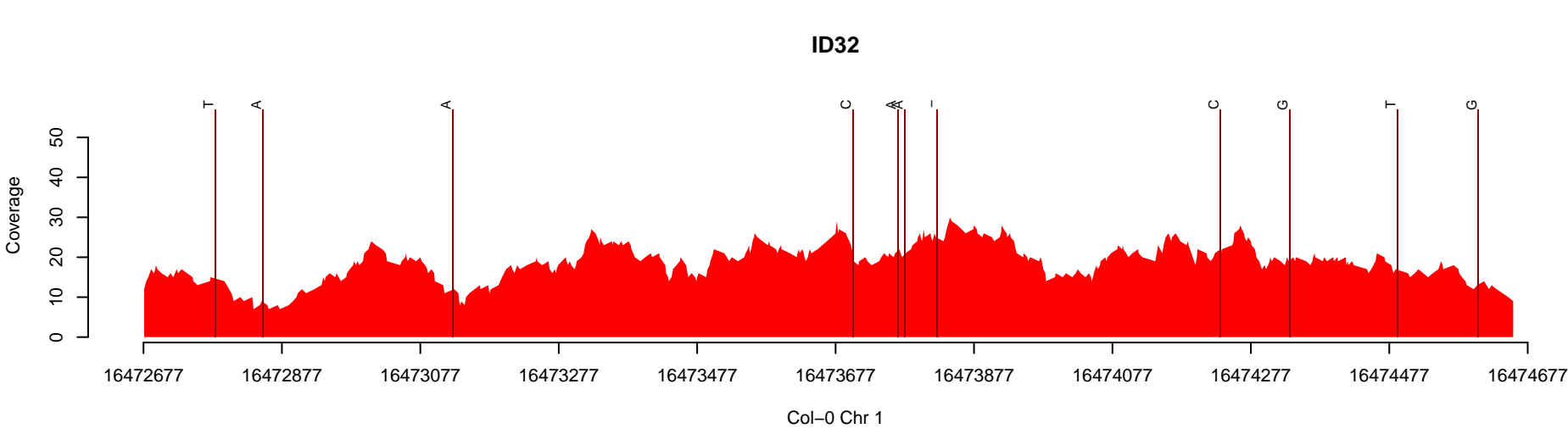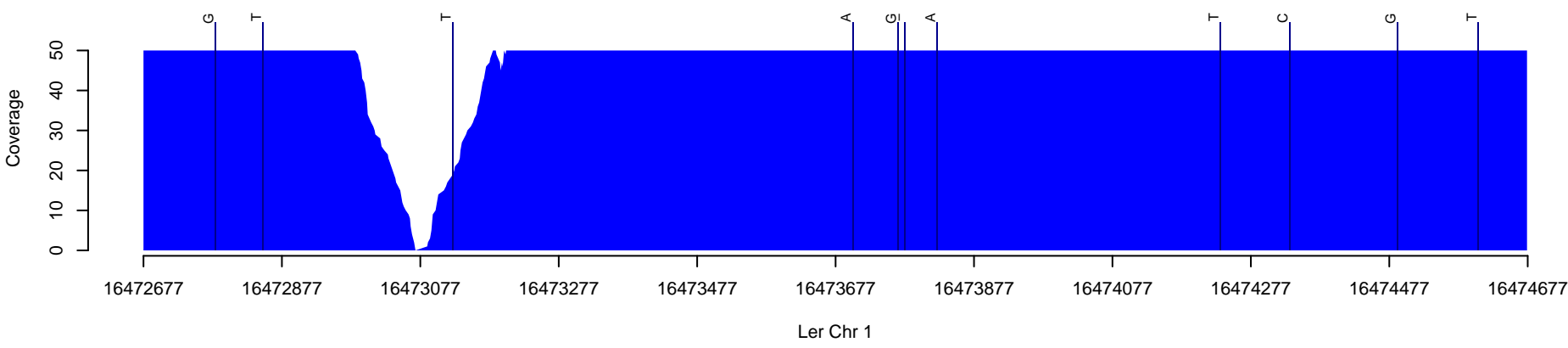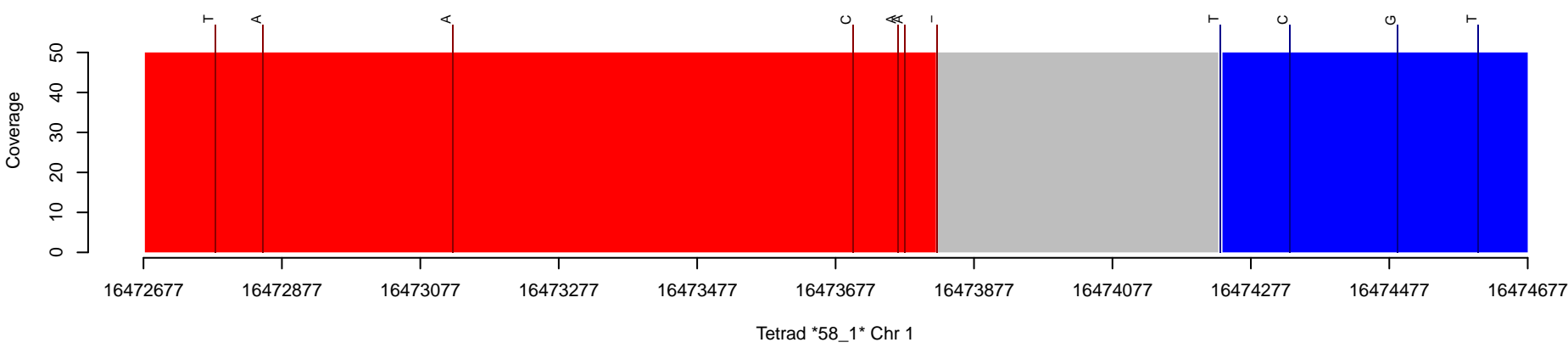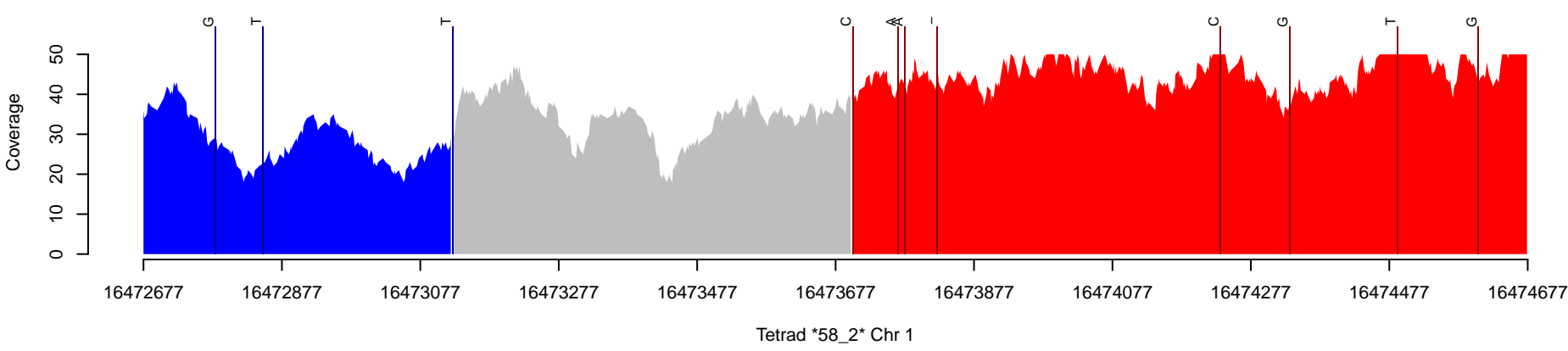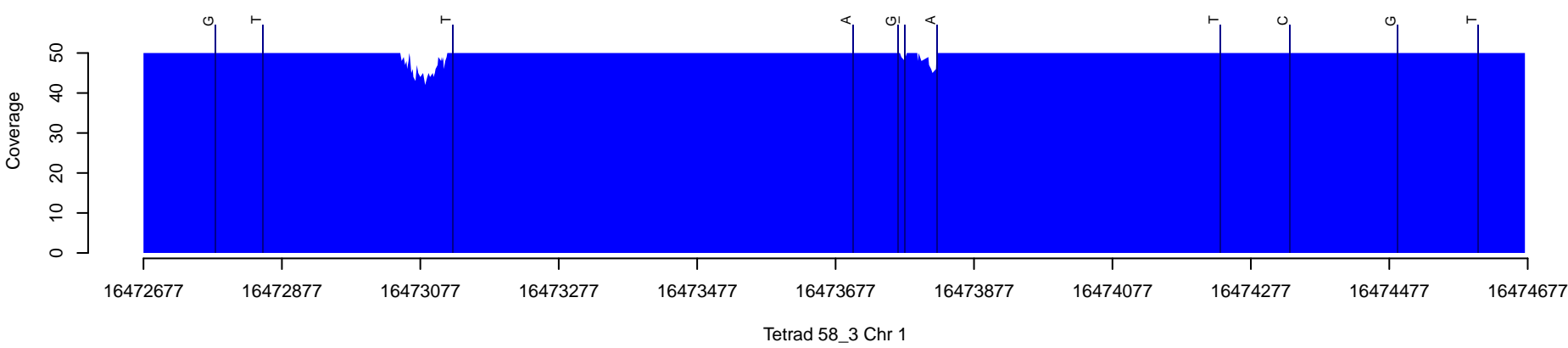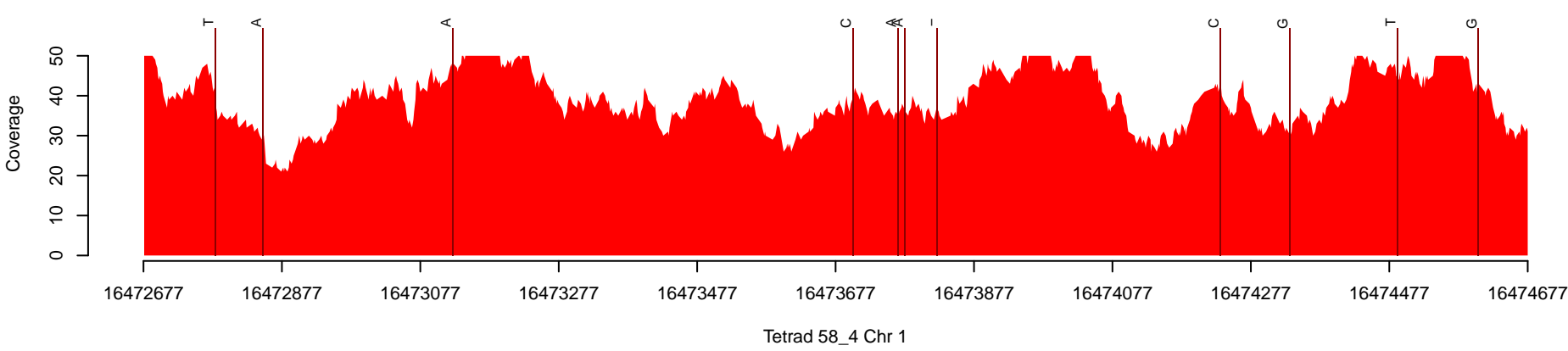

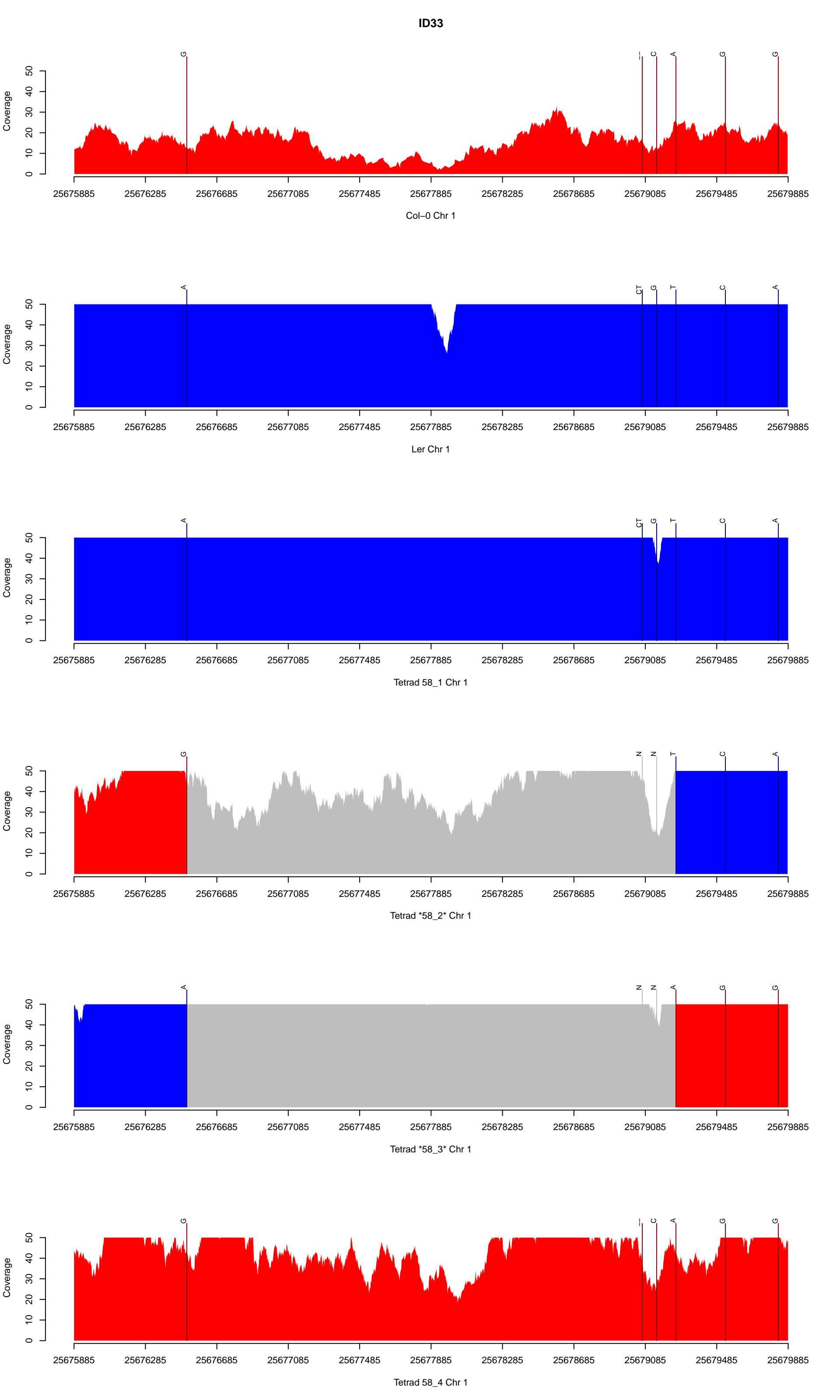

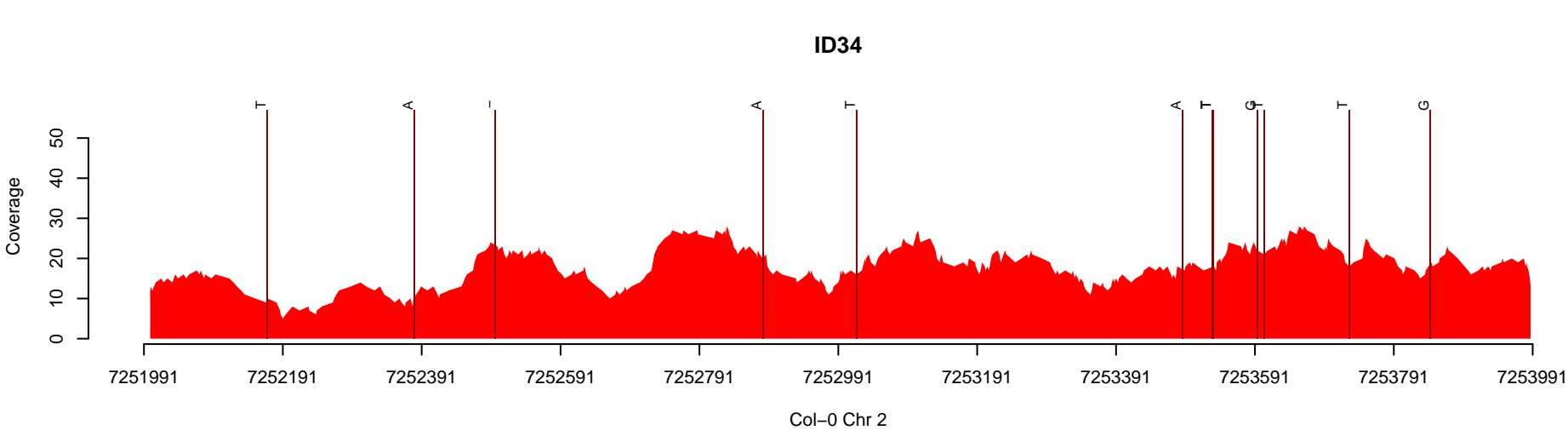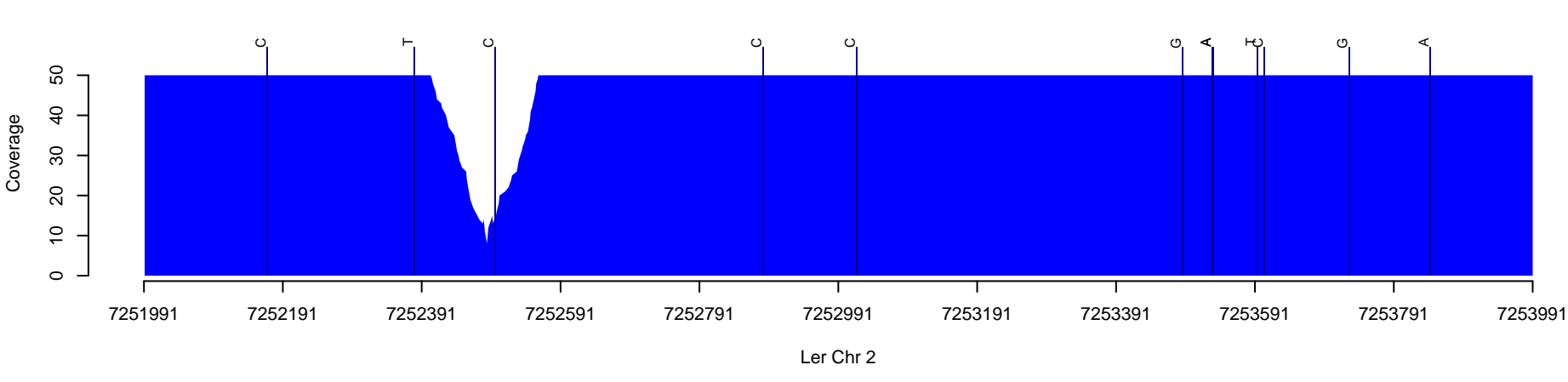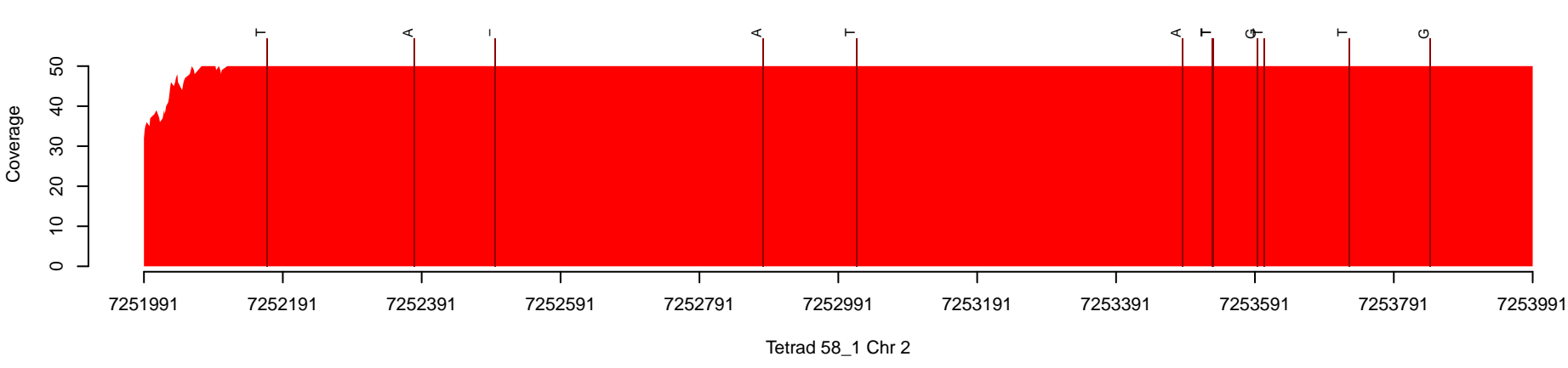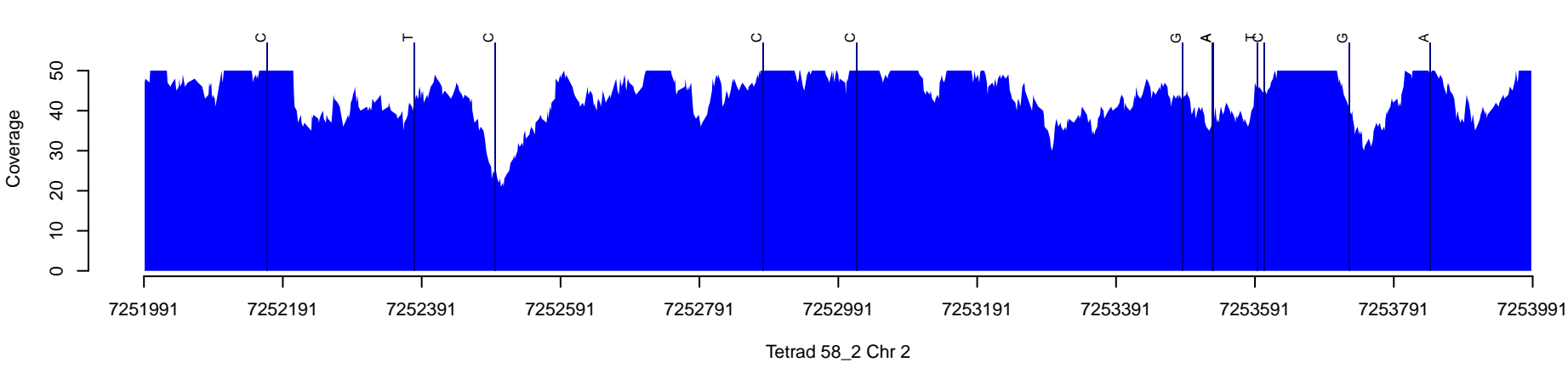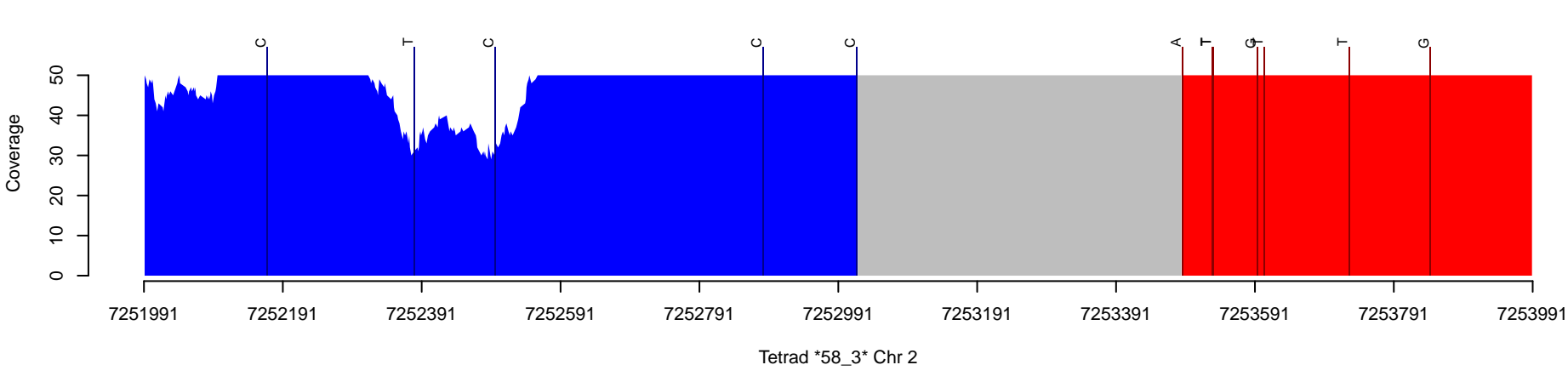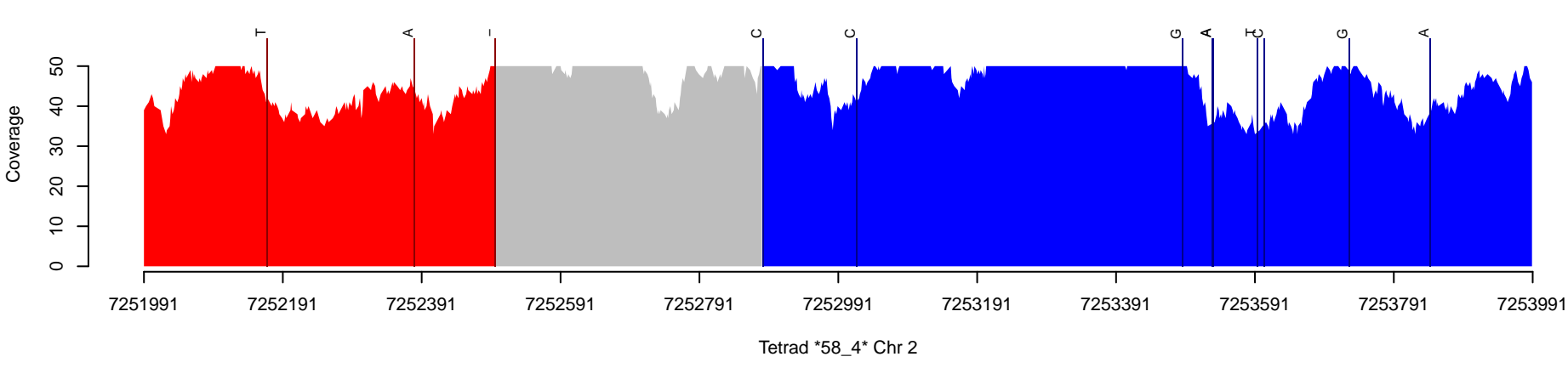

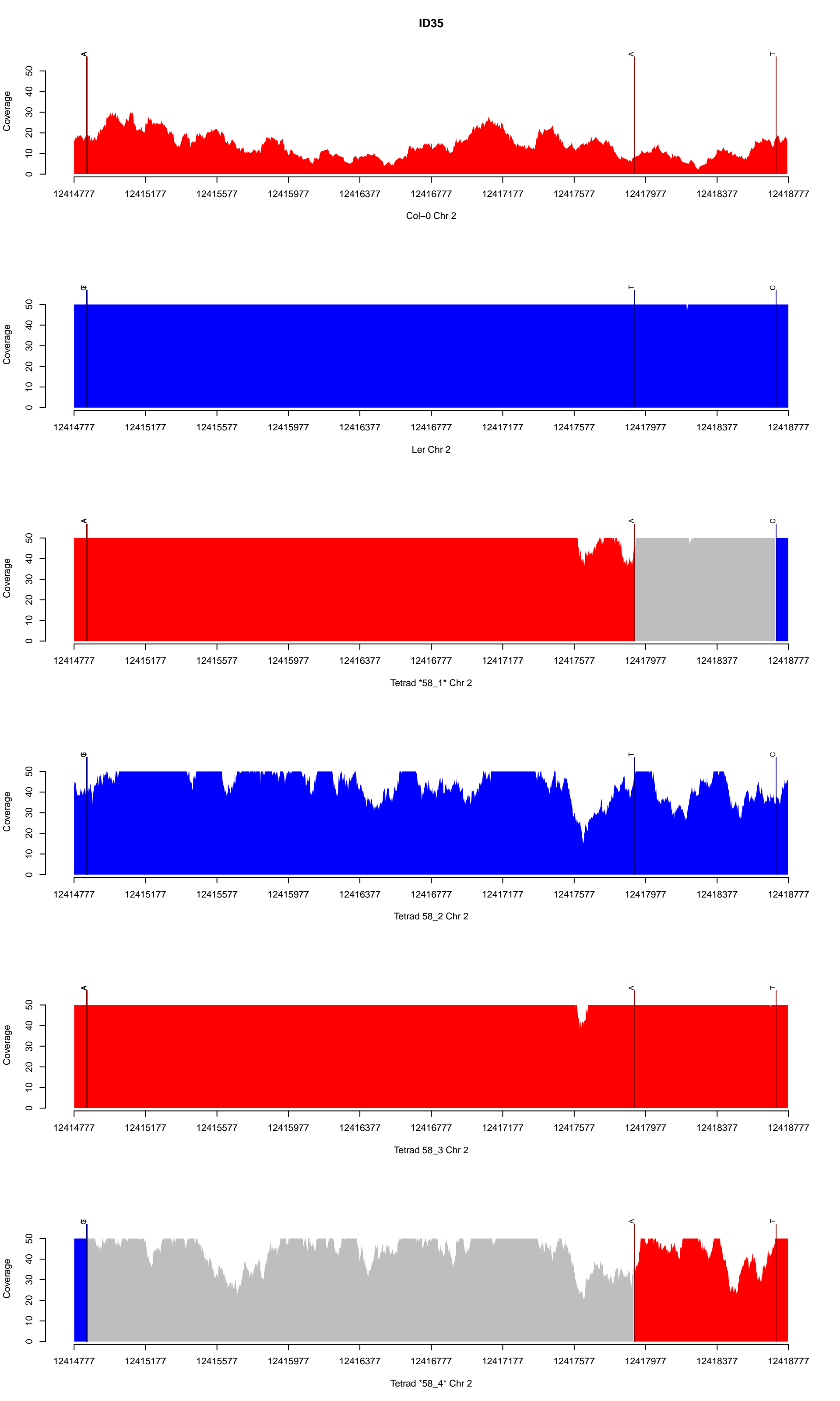

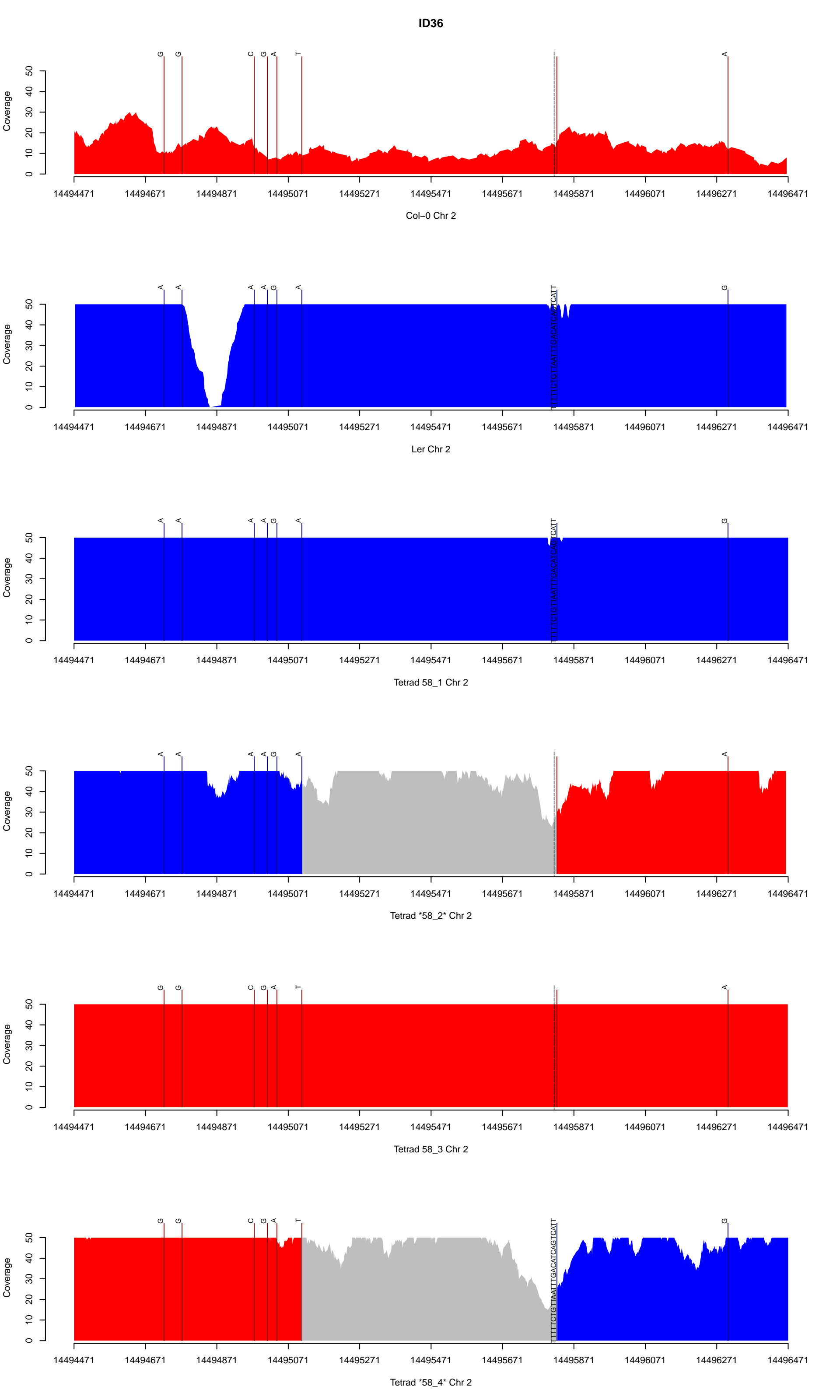

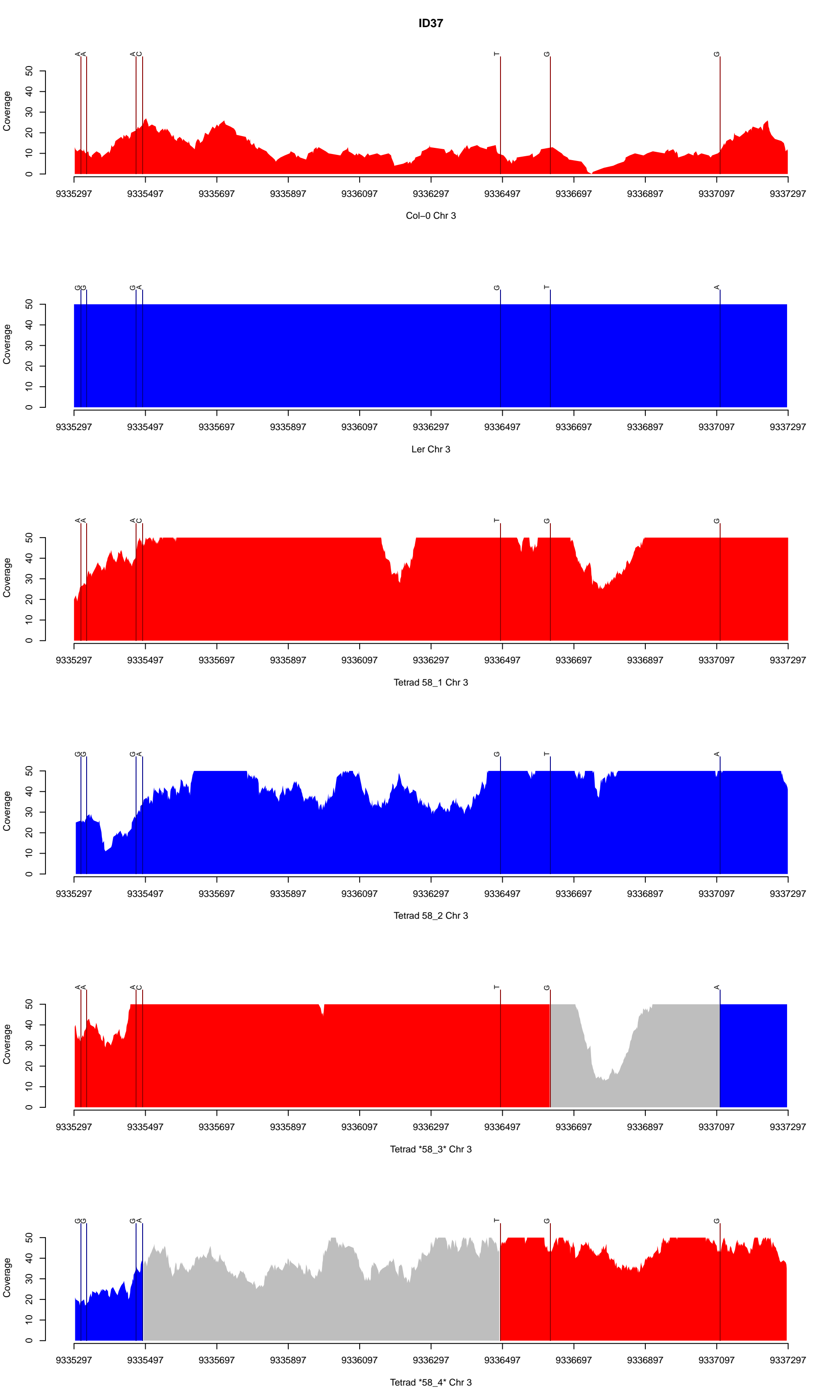

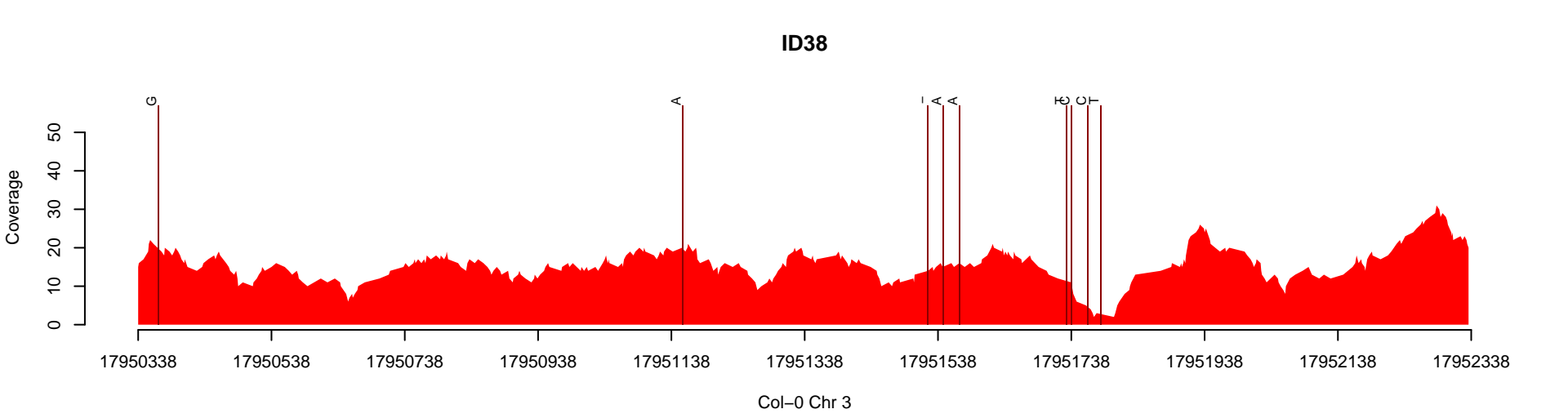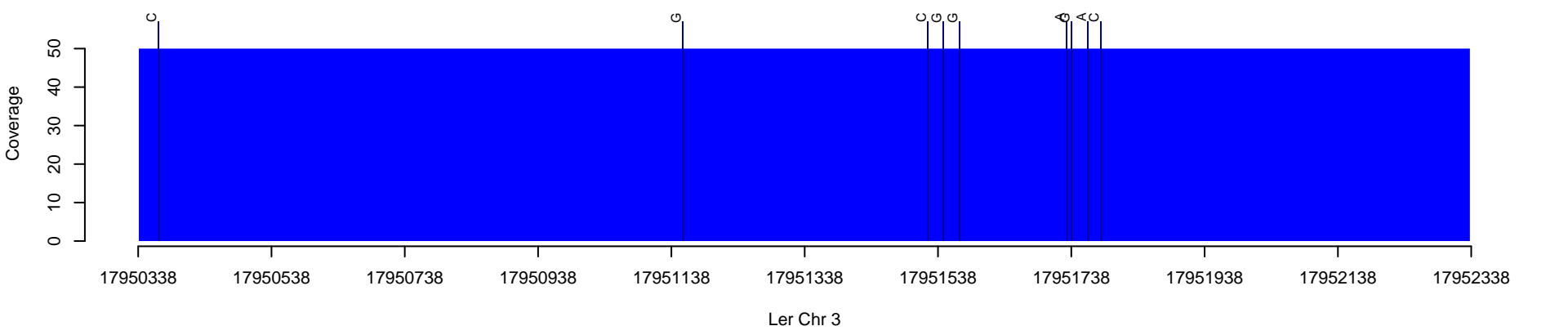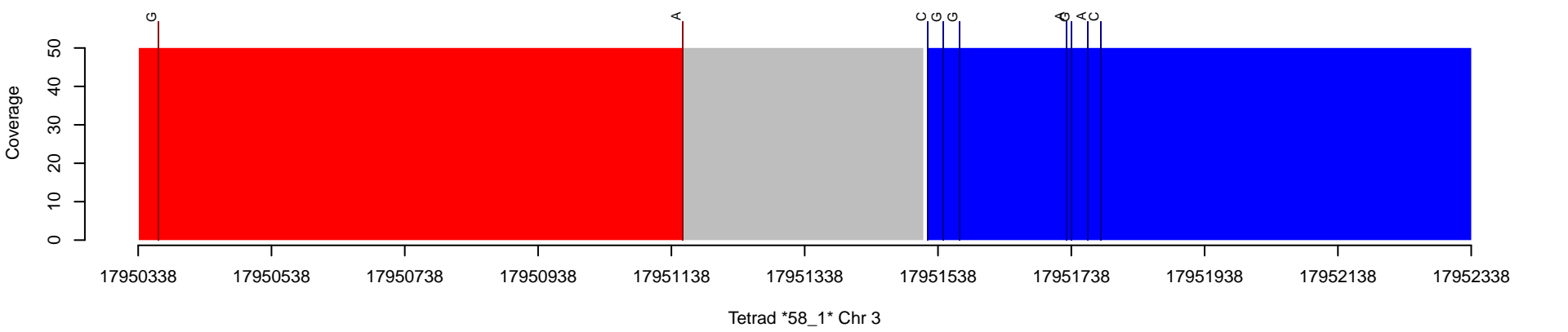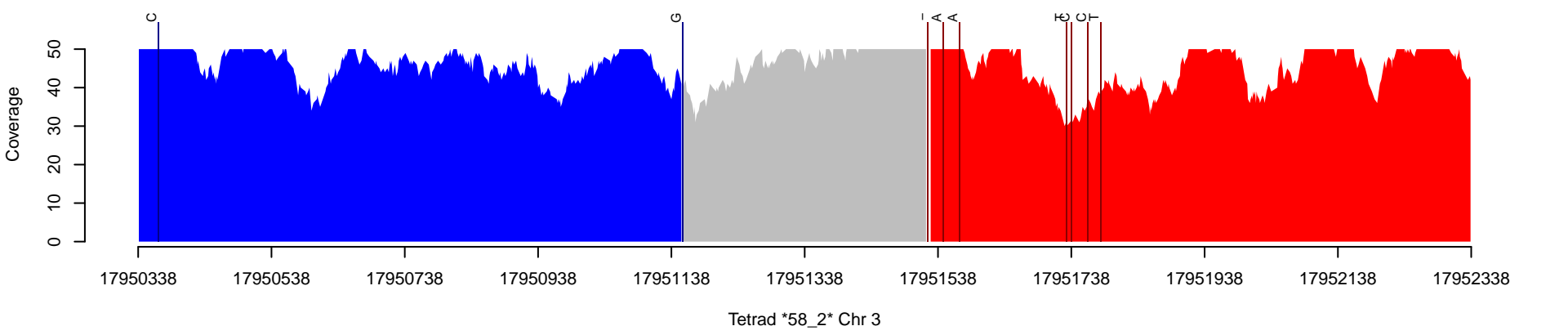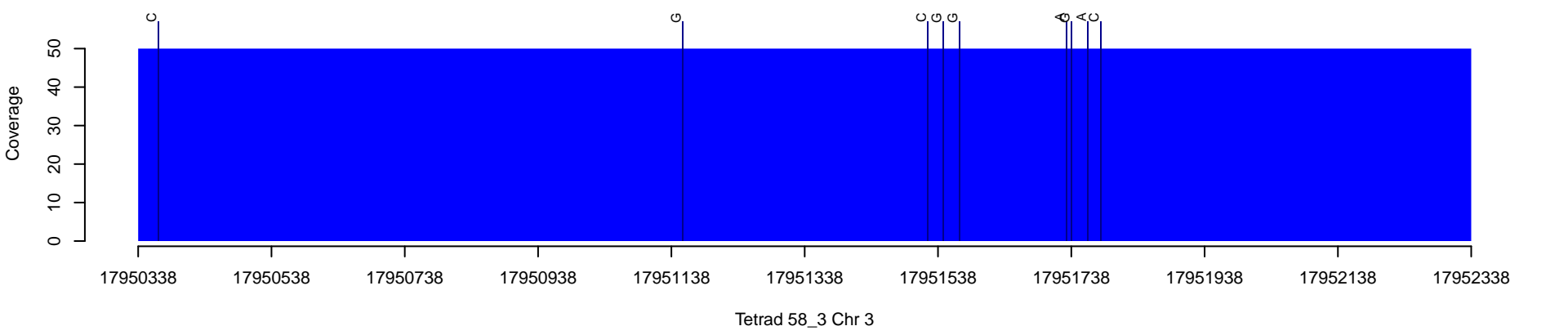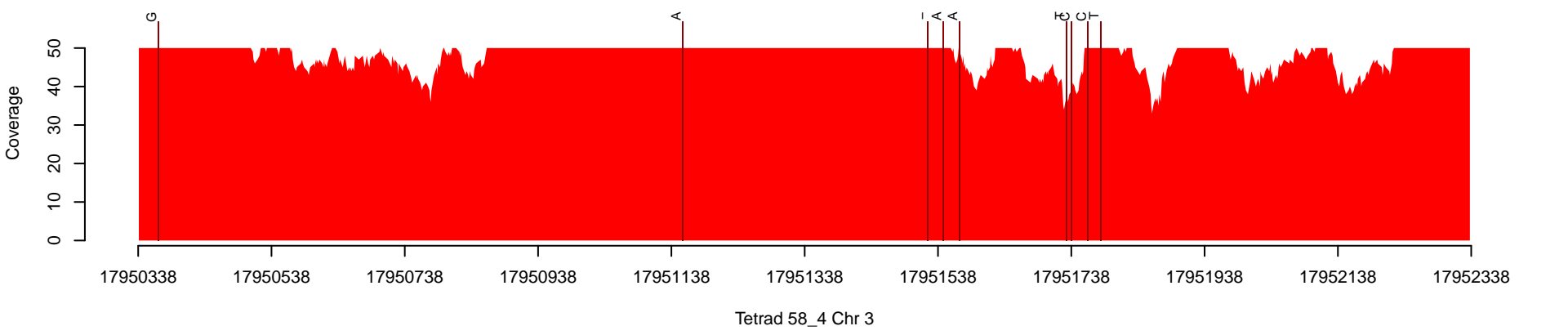

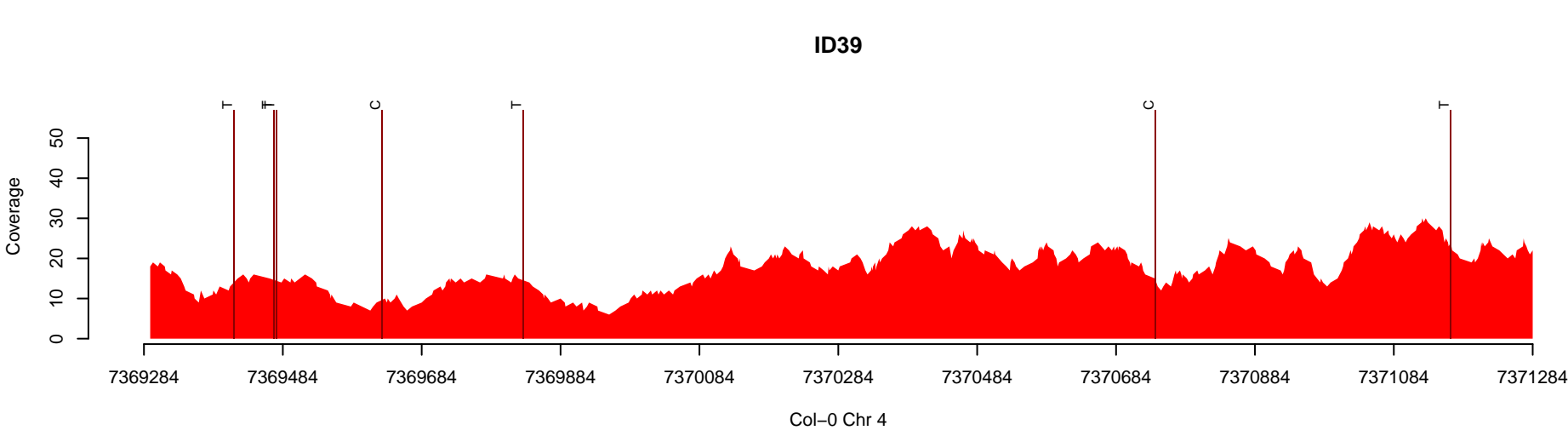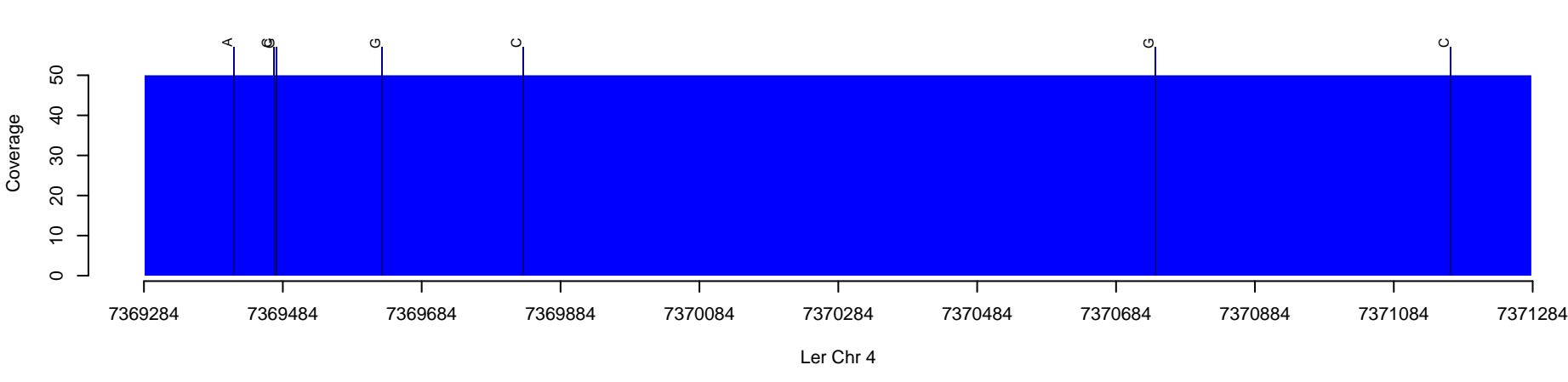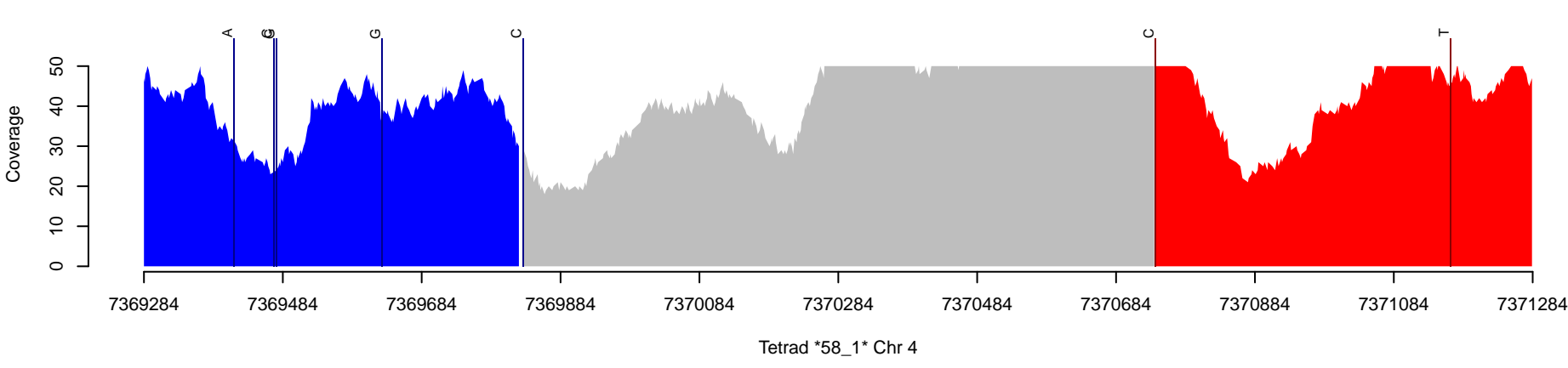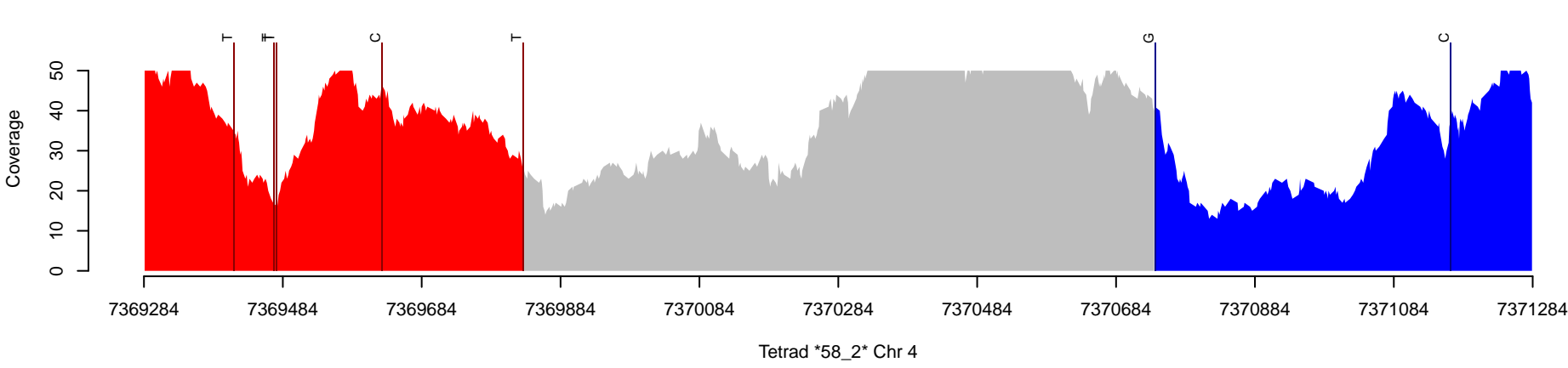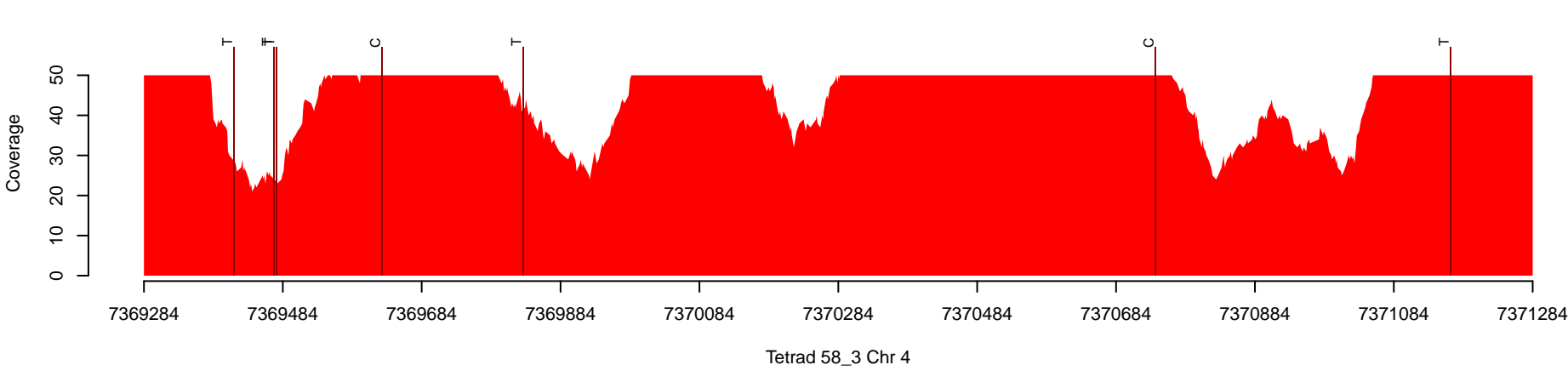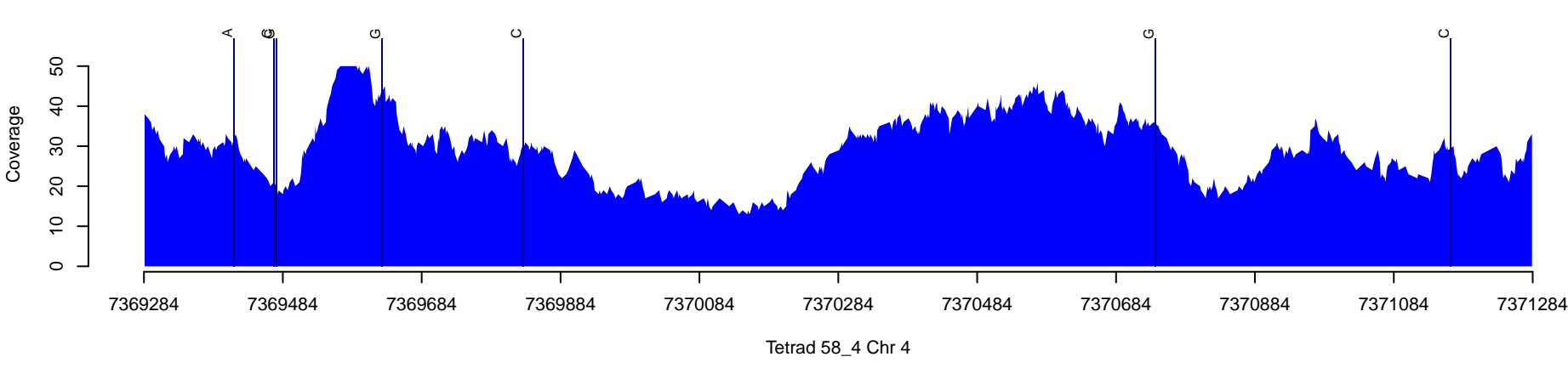

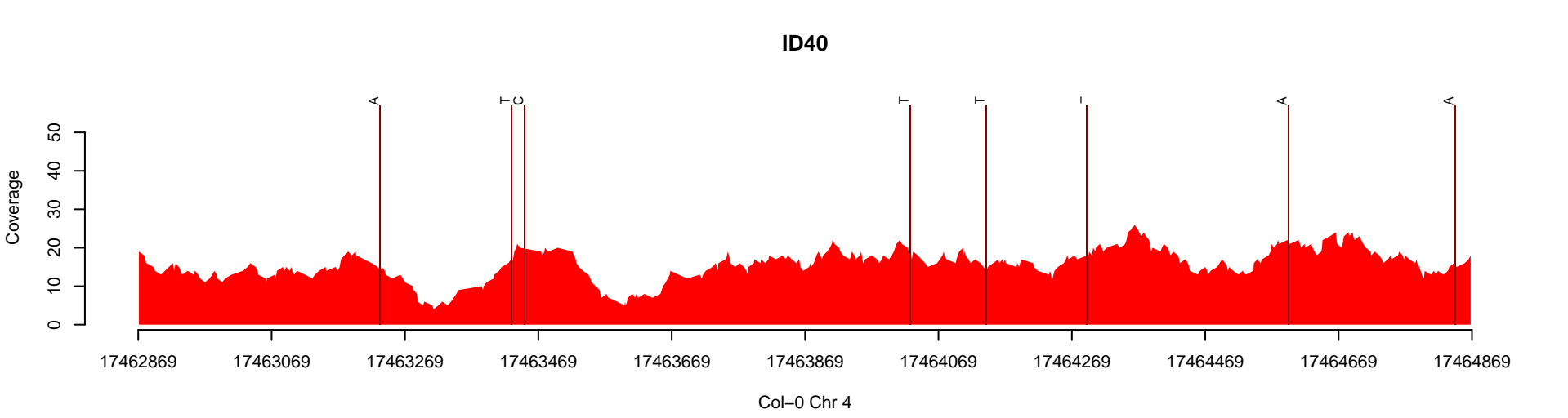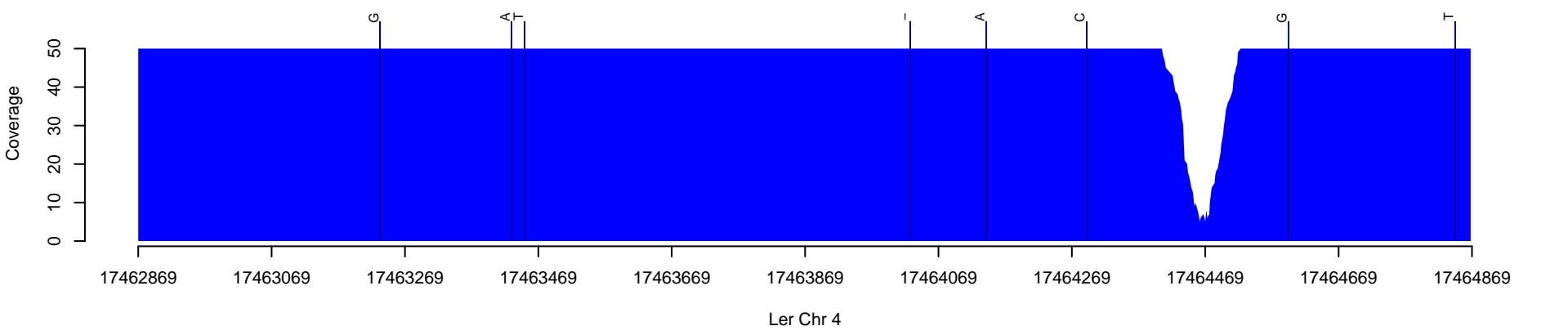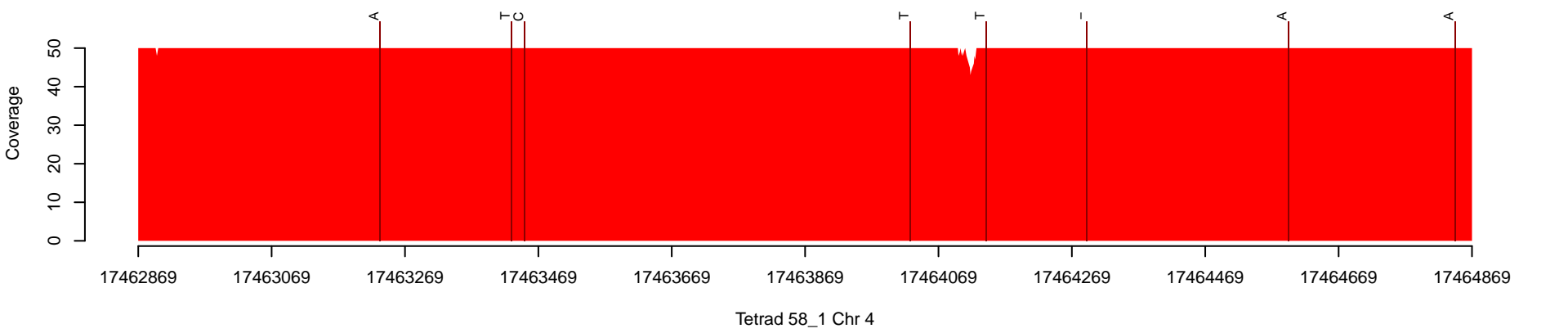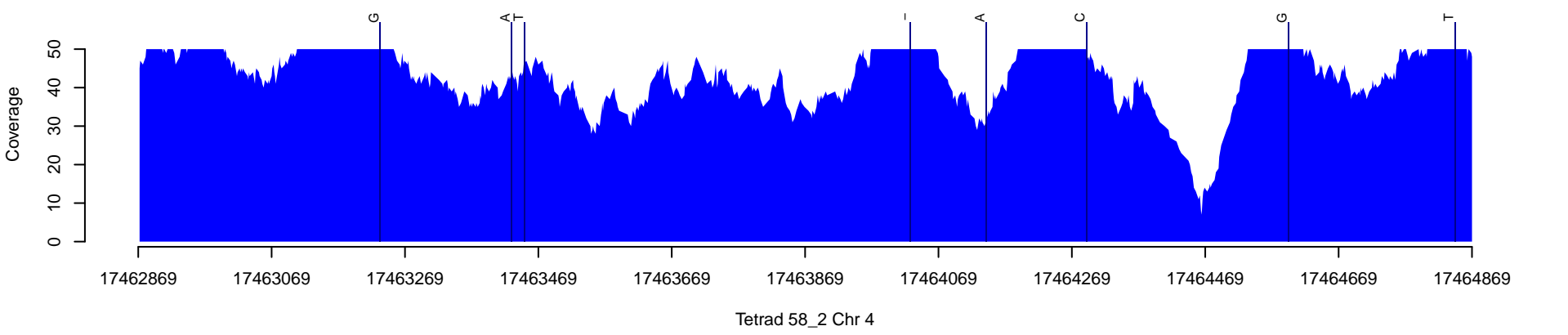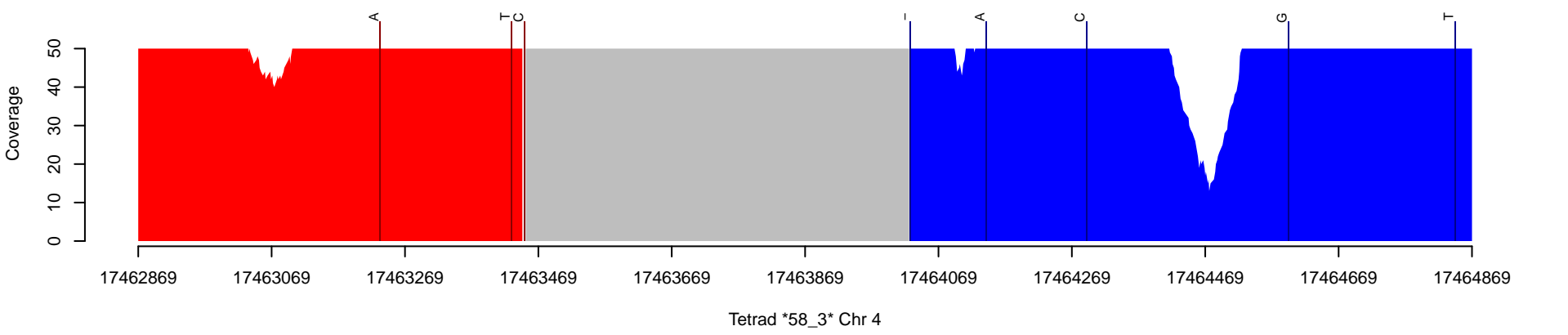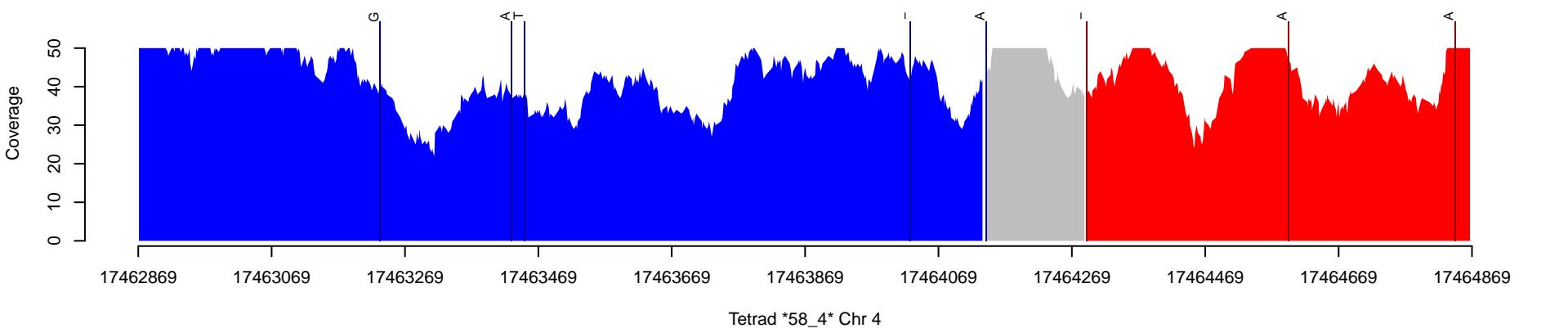

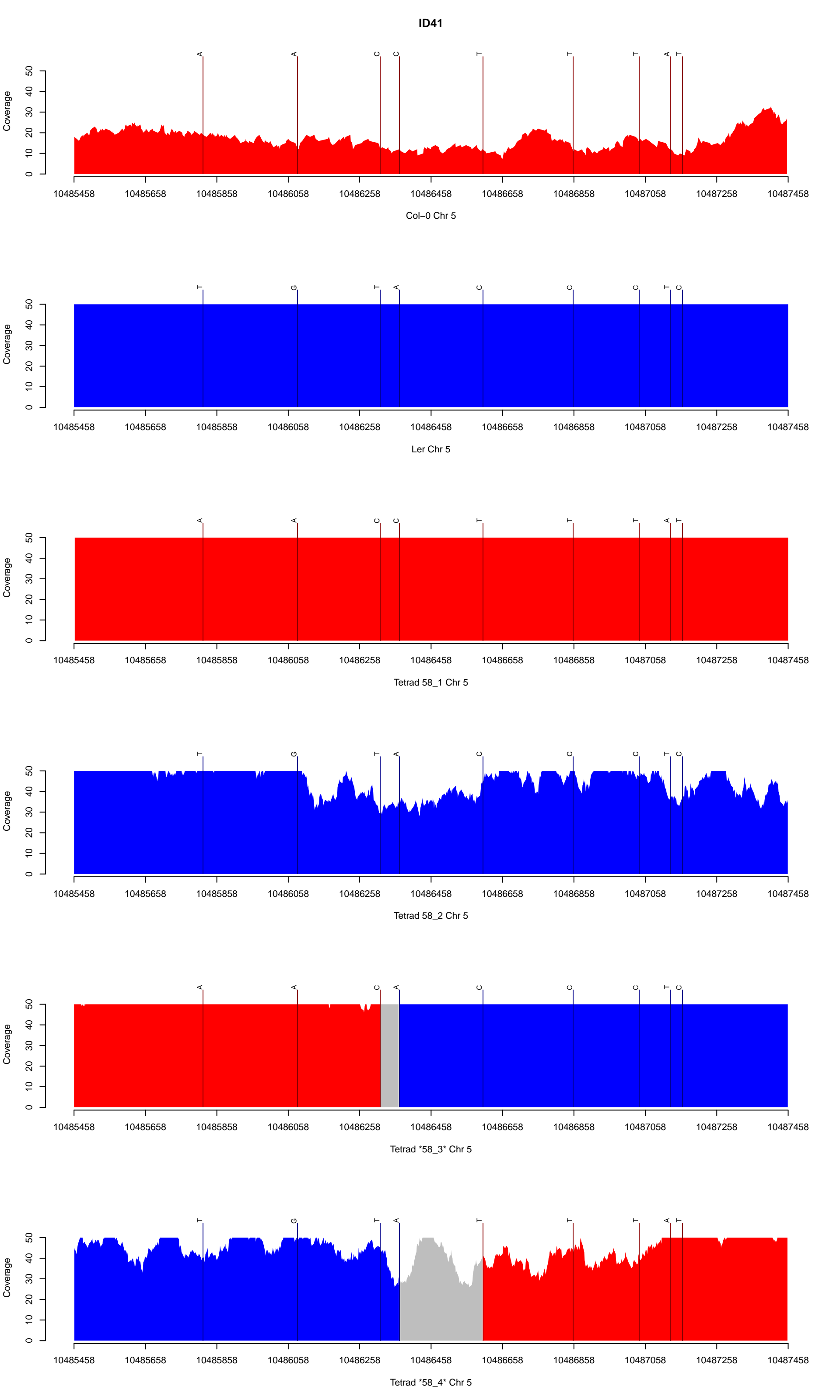

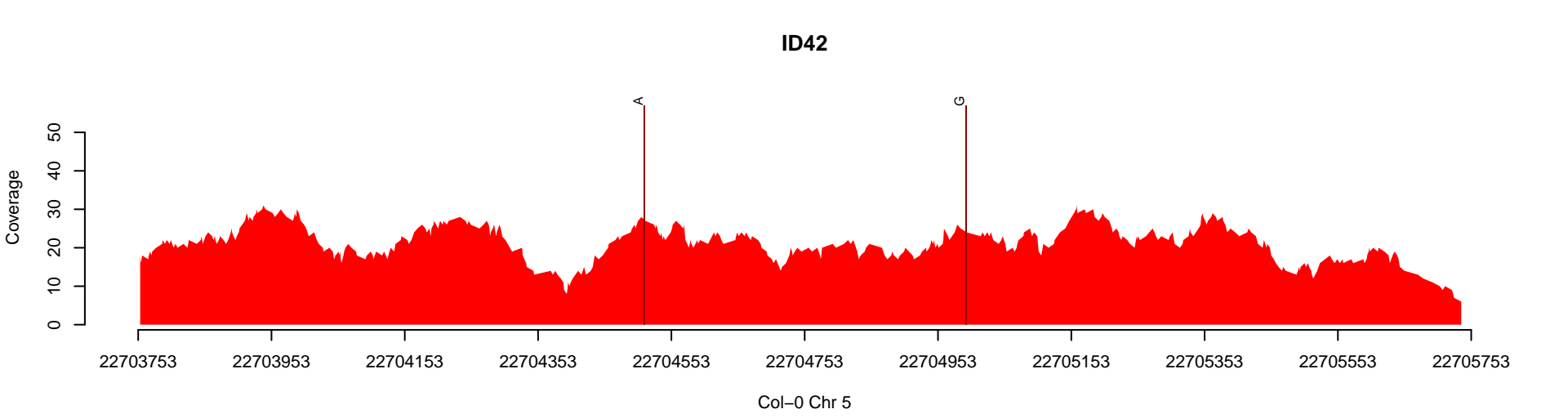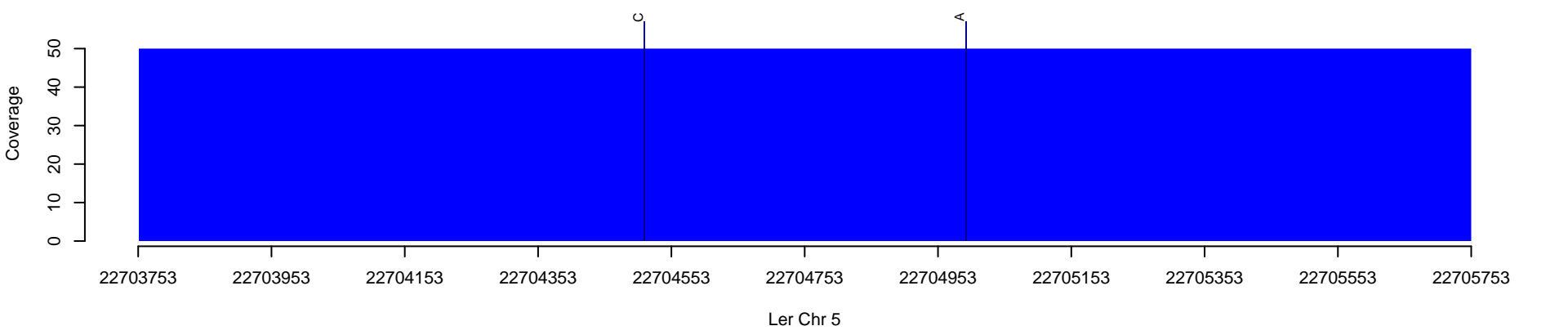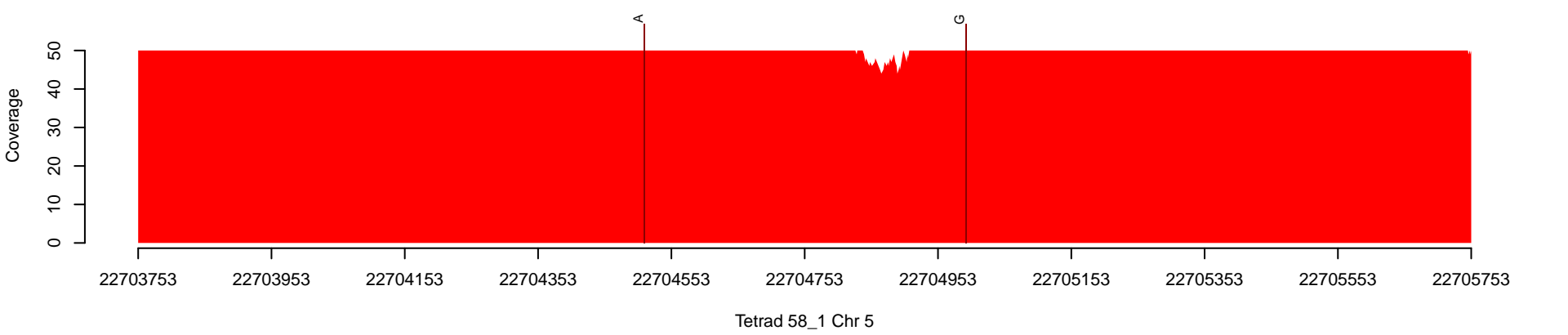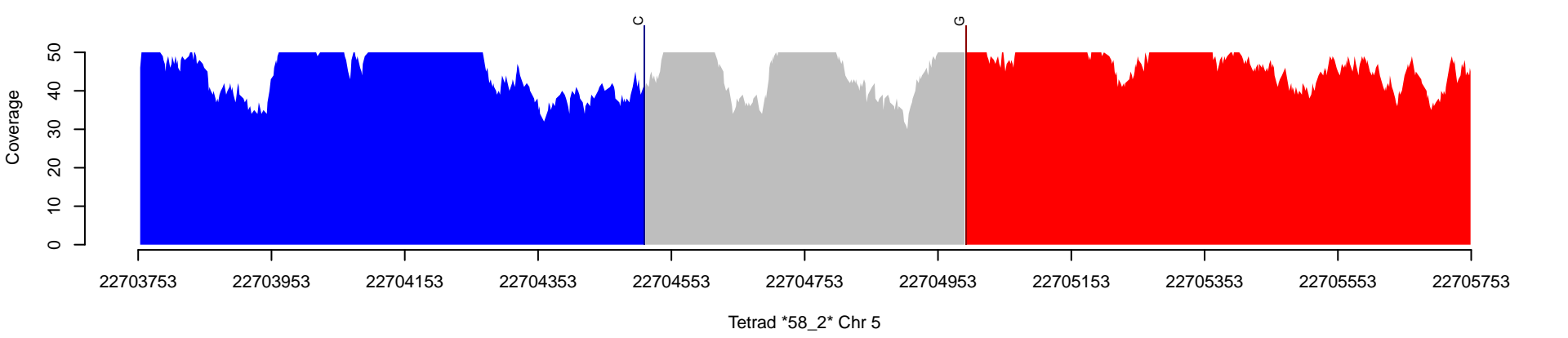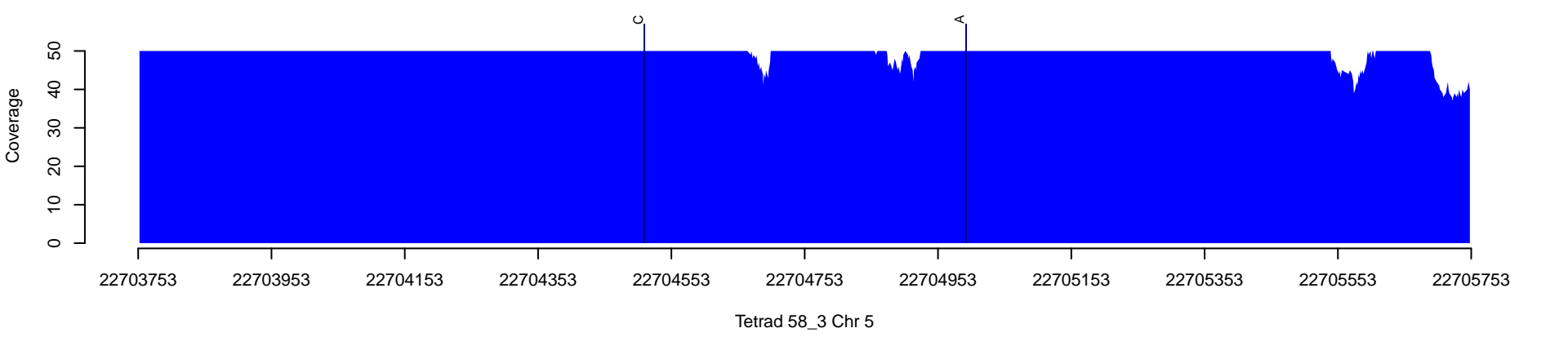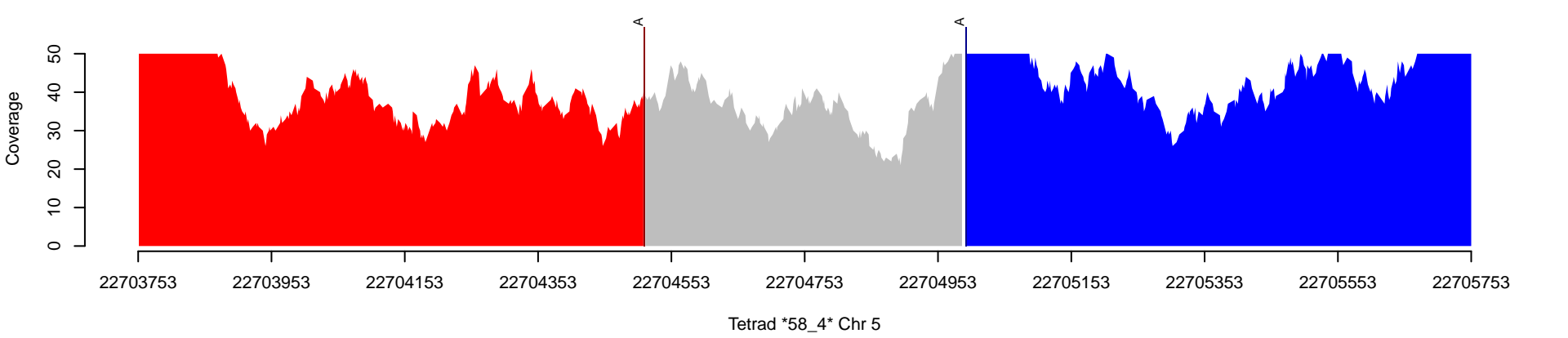

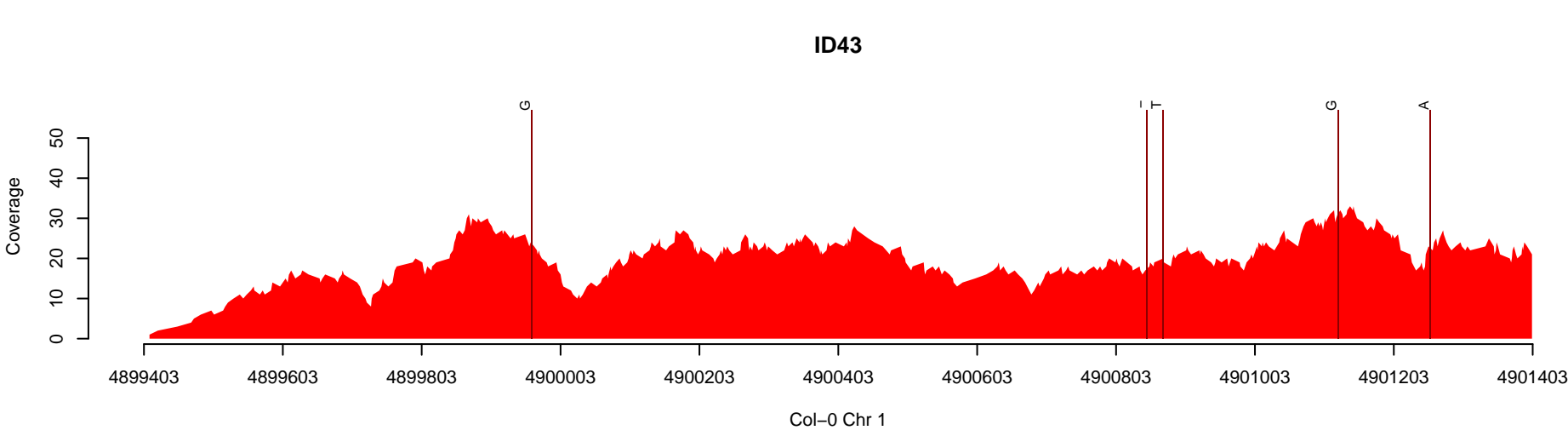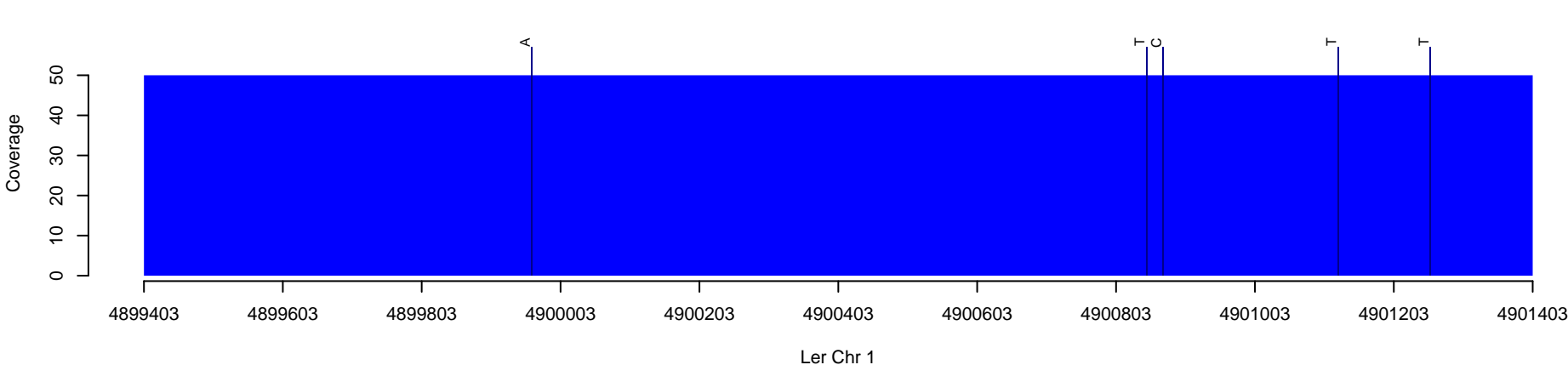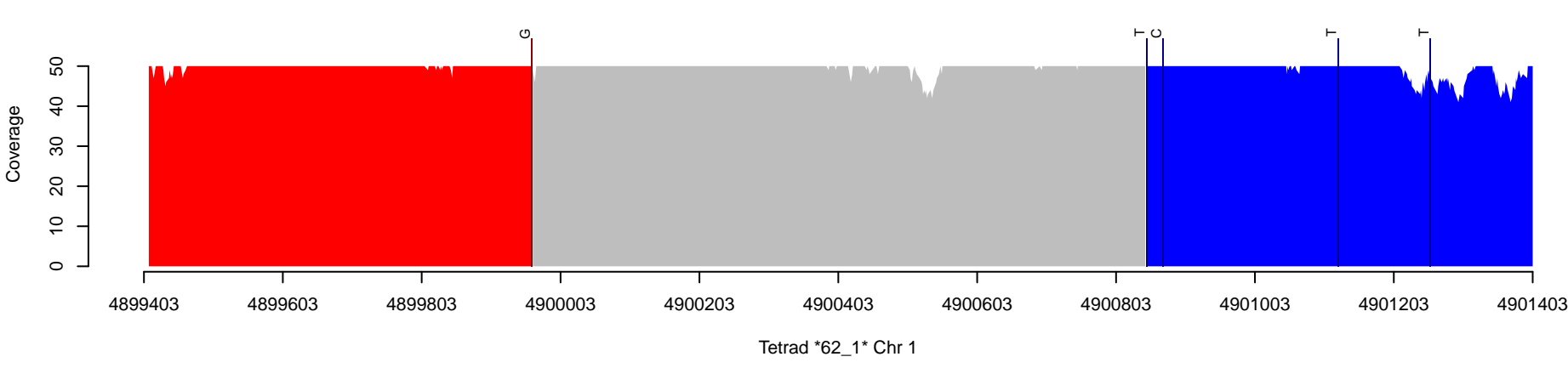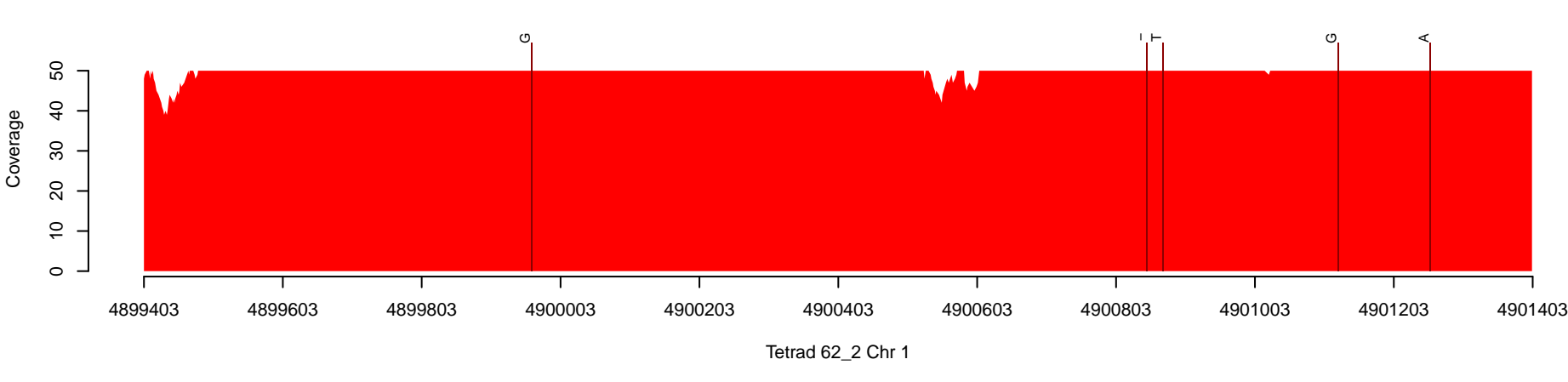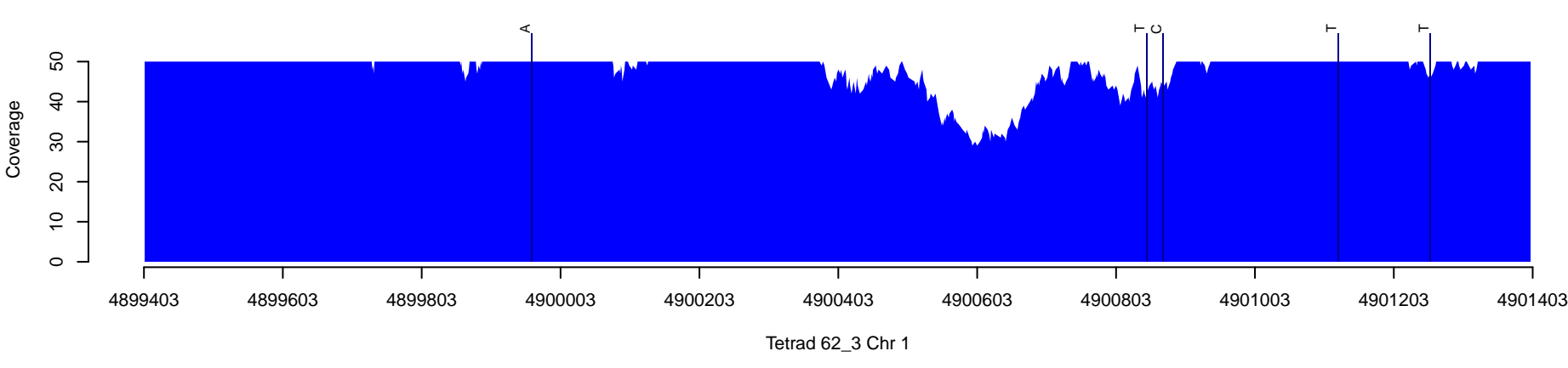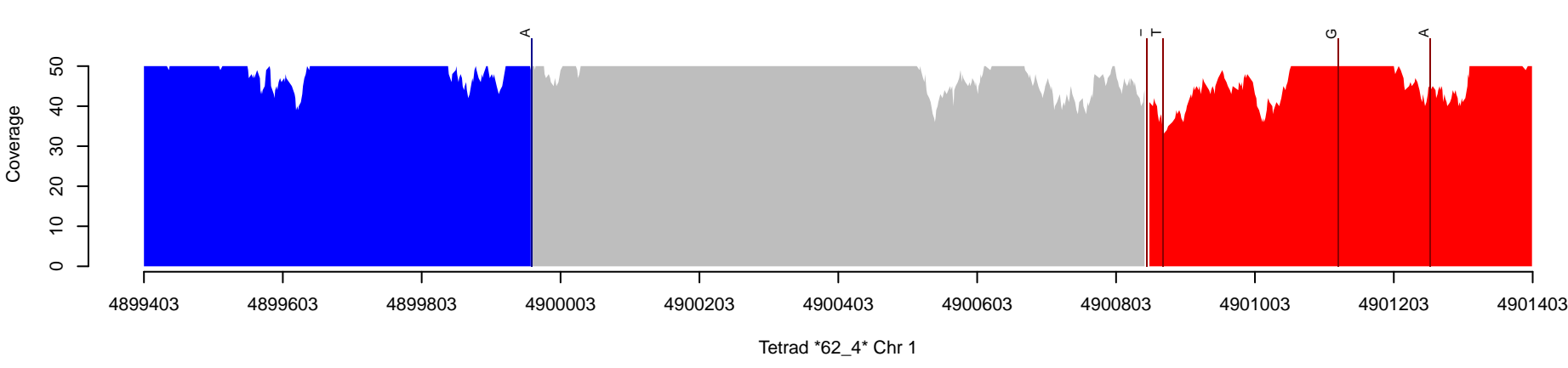

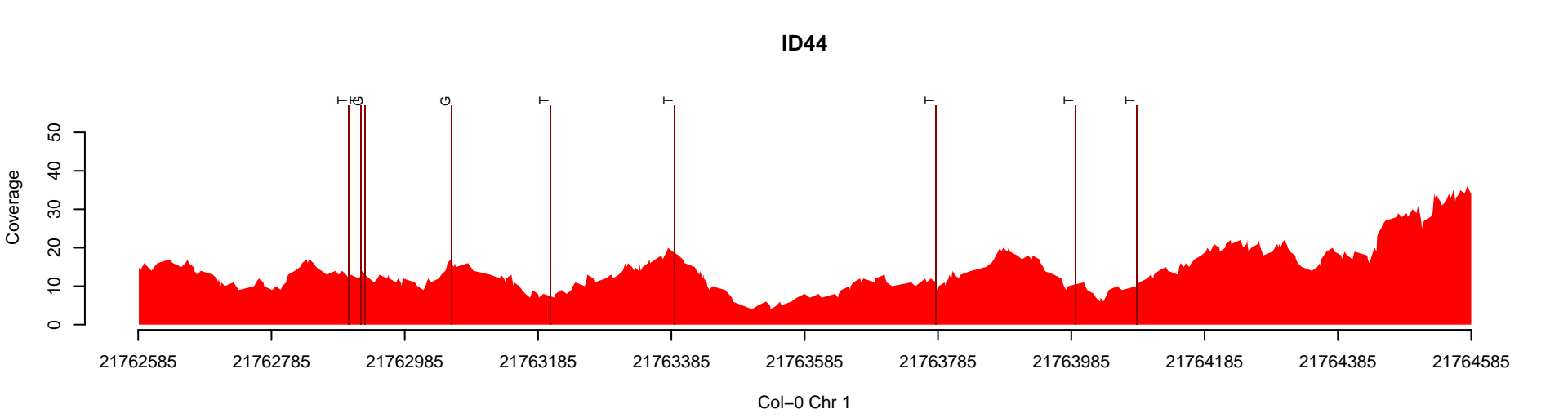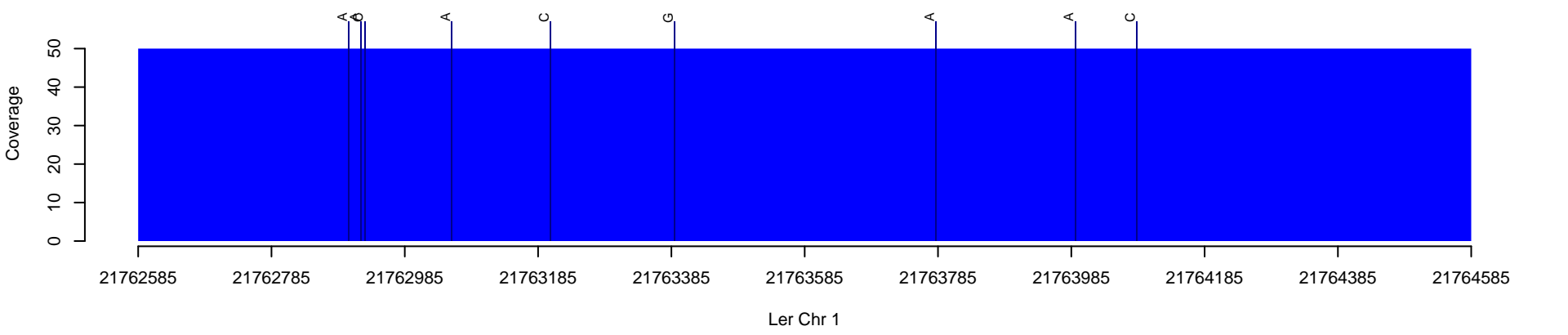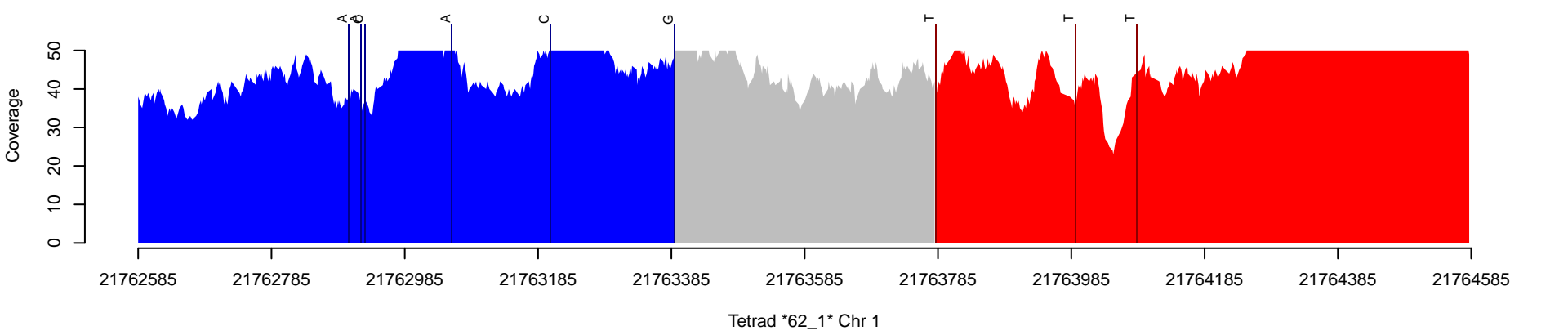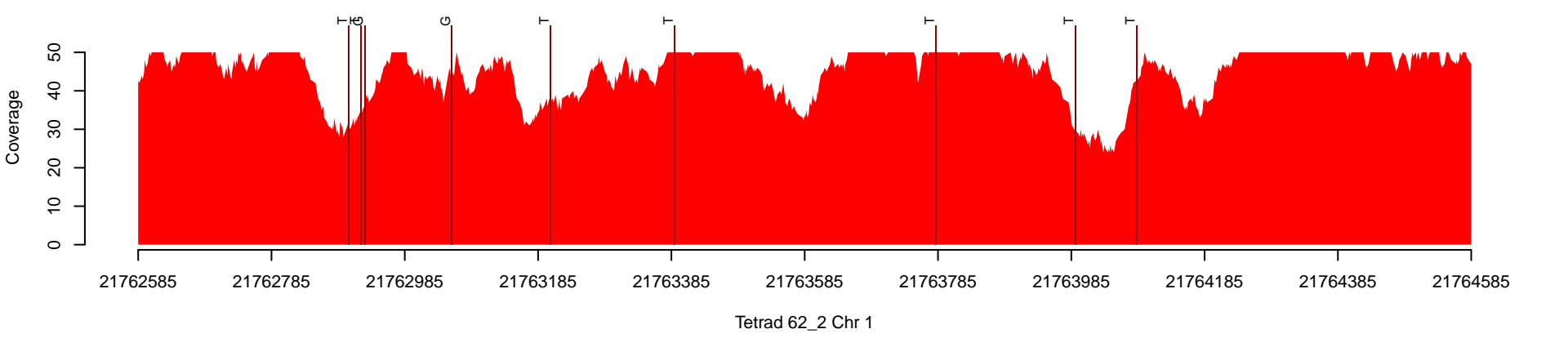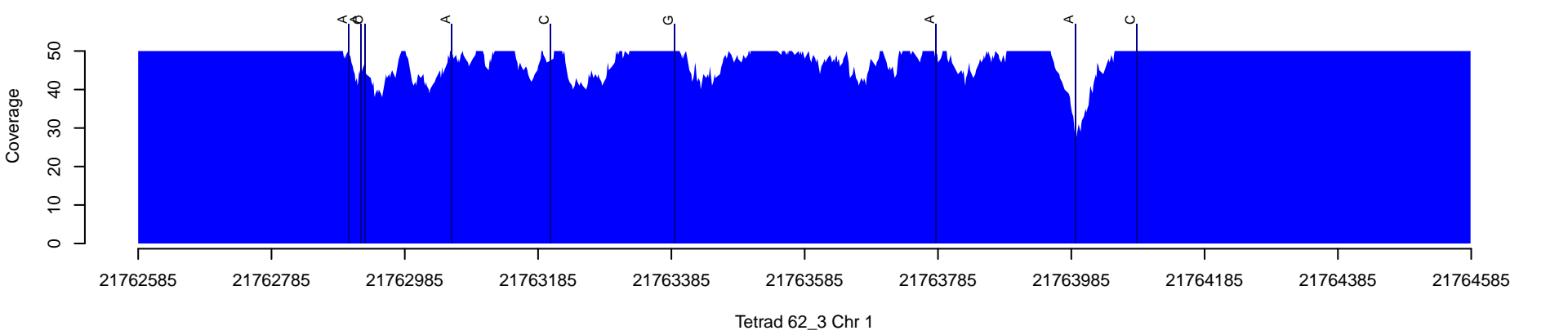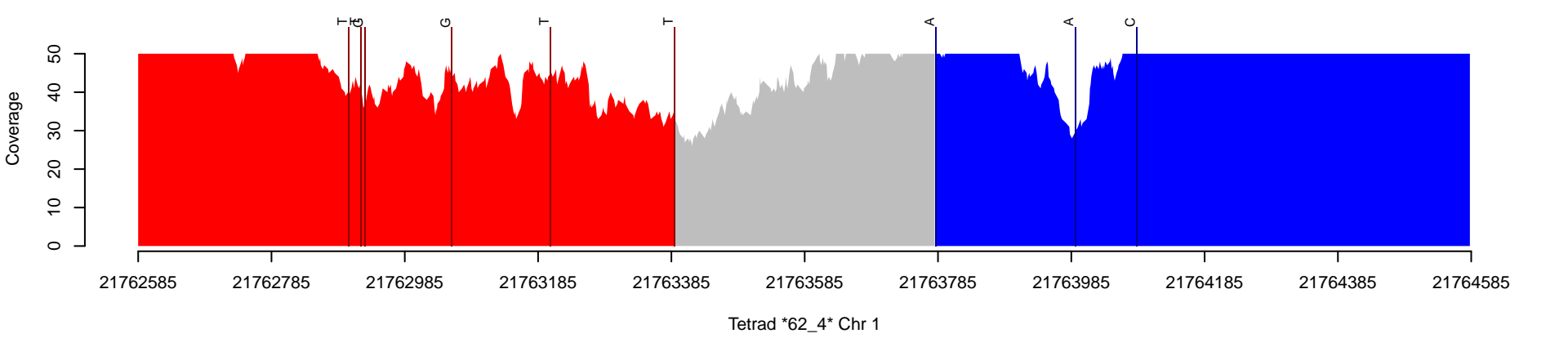

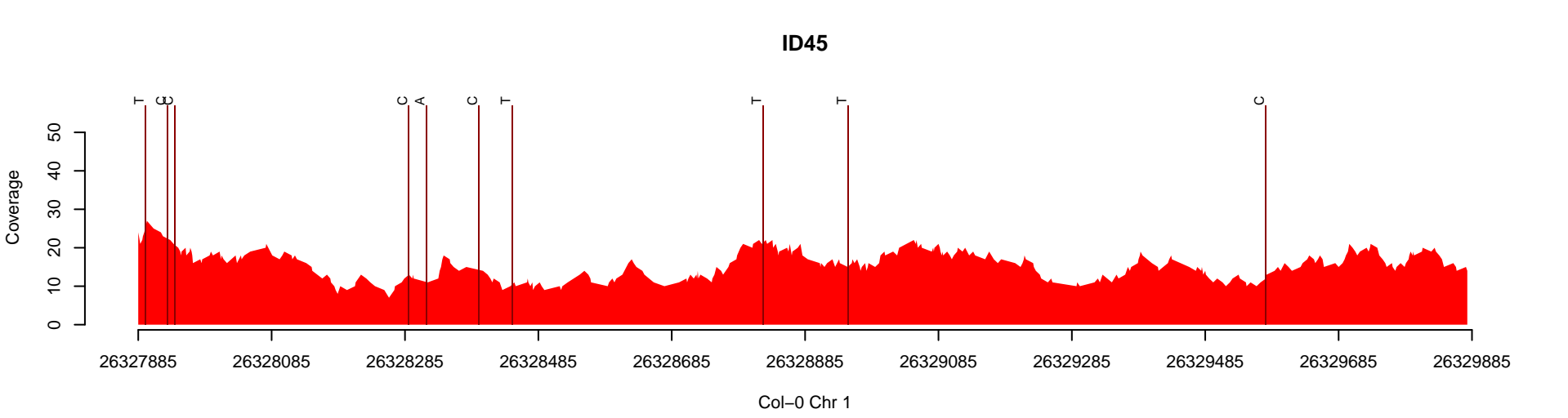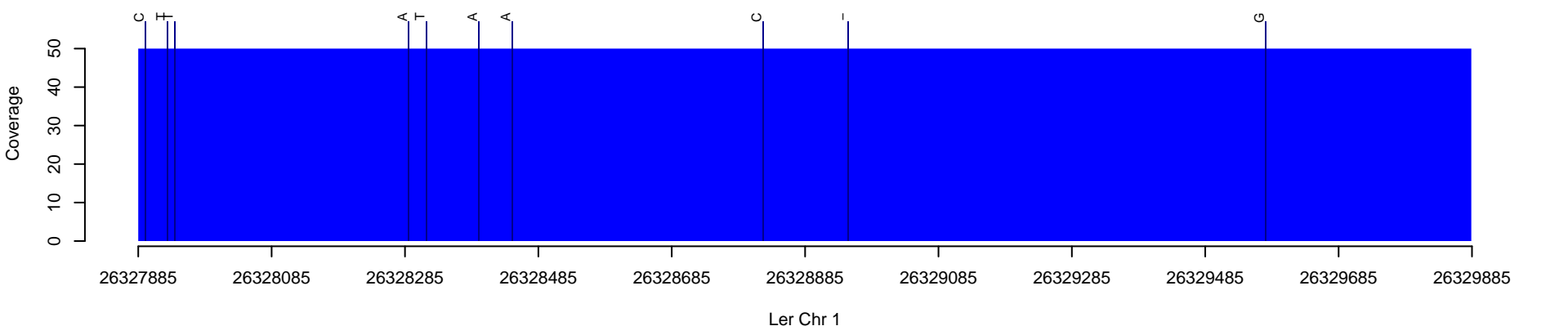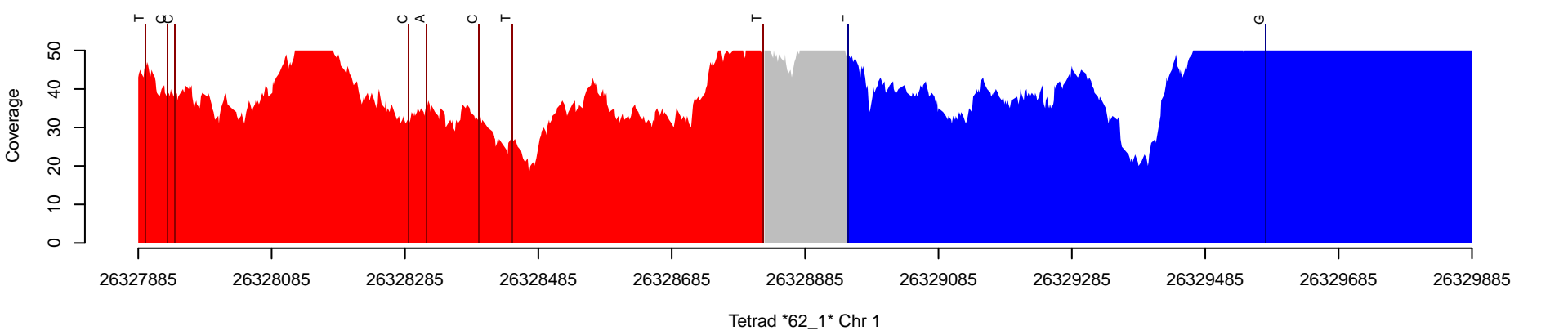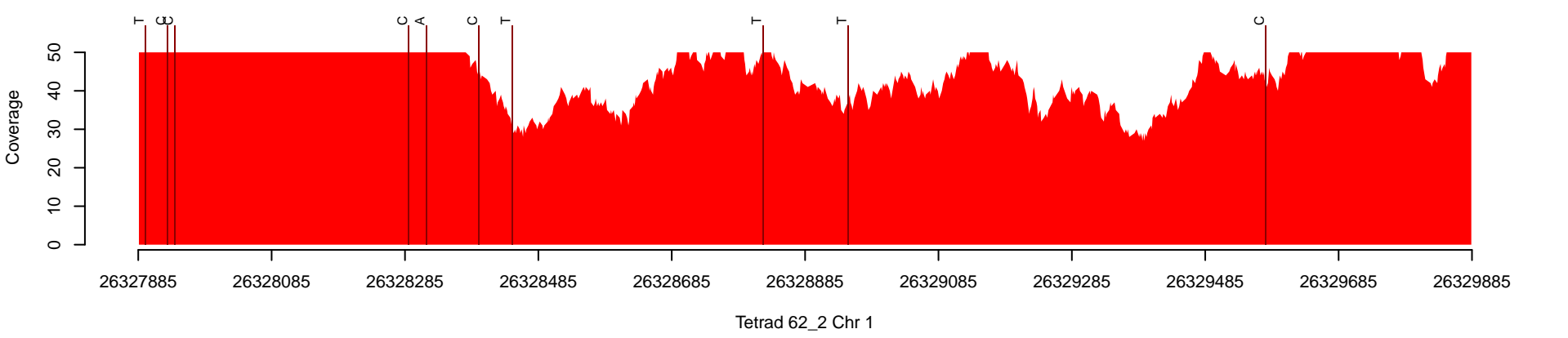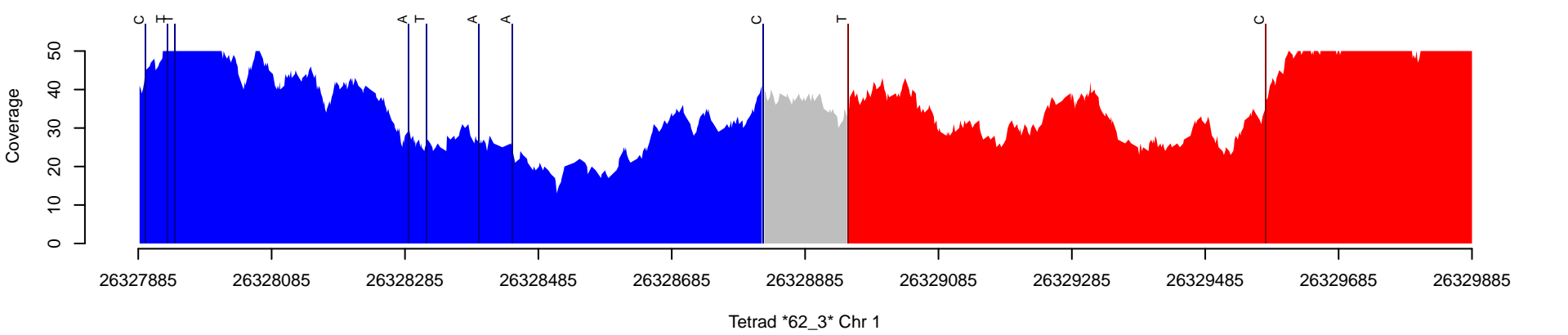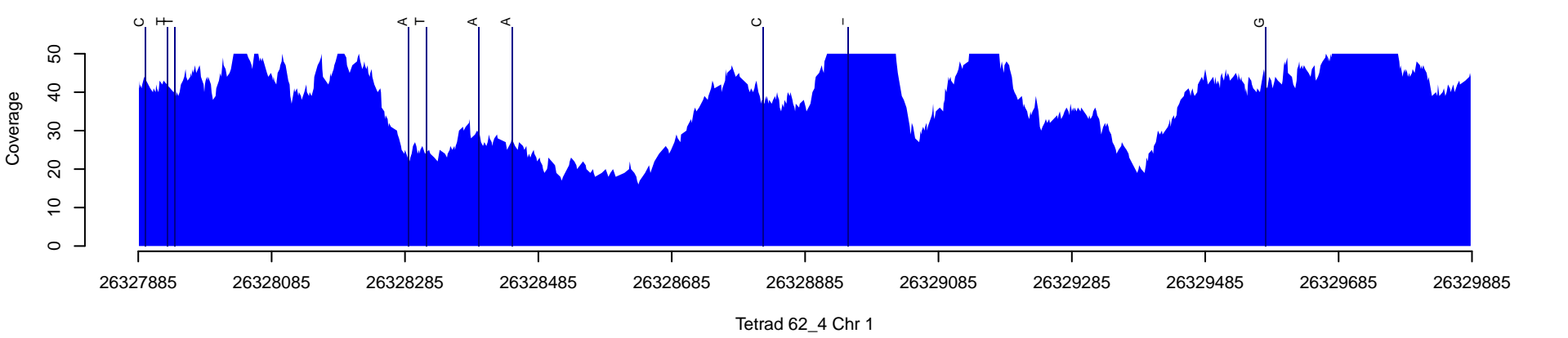

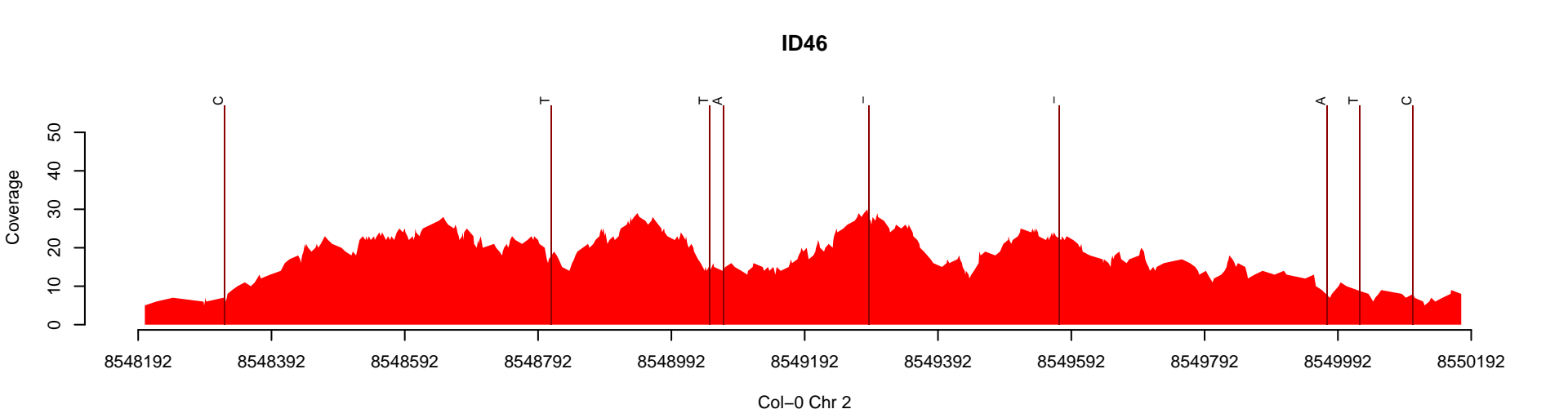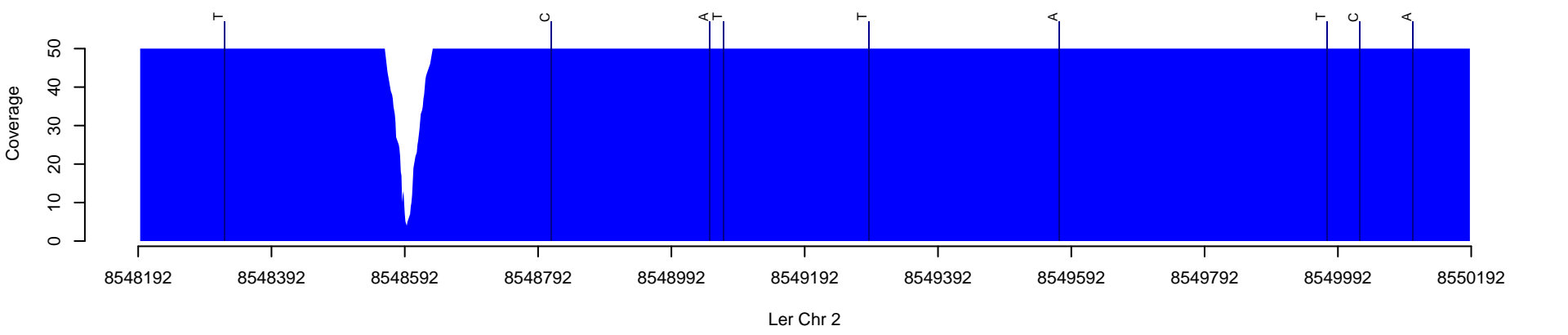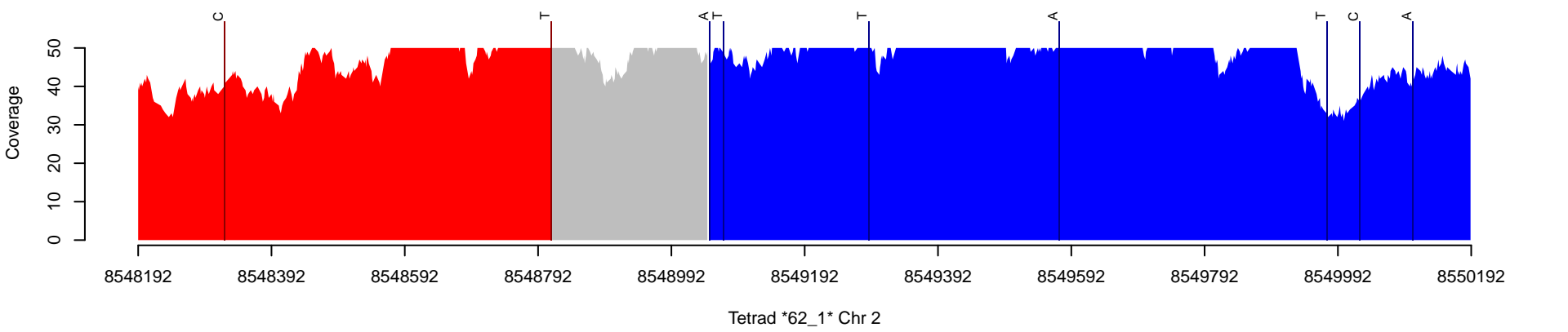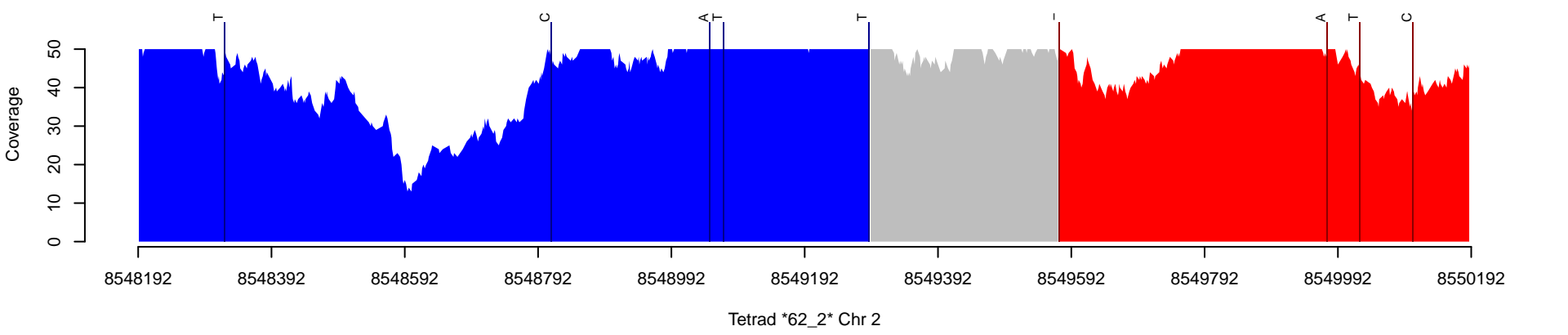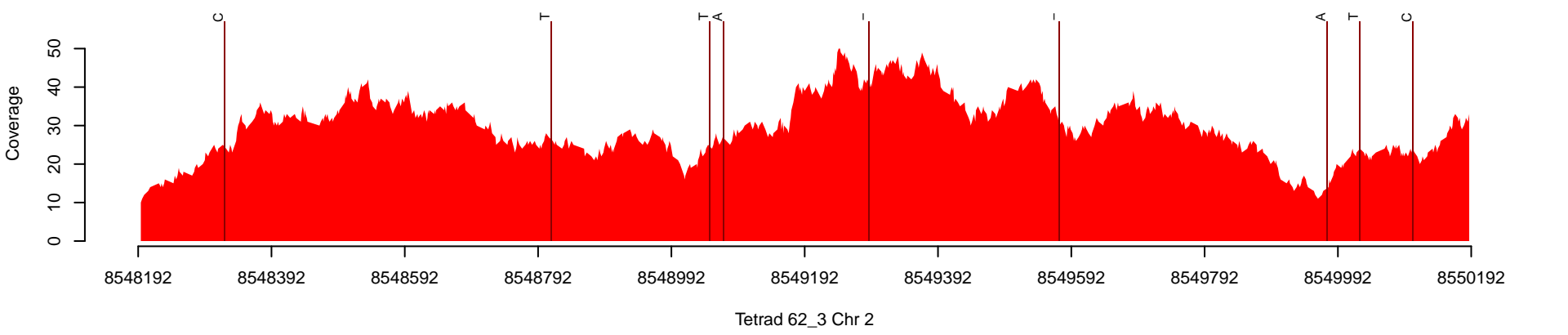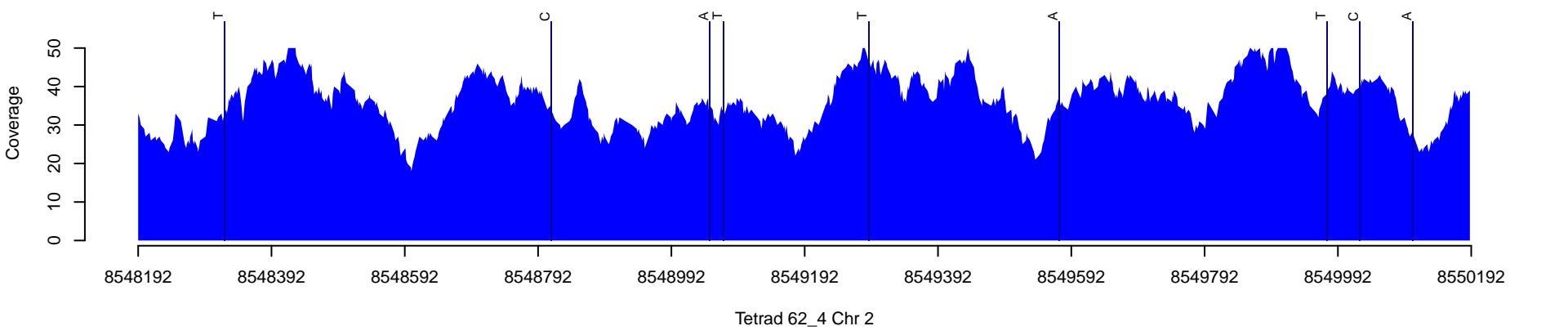

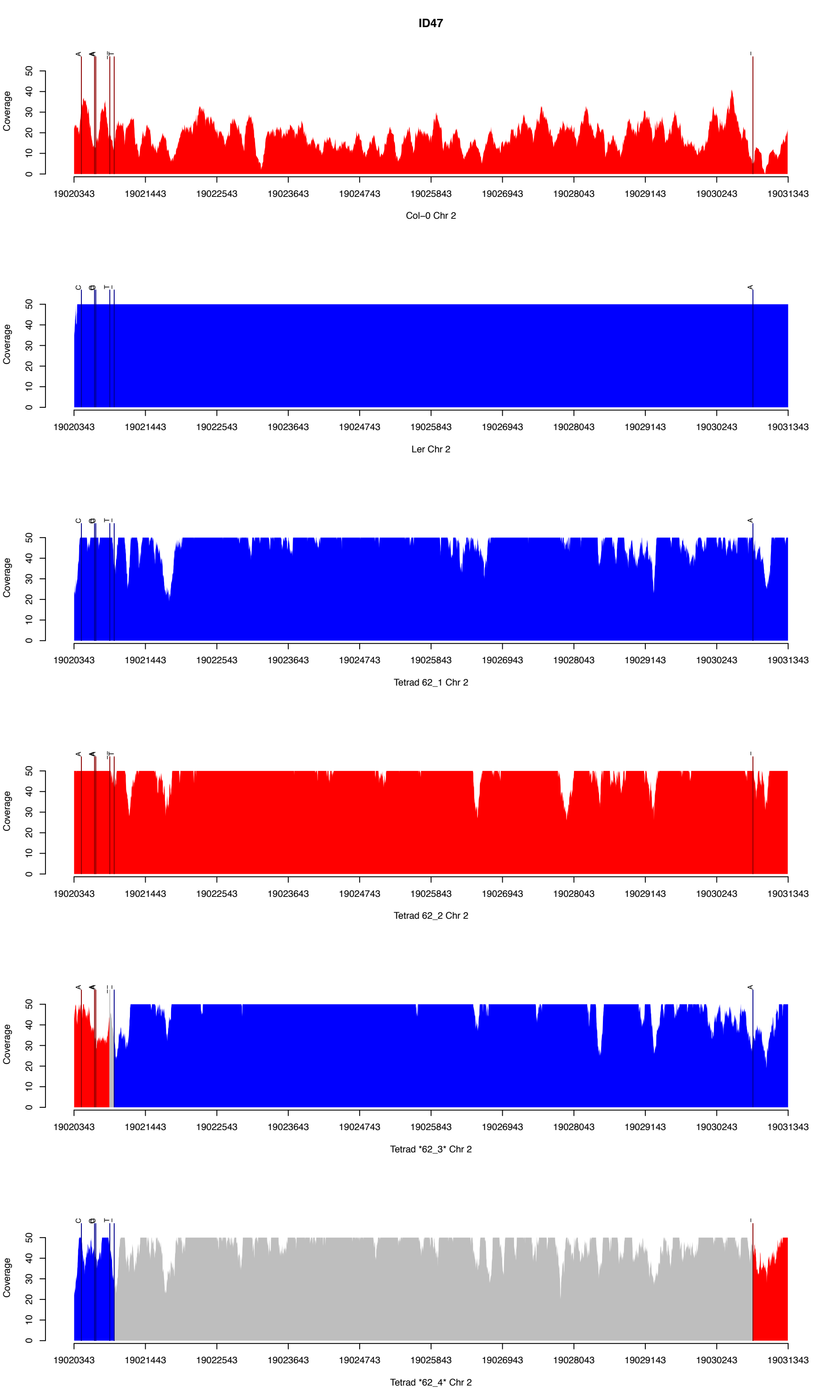

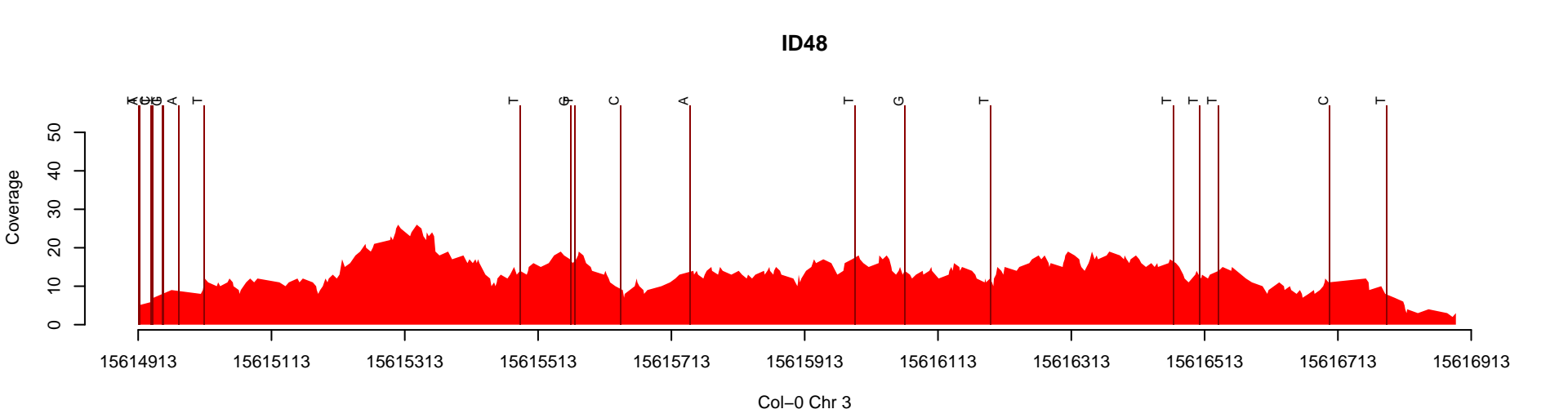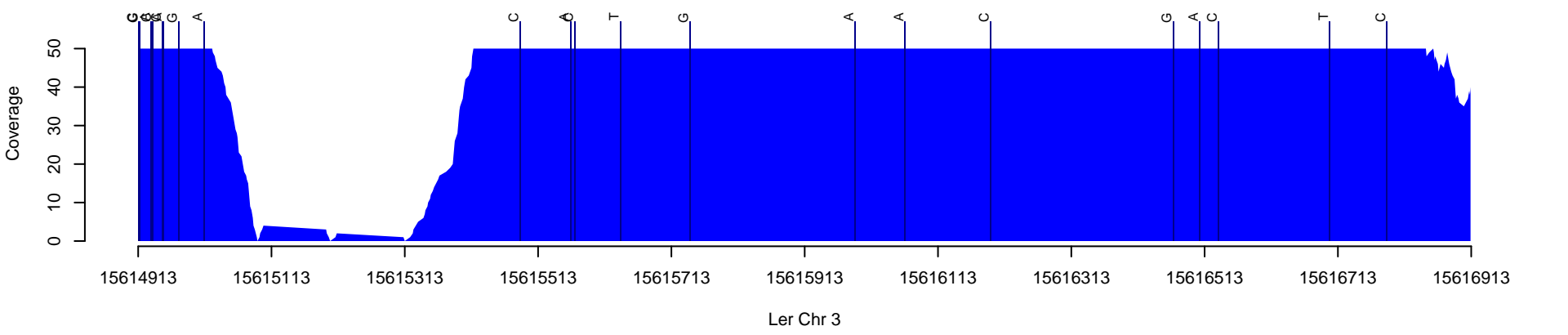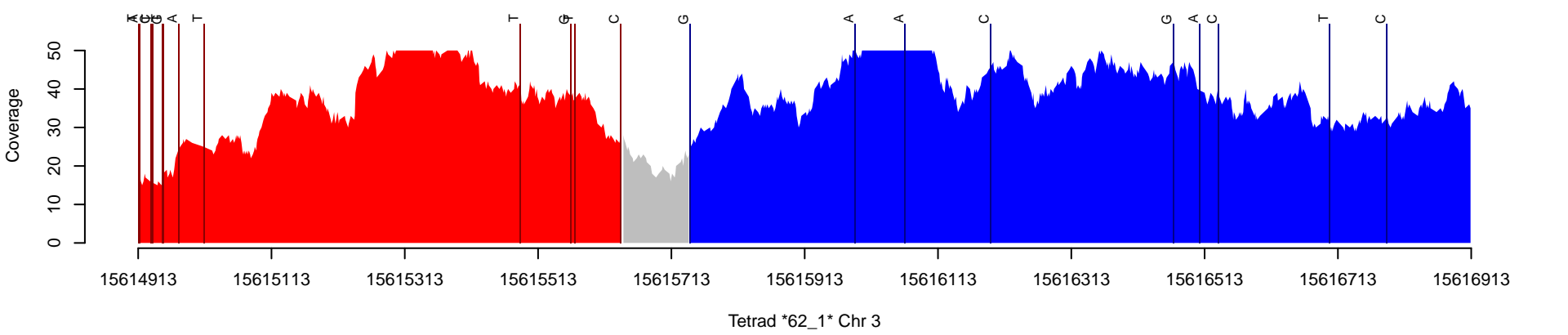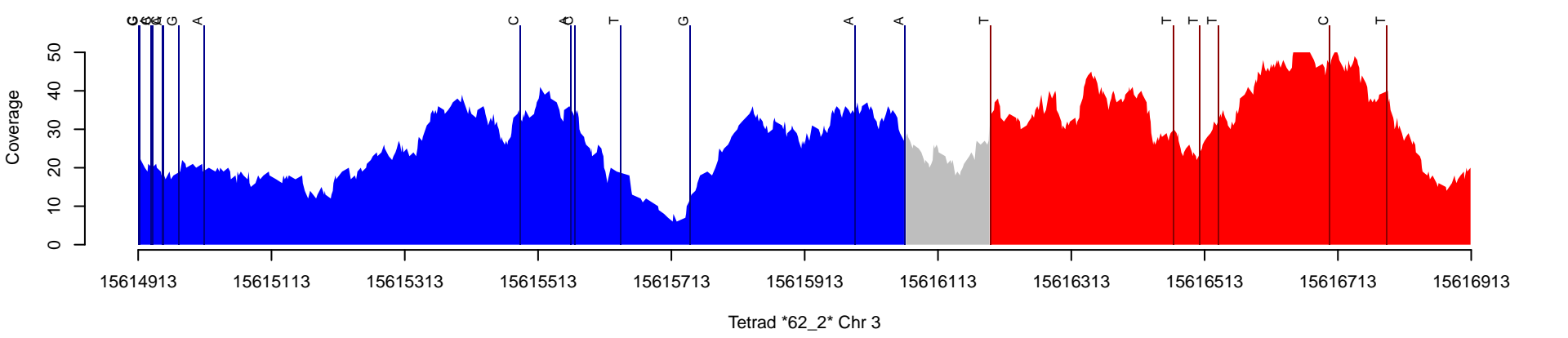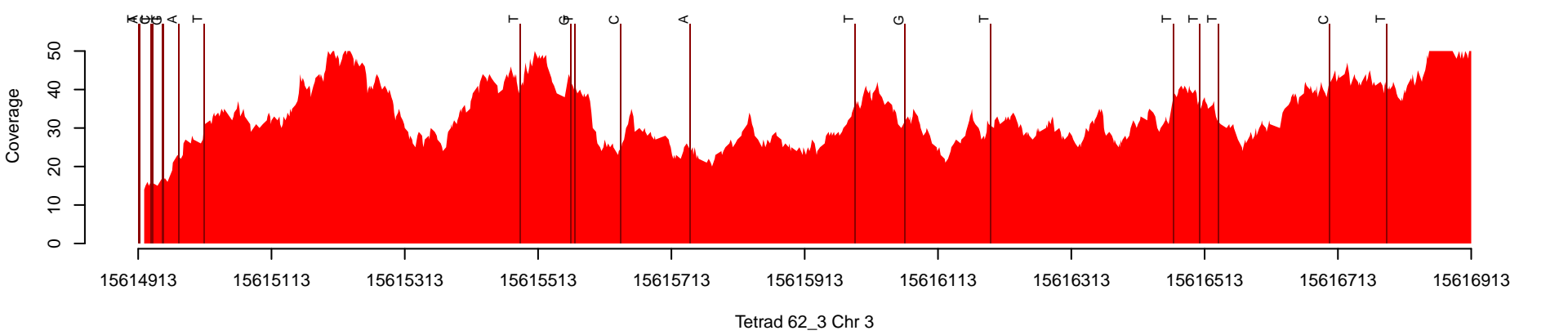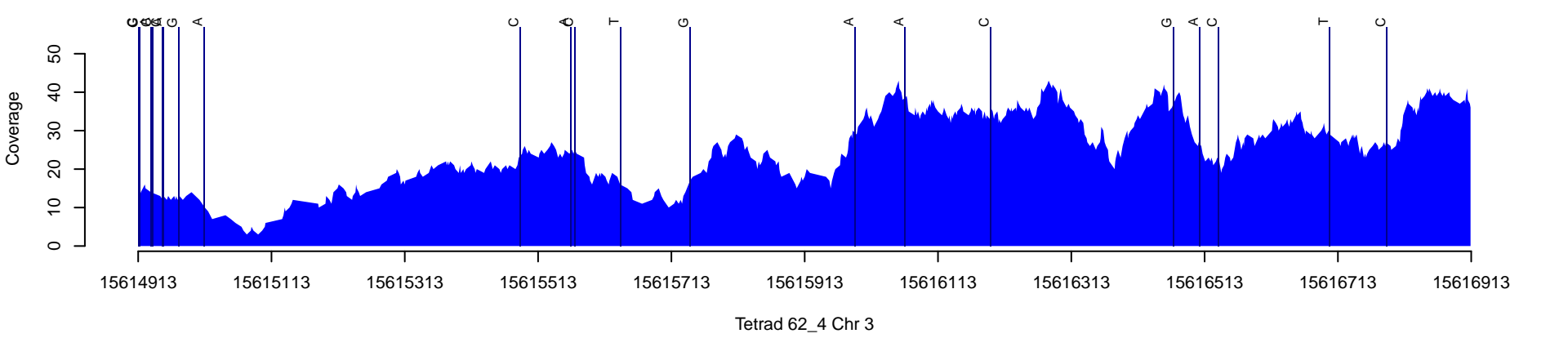

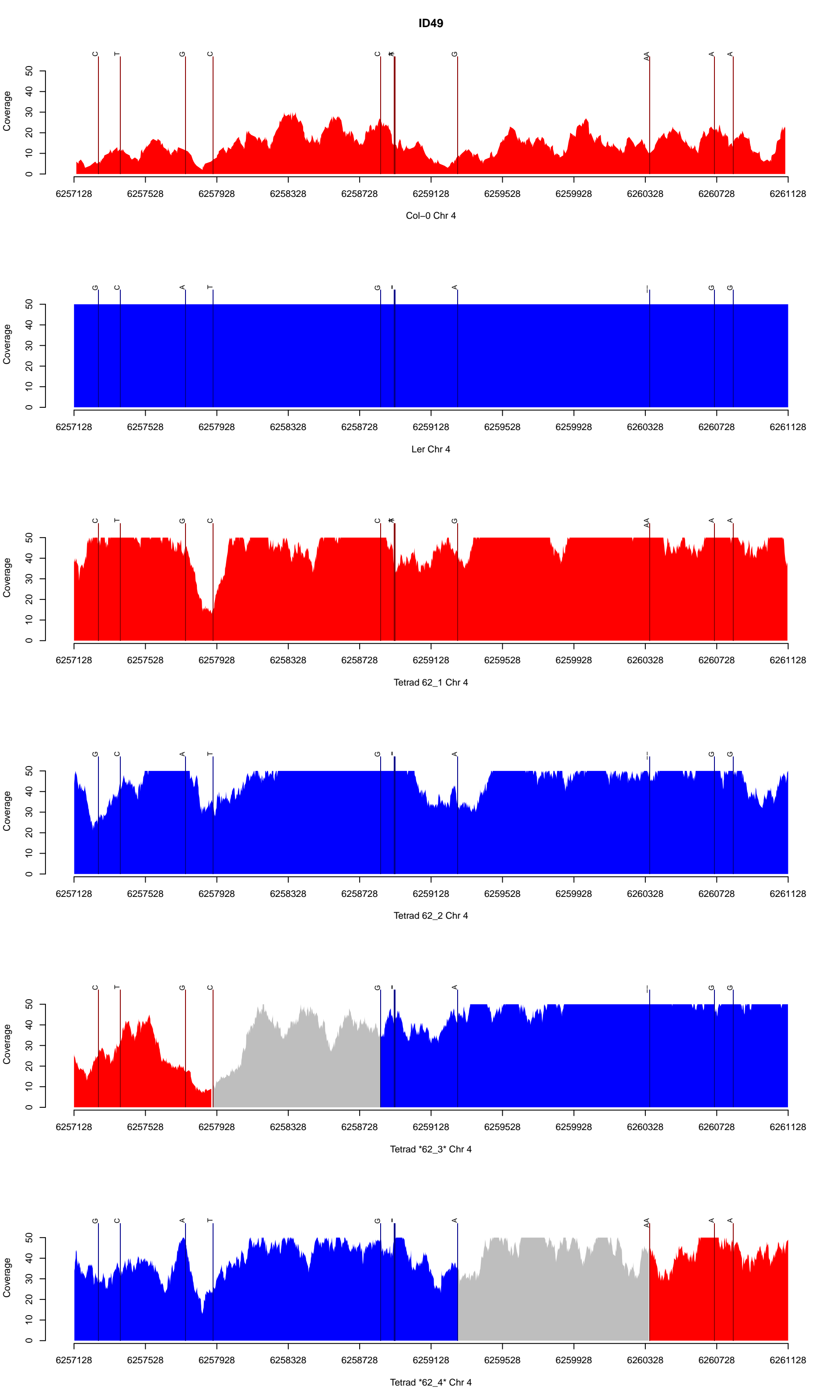

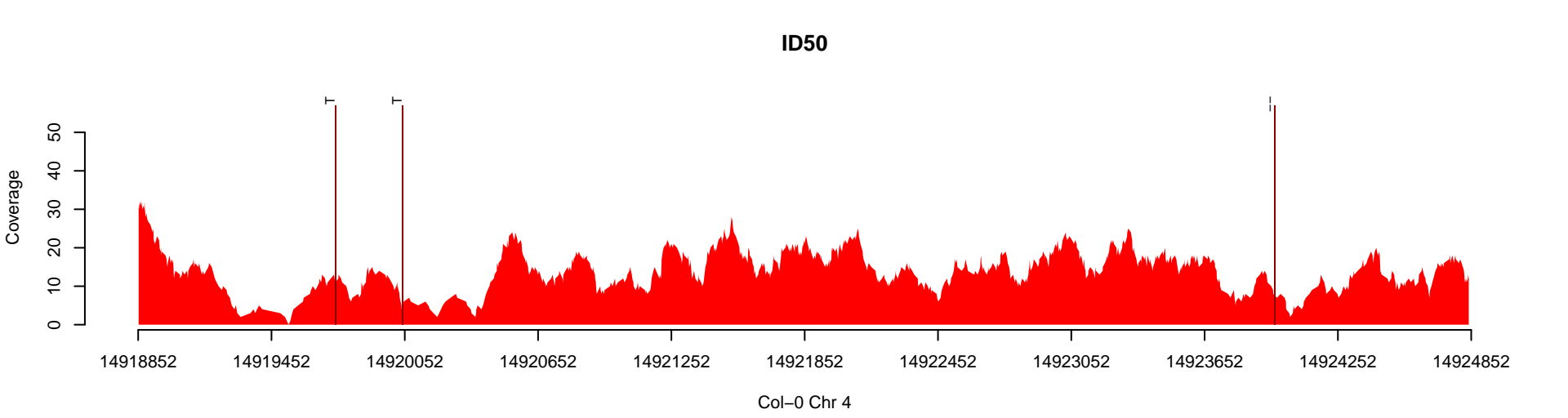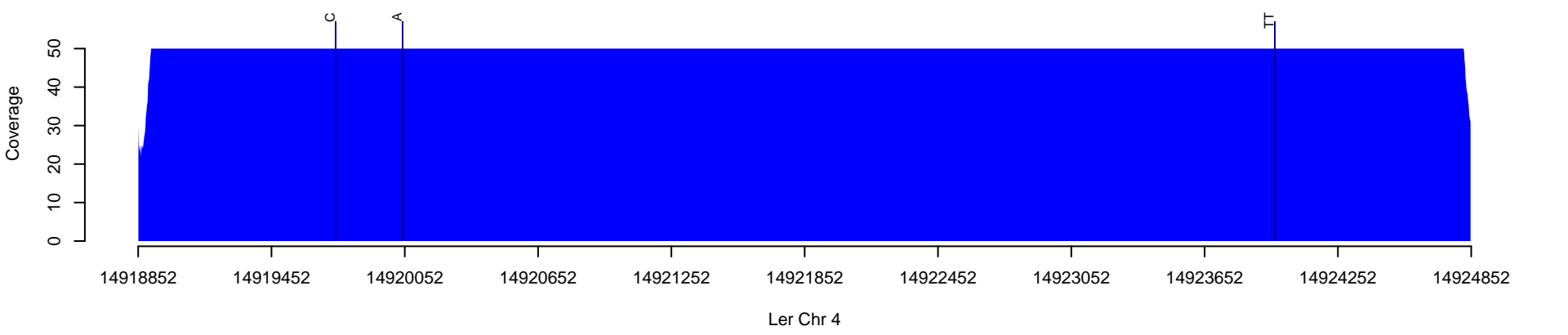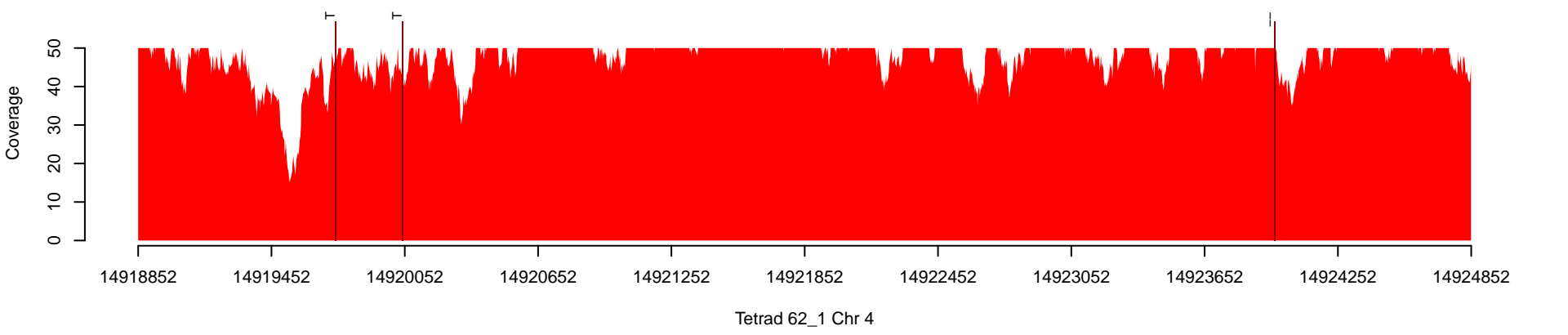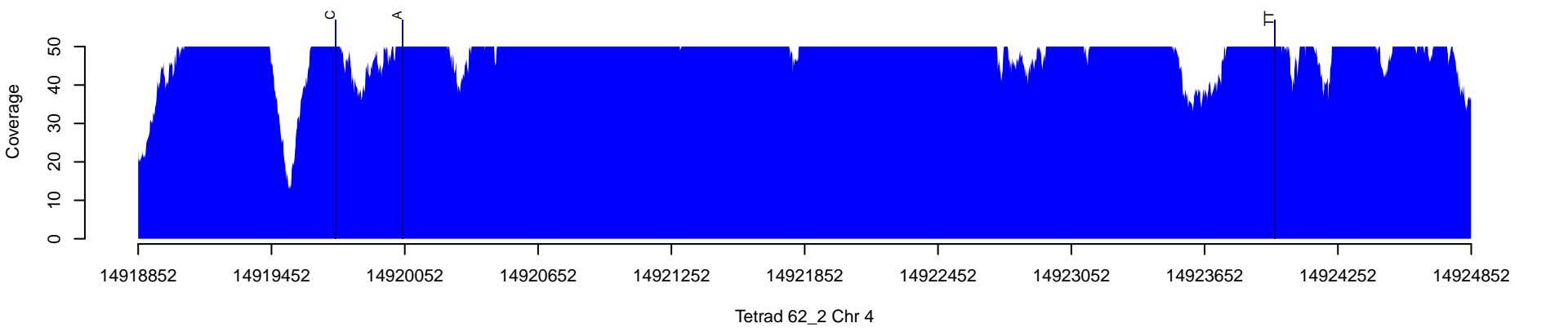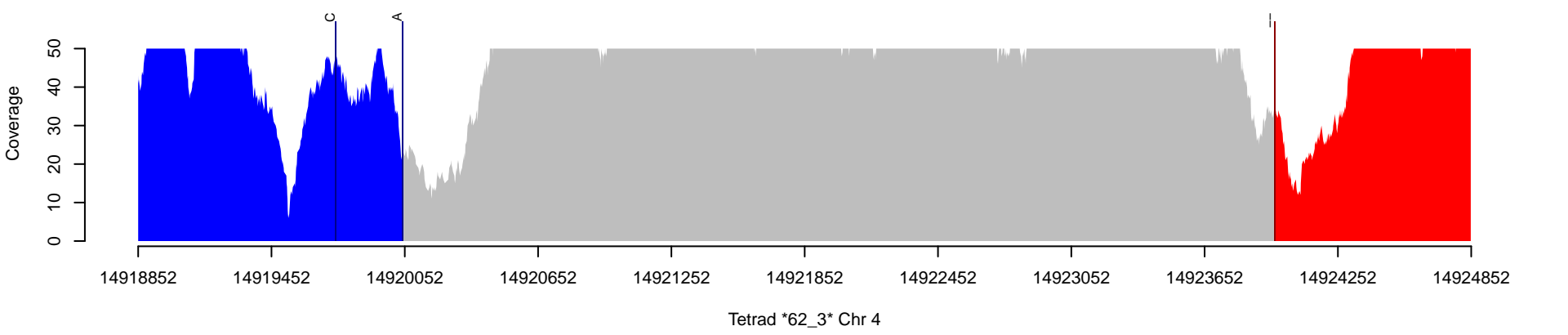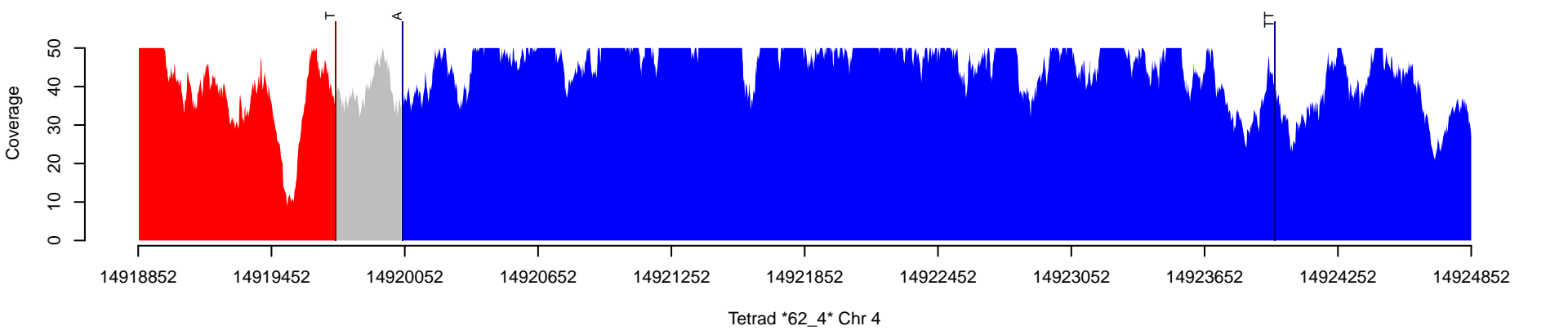

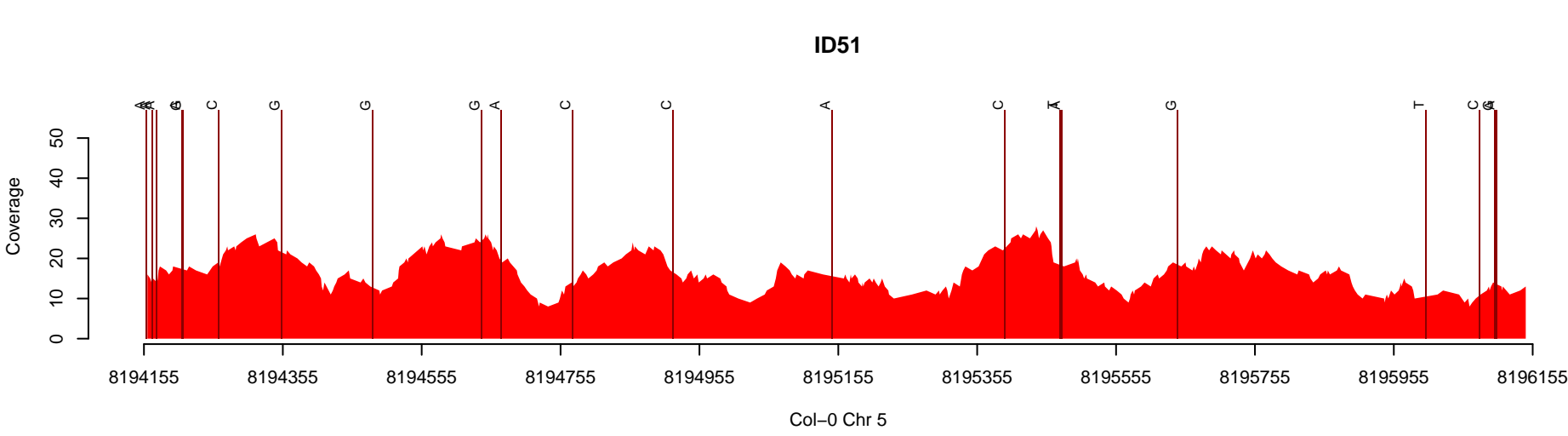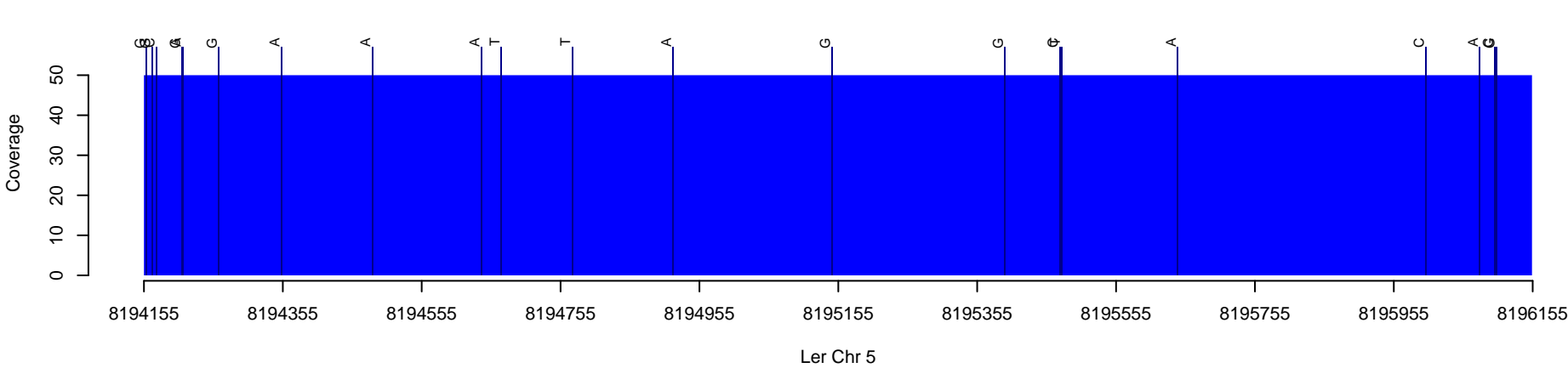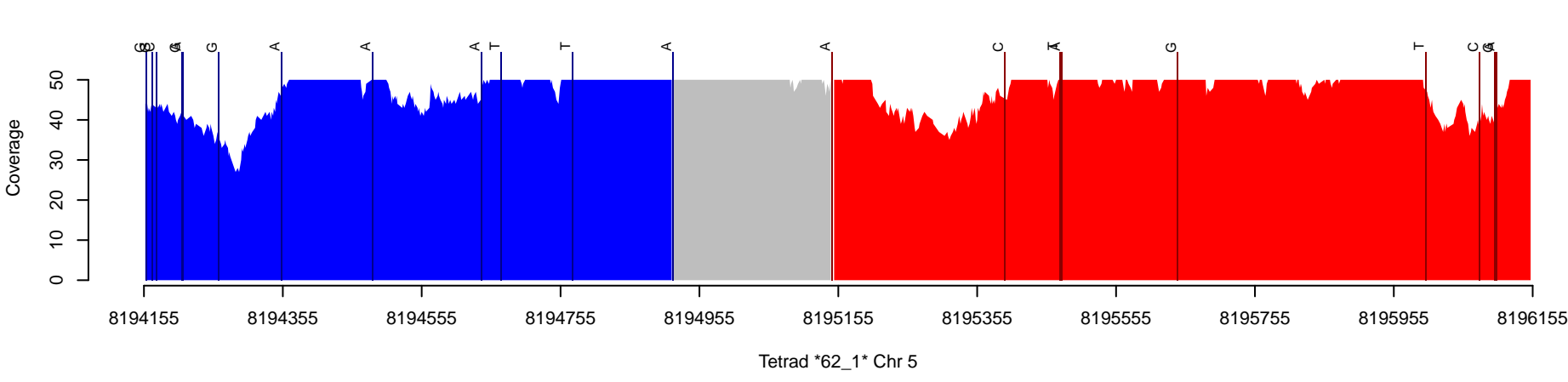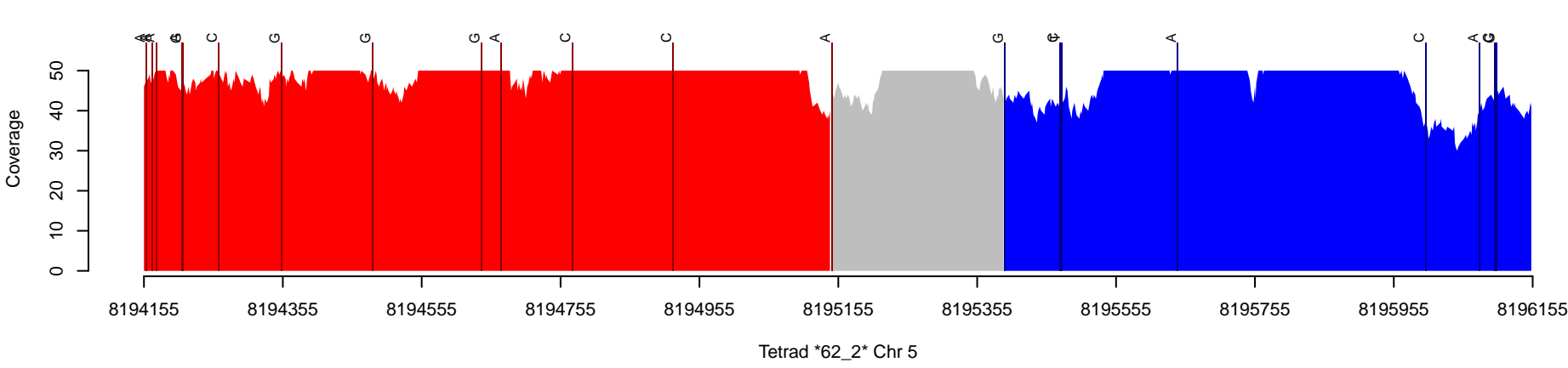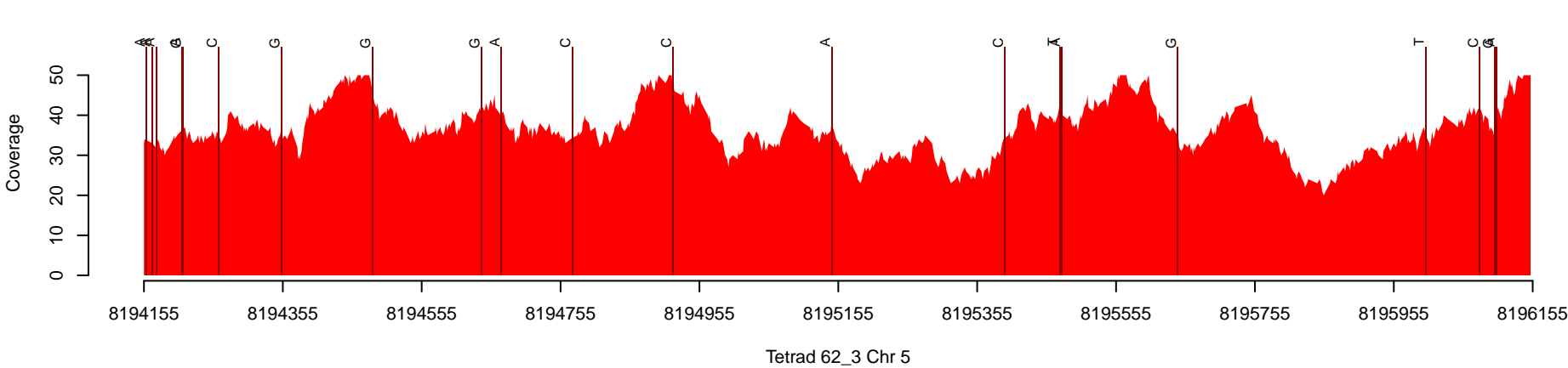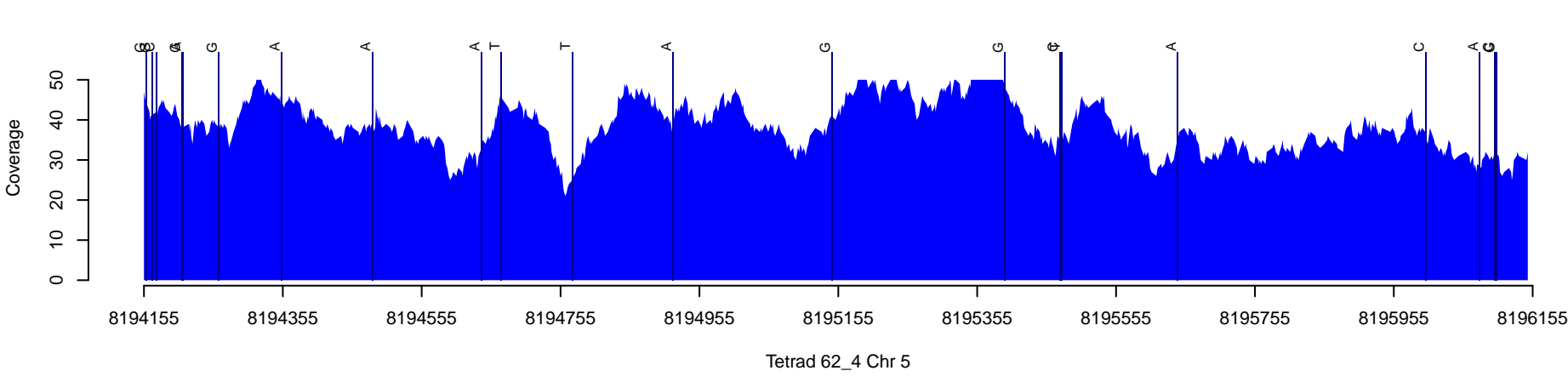

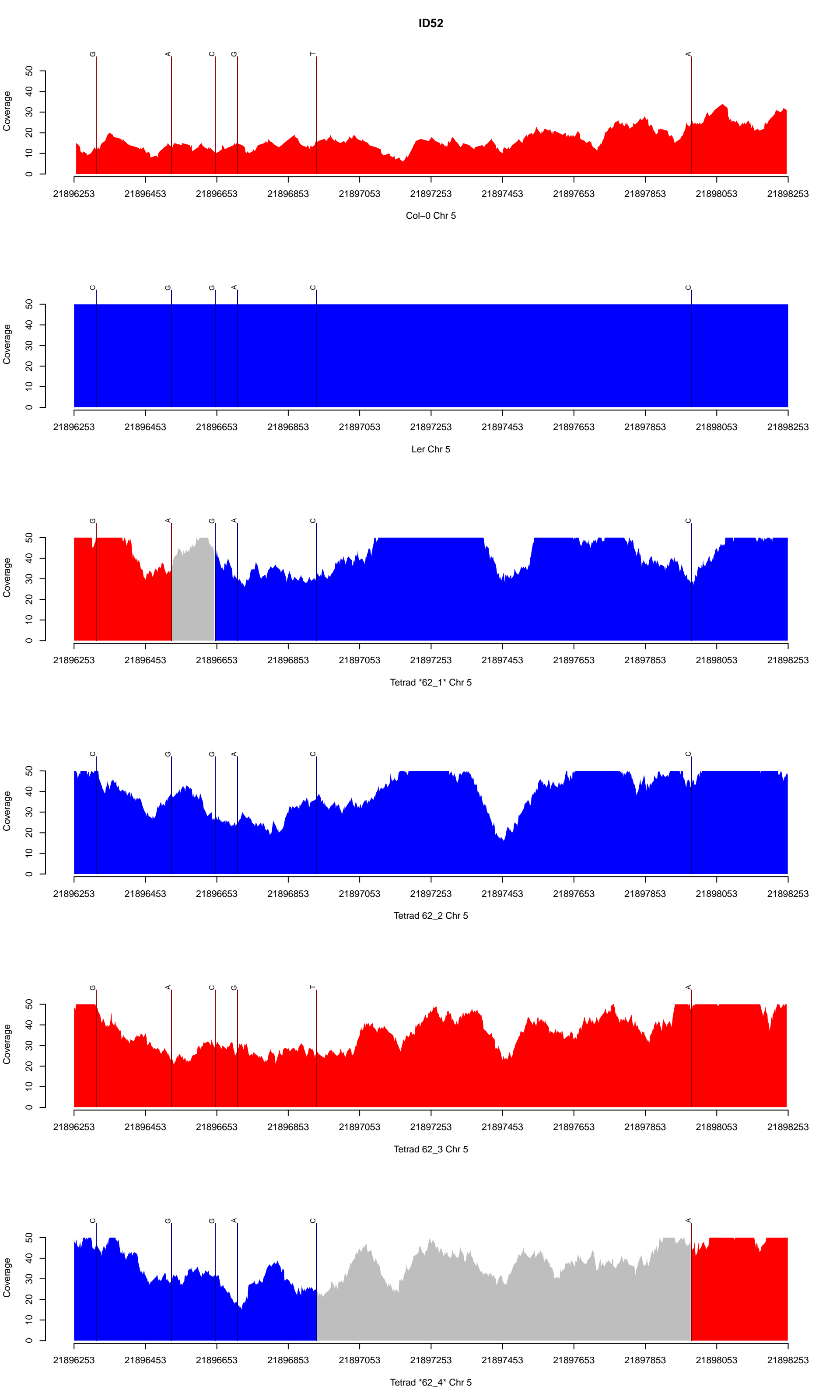

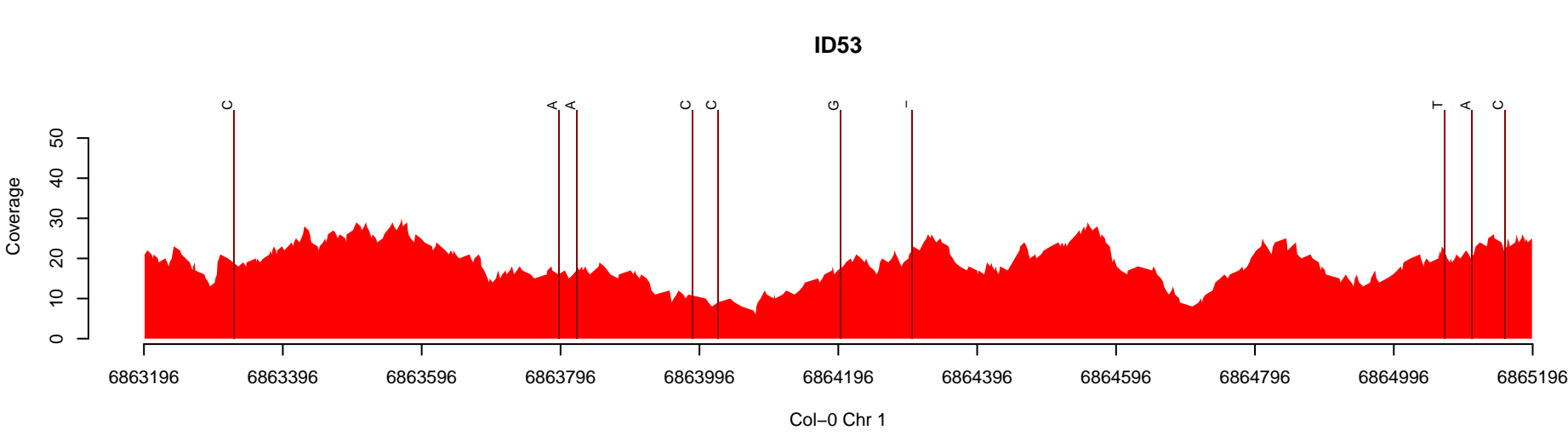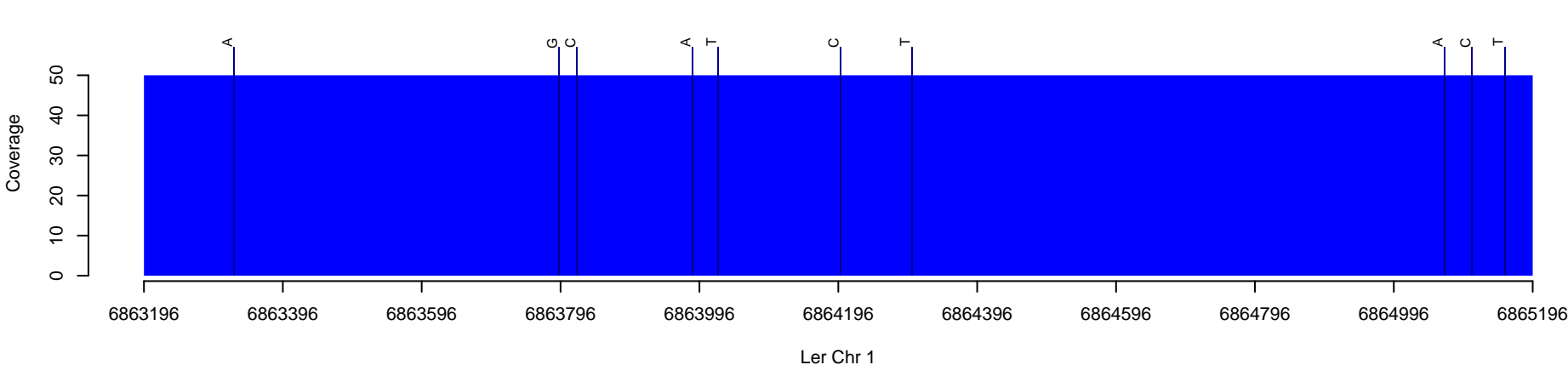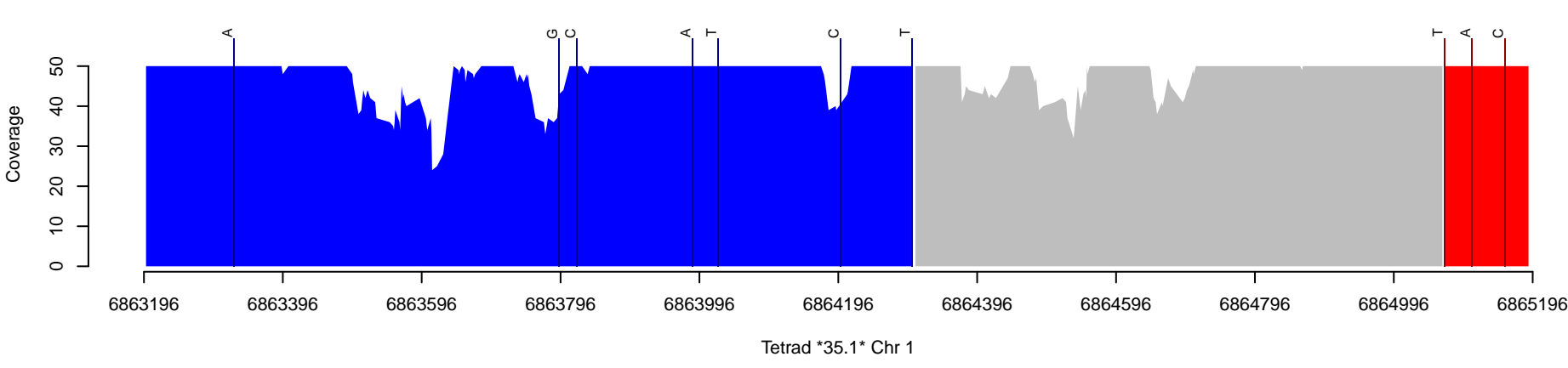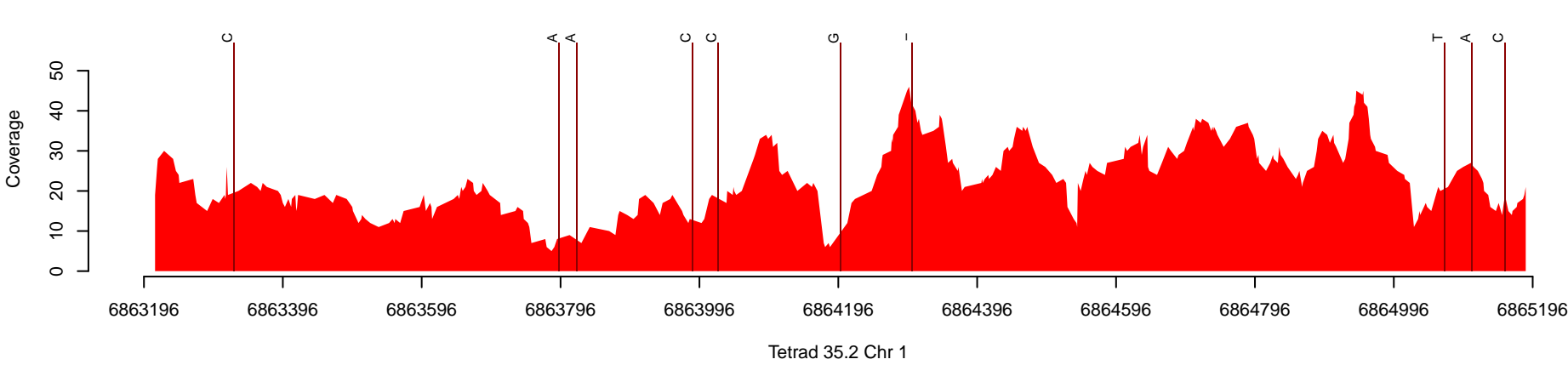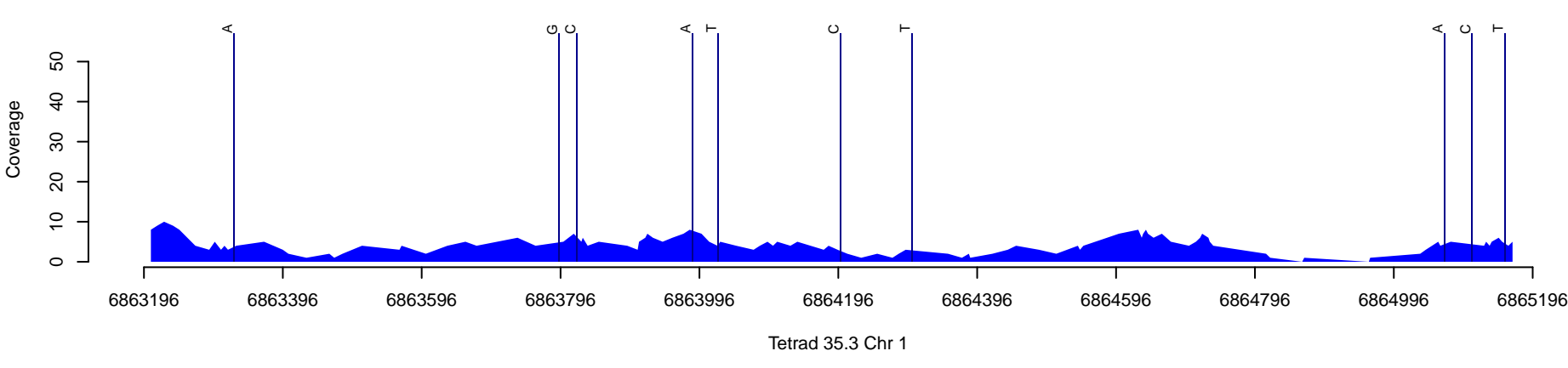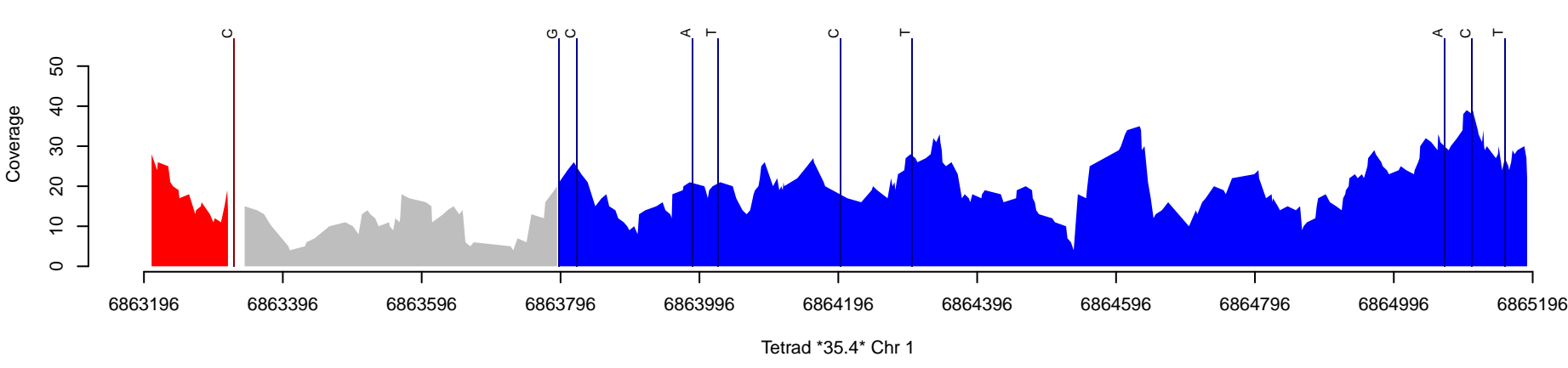

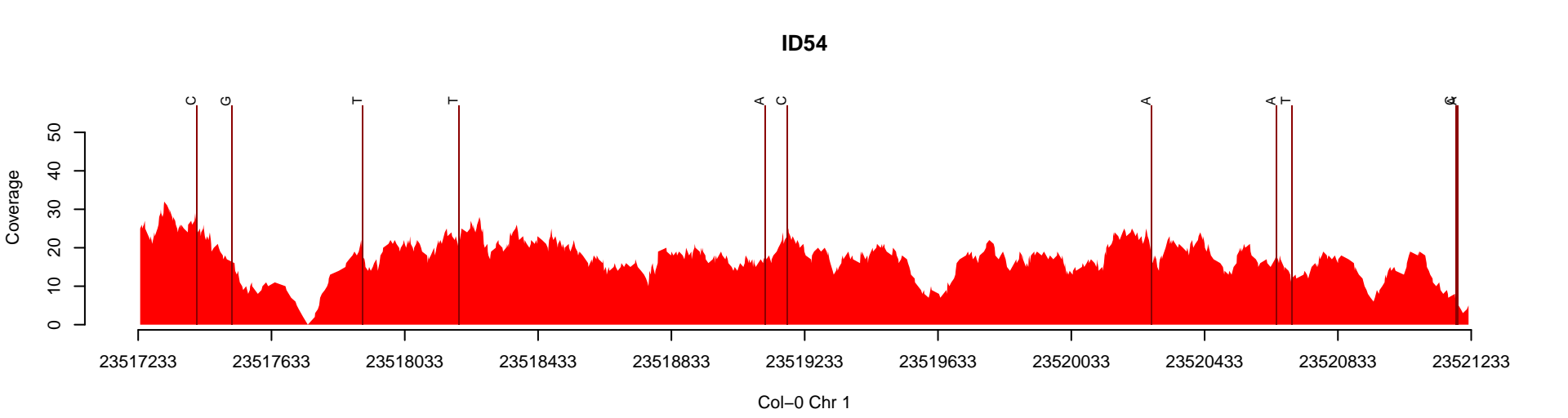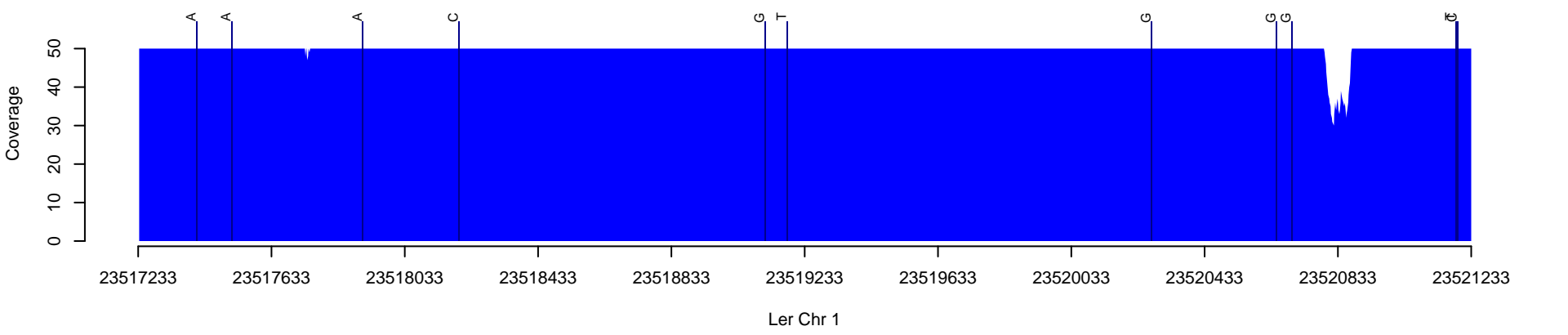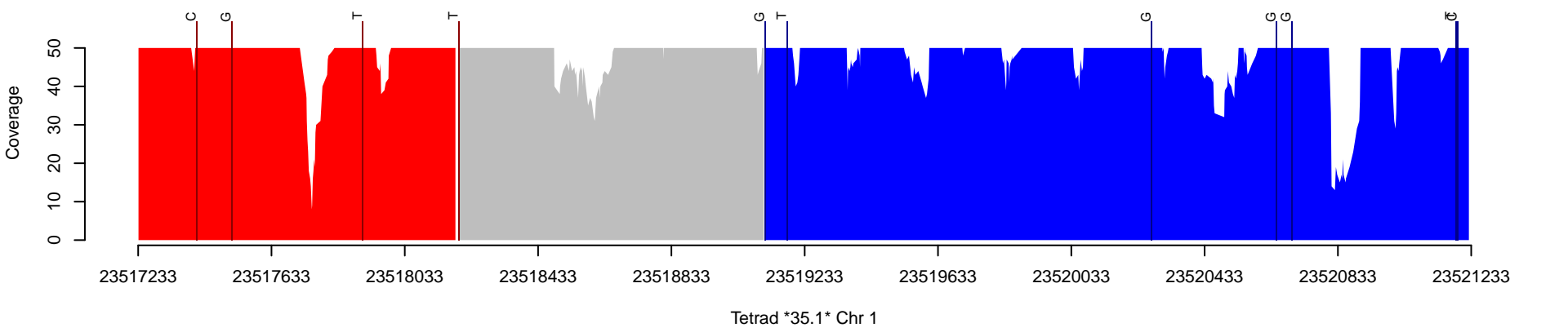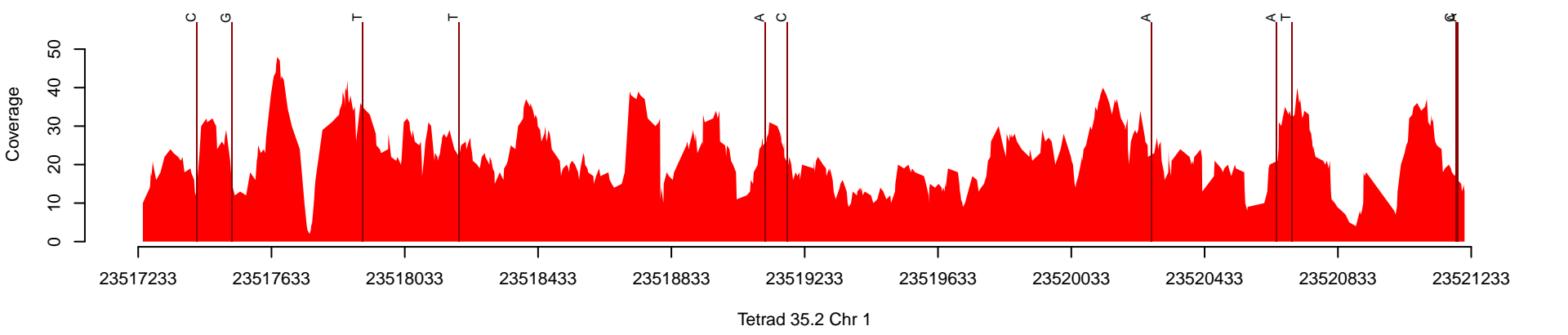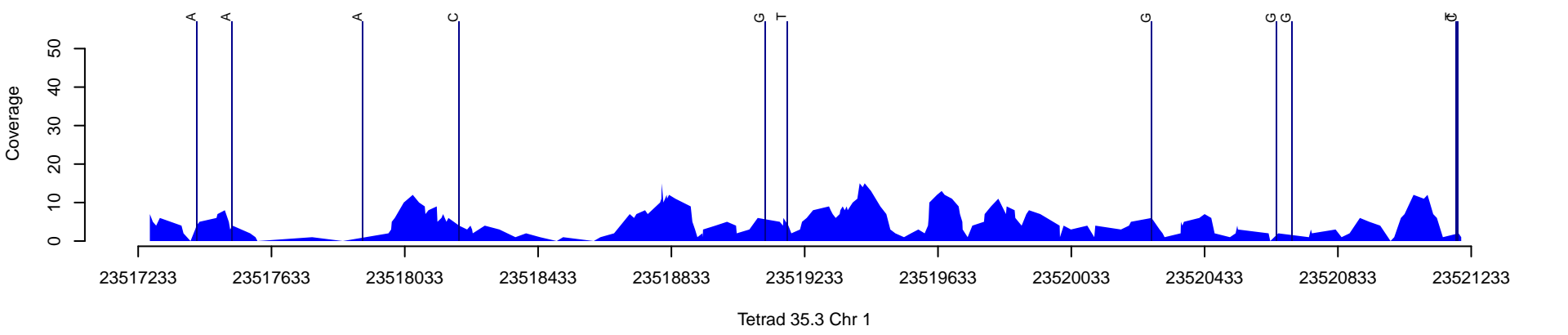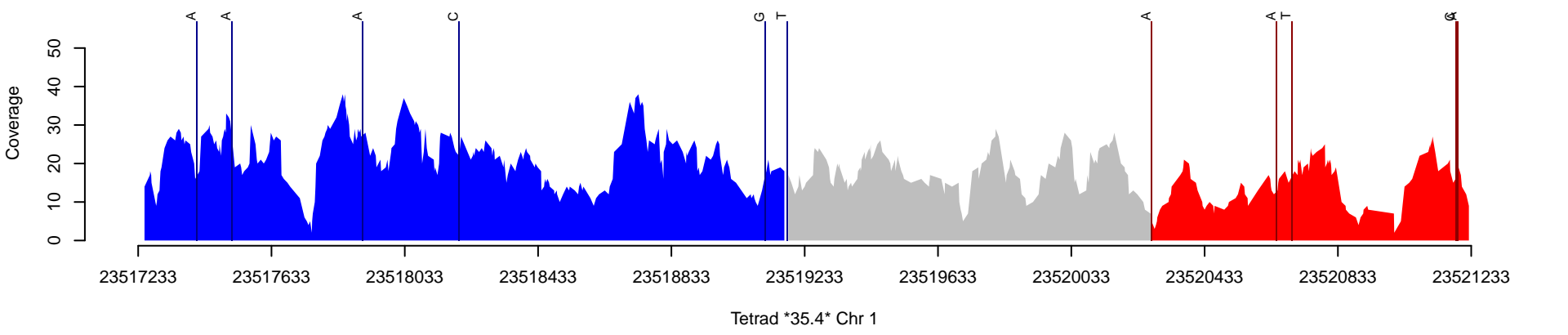

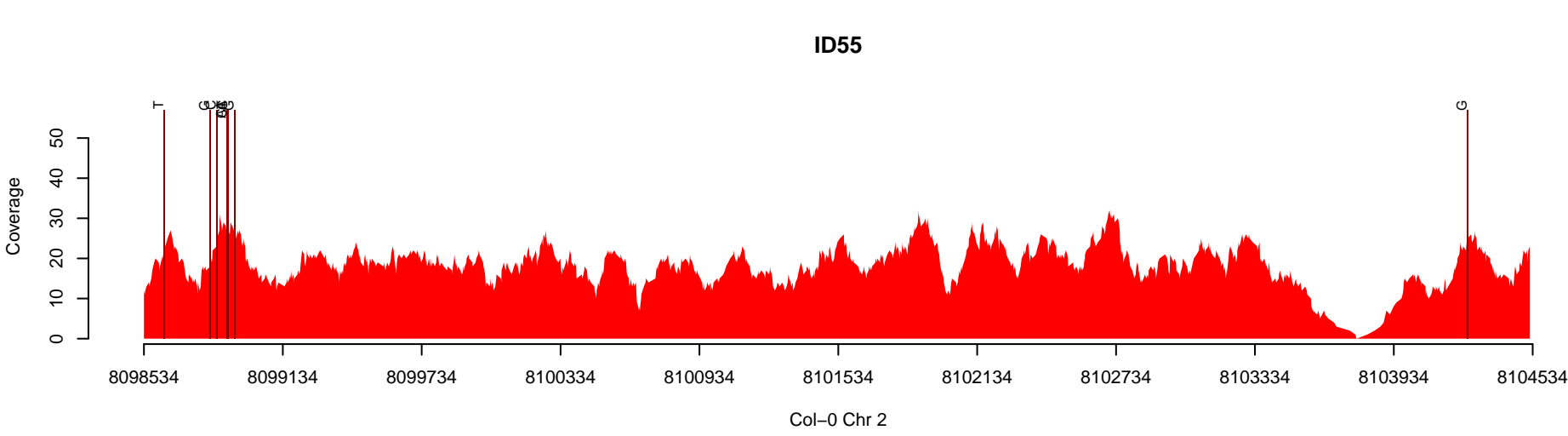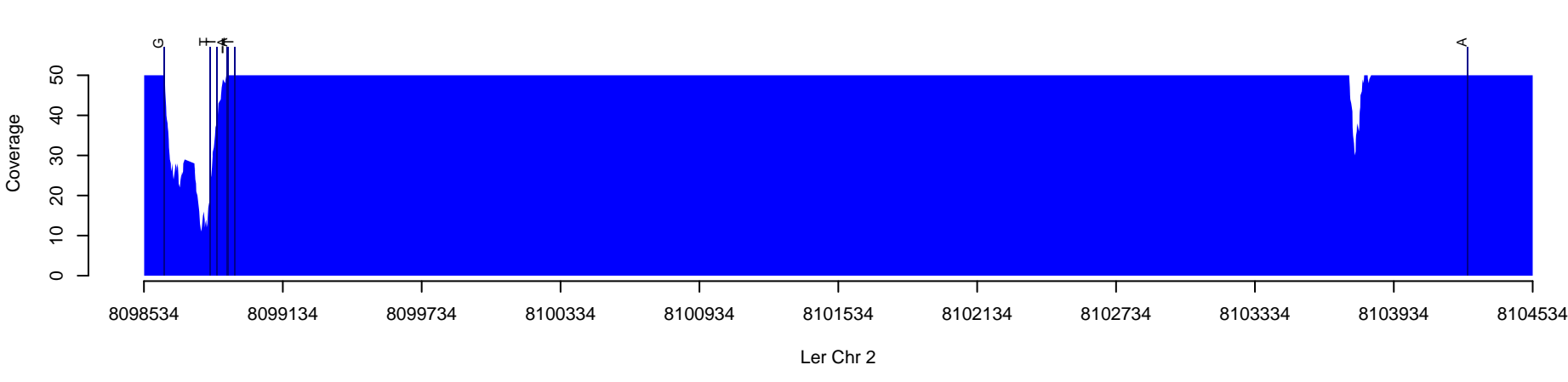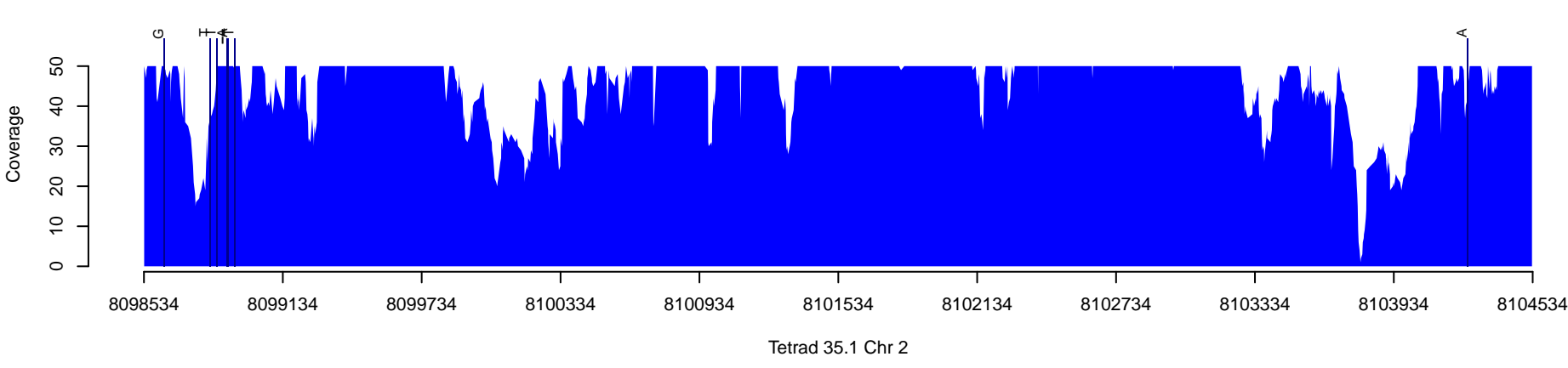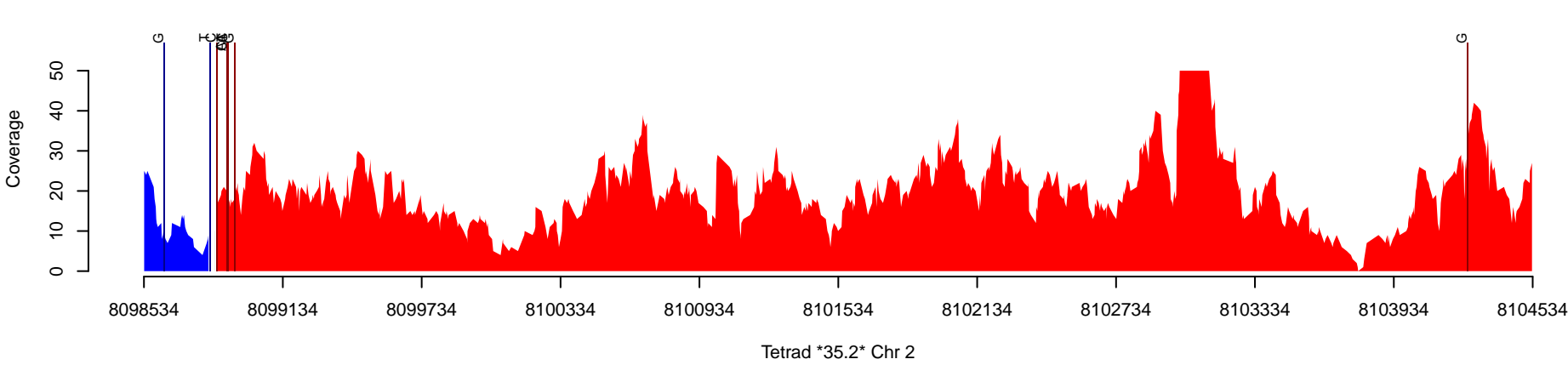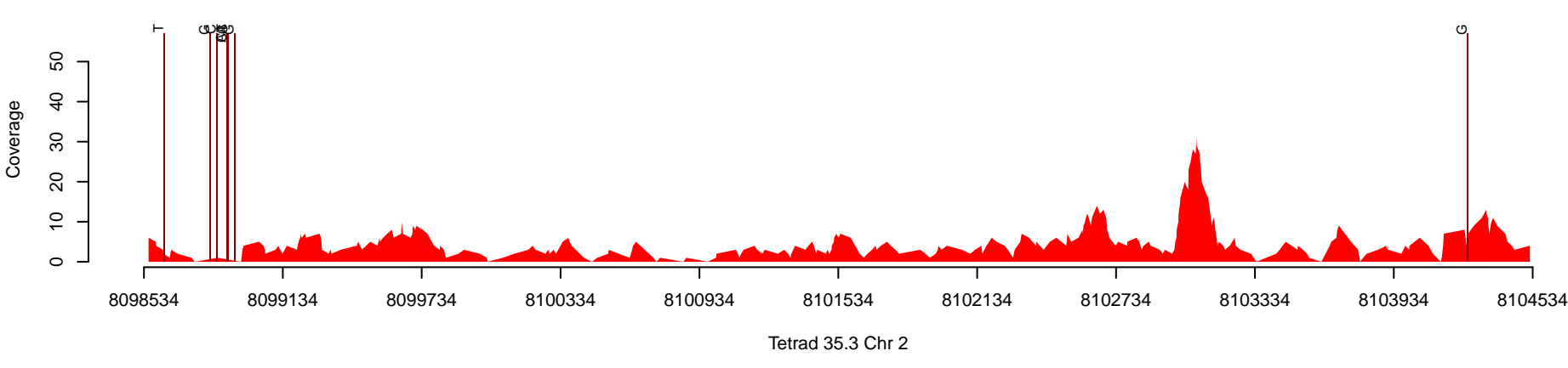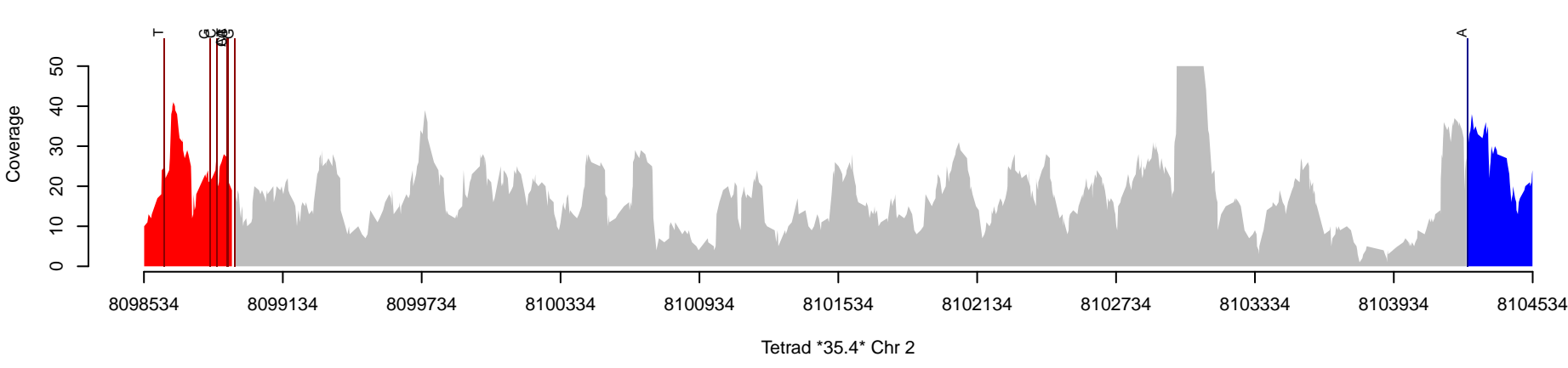

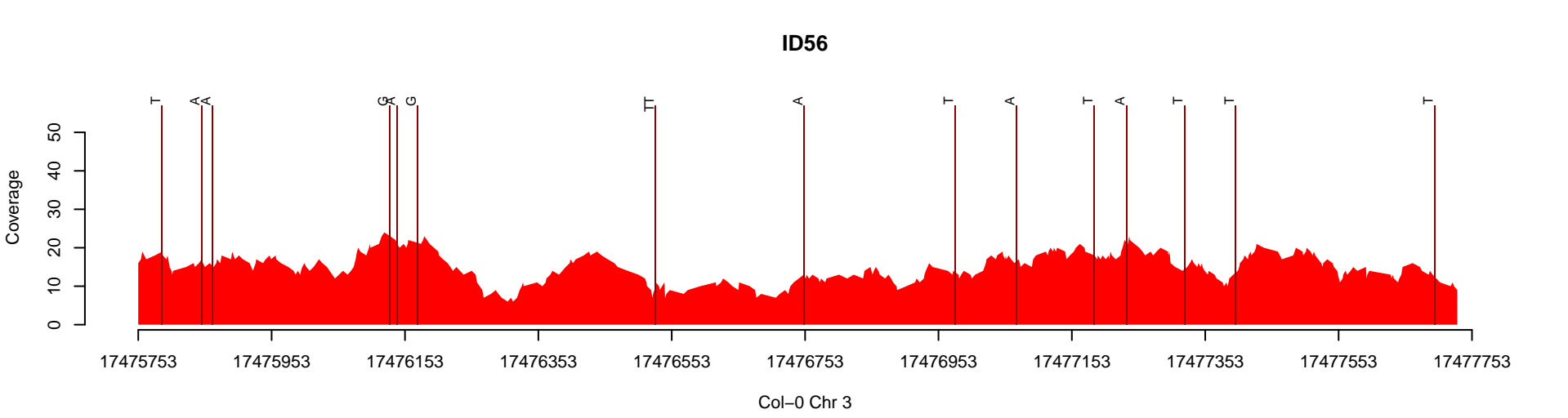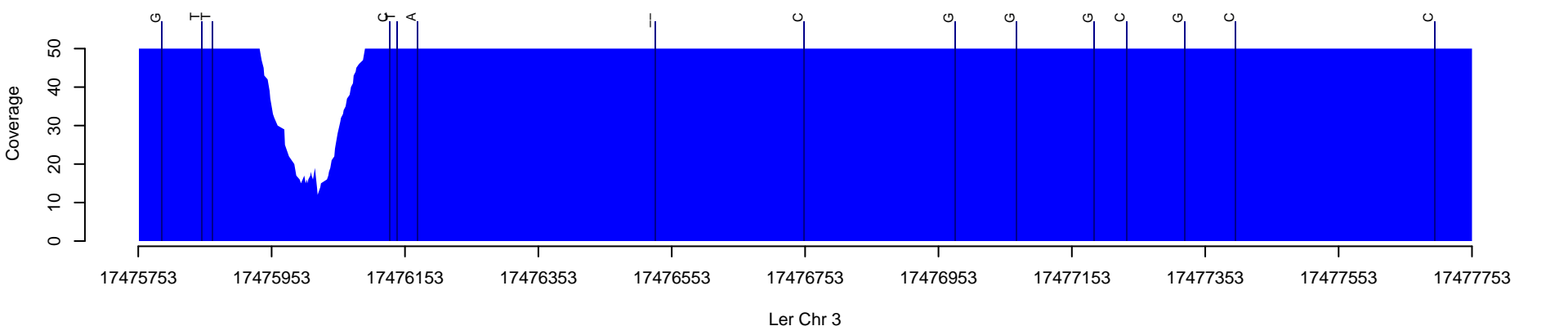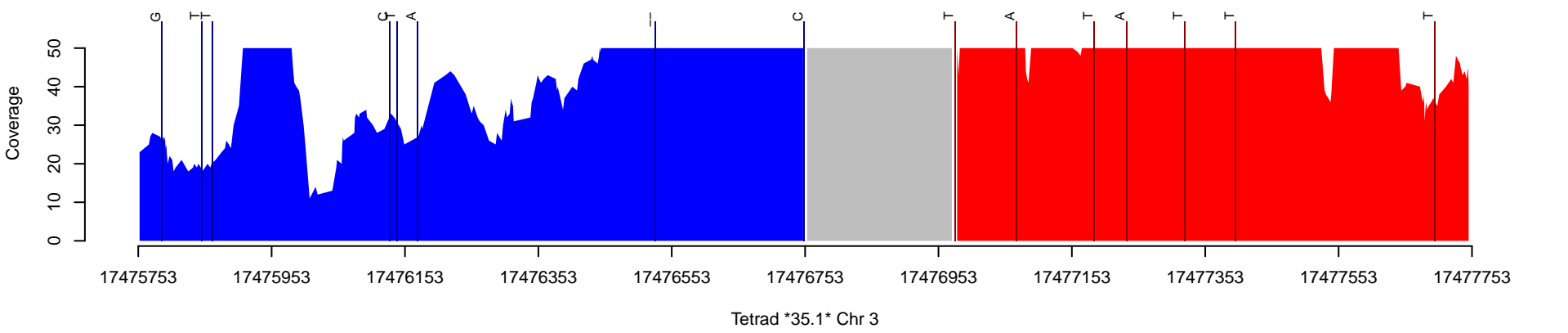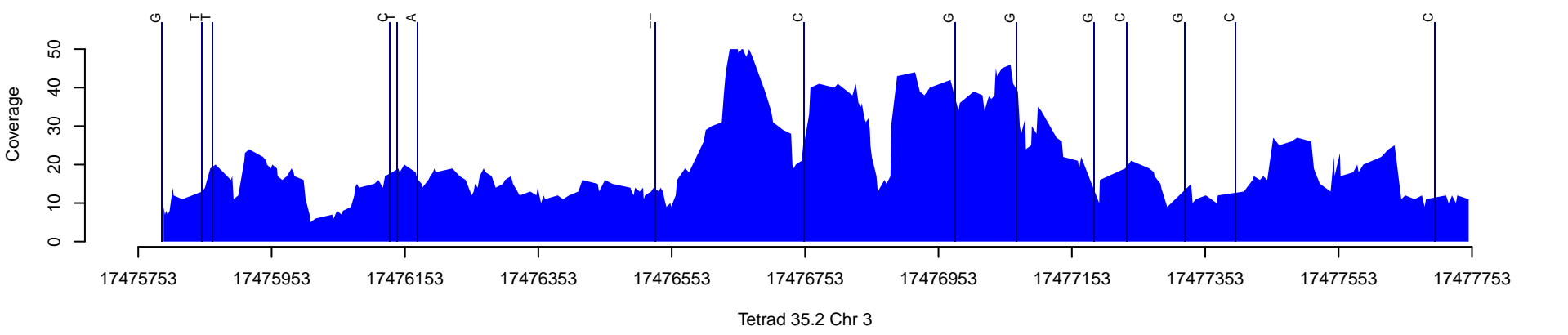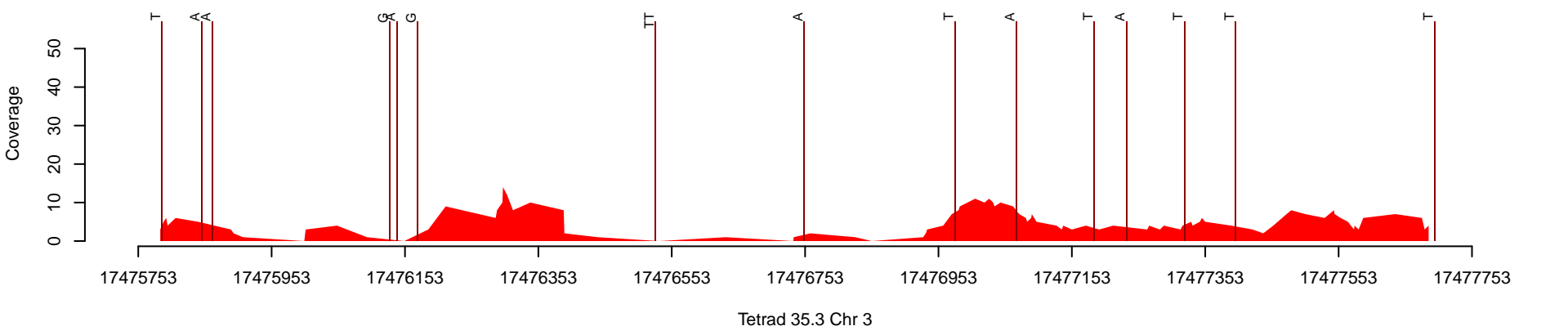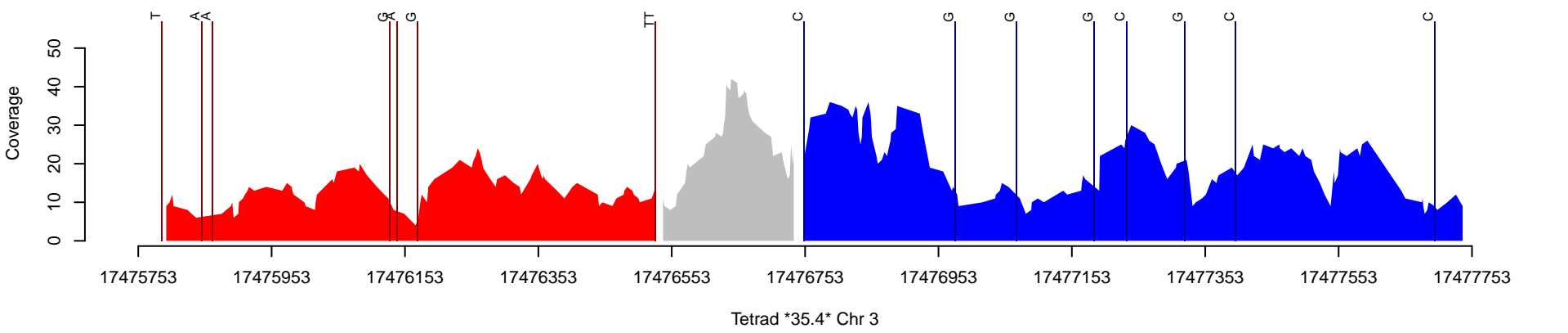

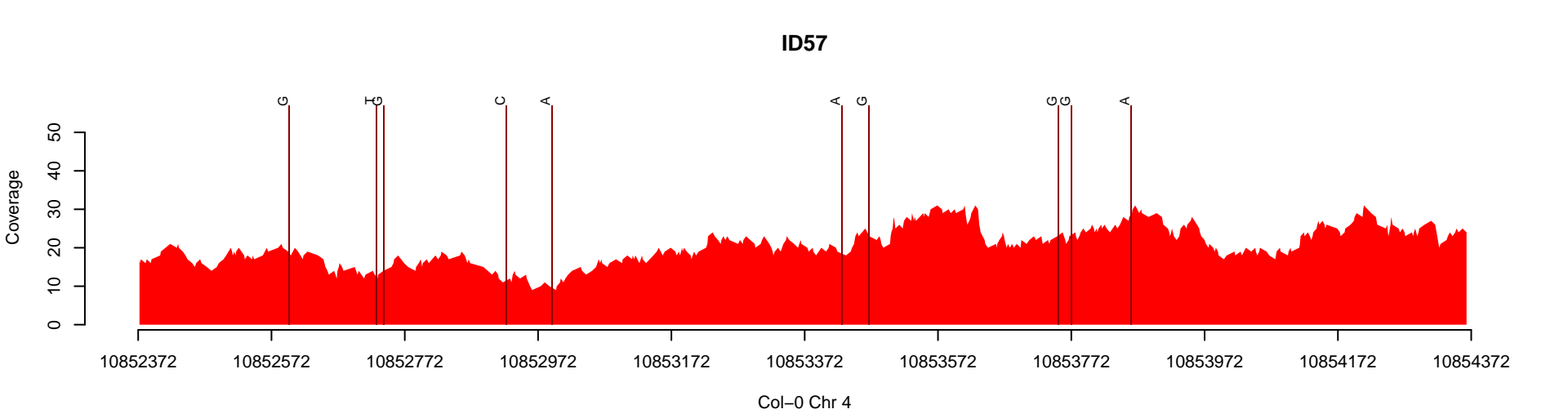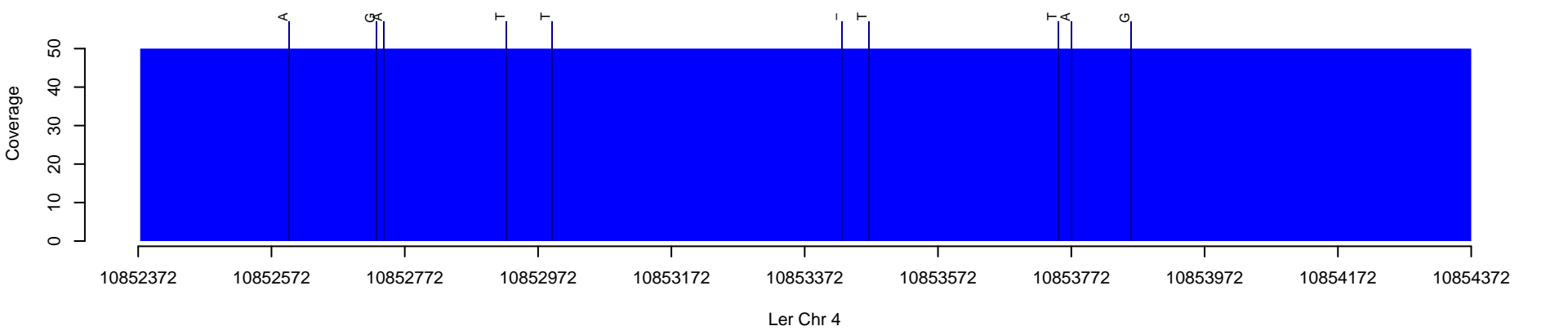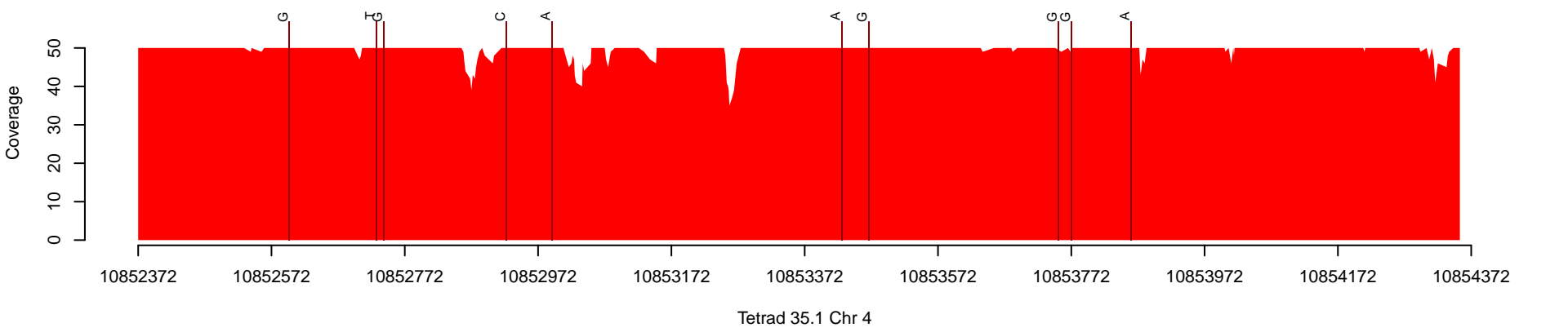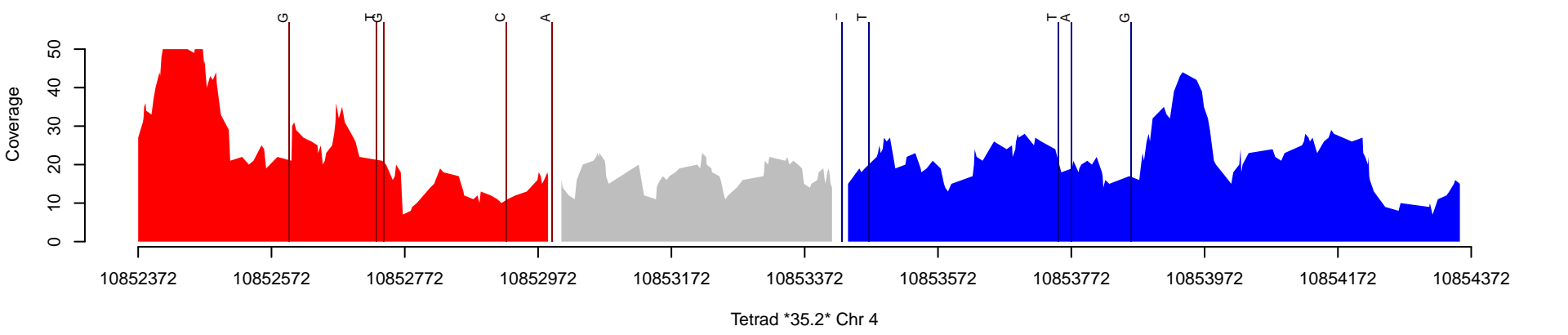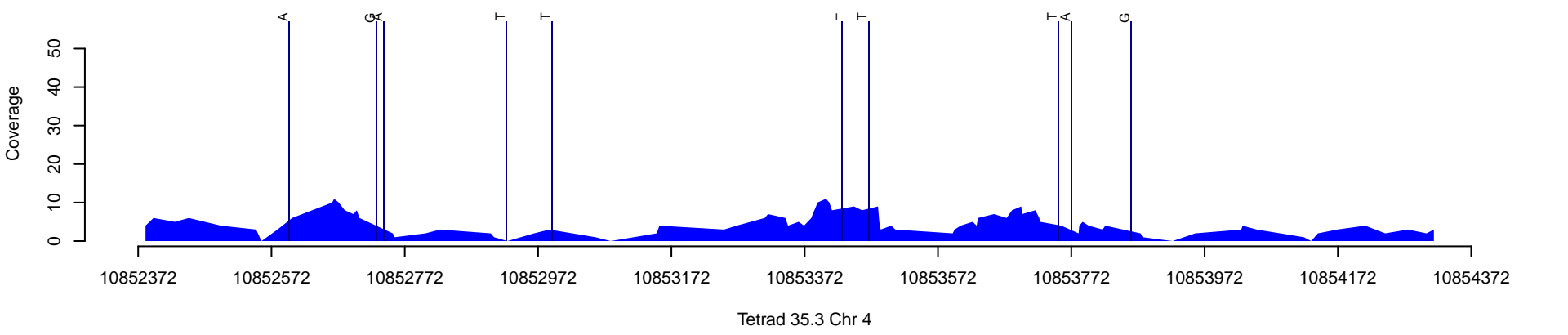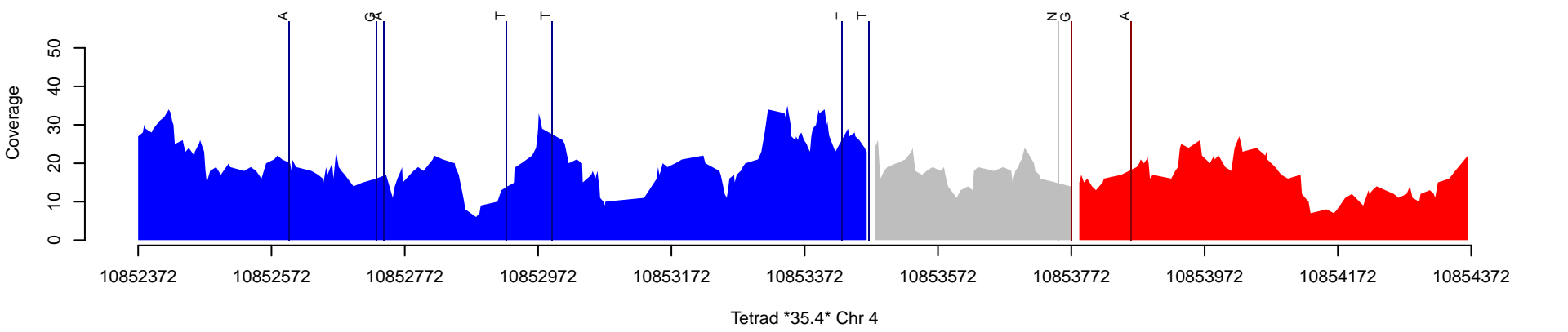

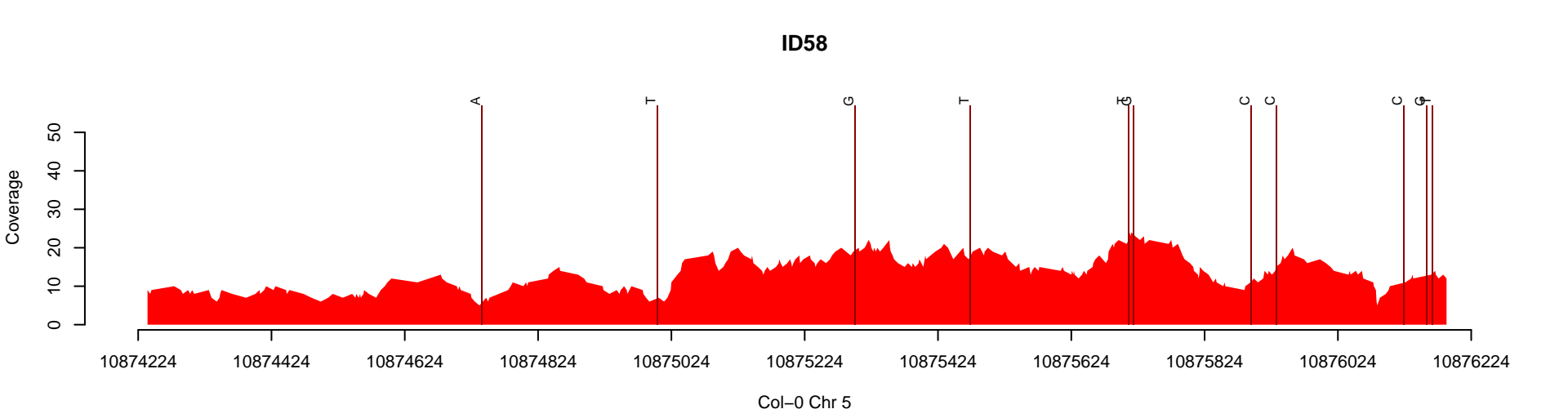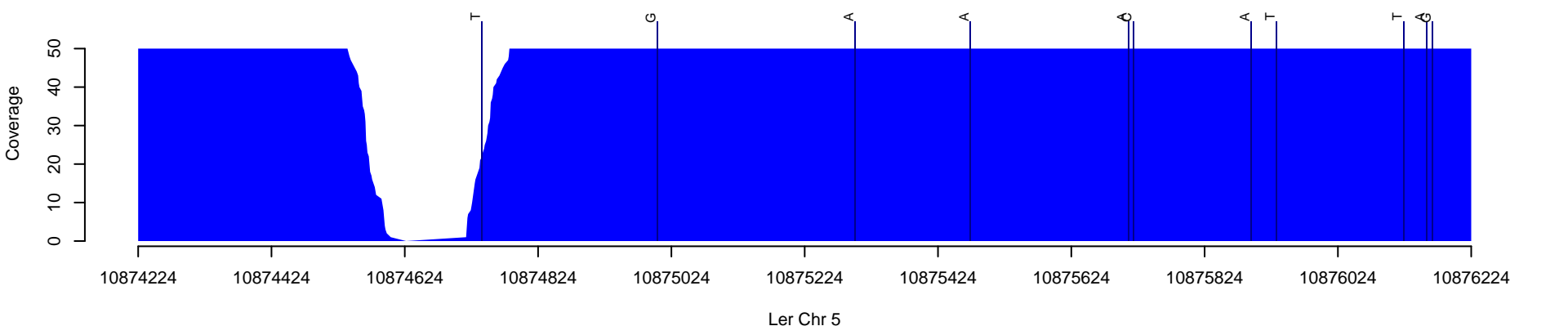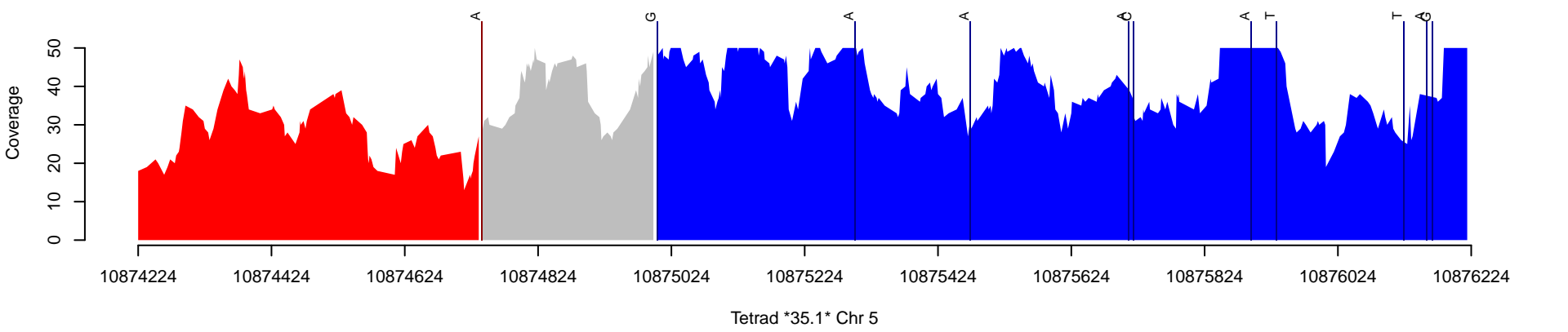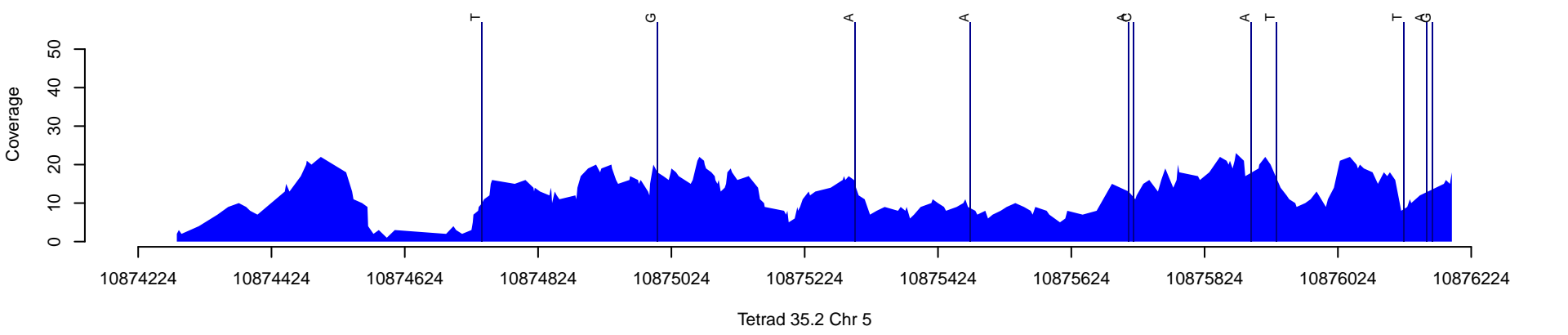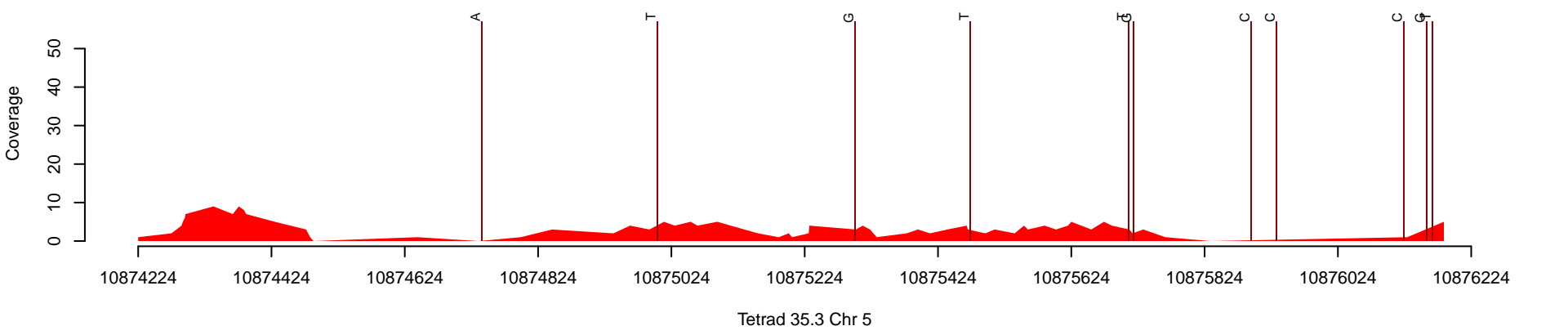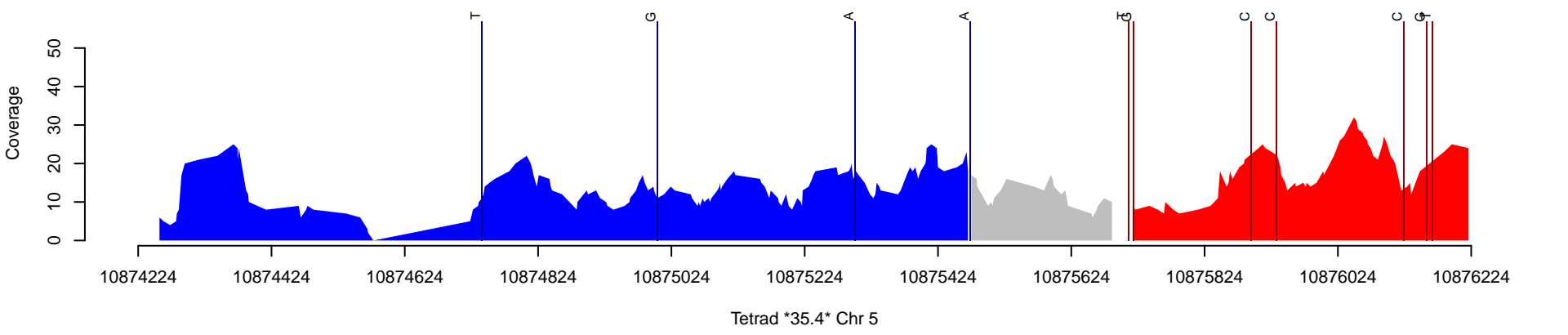

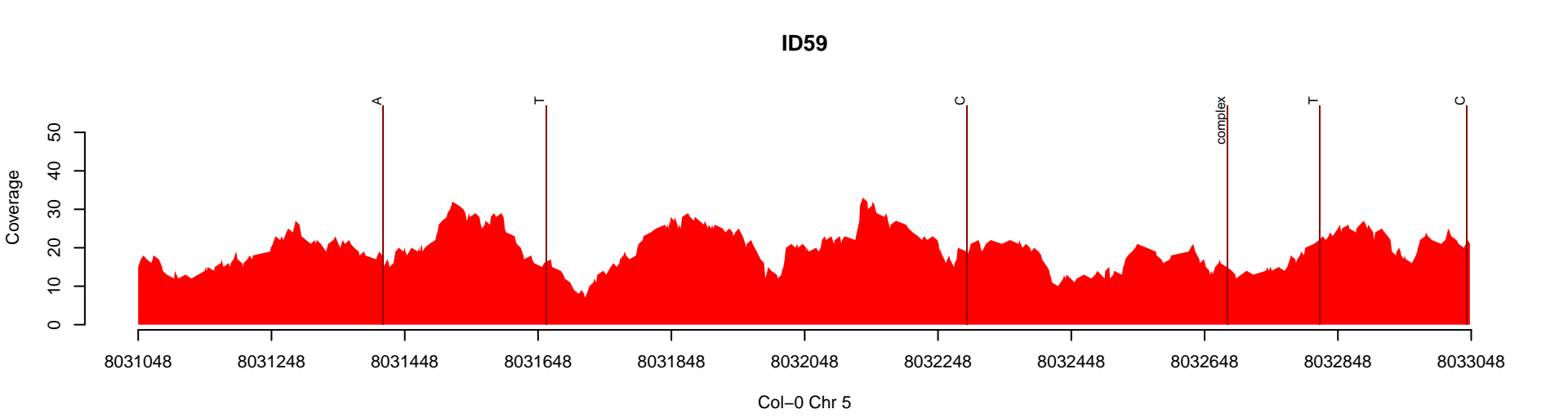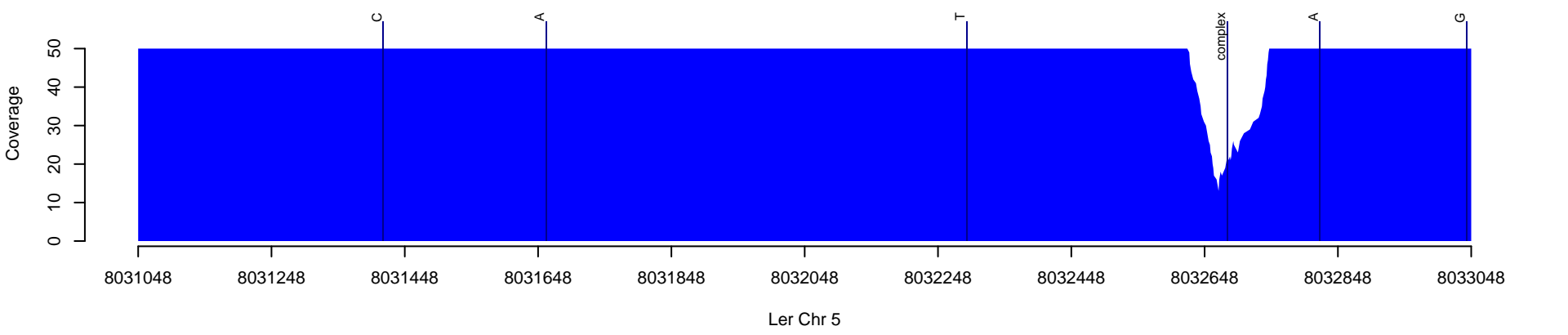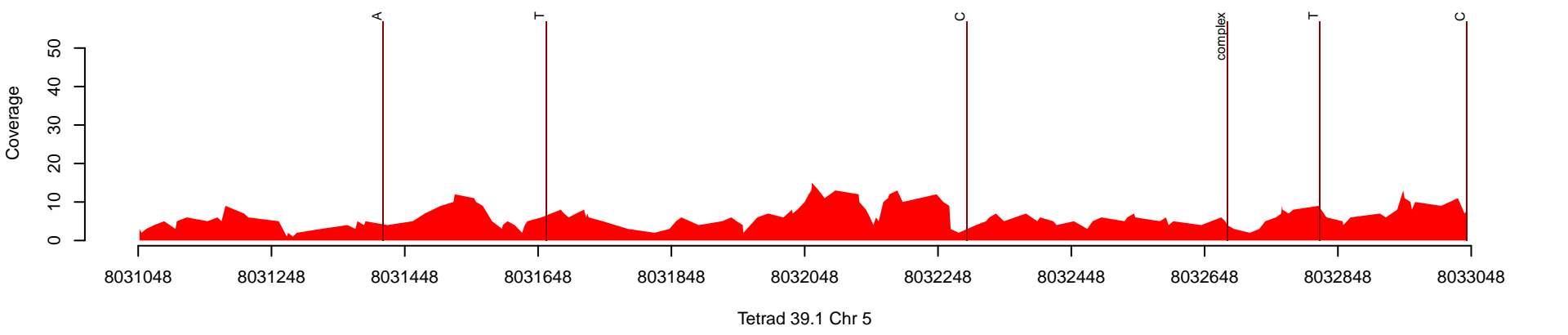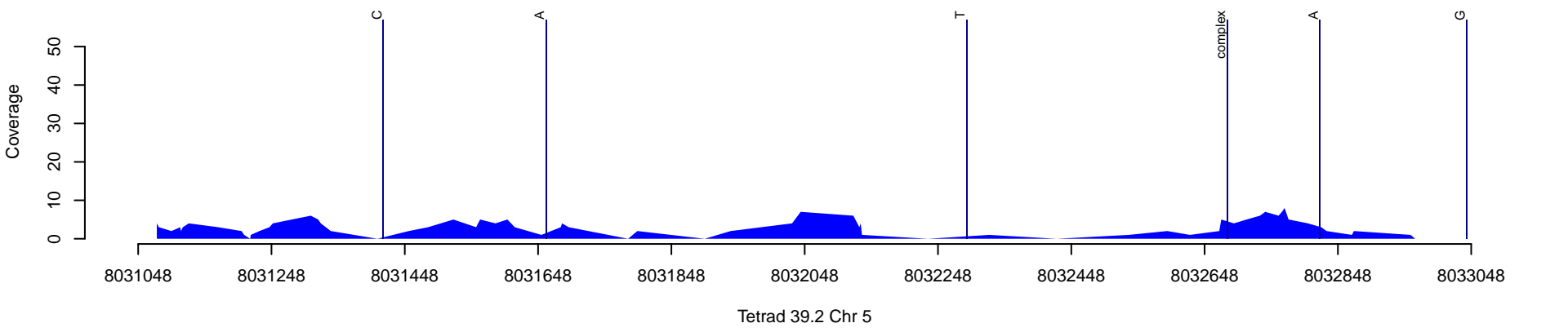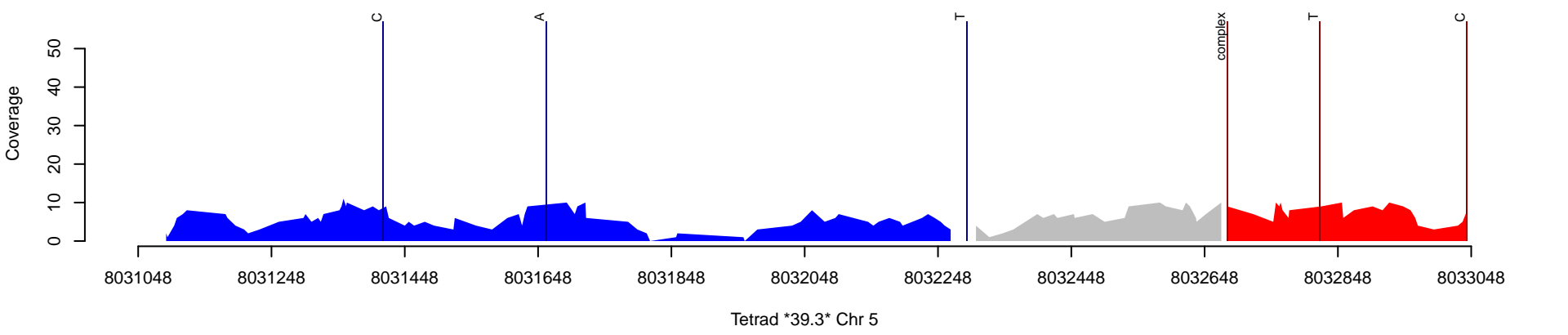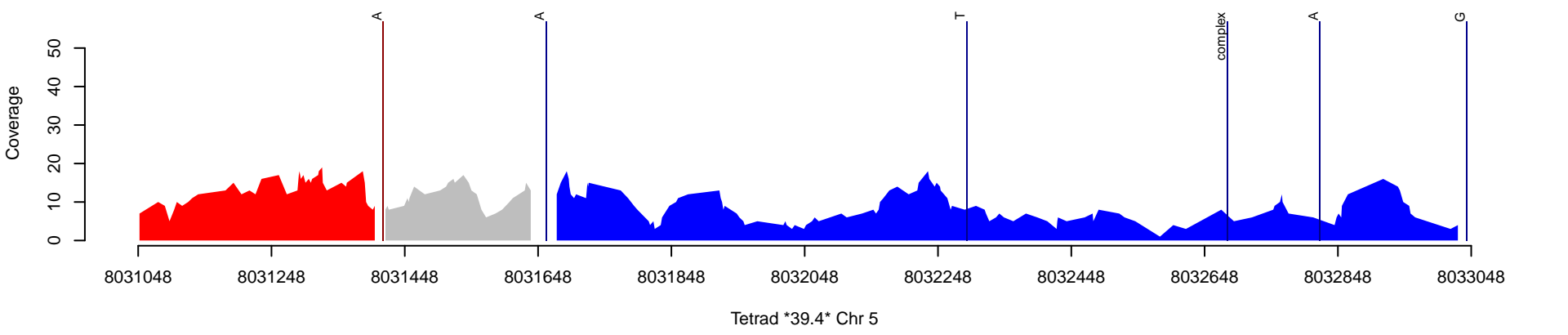

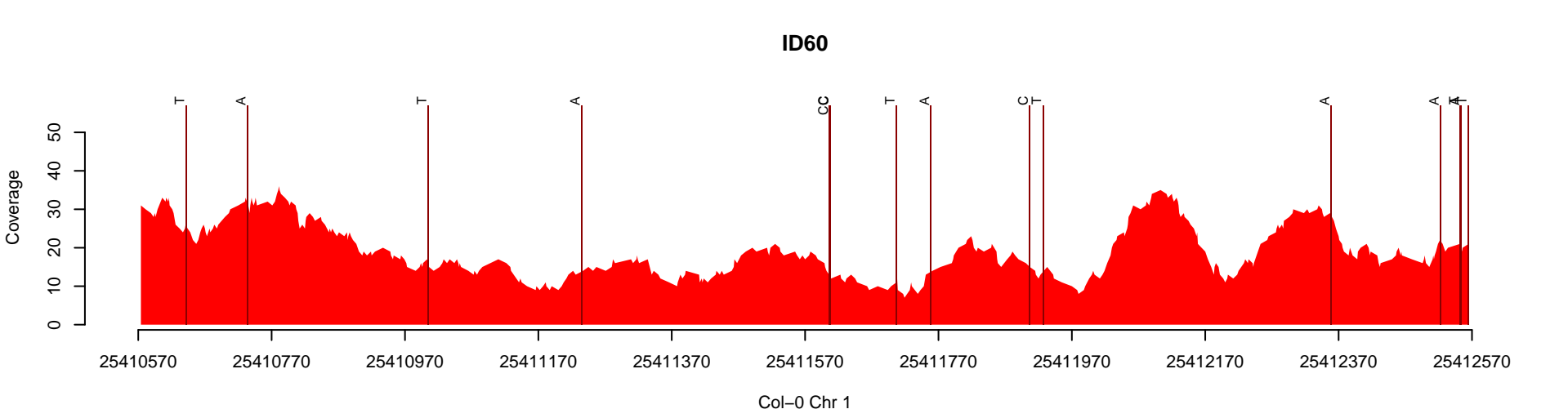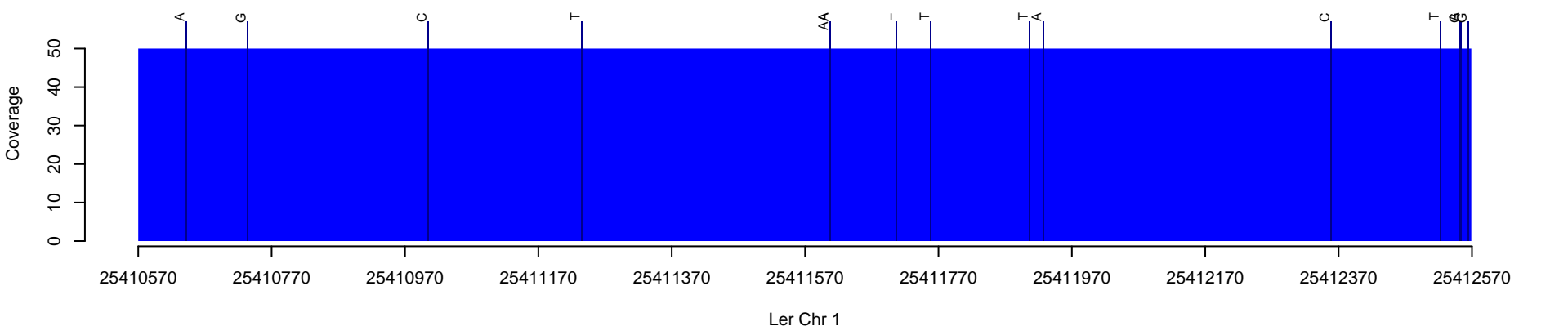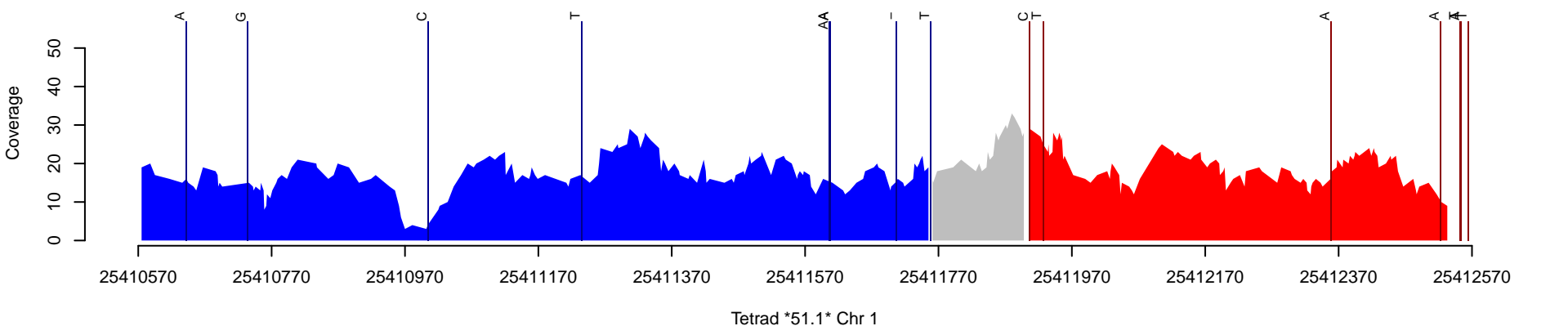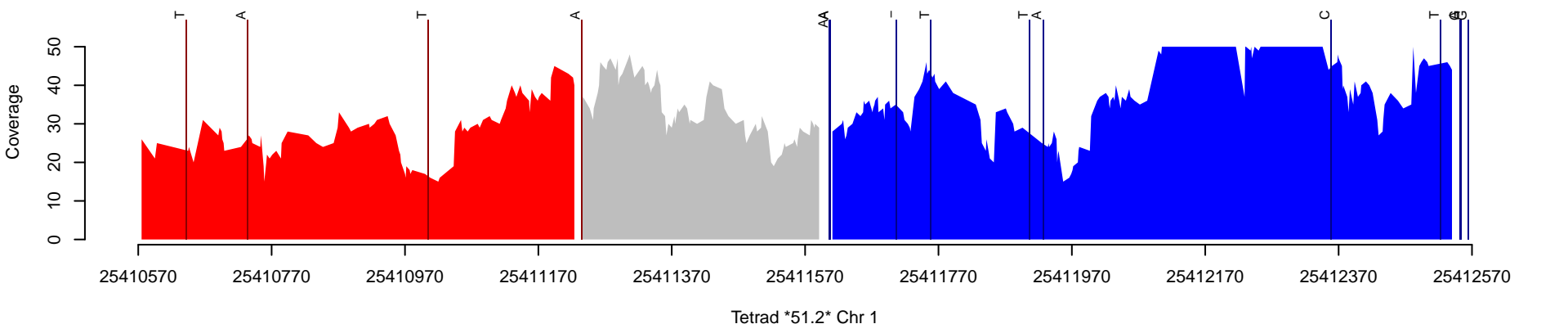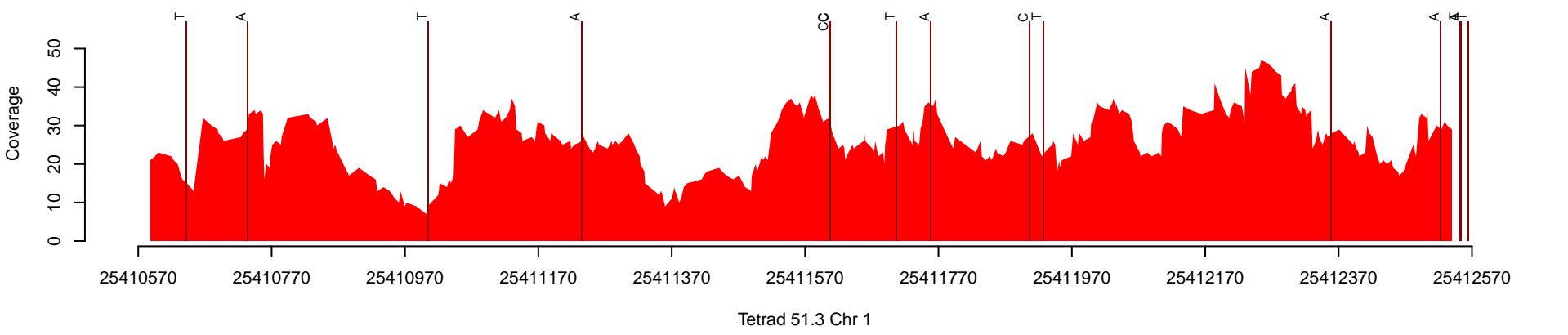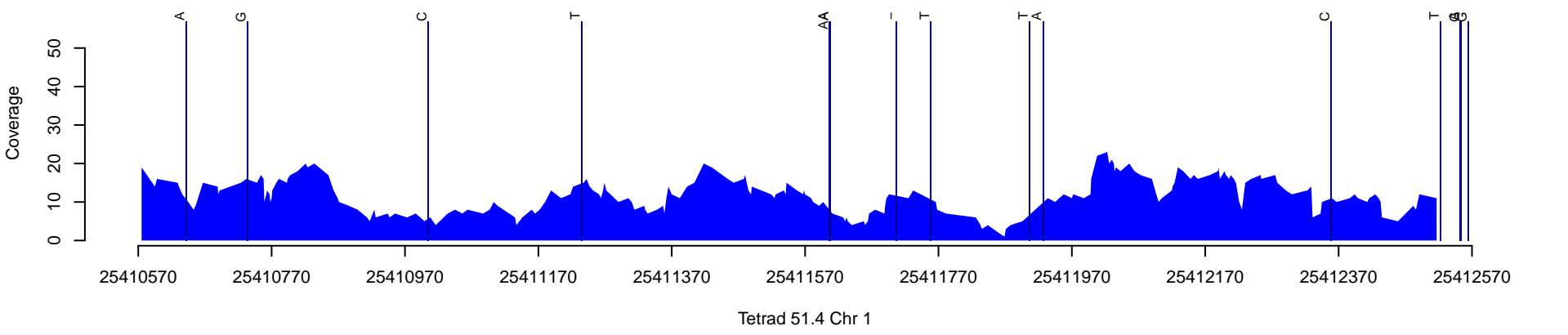

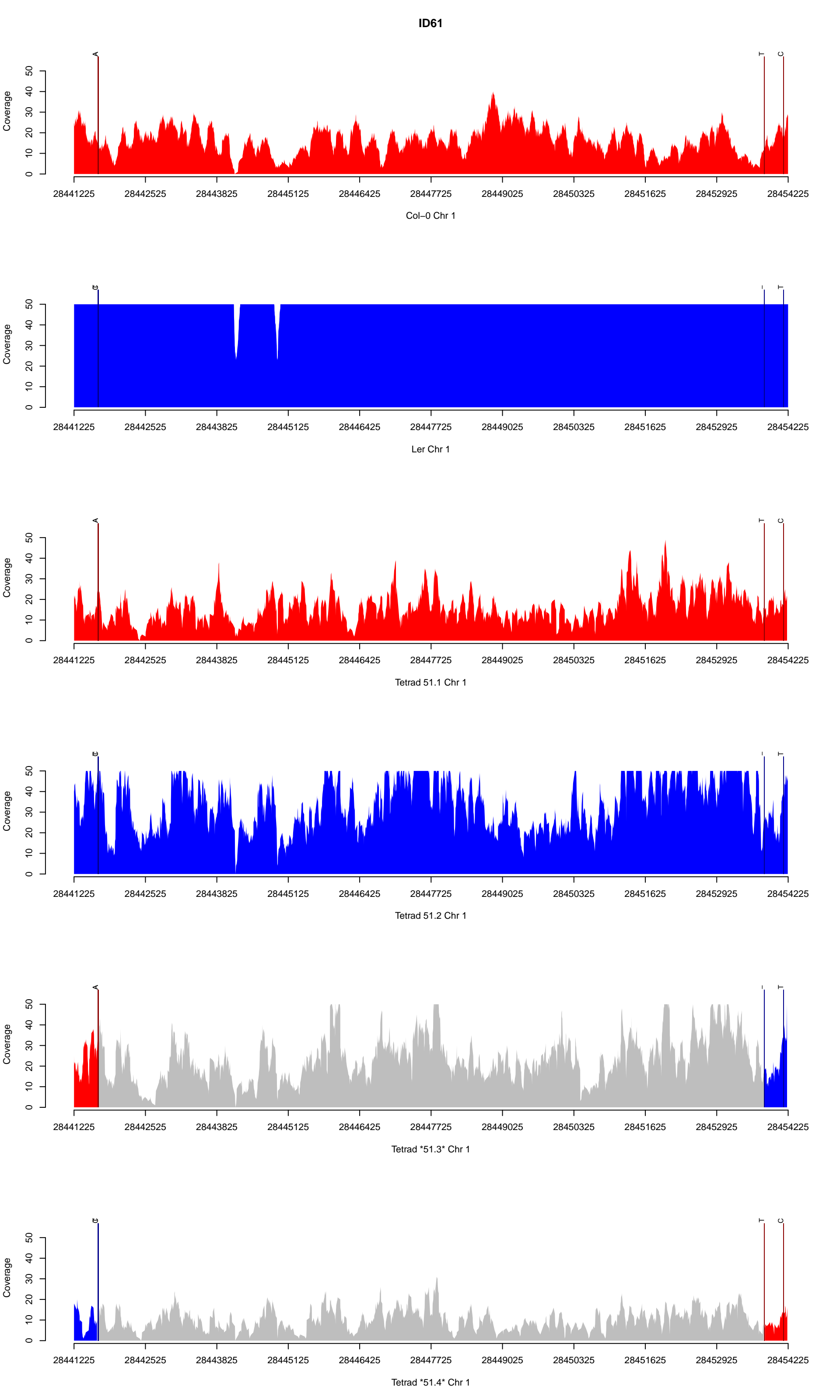

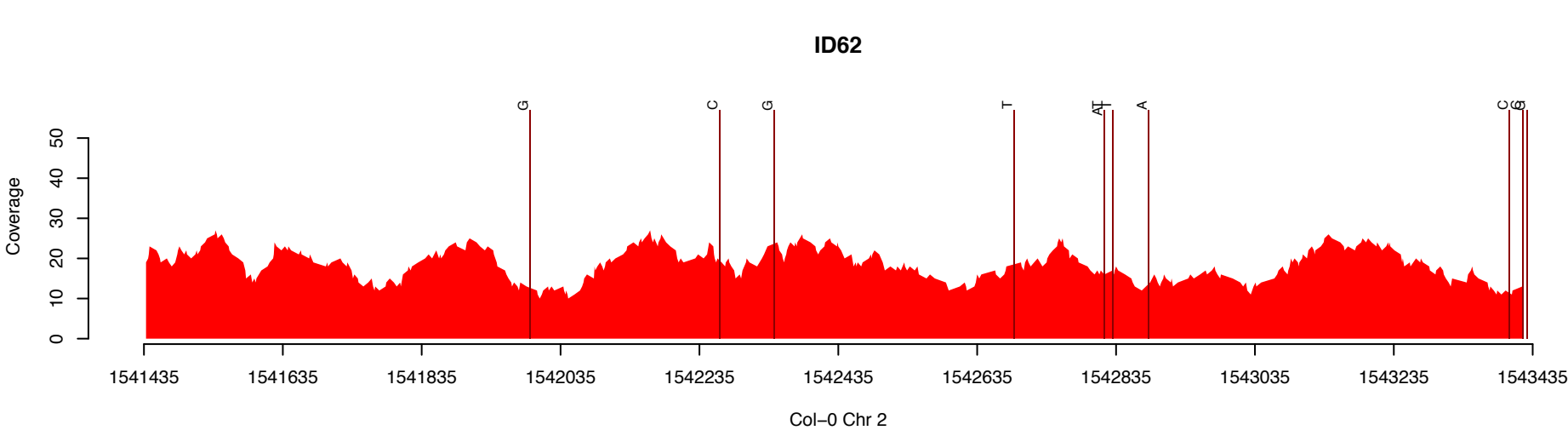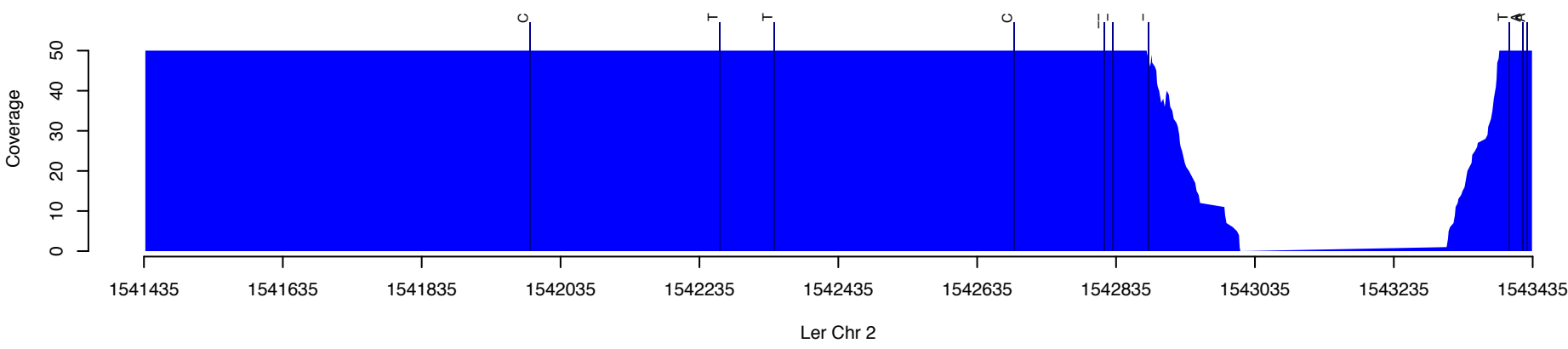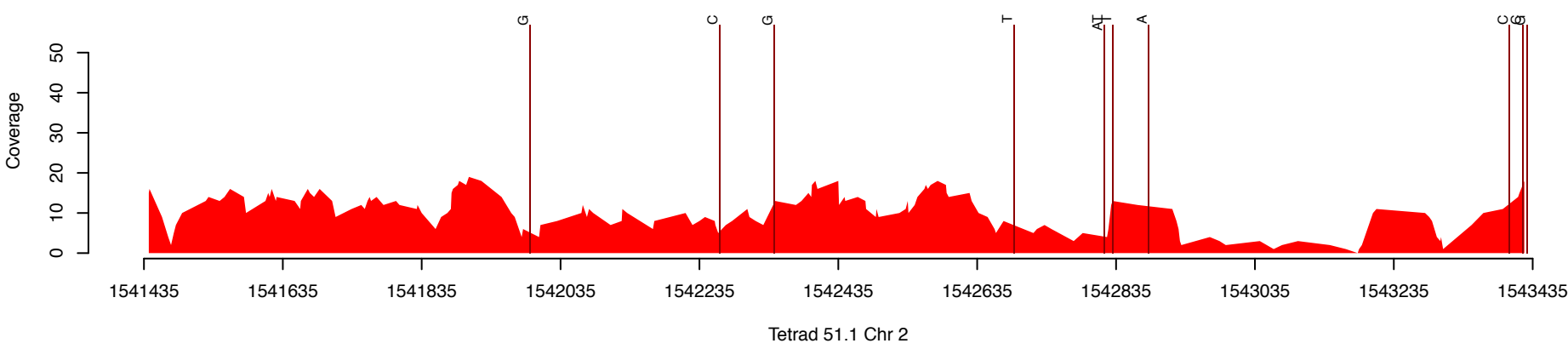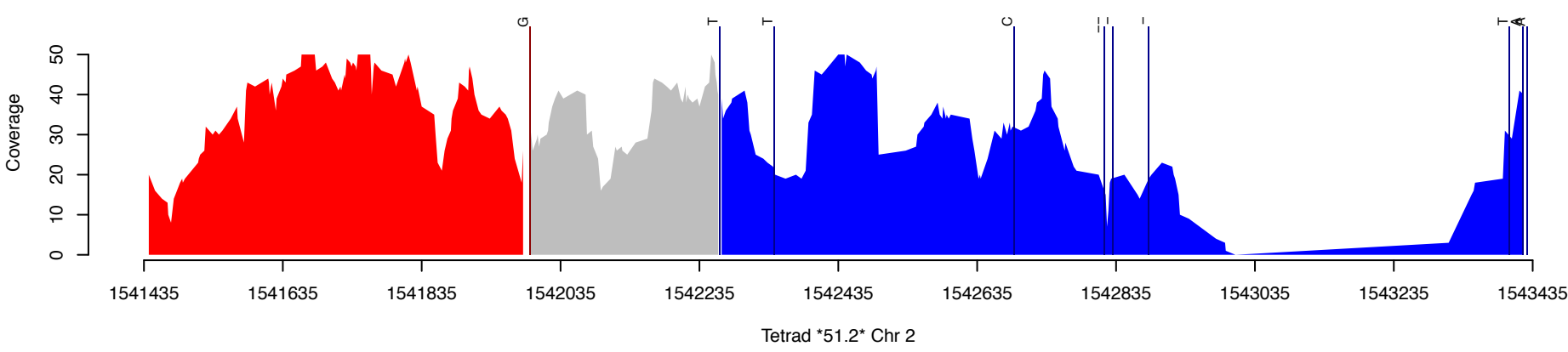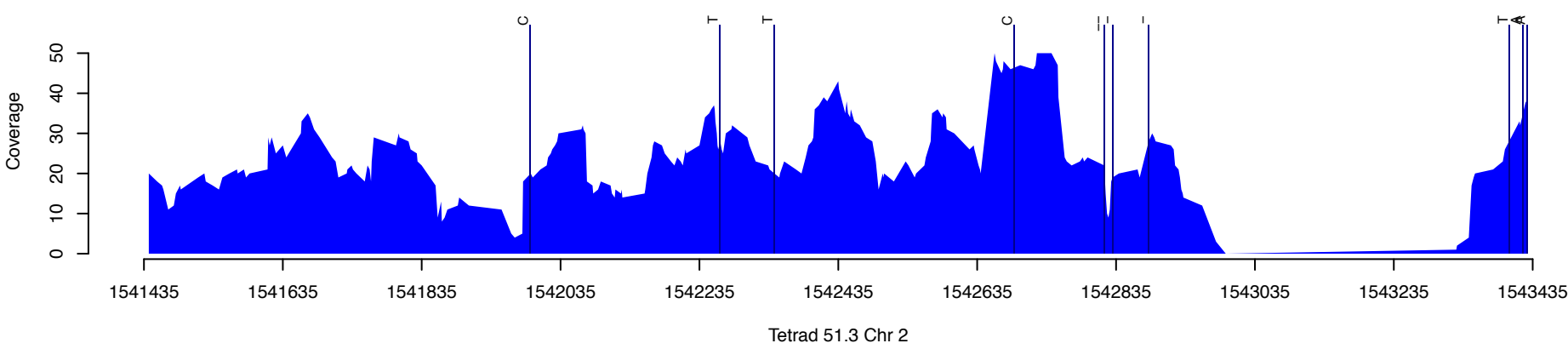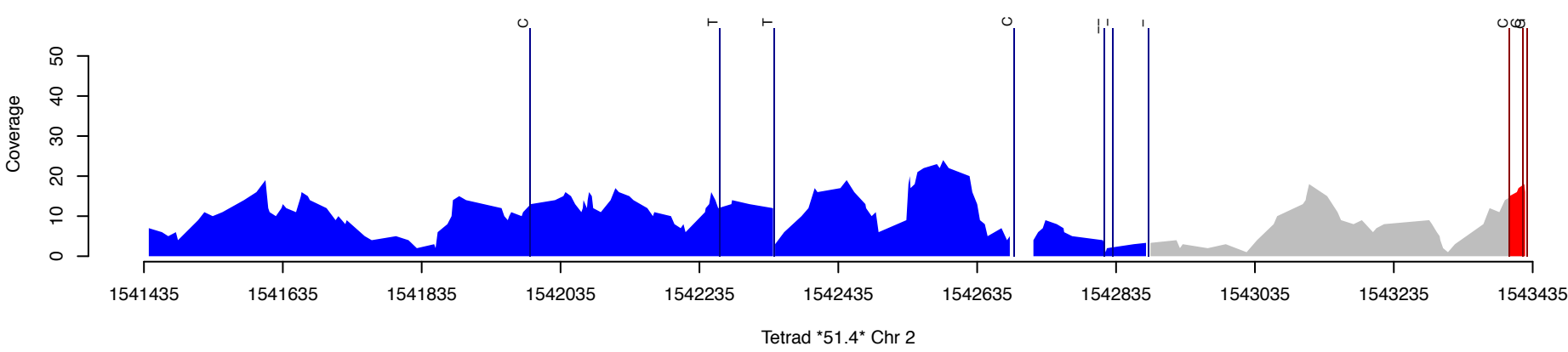

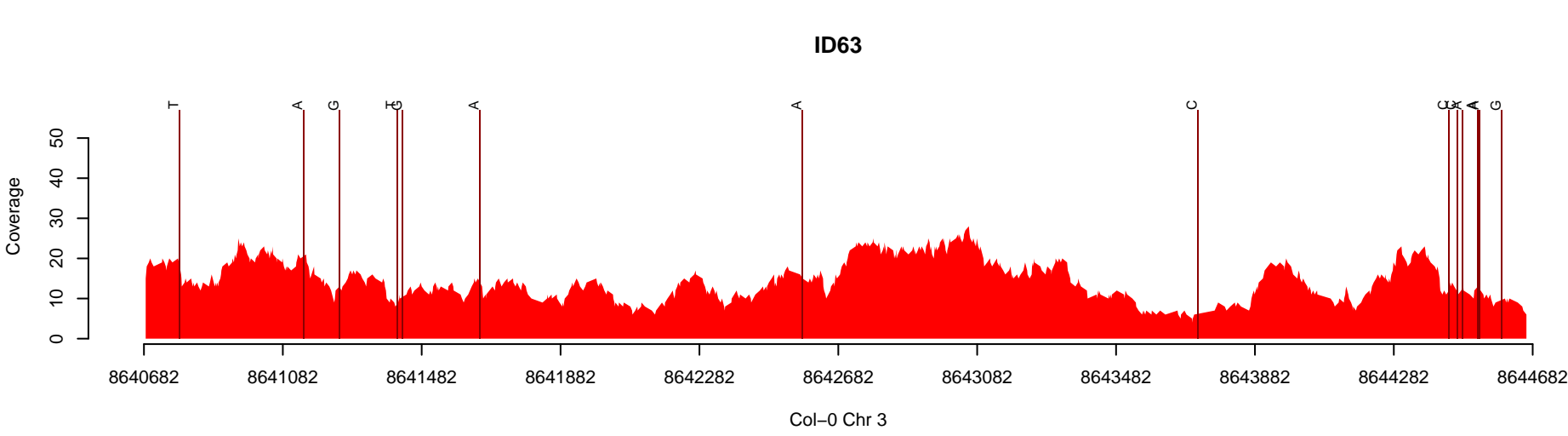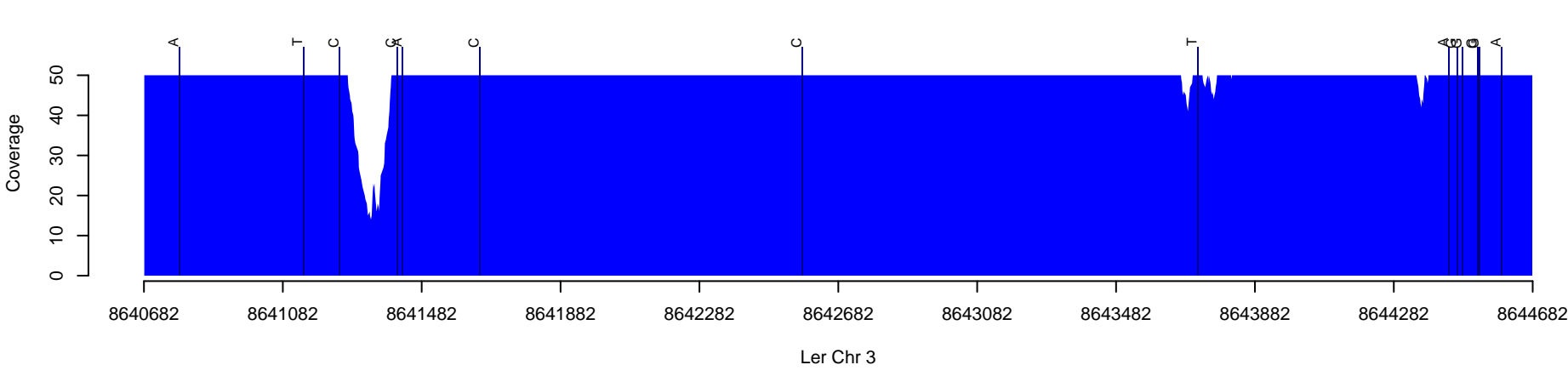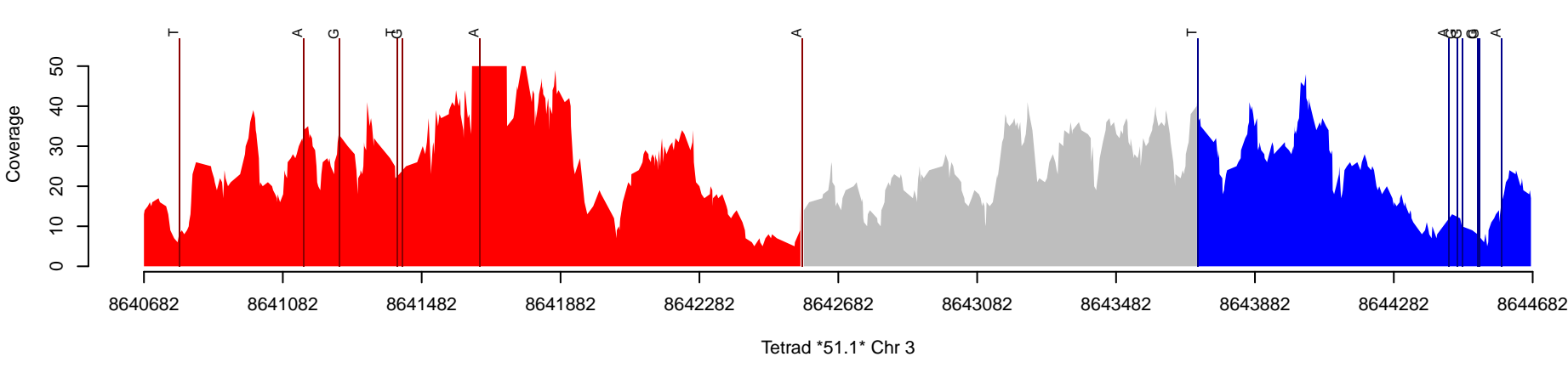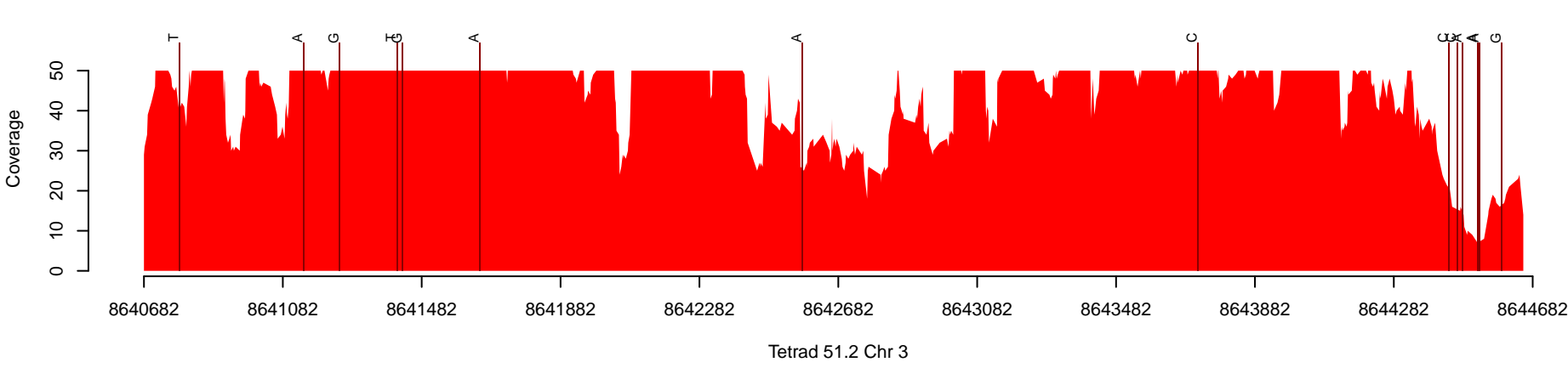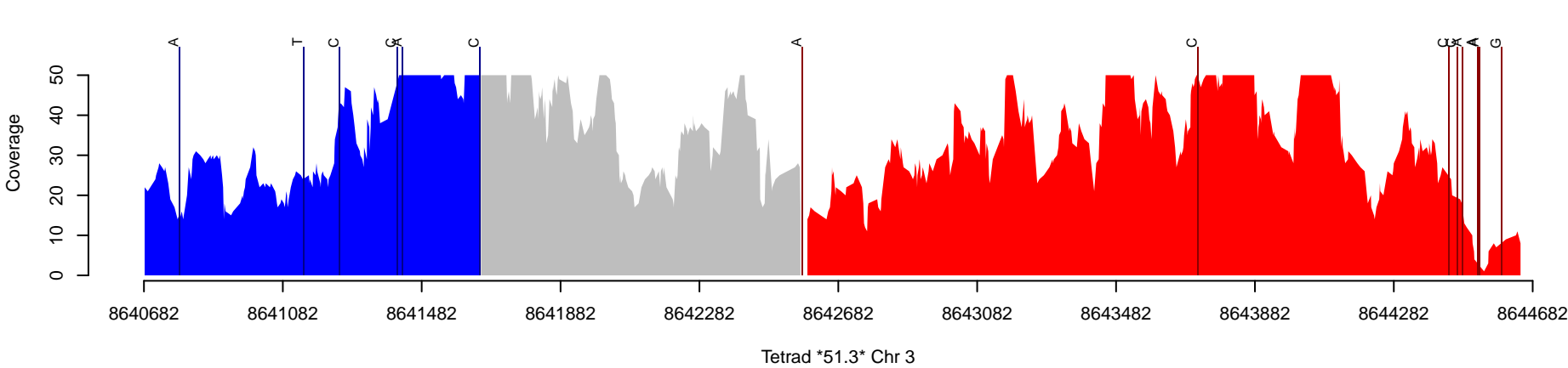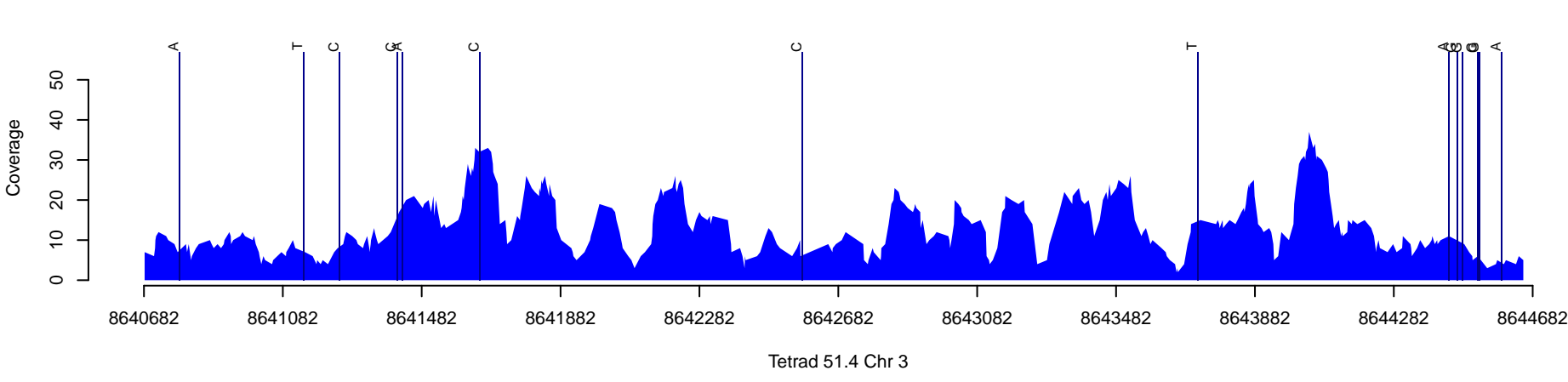

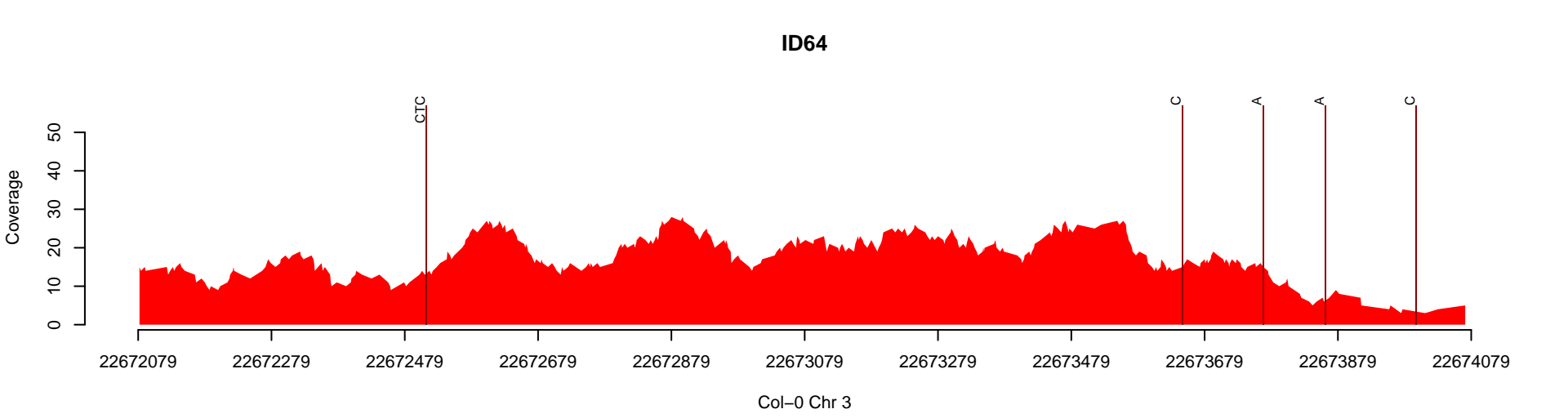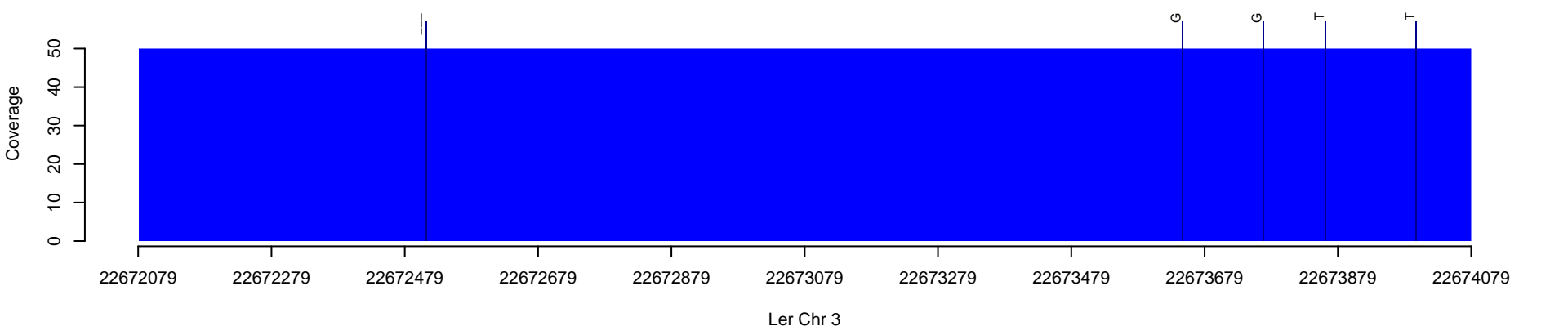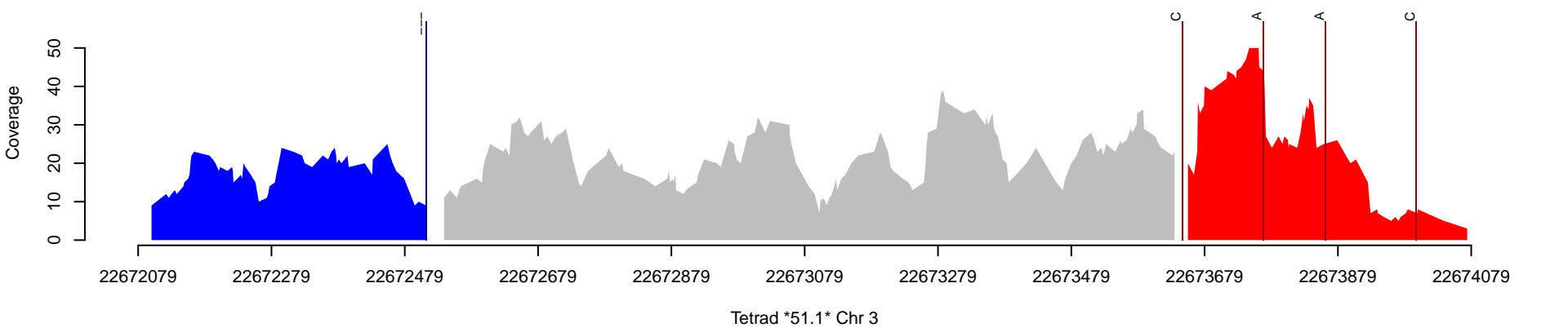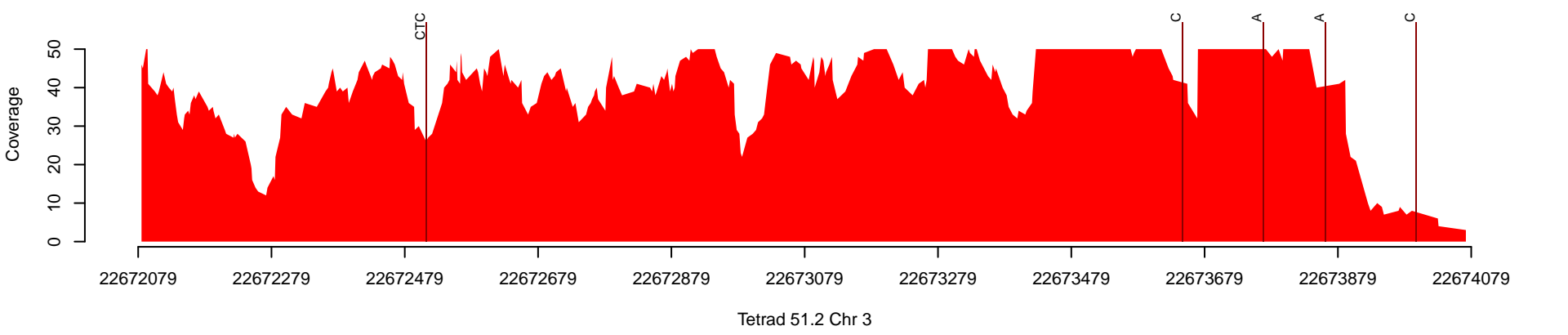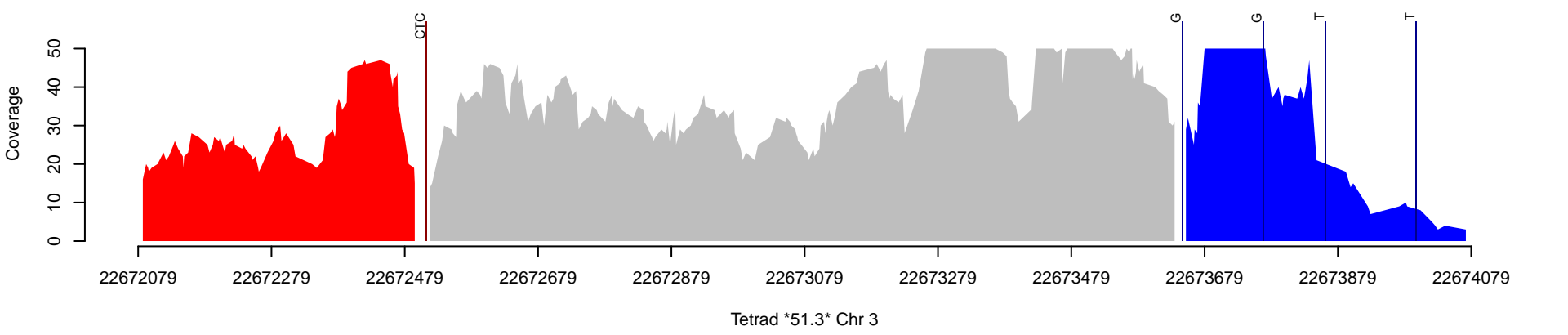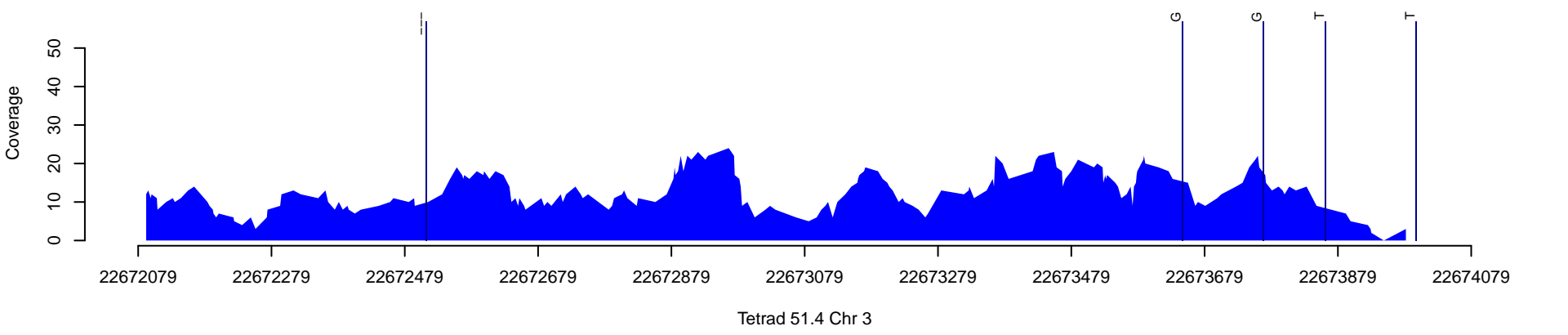

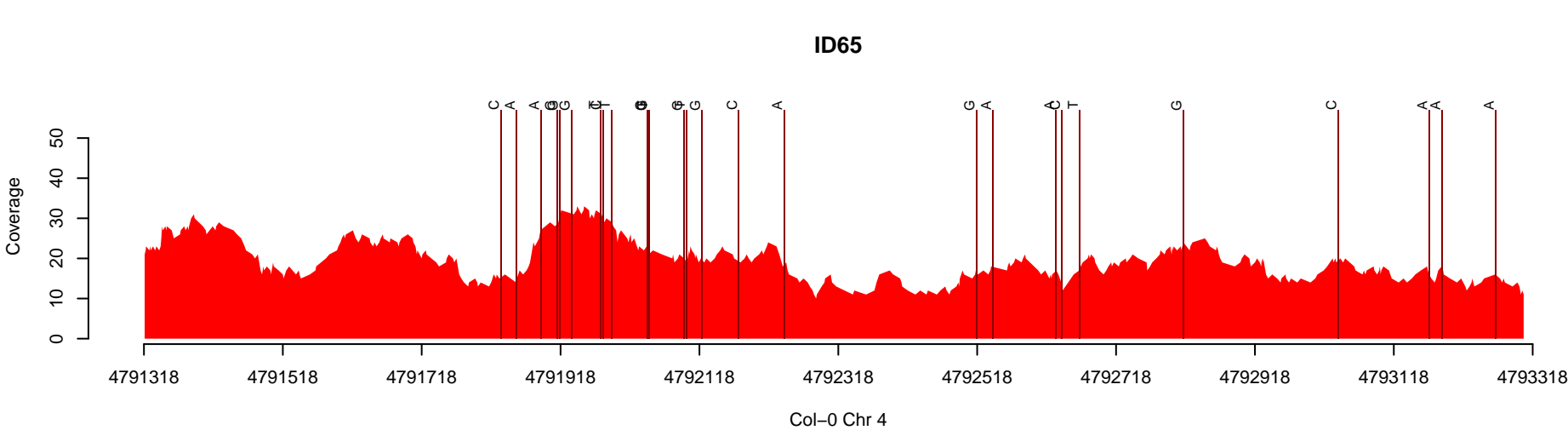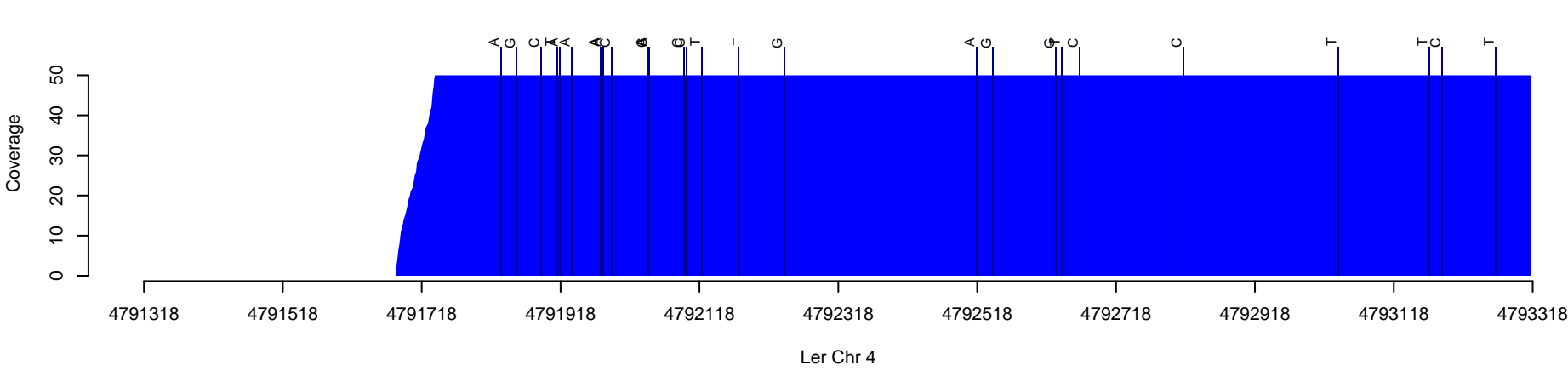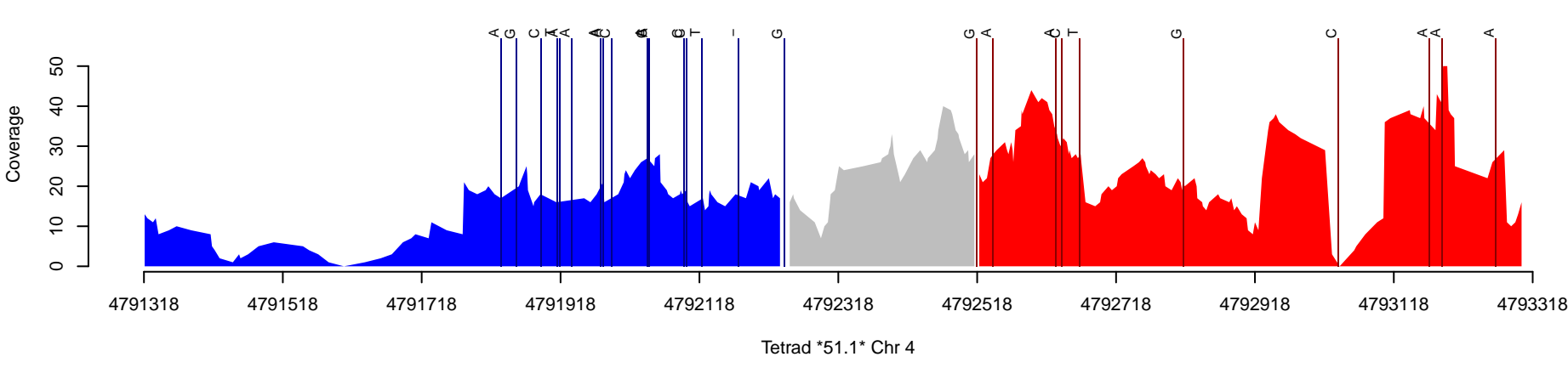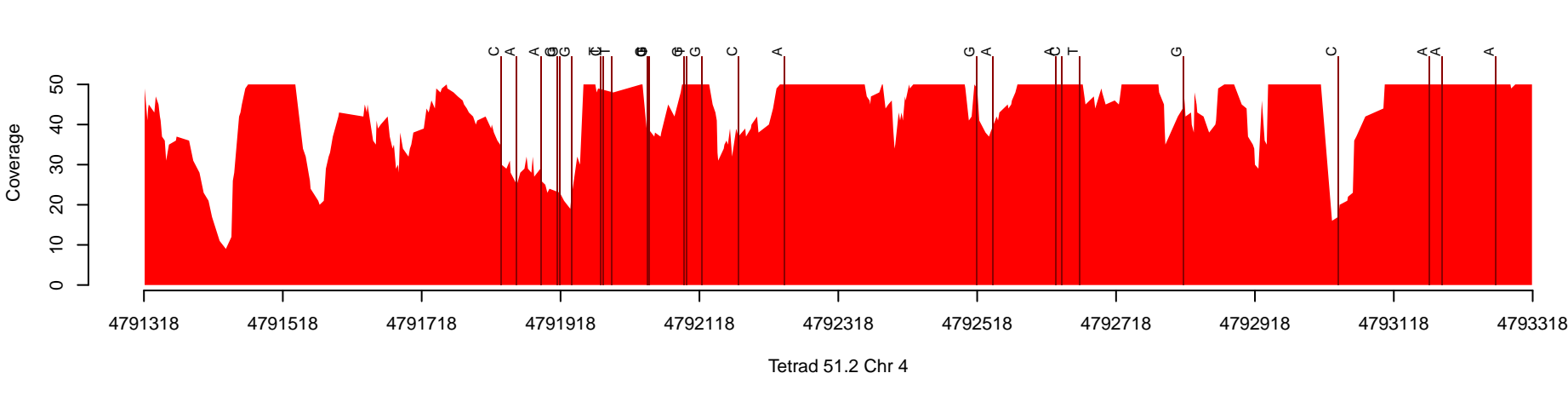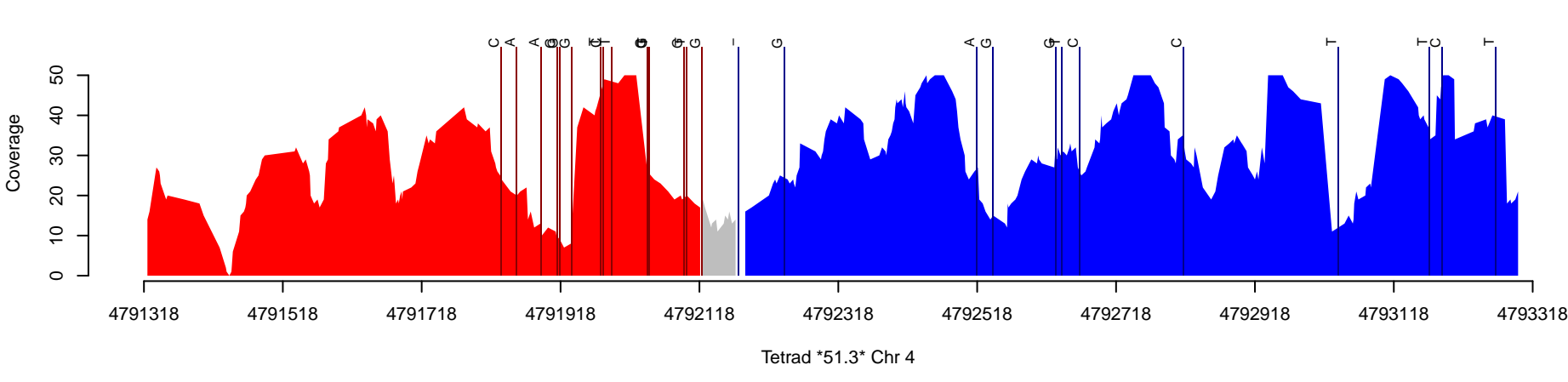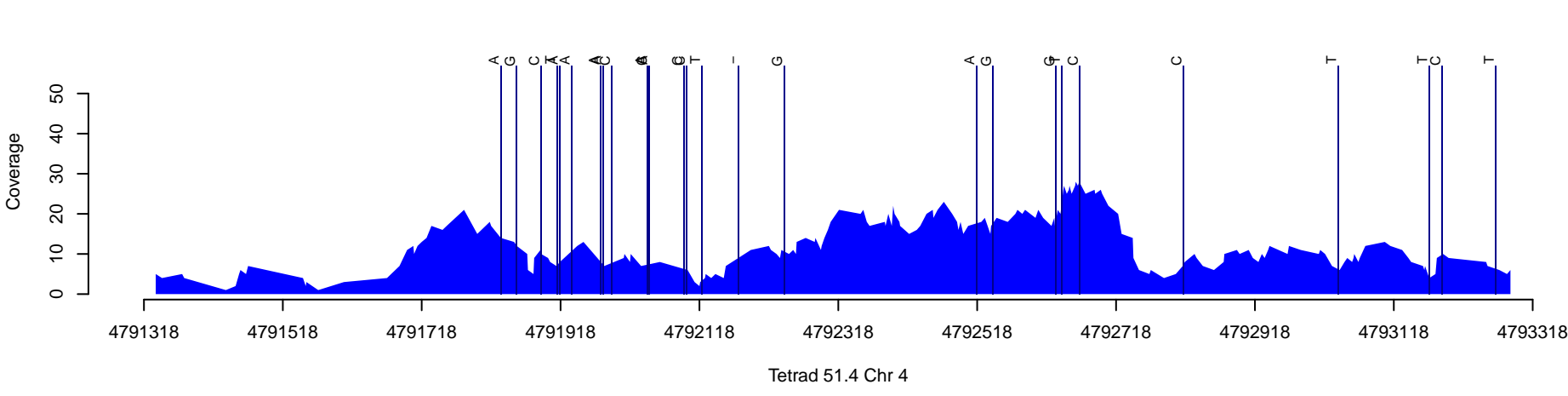

ID66

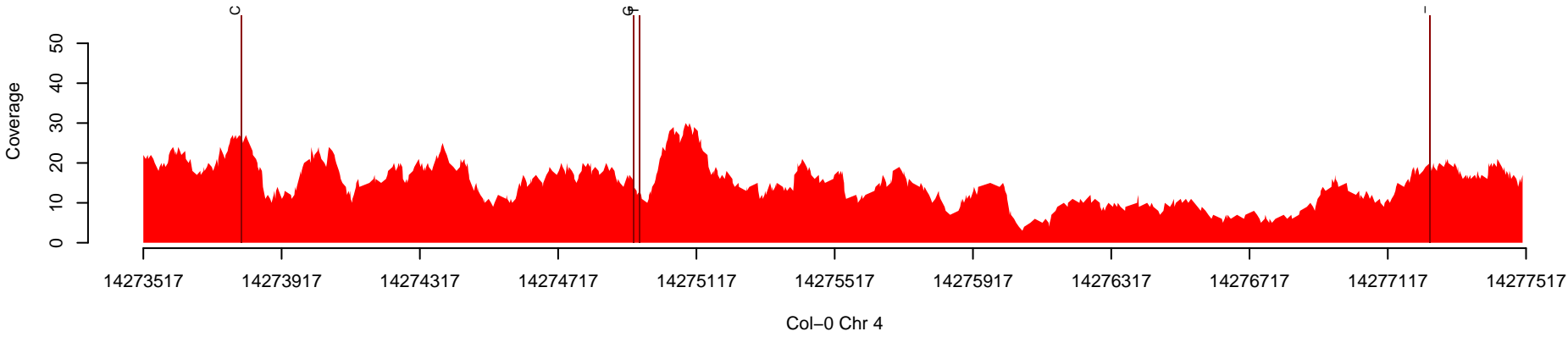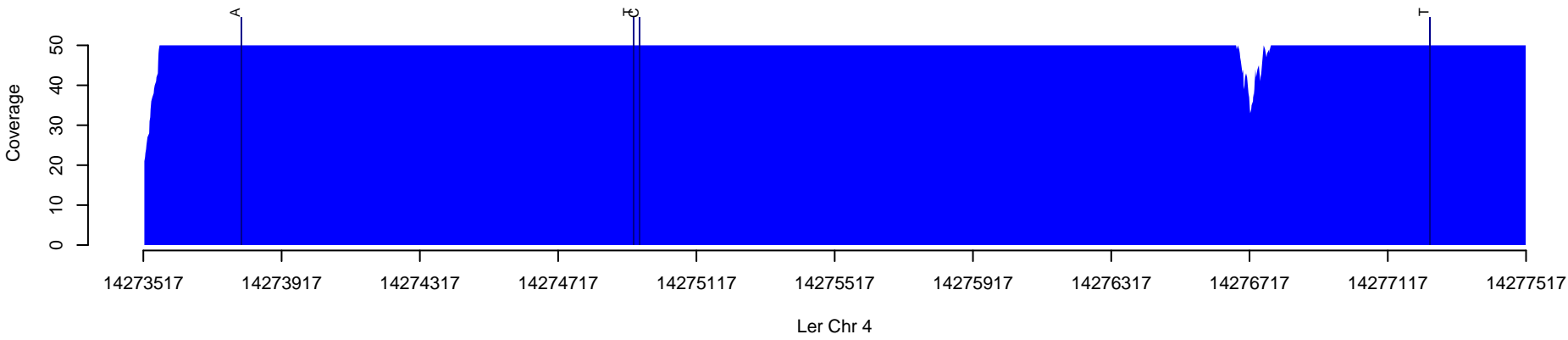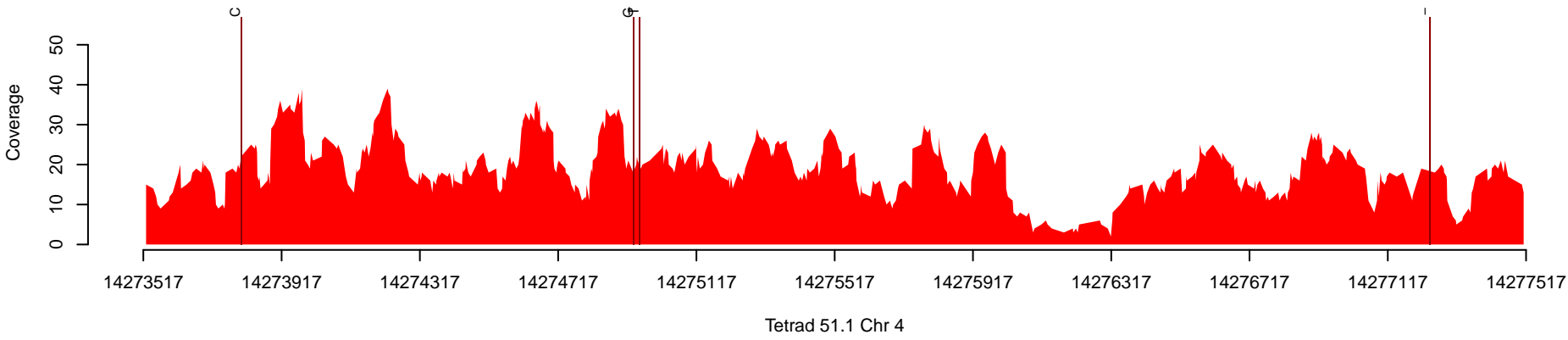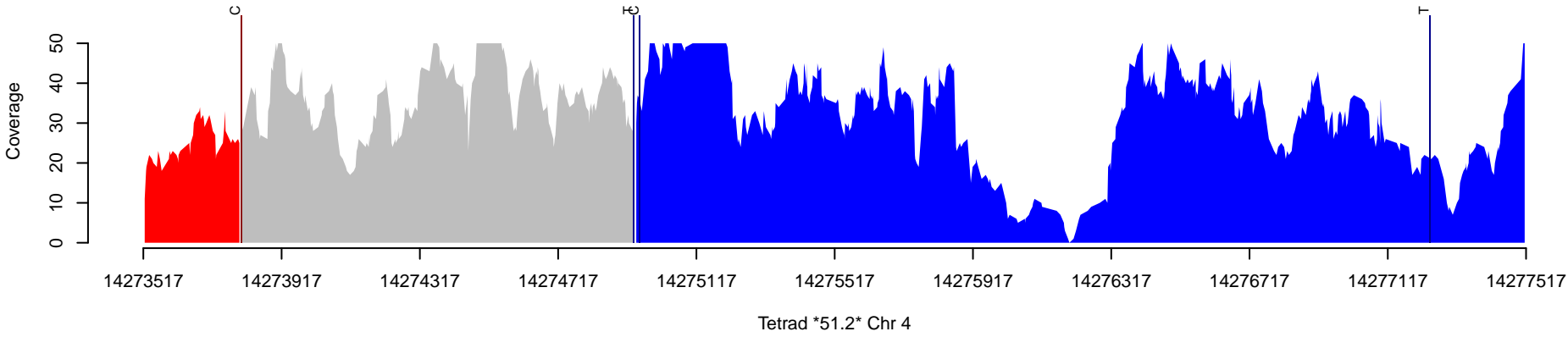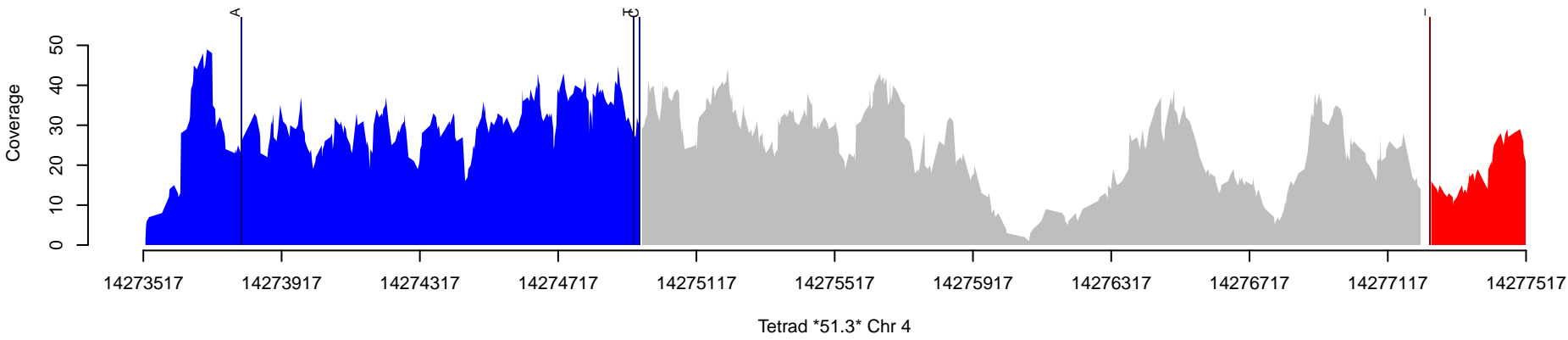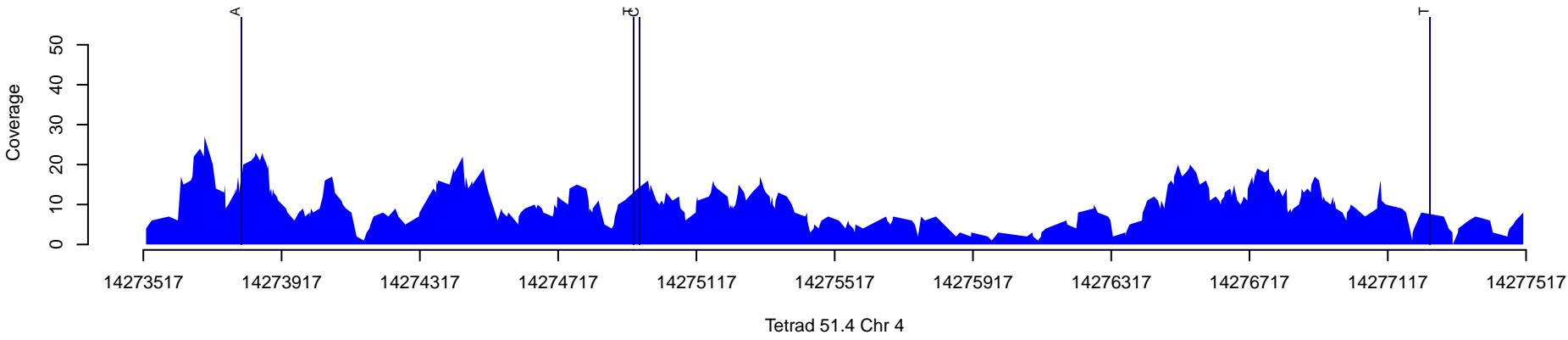

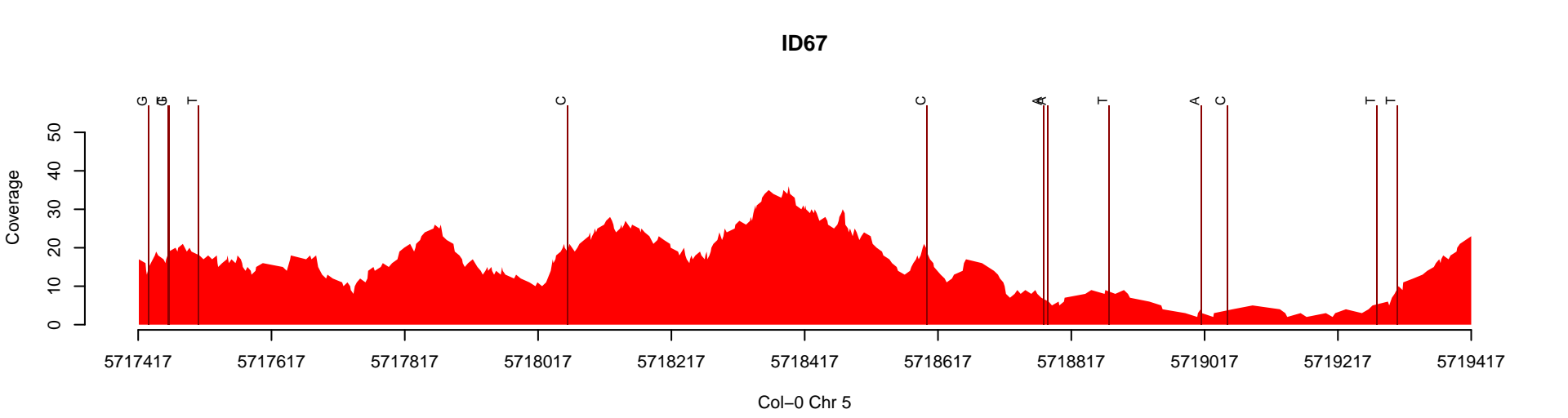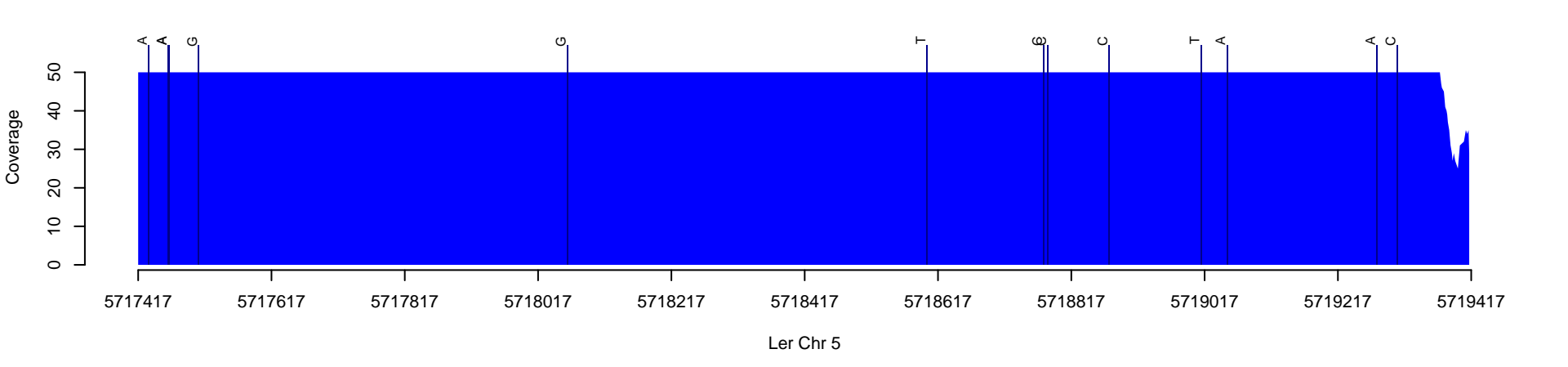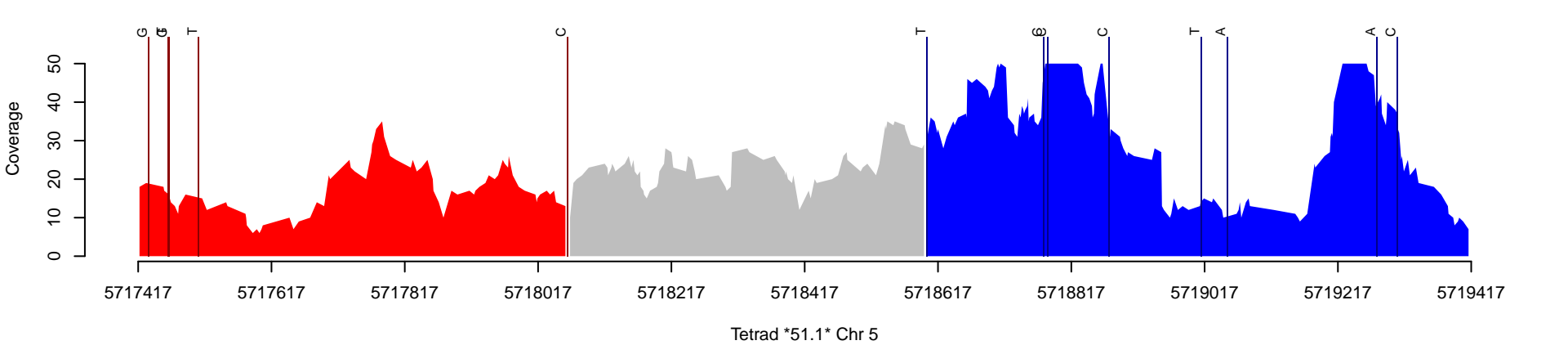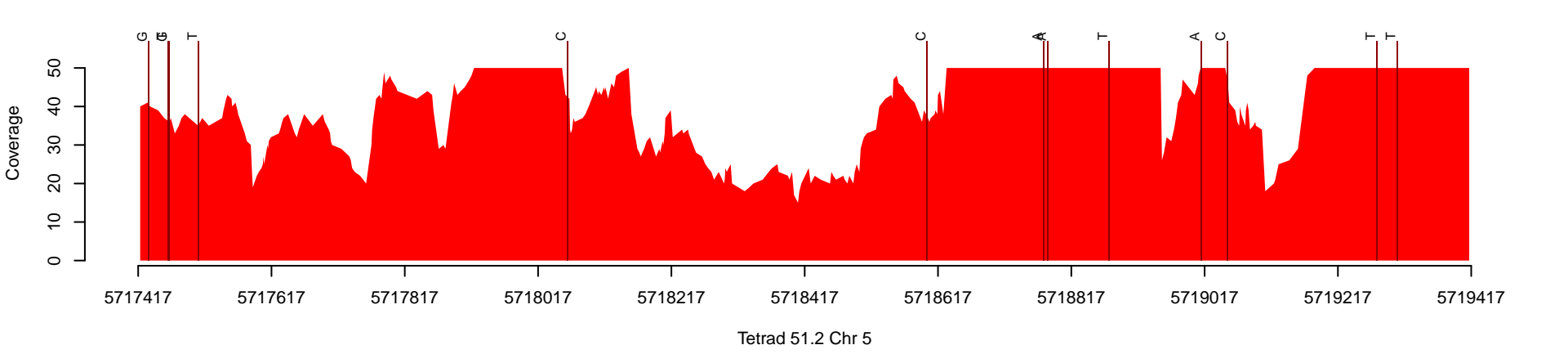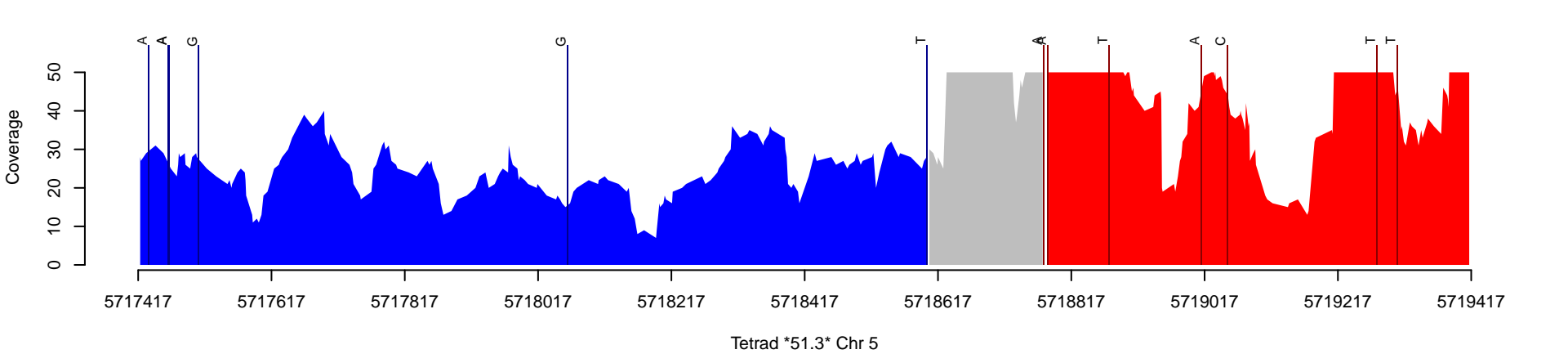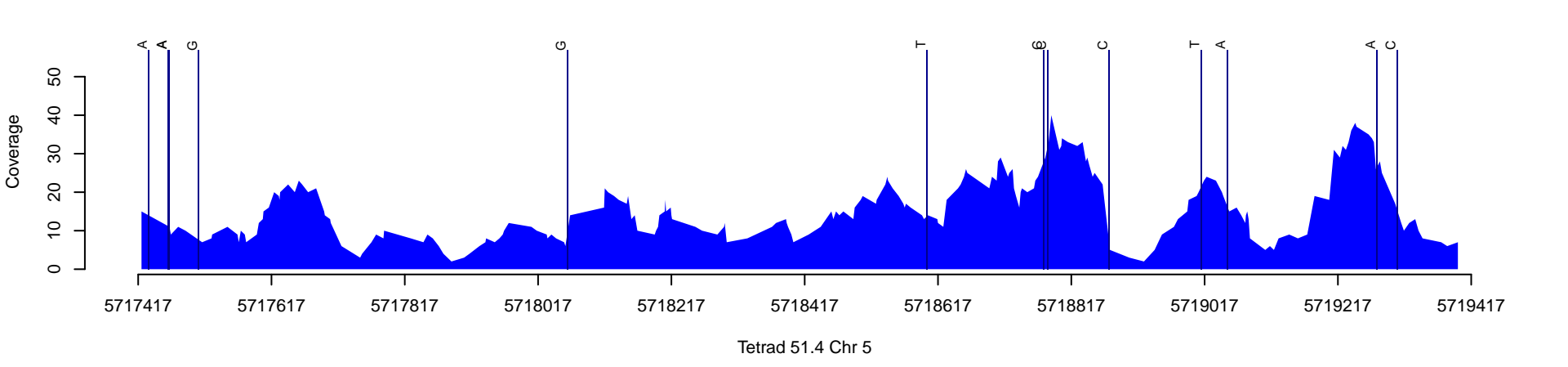

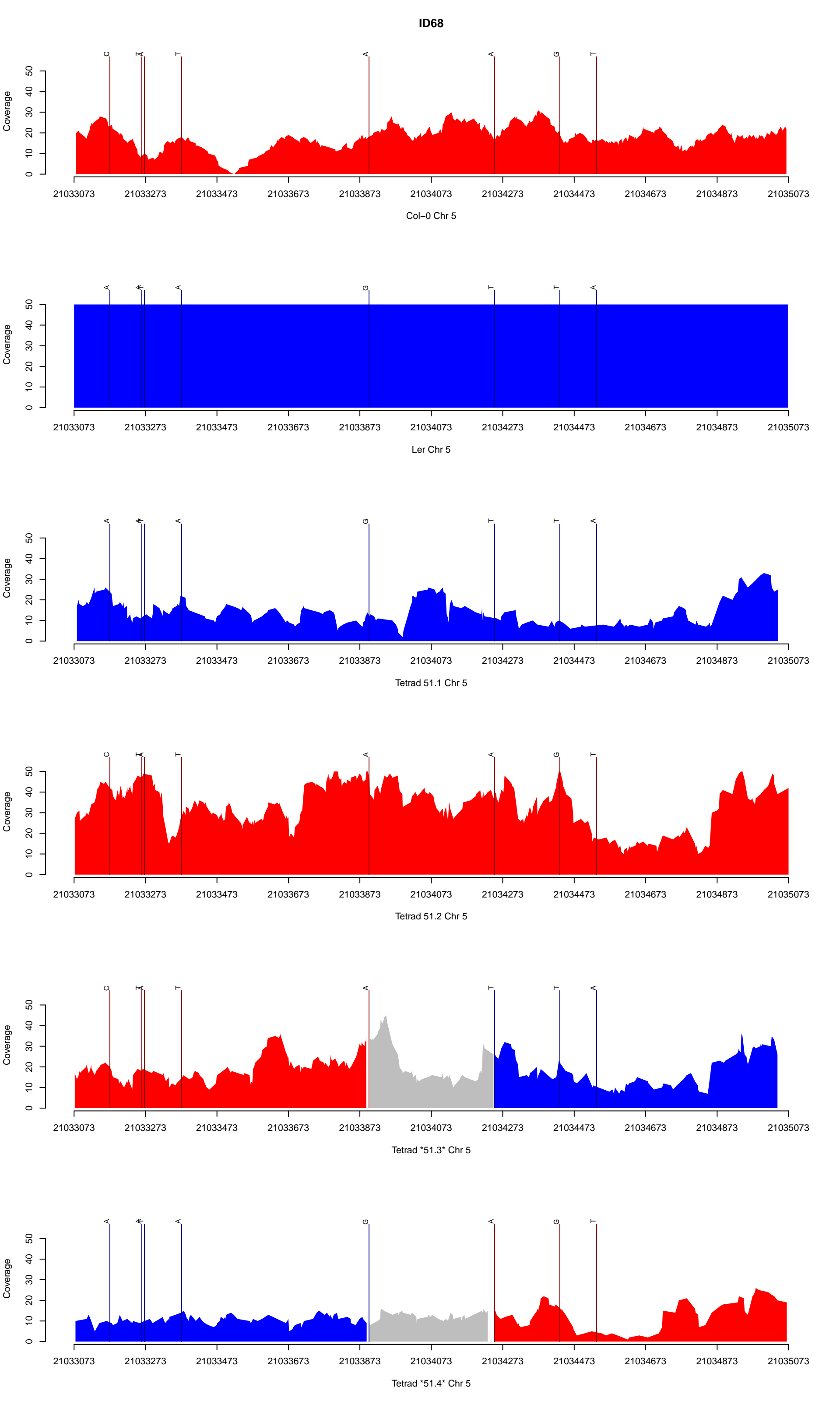

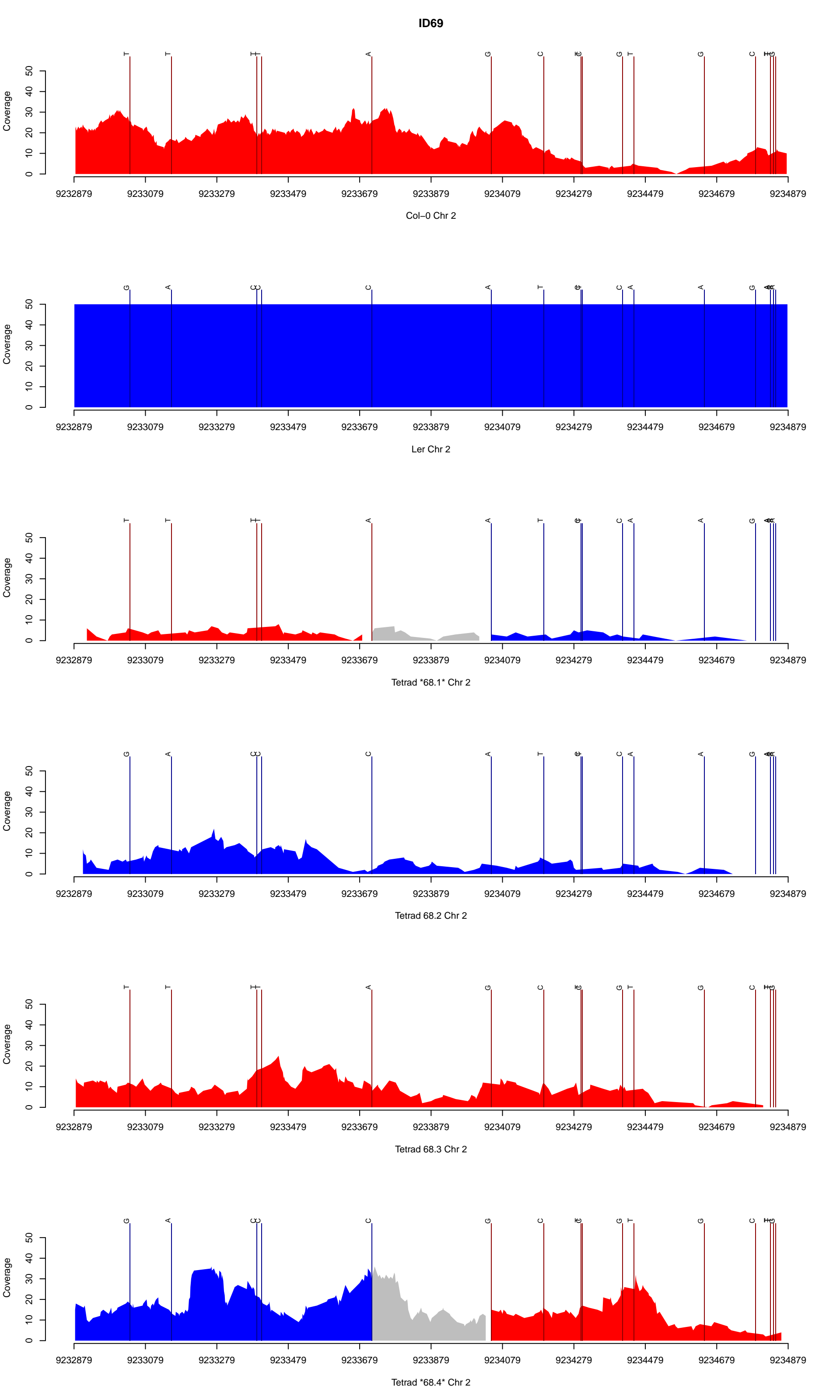

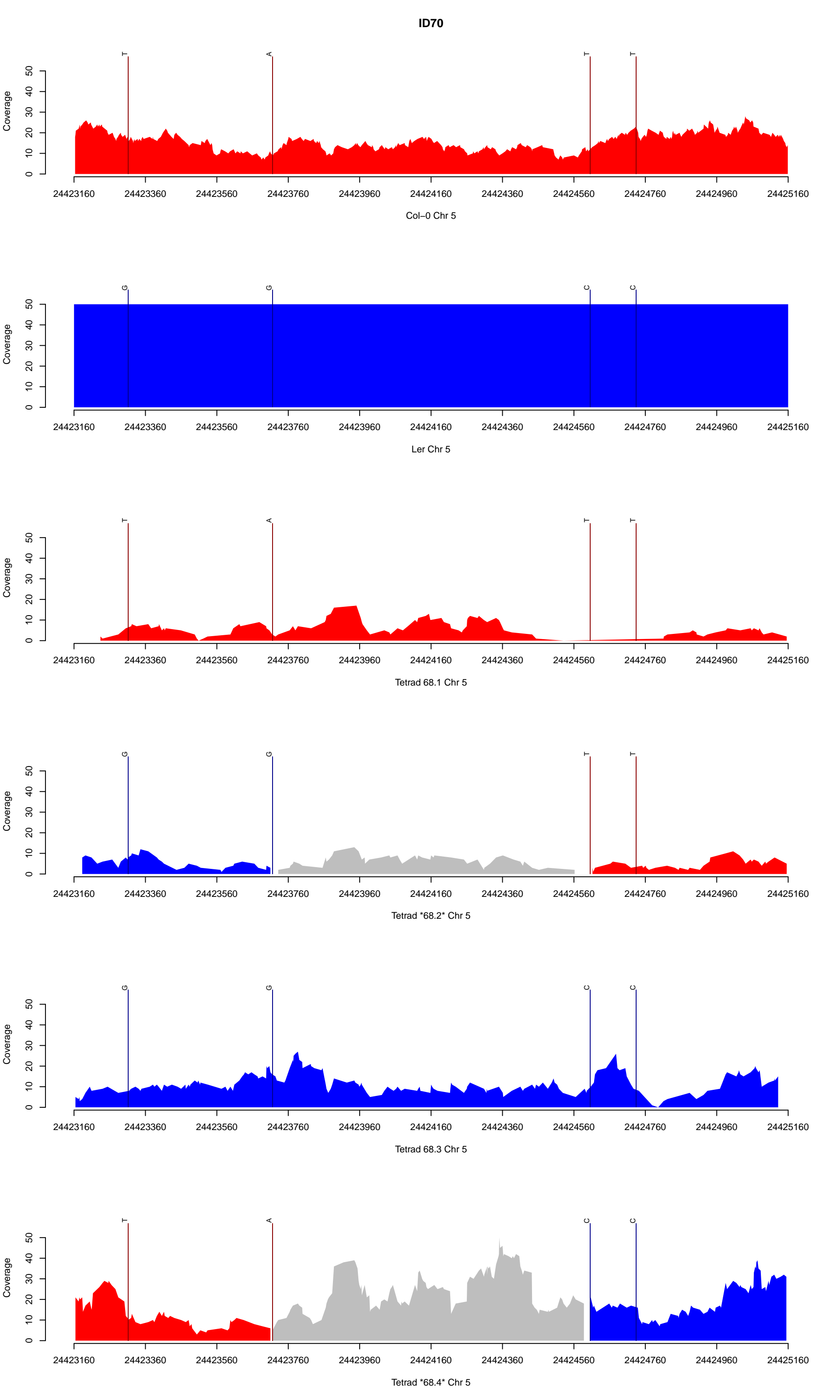

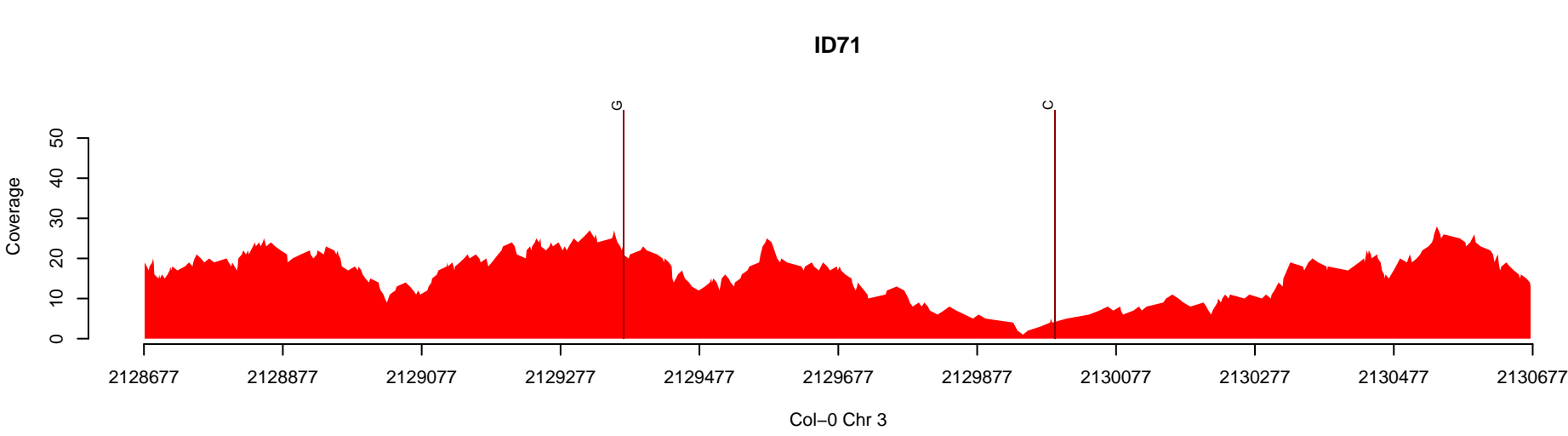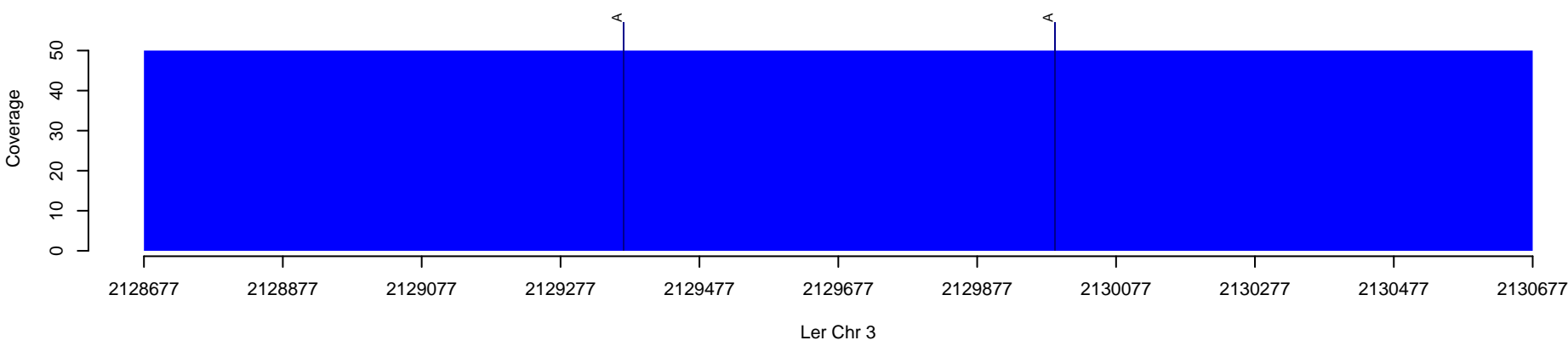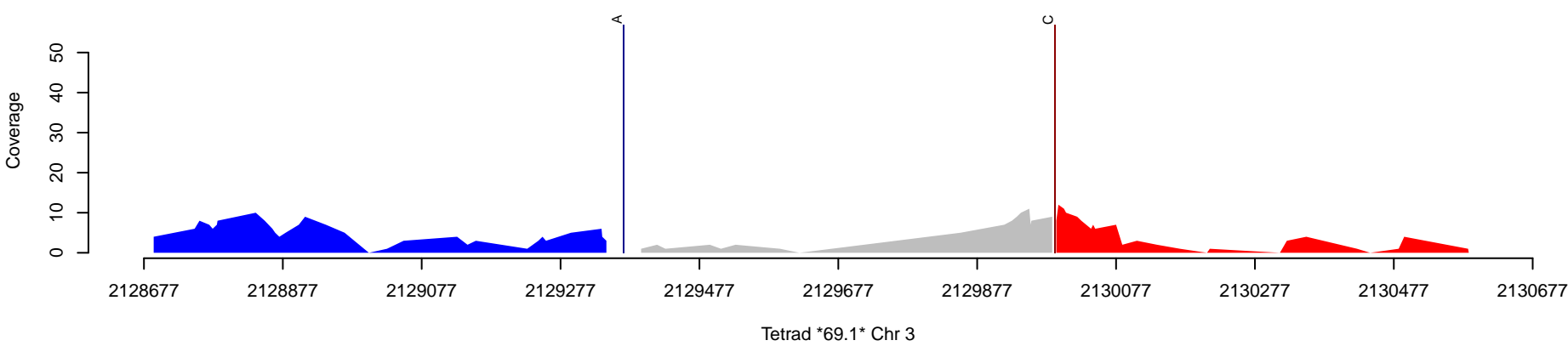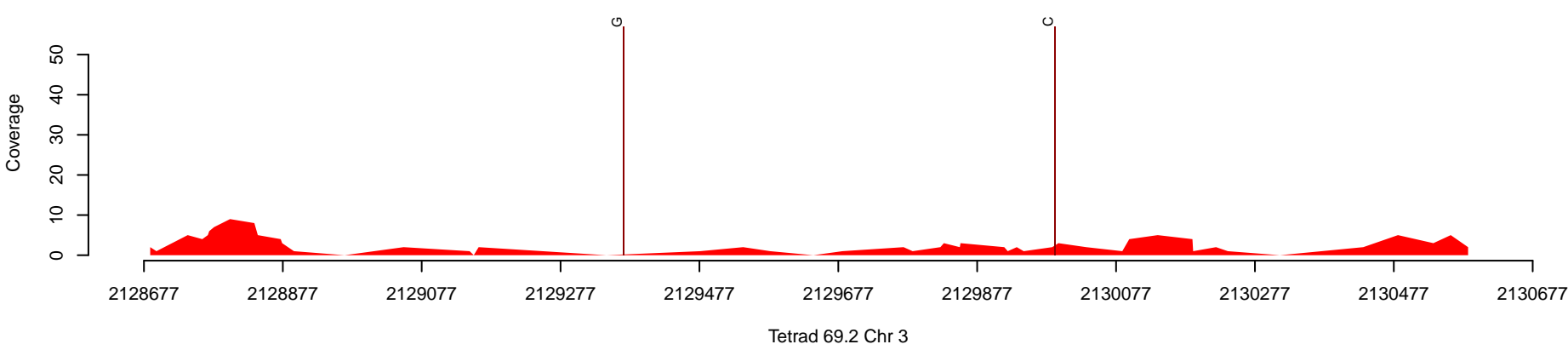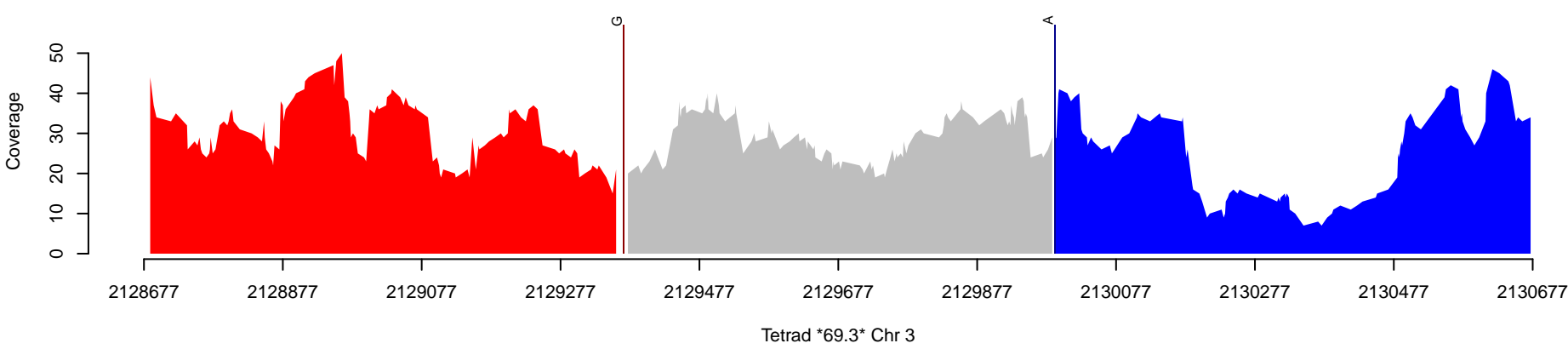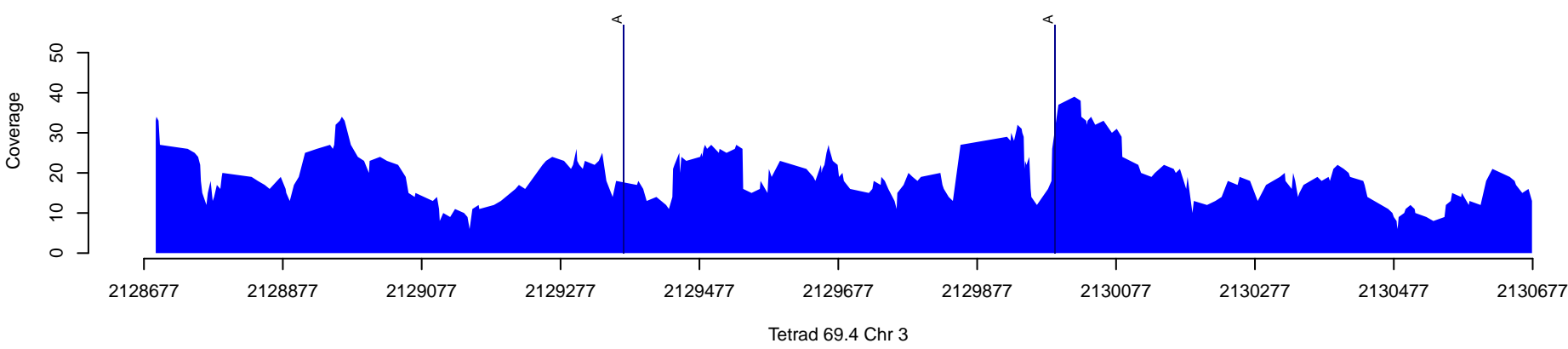

Supplement: Supplementary file 3. — Includes a visualization of the exact makeup of 71 COs identified in the tetrad samples, for which sufficient sequencing information was available. The colored areas indicate the number of short read alignments for each of the positions as indicated on the x-axis. Red and blue areas refer to regions that descended from Col-0 and Ler, respectively. Grey areas cannot be assigned to either of them. Vertical lines indicate sequence differences between the parental genotypes and have the respective genotype indicated next to them. Within the CO sites (between the outer borders of both grey areas) the polymorphism data are based on hand curated short read alignments and local assemblies. Outside the flanking markers the polymorphisms encompass the marker used for reconstructing the recombinant chromosomes only. Note each tetrad sample contains one recombinant chromosome and one that is derived from the Cvi parent. The Cvi alleles are not indicated in these plots. DOI: http://dx.doi.org/10.7554/eLife.01426.018 [file elife01426s003.pdf]
